# Incidence and death in 29 cancer groups in 2017 and trend analysis from 1990 to 2017 from the Global Burden of Disease Study

**DOI:** 10.1186/s13045-019-0783-9

**Published:** 2019-09-12

**Authors:** Longfei Lin, Lei Yan, Yuling Liu, Fang Yuan, Hui Li, Jian Ni

**Affiliations:** 1 Institute Chinese Materia Medica, China Academy of Chinese Medical Sciences, Beijing, China; 2 Fengtai District Community Health Center, Beijing, China; 3 School of Chinese Material Medica, Beijing University of Chinese Medicine, Beijing, China; 4 Beijing Hospital of Traditional Chinese Medicine, Capital Medical University, Beijing, China

**Keywords:** Global burden of disease, Cancer, Incidence and death, Trend analysis

## Abstract

**Background and aims:**

Cancer has become the second most serious disease threatening human health, followed by cardiovascular diseases. This study aimed to quantitatively estimate the mortality, morbidity, and analyze the trends of 29 cancer groups in 195 countries/regions between 1990 and 2017.

**Methods:**

Detailed information of 29 cancer groups were collected from the Global Burden of Disease (GBD) study in 2017 and age-standardized incidence rates (ASIR) and age-standardized death rates (ASDR) of 29 cancer groups were calculated based on gender, age, region, and country. Trend analyses were conducted for major cancer types.

**Results:**

In 2017, the global death population caused by cancer reached 9 million, which was nearly twice the number in 1990. The ASDR and ASIR of cancer in males were about 1.5 times those of females. Breast cancer showed the highest mortality rate in females in 2017. Individuals aged over 50 are at high risk of developing cancer and the number of cases and deaths in this age group accounted for more than 80% of all cancers in all age groups. Asia has the heaviest cancer burden due to its large population density. Different cancers in varied countries globally have their own characteristics. The ASDR and ASIR of some major cancers demonstrated changes from 1990 to 2017.

**Conclusions:**

Analyses of these data provided basis for future investigations to the common etiological factors, leading to the occurrence of different cancers, the development of prevention strategies based on local characteristics, socioeconomic and other conditions, and the formulation of more targeted interventions.

**Electronic supplementary material:**

The online version of this article (10.1186/s13045-019-0783-9) contains supplementary material, which is available to authorized users.

## Introduction

Due to population growth and aging, the morbidity and mortality associated with cancer are rapidly increasing globally, reflecting the changes in the prevalence and distribution of major risk factors of cancer [1, 2]. Additionally, the cancer spectrum is changing with increasing cancer cases. For example, cancers associated with infection and poverty were gradually replaced by cancers that are commonly found in developed countries (such as high-income countries of Europe, North America, Asia, and Oceania). However, differences exist in the types of cancers within the countries and regions and are mainly due to the differences in population risk factors caused by different stages of social and economic changes [3–5]. Over the past few decades, the survival rate of cancer patients has been improved significantly with the advancements in disease prevention, diagnosis, and treatment. However, owing to the growing population globally and increasingly prominent aging problem, cancer burden still displayed an increasing trend worldwide. In this study, the results of global burden of disease (GBD) 2017 study were utilized to demonstrate the burden of 29 cancer groups globally and trend analysis based on gender, age, and 195 countries/regions was also performed. In addition, the severe burden of cancers, such as breast cancer and liver cancer, that is associated with morbidity and mortality was analyzed from 1990 to 2017.

## Methods

### Data Source

Data regarding annual case numbers, death numbers, and the corresponding age-standardized rates of 29 cancer groups including different genders, regions, countries, and ages were collected from the GBD [6]. Data from 195 countries/regions were available and these countries and territories were divided into five regions according to the classification of sociodemographic index (SDI) of low, low-medium, medium, high-medium, and high regions [7]. Geographically, the world is divided into 21 regions (Table 1). Regional statistical analysis of morbidity and mortality benefitted studies on the epidemiology of malignant tumors, such as regional environment, national ethnicity, and living habits. The types of cancers were classified into 29 groups according to the International Classification of Diseases (ICD) and all the incidences of cancer were obtained either from a single cancer registry or aggregate database of cancer registry’s, which included the Cancer Incidence in Five Continents (CI5), Surveillance, epidemiology, and end results (SEER), or Nordic Cancer Registries database (NORDCAN). However, not all countries have a tumor registry system to cover the entire population. In most of the developing countries, tumor registration covers only few areas and underdeveloped areas do not have any tumor registries. Thus, the data from the areas with tumor registries were analyzed in our study. Also, based on the World Bank’s Human Development Index (HDI), the global socioeconomic development levels are divided into four levels, high, upper-middle, lower-middle, and low, and the correlation between cancer distribution and HDI differences was investigated [8, 9].

**Table 1:** The global death and incidence of all cancers and 29 specified cancer groups in 1990 and 2017

Tumor types	1990				2017			
	Death		Incidence		Death		Incidence	
	Number 10^3^(95% UI)	Age-standardized per 100,000 (95% UI)	Number 10^3^(95% UI)	Age-standardized per 100,000 (95% UI)	Number 10^3^(95% UI)	Age-standardized per 100,000 (95% UI)	Number 10^3^(95% UI)	Age-standardized per 100,000 (95% UI)
Total-Neoplasms	5753 (5903–5661)	142.5 (145.89–140.47)	12,144 (14146–10595)	296.09 (349.12–255.32)	9556 (9692–9396)	121.21 (122.92–119.12)	24,362 (27310–21911)	306.75 (343.47–275.8)
Esophageal cancer	311 (323–301)	7.72 (8.01–7.48)	310 (322–301)	7.57 (7.85–7.33)	436 (448–425)	5.48 (5.63–5.34)	473 (485–459)	5.9 (6.06–5.74)
Stomach cancer	769 (795–752)	19.32 (19.96–18.91)	864 (890–847)	21.32 (21.95–20.9)	865 (885–848)	10.98 (11.23–10.77)	1221 (1255–1189)	15.36 (15.78–14.97)
Liver cancer	453 (490–426)	10.8 (11.68–10.18)	472 (512–445)	11.05 (11.97–10.42)	819 (856–790)	10.21 (10.65–9.85)	953 (997–917)	11.8 (12.34–11.35)
Larynx cancer	94 (97–91)	2.27 (2.34–2.2)	133 (136–129)	3.14 (3.22–3.06)	126 (130–123)	1.57 (1.61–1.53)	211 (216–206)	2.59 (2.65–2.54)
Tracheal, bronchus, and lung cancer	1033 (1063–1015)	25.54 (26.25–25.1)	1079 (1110–1060)	26.34 (27.05–25.89)	1883 (1923–1844)	23.74 (24.24–23.25)	2163 (2213–2117)	27.13 (27.75–26.55)
Breast cancer	350 (381–333)	8.66 (9.37–8.28)	879 (936–846)	20.91 (22.16–20.19)	612 (641–589)	7.65 (8.01–7.37)	1961 (2023–1891)	24.19 (24.96–23.34)
Cervical cancer	189 (210–173)	4.45 (4.96–4.1)	416 (466–383)	9.1 (10.17–8.4)	260 (269–241)	3.21 (3.32–2.98)	601 (625–554)	7.38 (7.67–6.81)
Uterine cancer	59 (61–56)	1.48 (1.54–1.43)	184 (189–178)	4.41 (4.53–4.28)	85 (87–83)	1.07 (1.1–1.05)	407 (418–397)	5 (5.14–4.88)
Prostate cancer	208 (235–165)	5.95 (6.66–4.68)	478 (533–359)	12.67 (14.06–9.49)	416 (490–357)	5.5 (6.46–4.71)	1334 (1698–1171)	16.94 (21.53–14.84)
Colon and rectum cancer	503 (526–488)	13.31 (13.87–12.96)	826 (855–807)	21.2 (21.87–20.75)	896 (916–876)	11.52 (11.77–11.27)	1833 (1873–1792)	23.22 (23.73–22.7)
Lip and oral cavity cancer	97 (102–94)	2.38 (2.47–2.29)	186 (192–180)	4.41 (4.55–4.28)	194 (202–185)	2.42 (2.52–2.31)	390 (404–374)	4.84 (5.02–4.65)
Nasopharynx cancer	52 (54–49)	1.19 (1.25–1.13)	87 (93–81)	1.88 (2–1.76)	70 (72–67)	0.86 (0.89–0.82)	110 (116–104)	1.35 (1.42–1.28)
Other pharynx cancer	56 (61–49)	1.32 (1.43–1.16)	78 (83–70)	1.8 (1.93–1.63)	117 (124–102)	1.45 (1.53–1.26)	179 (189–160)	2.19 (2.3–1.96)
Gallbladder and biliary tract cancer	106 (119–101)	2.79 (3.14–2.68)	120 (133–115)	3.13 (3.46–3.02)	174 (185–154)	2.23 (2.38–1.99)	211 (225–186)	2.71 (2.89–2.39)
Pancreatic cancer	196 (200–193)	5.1 (5.18–5.02)	195 (199–192)	5.01 (5.1–4.94)	441 (449–433)	5.62 (5.72–5.52)	448 (456–439)	5.69 (5.8–5.57)
Malignant skin melanoma	34 (43–28)	0.85 (1.07–0.71)	118 (147–93)	2.74 (3.42–2.17)	62 (70–48)	0.78 (0.89–0.61)	309 (366–238)	3.87 (4.58–2.97)
Non-melanoma skin cancer	30 (32–29)	0.83 (0.86–0.79)	3751 (5756–2235)	95.39 (149.21–55.03)	65 (66–63)	0.85 (0.87–0.82)	7664 (10570–5251)	97.11 (134.21–66.66)
Ovarian cancer	96 (101–92)	2.37 (2.49–2.28)	152 (162–145)	3.54 (3.76–3.4)	176 (181–171)	2.2 (2.26–2.14)	286 (295–278)	3.54 (3.66–3.44)
Testicular cancer	7 (7–6)	0.14 (0.15–0.13)	40 (41–38)	0.75 (0.78–0.72)	8 (8–7)	0.1 (0.1–0.09)	71 (74–69)	0.9 (0.94–0.87)
Kidney cancer	68 (71–63)	1.69 (1.75–1.56)	207 (220–189)	4.72 (4.95–4.29)	139 (143–129)	1.77 (1.82–1.64)	393 (405–371)	4.94 (5.08–4.66)
Bladder cancer	115 (118–108)	3.15 (3.24–2.99)	249 (256–237)	6.42 (6.58–6.13)	197 (206–192)	2.57 (2.69–2.51)	474 (492–462)	6.04 (6.27–5.9)
Brain and nervous system cancer	142 (171–117)	3.04 (3.58–2.56)	194 (234–159)	3.97 (4.71–3.33)	247 (265–213)	3.12 (3.34–2.68)	405 (443–351)	5.17 (5.64–4.46)
Thyroid cancer	22 (24–21)	0.55 (0.6–0.52)	95 (101–90)	2.11 (2.24–2.01)	41 (44–40)	0.52 (0.56–0.51)	255 (272–246)	3.15 (3.36–3.03)
Mesothelioma	17 (21–14)	0.44 (0.52–0.37)	21 (25–18)	0.52 (0.62–0.43)	30 (31–29)	0.38 (0.39–0.37)	35 (36–34)	0.44 (0.45–0.42)
Hodgkin lymphoma	36 (39–27)	0.75 (0.83–0.58)	73 (79–56)	1.43 (1.56–1.1)	33 (38–28)	0.41 (0.48–0.35)	101 (119–88)	1.29 (1.51–1.12)
Non-Hodgkin lymphoma	135 (142–130)	3.19 (3.34–3.1)	207 (217–200)	4.75 (4.93–4.62)	249 (253–243)	3.18 (3.24–3.11)	488 (497–479)	6.18 (6.29–6.06)
Multiple myeloma	52 (61–48)	1.35 1.59–1.26)	65 (77–60)	1.65 (1.93–1.52)	107 (119–99)	1.37 (1.51–1.25)	153 (173–141)	1.92 (2.17–1.76)
Leukemia	265 (293–234)	5.81 (6.32–5.27)	354 (399–308)	7.42 (8.17–6.62)	348 (365–317)	4.5 (4.73–4.12)	518 (548–472)	6.76 (7.16–6.15)
Other malignant neoplasms	220 (229–206)	5.07 (5.25–4.79)	308 (325–287)	6.74 (7.02–6.33)	360 (371–331)	4.61 (4.75–4.25)	716 (740–656)	9.16 (9.47–8.4)

*UI* uncertainty interval

### Statistical analyses

The age-standardized incidence rate (ASIR) and age-standardized death rate (ASDR) were adopted to quantify the trends associated with morbidity and mortality in the 29 cancer groups in various regions [10]. The ASIR or ASDR (per 100,000 population) was calculated according to the direct method by summing up the products of age-specific rates (*α*_*i*_, where *i* denotes the *i*th age class) and the number of persons (*β*_*i*_) in the same age subgroup *i* of the chosen reference standard population, followed by dividing the sum of the standard population weights, i.e., [11].

$$ \mathrm{ASIR},\mathrm{ASDR}=\frac{\sum_{i=1}^A{\alpha}_i{\beta}_i}{\sum_{i=1}^A{\beta}_i}\times \mathrm{100,000} $$

Standardization was considered imperative for this study as it eliminates the bias when comparing the ratios or rates. For instance, standardization can eliminate the influence of internal differences (such as gender, age, etc.) in the subjects of two groups, and subsequently allows the analysis of any substantive differences. The age-standardized rate (per 100,000 populations) was calculated by direct methods (in which the actual rates of all groups of comparative data are known, and the rates are standardized by standard population or standard population composition) in this study. The global cancer incidence from 1990 to 2017 was calculated by using the Joinpoint regression model. The National Cancer Institute (NCI) Joinpoint regression program software (version 4.1.0, Statistical Research and Applications Branch, NCI) was used to conduct the trend-change analyses and identify statistically significant differences, which in turn assists in calculating the annual percentage change (APC) for each trend phase.

## Results

### Overall status of global cancer burden in 2017

The population incidence, standardization rate, and death number with cancers globally and in the 29 cancer groups from 1990 and 2017 are presented in Table 1. In 2017, the global death number caused by tumor exceeded 9 million [9453325 (9,835,057–9,028,666)]. Although the death number was almost doubled when compared to 1990, the ASDR showed a downward trend and the ASIR showed an increasing tendency. According to the 2017 cancer data, cancers with highest mortality rates included tracheal, bronchus, and lung (TBL) cancer, colon and rectal cancer, stomach cancer, and liver cancer. The ASDRs for these cancers were more than 10, with values of 23.74 (24.24–23.25), 11.52 (11.77–11.27), 10.98 (11.23–10.77), and 10.21 (10.65–9.85), respectively. The number of deaths attributed to these cancers were 1,883,066 (1,922,809–1,844,246), 896,040 (915,720–876,279), 864,989 (884,655–848,254), and 819,435 (855,505–789,721), respectively. Nearly 75% (74.8%) of cancer deaths globally in 2017 were caused by ten cancers. These ten cancers were ranked in descending order, which included TBL, colon and rectal, stomach, liver, breast, pancreatic, esophageal, prostate cancers, other malignant neoplasms, and leukemia. TBL cancer was considered as the most deadliest cancer, accounting for 19.9% of the total number of cancer deaths. Four cancers (TBL, colon and rectal, stomach, and liver cancers) also had higher ASIRs with values of 27.13 (27.75–26.55), 23.22 (23.73–22.7), 15.36 (15.78–14.97), and 11.8 (12.34–11.35), respectively. In addition, the ASIRs of breast cancer, prostate cancer, and non-melanoma skin cancer were also higher than 10.

In contrast to 1990, the ASIR of thyroid cancer was considered to be the most significant increase (49.3%). Moreover, the ASIRs of prostate cancer, malignant skin melanoma, brain and nervous system cancer, thyroid cancer, and non-Hodgkin lymphoma were increased by over 30%. The ASIR of breast cancer was 24.19 (24.96–23.34), which was increased by 15.7% when compared to 1990. Among all the cancer groups, non-melanoma skin cancer demonstrated a high incidence rate of 97.11 (134.21–66.66) but showed a low mortality rate of 0.85 (0.87–0.82).

### Global cancer burden affected by gender and age

**Fig. 1:**
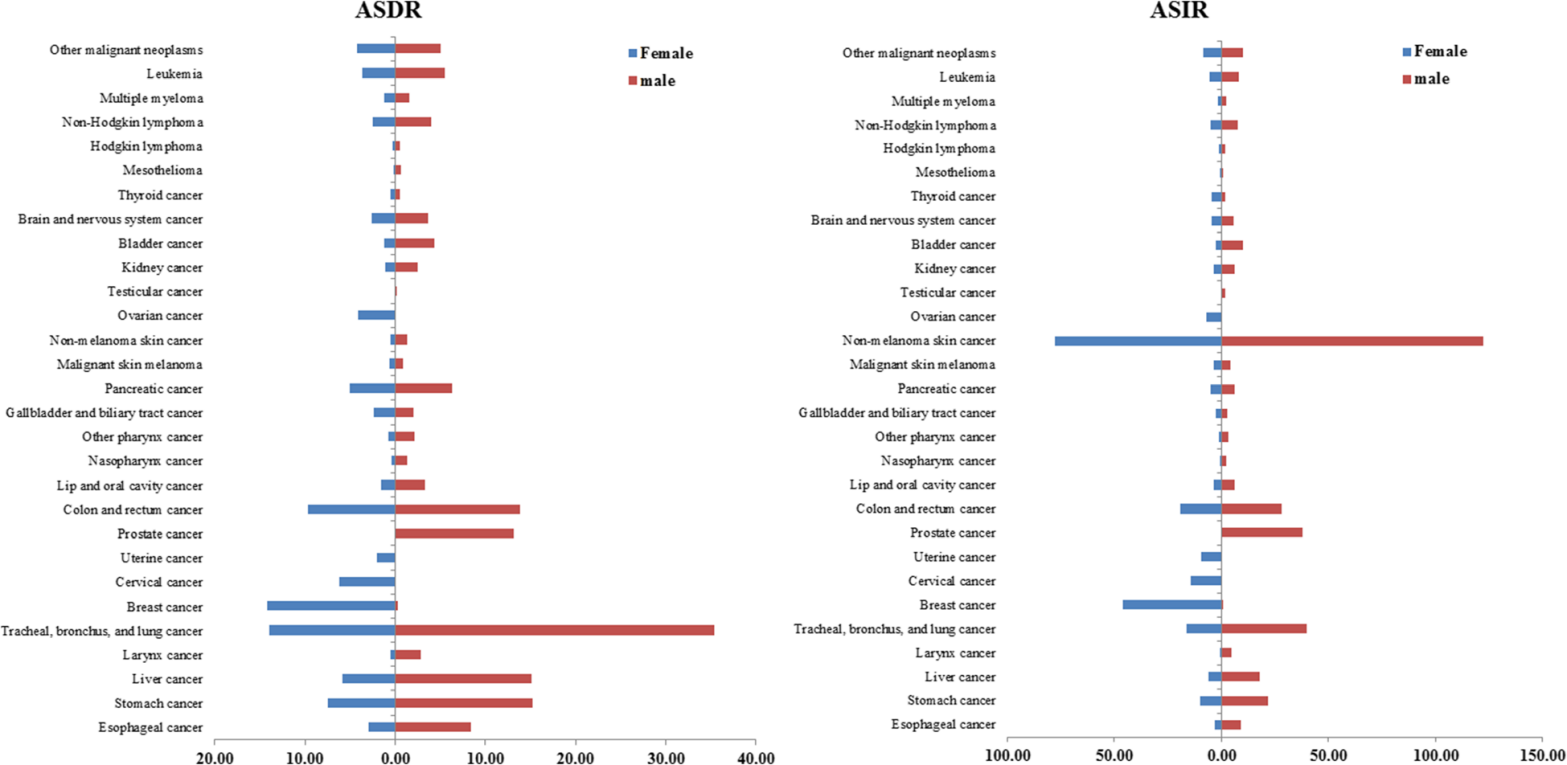
The age standardized global deaths and incidence of 29 specified cancer groups in 2017 by gender

In terms of gender, the morbidity and mortality of cancer in males were higher than that of females and the value for males was 1.5 times higher than that of females (Fig. 1 and Additional file 1: Tables S1 and S2). In 2017, the total number of global deaths due to cancer in males was 5,390,260 (5,690,016–5,071,487) and in females was 4,063,665 (4,239,167–3,847,191). In males, TBL cancer demonstrated highest ASDR [35.38 (36.35–34.39)], with 1,286,779 deaths (1,322,428–1,249,980), and this accounted for 23.9% of total deaths associated with cancers in females. The ASDRs of stomach cancer [15.19(15.67–14.77)], liver cancer [15.10(16.08–14.37)], colon and rectal cancer [13.79(14.22–13.30)], and prostate cancer [13.11(15.33–11.18)] were next to TBL cancer in males. The ASIRs of prostate cancer in males were high at 37.86 (47.99–33.03), which was secondary to non-melanoma skin cancer [122.15(170.28–83.87)] and TBL cancer [39.90(41.13–38.72)]. The ASDR of breast cancer remained the highest of all female malignancies. In 2017, the global number of deaths occurred due to breast cancer was 600,728 (629,932–578,725), accounting for 14.8% of all the total deaths caused by varied types of cancers in females. In addition, cervical cancer, uterine cancer, and ovarian cancer were also associated with high mortality rates. The morbidity and mortality of cancer in males were also higher than that of females in North America, especially in esophageal cancer, laryngeal cancer, and other cancer such as pharyngeal cancer, non-melanoma skin cancer, bladder cancer, and mesothelioma. The ASDR of these in males were three times that of women.

**Fig. 2:**
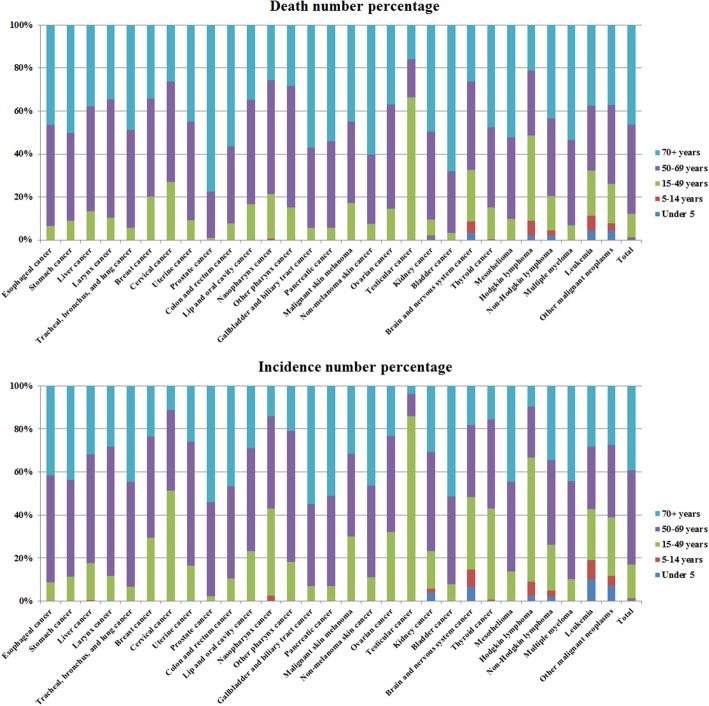
Age-specific global contributions of cancer types to total cancer incidence in 2017

The cancer data of different age groups in 2017 was shown in Fig. 2 (details in Additional file 1: Table S3). People aged over 50 years were at high risk of developing cancer. The number of cancer patients and deaths accounted for more than 80% in all age groups. The number of people over 50 years who died with esophageal, stomach, TBL, uterine, colon and rectal, prostate, gallbladder, and biliary tract, as well as bladder cancers accounted for more than 90% of total deaths. The incidence of testicular cancer demonstrated to be the highest in 15- to 49-year-old group. The number of new incidences of testicular cancer cases in this age group was close to 90% of the total new cases, and the number of deaths in this age group accounted for more than 60% of the total deaths. The incidence and death rates in children with cancer (under 15 years old) remained low (less than 1% of the total numbers) and there were fewer types of cancers present in children than in adults. The types of cancers in children below 5 years of age mainly included kidney cancer, brain, and nervous system cancers, Hodgkin lymphoma, non-Hodgkin lymphoma, leukemia, and other malignant neoplasms. Liver cancer and nasopharyngeal cancer were also found in children of 5–14 years age.

### Global cancer burden in different regions

**Fig. 3:**
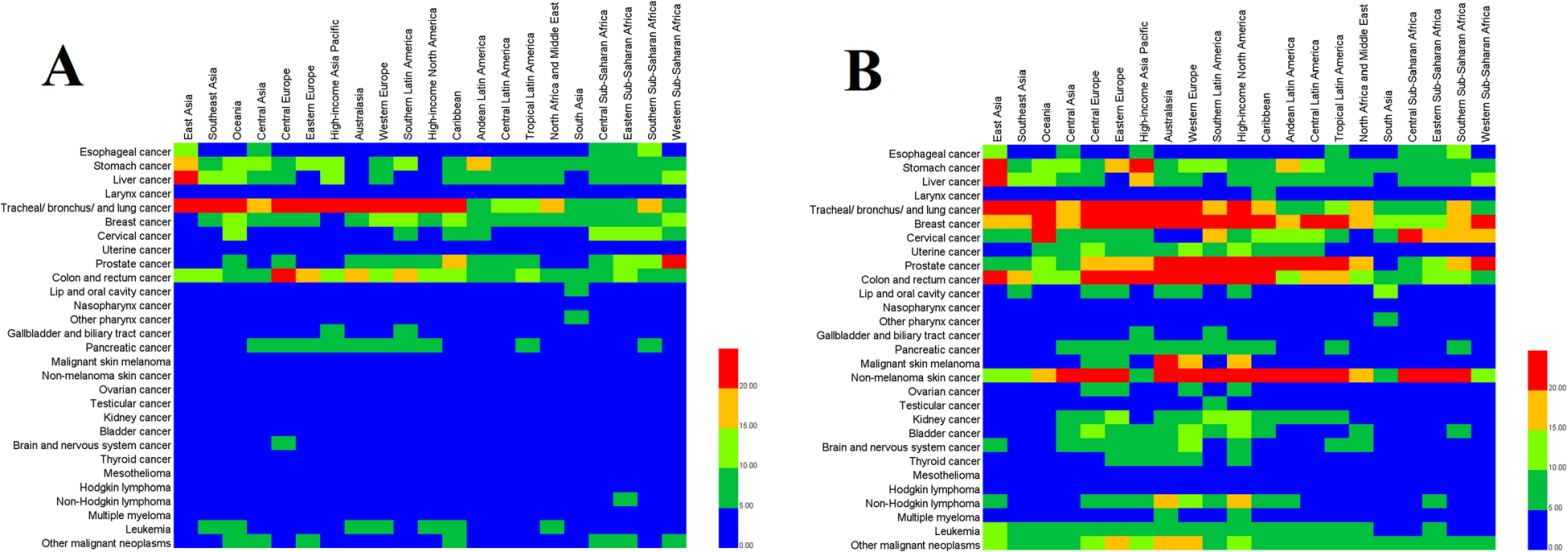
Cancers ranked by age-standardized deaths and incidence in 21 regions in 2017. **a** Age-standardized deaths per 100,000. **b** Age-standardized incidence per 100,000)

The number of deaths and incidences, as well as the ASDRs and ASIRs of 29 cancer groups in 21 regions in 2017 were presented in a table format and the specific results are shown in Fig. 3 (details in Additional files 2, 3, 4, 5, 6, and 7). Asia had the highest total number of cancer cases due to its high population density. According to the GBD, the total population in East Asia is 1.486 billion, which accounted for 19.45% of the global population. In 2017, there were 2,731,480 (2,892,040–2,542,159) total deaths due to cancer in East Asia, accounting for 28.9% of the world’s total number of deaths. This was much higher than its population proportion. South Asia and Western Europe followed East Asia, in which the global cancer death proportions included 12.8% and 12.3%, respectively. With regard to different types of cancers, East Asia also had high cancer burdens. The ASDR for esophageal, stomach, liver, TBL, and nasopharyngeal cancers in East Asia accounted for more than 30% of the total globally of the corresponding cancer groups, wherein liver cancer was shown to be the highest (54.1%), followed by esophageal cancer (over 50%). The ASDRs for these cancers accounted for 70.9% of the total cancer groups’ ASDRs in East Asia, and similar results were observed for ASIRs. South Asia also comprises a high proportion of the world population, which shares 1.783 billion people of 23.3% global people. The ASDRs of larynx, lip and oral cavity, and other pharyngeal cancers in South Asia accounted for 35.0%, 47.7%, and 58.8% of the global total ASDRs in the same cancer group, respectively. However, the characteristics of cancer in different regions of Asia varied as well. For example, although liver cancer accounted for 54% of global deaths in East Asia, it only accounted for 5.6% of the world’s total death number in South Asia, which was only about one-tenth that of the East Asia. Also ASDR of lip and oral cavity cancer in South Asia was about four times that of the East Asia. The three highest TBL cancer burden regions was East Asia, Central Europe, and high-income North America, and ASDRs were 35.94(37.34–34.41), 34.30(35.35–33.25) and 34.09(34.99–33.25), respectively.

**Fig. 4:**
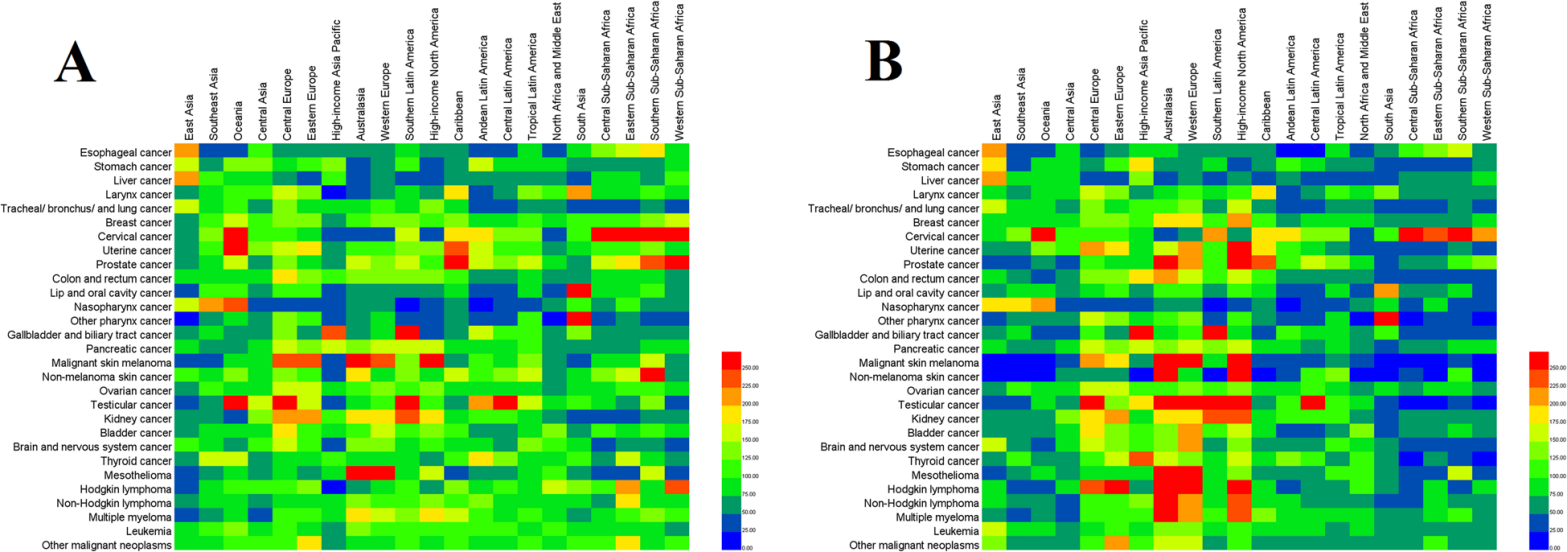
The age-standardized deaths and incidence of cancers in 21 regions compared with those globally in 2017. **a** Comparison of age-standardized deaths. **b** Comparison of age-standardized incidence

The ASDR and ASIR of 29 cancer groups in 21 regions were compared with global levels and different disease characteristics of each region. Statistical results were presented in Fig. 4 and Additional files 2, 3, 4, 5, 6, and 7. The ASDRs for malignant skin melanoma and mesothelioma in Australasia were 5.7 and 4.7 times higher than that of the global average and the ASIR of malignant skin melanoma was more than 12 times the world average. In 2017, the global ASIR of malignant skin melanoma was 3.87 (4.58–2.97), whereas that of Australasia was 48.33 (58.10–33.54). Moreover, the ASDR of malignant skin melanoma in Central Europe, Eastern Europe, Western Europe, and high-income North America was also two times higher than that of the global levels. The ASDRs of esophageal, stomach, liver, TBL, and nasopharyngeal cancers in East Asia were more than 1.5 times higher when compared to the global levels. The ASDRs of stomach, liver, cervical, nasopharynx, other pharynx, gallbladder, and biliary tract cancers in high-income North America were all below 50% that of the global levels.

**Fig. 5:**
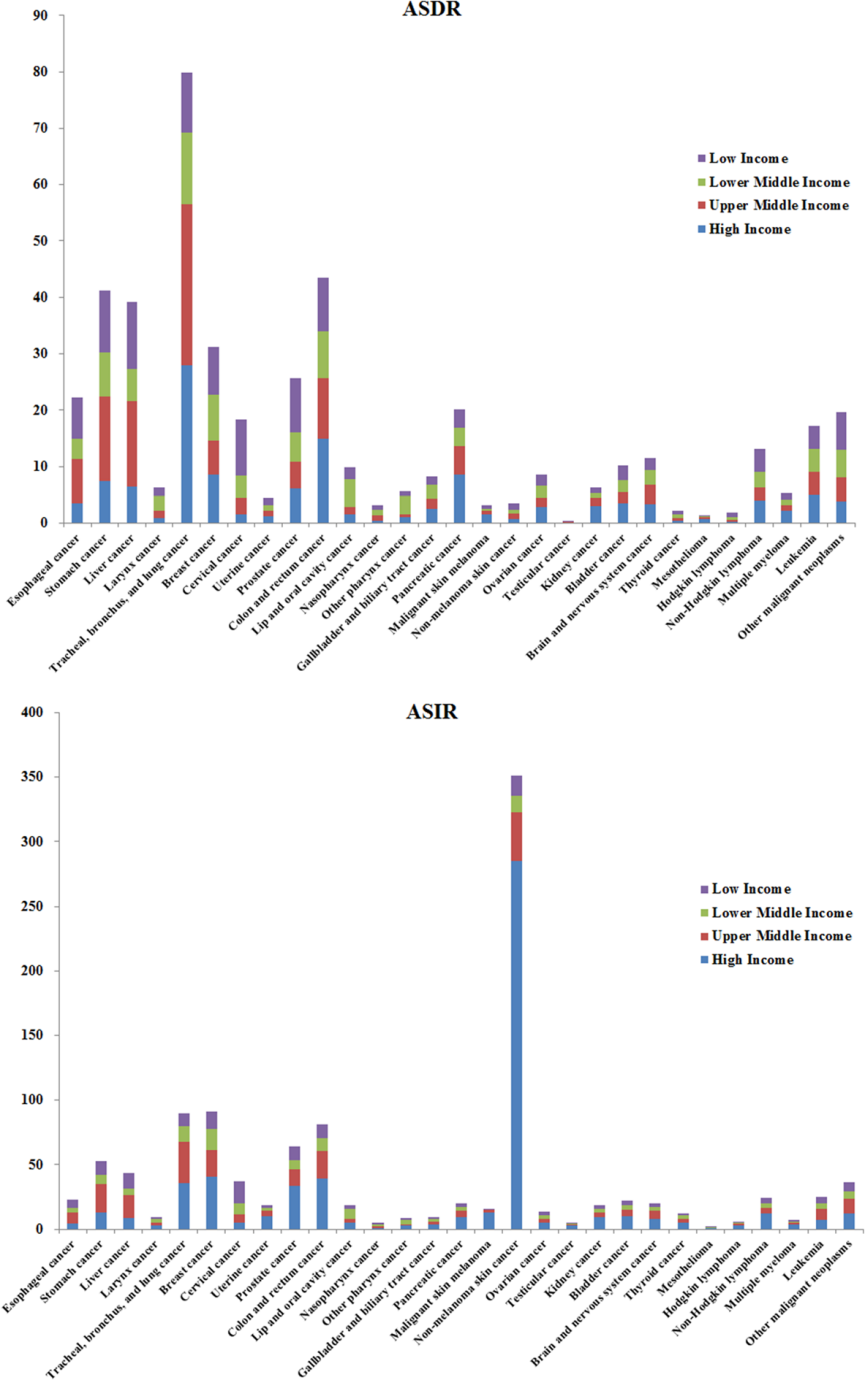
The age-standardized deaths and incidence (per 100,000) of 29 specified cancer groups in 2017 by different HDI regions

In terms of different socioeconomic levels, the incidence and deaths associated with cancer, as well as the distribution of different cancer groups were related to HDI. The results are presented in Fig. 5 and Additional file 8. In 2017, the total number of cancer deaths and the incidence in high- and upper-middle HDI regions were 2.7 times and 5.2 times higher than those of the lower-middle and low HDI regions, respectively. The ASDRs and ASIRs were also higher than those of the middle and low HDI regions. However, the difference in ASDRs between the two regions was not as large as in the ASIRs. This was partly due to higher ASDRs of several cancer groups in low and middle HDI regions. For instance, the ASDR of cervical cancer in low HDI region was 6.3 times higher than that in the high HDI region. TBL cancer demonstrated a high mortality rate in all the four regions, and the ASDRs of TBL cancer in the high, upper-middle, and lower-middle HDI regions were 27.92 (28.41–27.42), 28.62 (29.54–27.65), and 12.71 (13.56–12.03), respectively. In the low HDI region, liver cancer had the highest ASDR, with 11.98 (17.75–10.16). This was followed by the ASDRs of colon and rectal cancer, liver cancer, and breast cancer in the high, upper-middle, and lower-middle HDI regions. The ASIR of non-melanoma skin cancer in the high HDI region was 285.22 (390.40–197.62), which was far higher than that in other regions.

With a declination in the SDI, the total ASDRs and ASIRs of the 29 cancer groups also showed decreasing tendency. The ASDR and ASIR of cancers in the high SDI region were 123.50 (129.39–117.90) and 596.90 (730.58–490.57), while those in the low SDI region were only 98.00 (107.52–87.37) and 124.85 (141.73–108.92), respectively. Similar trend was observed in the ASDRs and ASIRs of some cancer groups, including TBL, colon and rectal, pancreatic, kidney cancers, and leukemia, in these five regions. High ASDRs for TBL cancer, as this was associated with high mortality rate, included 28.29 (28.81–27.77) and 29.99 (31.15–28.74) in the high SDI and high-middle SDI regions, while those in the low-middle and low SDI regions were only 11.0 (11.73–10.38) and 10.82 (11.73–10.38), respectively. However, the ASDRs and ASIRs of certain cancer groups showed opposite trends. Some ASDRs and ASIRs showed rising trends based on the reduction in SDI. Cervical cancer exhibited a most obvious tendency. The ASDRs of cervical cancer were 1.51 (1.56–1.47), 2.35 (2.46–2.08), 3.54 (3.72–2.94), 4.45 (5.10–4.07), and 6.27 (6.97–5.68) in the five SDI regions, ranking from high SDI to low SDI. The detailed results are presented in Table 2.

**Table 2:** The age standardized of death and incidence of 29 specified cancer groups in 2017 by different SDI regions

Tumor types	Age-standardized deaths per 100,000 (95% UI)					Age-standardized incidence per 100,000 (95% UI)				
	High-middle SDI	High SDI	Low-middle SDI	Low SDI	Middle SDI	High-middle SDI	High SDI	Low-middle SDI	Low SDI	Middle SDI
Esophageal cancer	7.22 (7.63–6.84)	3.5 (3.58–3.42)	4.63 (4.88–4.41)	4.54 (4.84–4.28)	6.79 (7.13–6.45)	8.1 (8.59–7.62)	4.3 (4.42–4.18)	4.42 (4.67–4.21)	4.29 (4.57–4.04)	6.89 (7.26–6.55)
Stomach cancer	14.62 (15.24–14.02)	7.41 (7.63–7.21)	9.21 (9.64–8.84)	9.31 (9.83–8.74)	12.37 (12.83–11.93)	21.31 (22.49–20.25)	13.34 (13.81–12.92)	9.36 (9.85–8.94)	8.91 (9.39–8.39)	16.44 (17.19–15.7)
Liver cancer	12.88 (13.71–12.12)	6.66 (6.93–6.44)	7.17 (8.14–6.69)	5.91 (7.05–5.31)	13.72 (14.44–13.01)	15.73 (16.95–14.61)	8.94 (9.43–8.57)	6.96 (7.89–6.48)	5.72 (6.88–5.13)	15.06 (16.01–14.12)
Larynx cancer	1.46 (1.5–1.42)	0.92 (0.94–0.89)	2.36 (2.51–2.22)	2.68 (2.89–2.44)	1.48 (1.58–1.42)	2.68 (2.76–2.59)	2.82 (2.91–2.74)	2.64 (2.82–2.48)	2.84 (3.07–2.59)	2.17 (2.28–2.08)
Tracheal, bronchus, and lung cancer	29.99 (31.15–28.74)	28.29 (28.81–27.77)	11.01 (11.73–10.38)	10.82 (11.51–10.12)	23.46 (24.65–22.38)	34.46 (35.93–32.94)	36.25 (37.04–35.5)	10.41 (11.13–9.81)	10.08 (10.72–9.46)	24.76 (26.16–23.5)
Breast cancer	6.89 (7.16–6.38)	8.42 (8.64–8.18)	8.59 (10.99–7.62)	7.31 (7.95–6.72)	6.32 (6.67–5.7)	23.93 (25.15–22)	40.99 (42.21–39.76)	16.5 (20.62–14.75)	11.62 (12.7–10.7)	17.86 (18.92–16.12)
Cervical cancer	2.35 (2.46–2.08)	1.51 (1.56–1.47)	4.45 (5.1–4.07)	6.27 (6.97–5.68)	3.54 (3.72–2.94)	5.73 (6.04–5.02)	4.68 (4.84–4.52)	9.04 (10.09–8.17)	11.83 (13.06–10.7)	7.89 (8.29–6.49)
Uterine cancer	1.06 (1.1–1.03)	1.22 (1.25–1.18)	1.1 (1.2–1.01)	0.91 (1.02–0.8)	0.89 (0.93–0.85)	4.94 (5.17–4.73)	9.93 (10.28–9.6)	2.35 (2.54–2.18)	1.49 (1.68–1.3)	3.19 (3.37–3.03)
Prostate cancer	4.52 (5.2–3.79)	5.96 (7.96–4.96)	6.81 (7.73–5.32)	6.26 (7.02–4.71)	4.79 (5.7–4.11)	13.4 (15.26–11.2)	33.38 (47.04–28.96)	9.22 (10.37–7.39)	6.73 (7.54–5.11)	10.54 (12.57–9.11)
Colon and rectum cancer	12.58 (12.92–12.19)	14.84 (15.19–14.46)	7.85 (8.26–7.41)	8.12 (8.57–7.66)	9.05 (9.39–8.65)	25.05 (25.93–24.13)	39.2 (40.17–38.15)	9.62 (10.11–9.09)	8.76 (9.25–8.26)	15.98 (16.62–15.23)
Lip and oral cavity cancer	1.51 (1.56–1.46)	1.5 (1.54–1.46)	4.7 (5.13–4.31)	4.34 (4.64–4.01)	2.26 (2.36–2.14)	3.14 (3.26–3.03)	5 (5.14–4.85)	7.45 (8.18–6.81)	5.94 (6.36–5.52)	4.2 (4.38–3.97)
Nasopharynx cancer	0.92 (1–0.85)	0.42 (0.44–0.41)	0.86 (0.91–0.81)	0.89 (0.96–0.83)	1.14 (1.22–1.07)	1.6 (1.8–1.42)	0.75 (0.79–0.7)	1.18 (1.27–1.1)	1.14 (1.24–1.04)	1.76 (1.9–1.63)
Other pharynx cancer	0.72 (0.76–0.68)	0.95 (0.99–0.91)	2.96 (3.25–2.41)	3.42 (3.81–2.55)	1.1 (1.24–0.98)	1.27 (1.34–1.2)	2.58 (2.69–2.48)	3.44 (3.76–2.83)	3.78 (4.21–2.85)	1.48 (1.69–1.33)
Gallbladder and biliary tract cancer	1.88 (1.99–1.58)	2.47 (2.58–2.3)	2.33 (2.9–2.07)	2.38 (2.74–1.91)	1.9 (2.08–1.59)	2.06 (2.22–1.68)	3.97 (4.36–3.6)	2.18 (2.72–1.94)	2.21 (2.54–1.76)	1.89 (2.06–1.57)
Pancreatic cancer	6.03 (6.21–5.86)	8.54 (8.72–8.36)	3.46 (3.71–3.29)	2.63 (2.83–2.41)	3.83 (3.98–3.66)	5.87 (6.04–5.7)	9.26 (9.47–9.03)	3.22 (3.46–3.06)	2.44 (2.62–2.25)	3.61 (3.76–3.45)
Malignant skin melanoma	0.78 (0.93–0.65)	1.59 (1.91–1.12)	0.35 (0.42–0.28)	0.36 (0.46–0.3)	0.37 (0.42–0.3)	2.65 (3.16–2.19)	13.27 (16.71–9.91)	0.51 (0.6–0.41)	0.43 (0.55–0.36)	0.79 (0.91–0.65)
Non-melanoma skin cancer	0.89 (0.92–0.86)	0.66 (0.68–0.64)	0.88 (0.94–0.83)	0.87 (0.99–0.7)	0.99 (1.02–0.96)	34.25 (48.65–22.94)	287.52 (393.27–199.28)	18.59 (28.88–10.81)	10.4 (15.78–6.54)	32.04 (45.68–20.76)
Ovarian cancer	2.01 (2.07–1.95)	2.81 (2.9–2.71)	2.18 (2.48–1.98)	1.63 (1.9–1.46)	1.72 (1.79–1.65)	3.3 (3.42–3.19)	4.82 (4.99–4.66)	3.44 (4–3.11)	2.31 (2.69–2.05)	2.92 (3.05–2.8)
Testicular cancer	0.11 (0.11–0.11)	0.1 (0.11–0.1)	0.1 (0.12–0.09)	0.07 (0.08–0.07)	0.1 (0.1–0.1)	1.1 (1.18–1.03)	3.07 (3.24–2.91)	0.27 (0.31–0.23)	0.12 (0.13–0.1)	0.63 (0.68–0.59)
Kidney cancer	1.93 (2.01–1.81)	2.97 (3.07–2.73)	0.84 (0.9–0.78)	0.65 (0.73–0.55)	1.04 (1.11–0.98)	5.12 (5.35–4.87)	8.94 (9.25–8.55)	2.64 (2.82–2.44)	2.12 (2.39–1.8)	3.08 (3.27–2.89)
Bladder cancer	2.52 (2.66–2.44)	3.44 (3.53–3.34)	2.2 (2.45–2.06)	1.86 (2.19–1.7)	1.69 (1.88–1.61)	5.97 (6.32–5.77)	10.26 (10.59–9.94)	3.79 (4.16–3.54)	2.59 (3.06–2.37)	3.41 (3.78–3.25)
Brain and nervous system cancer	3.67 (3.97–3.21)	3.36 (3.54–2.77)	2.52 (2.95–2.15)	2.32 (2.58–1.8)	2.99 (3.23–2.47)	7.19 (8.32–6.19)	8.45 (9.09–7.1)	2.91 (3.4–2.48)	2.58 (2.91–2.05)	4.54 (5.02–3.72)
Thyroid cancer	0.44 (0.46–0.42)	0.44 (0.45–0.42)	0.61 (0.69–0.57)	0.62 (0.68–0.55)	0.55 (0.61–0.52)	3.3 (3.5–3.12)	5.17 (5.42–4.98)	2.14 (2.47–1.95)	1.63 (1.85–1.46)	2.61 (2.97–2.45)
Mesothelioma	0.25 (0.26–0.24)	0.75 (0.78–0.72)	0.22 (0.25–0.19)	0.18 (0.26–0.14)	0.19 (0.2–0.19)	0.3 (0.31–0.28)	0.85 (0.89–0.82)	0.26 (0.31–0.23)	0.22 (0.32–0.17)	0.23 (0.24–0.22)
Hodgkin lymphoma	0.3 (0.33–0.24)	0.27 (0.34–0.24)	0.7 (0.87–0.58)	0.59 (0.8–0.5)	0.29 (0.34–0.24)	1.79 (1.97–1.39)	2.82 (3.82–2.56)	0.95 (1.15–0.78)	0.7 (0.95–0.6)	0.76 (0.89–0.62)
Non-Hodgkin lymphoma	2.56 (2.64–2.49)	3.84 (3.92–3.75)	2.96 (3.17–2.74)	2.99 (3.21–2.66)	2.51 (2.61–2.42)	5.38 (5.56–5.19)	12.07 (12.39–11.75)	3.41 (3.67–3.16)	3.22 (3.46–2.86)	3.68 (3.83–3.54)
Multiple myeloma	1.08 (1.16–0.98)	2.13 (2.55–1.9)	1.04 (1.19–0.95)	1.03 (1.16–0.95)	0.88 (0.92–0.79)	1.52 (1.63–1.36)	3.72 (4.57–3.4)	1.08 (1.23–0.99)	1.02 (1.15–0.94)	1.06 (1.12–0.94)
Leukemia	4.3 (4.5–3.81)	4.81 (4.93–4.69)	3.95 (4.54–3.58)	3.74 (4.16–3.14)	4.15 (4.36–3.62)	8.68 (9.46–7.43)	7.73 (8.02–7.43)	4.54 (5.21–4.11)	4.14 (4.62–3.51)	6.47 (6.98–5.68)
Other malignant neoplasms	4.93 (5.19–4.23)	3.71 (3.92–3.33)	5.14 (5.51–4.85)	5.26 (5.69–4.69)	4.05 (4.25–3.82)	12.95 (13.73–11.35)	12.81 (13.77–11.71)	5.73 (6.12–5.36)	5.59 (6.03–5.02)	7.66 (8.08–7.08)

### Global cancer burden in different countries

**Fig. 6:**
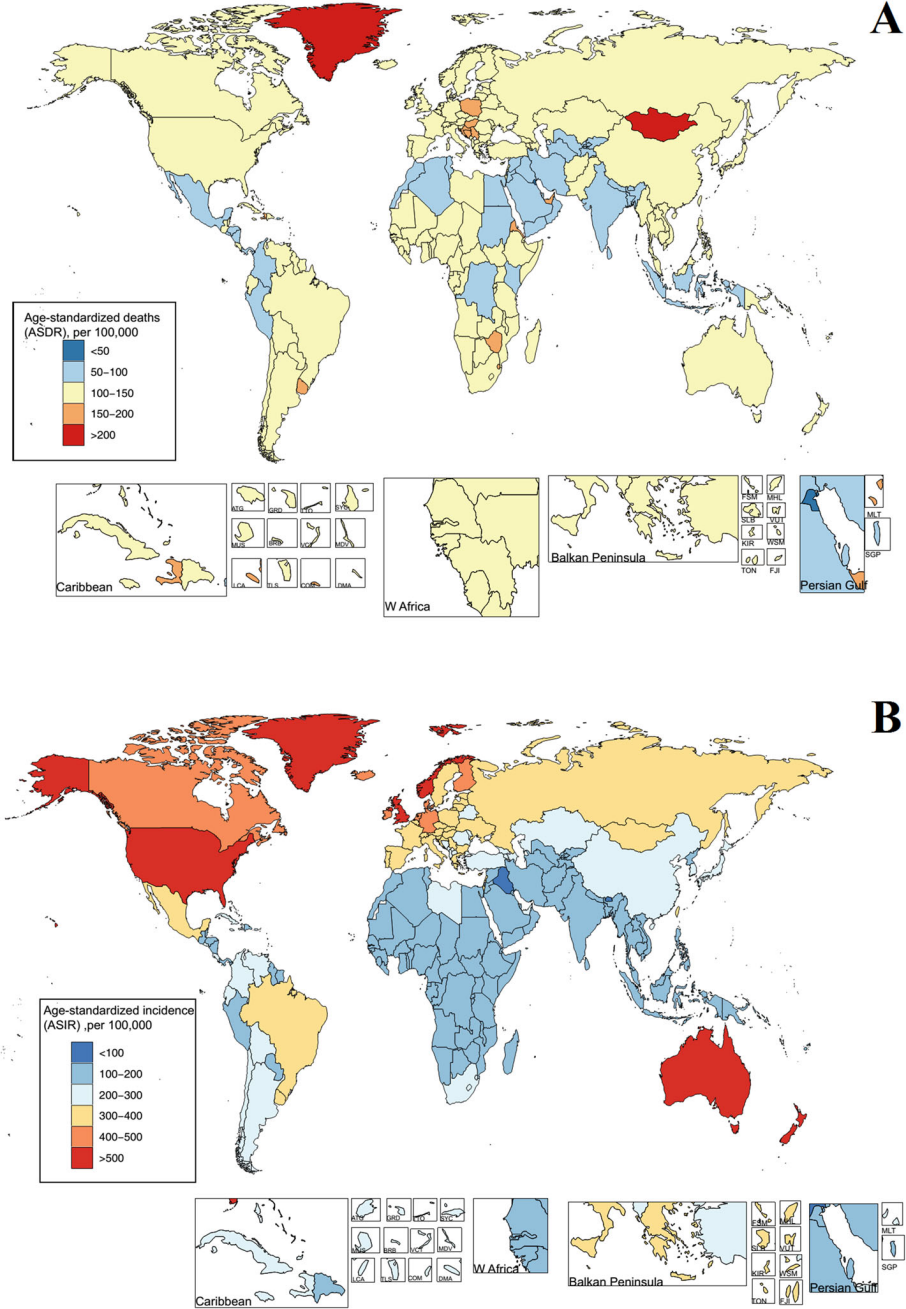
The global disease burden of cancers in 195 countries and territories. **a** ASDR of cancers in 2017. **b** ASIR of cancers in 2017

**Fig. 7:**
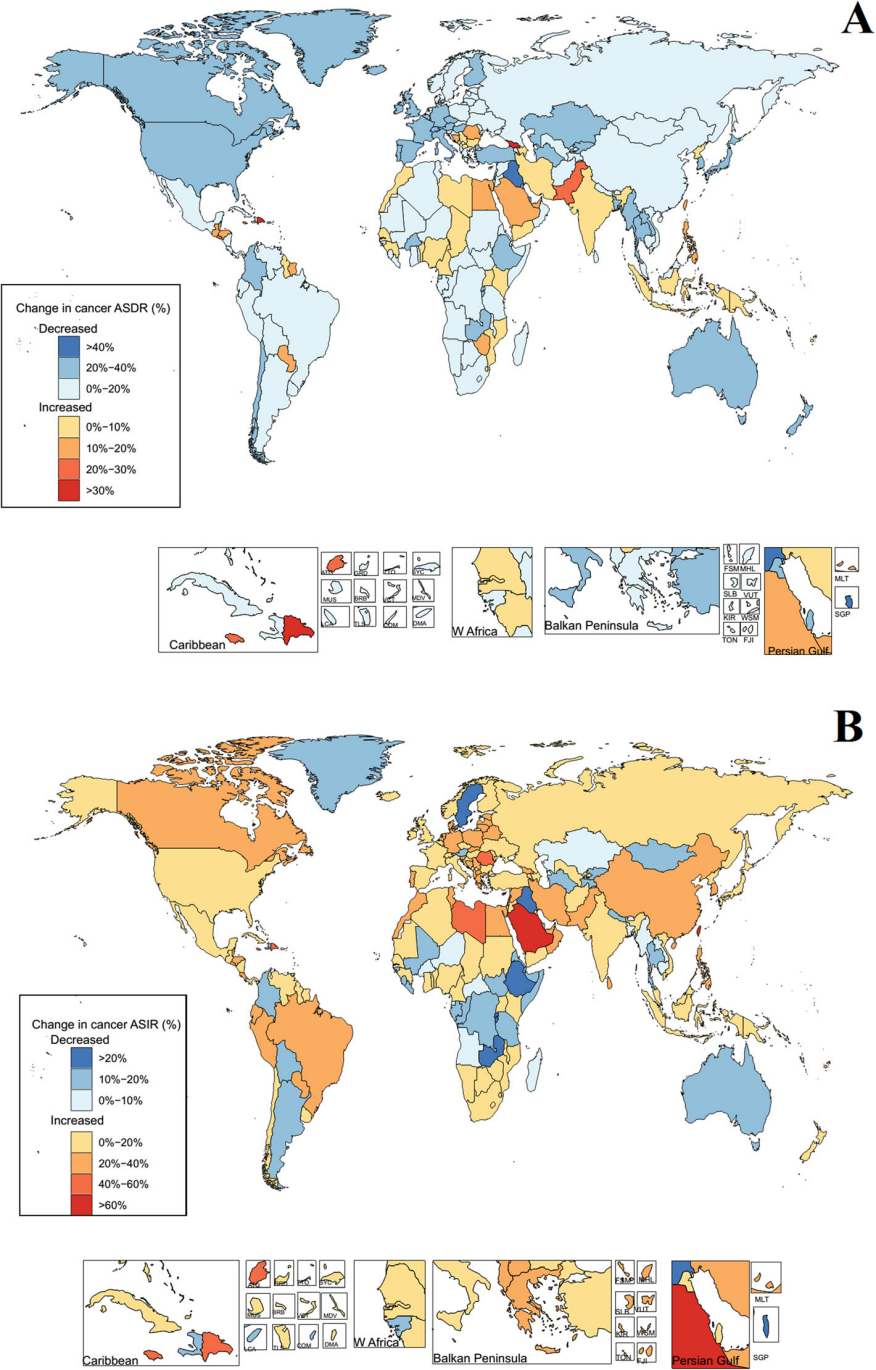
The relative changes in ASDR (**a**) and ASIR (**b**) of 29 specified cancer groups between 1990 and 2017

The total ASDRs and ASIRs of all cancer groups in 195 countries in 2017 were counted and displayed in Fig. 6 (details in Additional file 9 to Additional file 10). Among them, the ASDRs in 155 countries were greater than 100. Greenland and Mongolia had the highest ASDRs, with 216.48 (243.19–191.37) and 238.41 (270.33–205.93), respectively. The ASDRs of Kuwait, Maldives, and Iraq remained the lowest, and were 49.53 (57.50–42.98), 56.31 (64.74–48.21), and 56.25 (63.26–49.69), respectively. Among the 195 countries, the total ASIRs in 193 countries were greater than 100 and those in 95 countries were more than 200. Australia and New Zealand showed the highest ASIRs, with 830.08 (1093.13–612.30) and 856.85 (1169.99–588.05), respectively. This was due to the regional characteristics of cancer, i.e., malignancy of skin melanoma [12, 13]. In addition, the ASDRs and ASIRs in 1990 and 2017 were analyzed using the increased rate as index. The increase in the ASDRs and ASIRs in 195 countries was illustrated in Fig. 7 and Additional file 9 to Additional file 10. The results suggested that the ASDRs of 138 countries were decreased, while only 57 countries showed an increasing trend, with increasing rates of less than 40%. The largest increase in ASDRs was found in Georgia and the Dominican Republic, whose values were 34.22% and 31.11%, respectively. The increasing rates of ASIRs in these countries were also high, which were 33.44% and 53.40%, respectively. The increasing rates of liver cancer, prostate cancer, other pharynx cancer, brain and nervous system cancer, pancreatic cancer, ovarian cancer, and thyroid cancer in Georgia and the Dominican Republic were greater than 40%. Countries with a declination of more than 40% included Singapore, Bahrain, and Iraq, and the decreased rate was − 42.69%, − 48.88%, and − 41.79%, respectively.

**Fig. 8:**
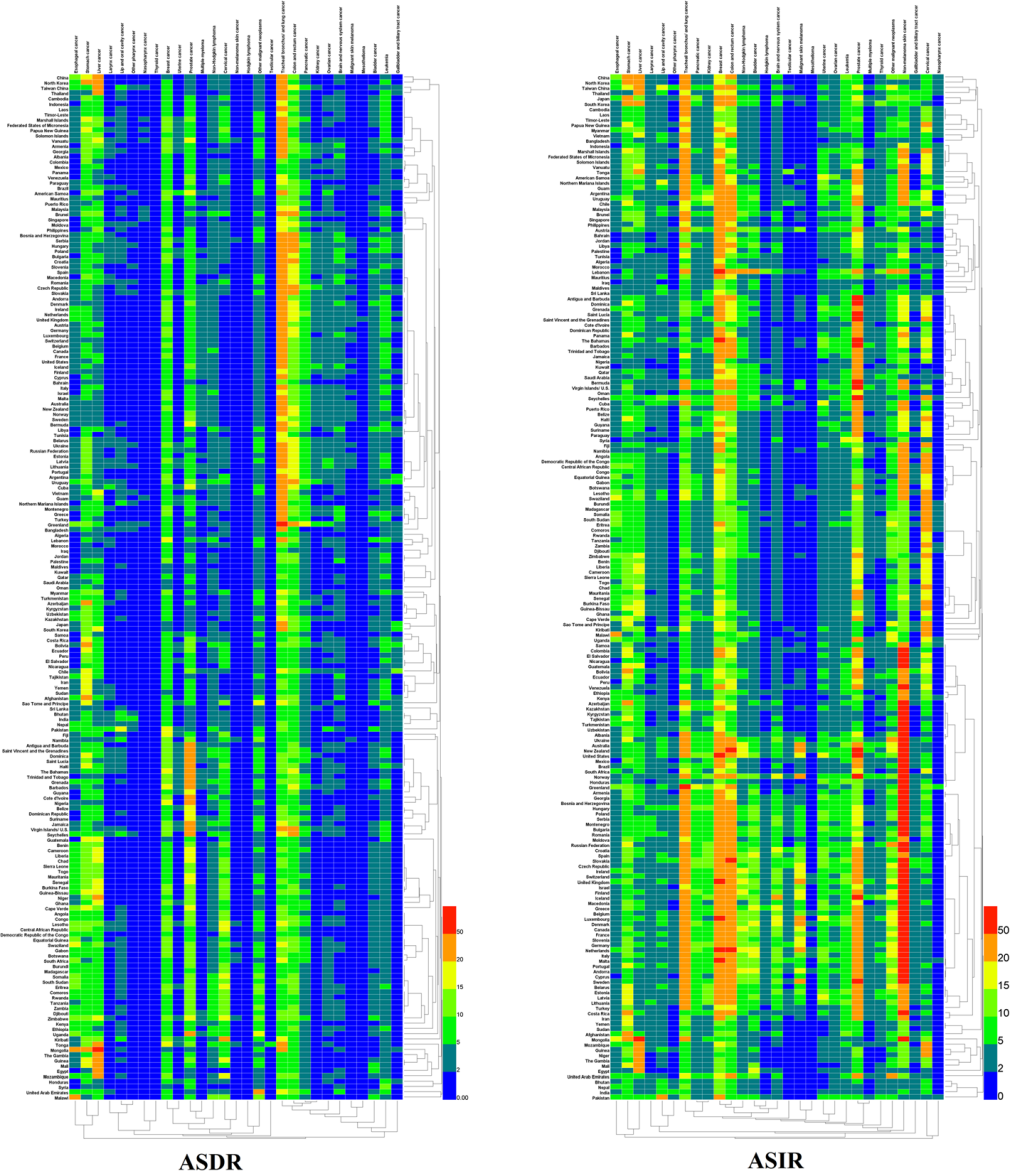
Twenty-nine specified cancers ranked by age-standardized deaths and incidence in 195 countries and territories in 2017. *ASDR* age-standardized deaths per 100,000, *ASIR* age-standardized incidence per 100,000

In addition, the ASDRs and ASIRs of 29 cancer groups in 2017 were analyzed and the results are presented in Fig. 8 (details in Additional file 9 to Additional file 10). The mortality rates of TBL, stomach, liver, breast, colon and rectal, and prostate cancers were higher when compared to other cancers in all the countries. TBL cancer was considered to be the most common one, with ASDRs of greater than 5 in all countries. Among all the countries, TBL cancer in Greenland had the highest ASDR [76.18 (81.95–69.90)], accounting for more than one-third of the ASDR from all cancers in the country. Among all cancer groups in all the countries, liver cancer in Mongolia had the highest ASDR, which was as high as 100.14 (111.81–87.01) and accounted for about one-half of the total ASDR of the country.

**Fig. 9:**
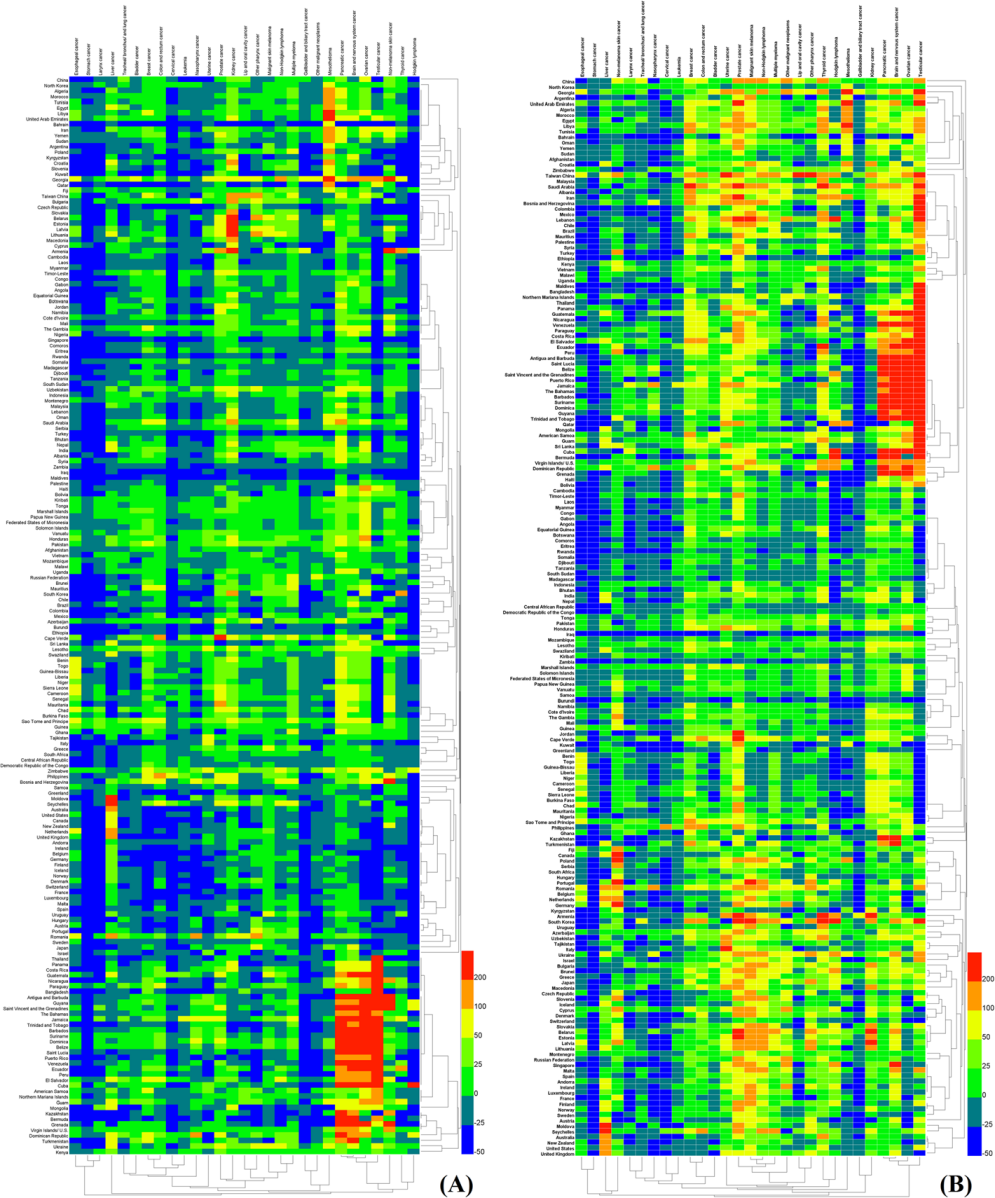
The relative changes in ASDR (**a**) and ASIR (**b**) of 29 specified cancer groups between 1990 and 2017 in 195 countries and territories. **a** Comparison of age-standardized deaths. **b** Comparison of age-standardized incidence

The increase in ASDRs and ASIRs in 29 cancer groups in 2017 was analyzed by comparing with those in 1990. The rates were calculated by the following formula: [(2017ASDR or ASIR) − (1990ASDR or ASIR)]/(1990ASDR or ASIR) × 100 and the results are presented in Fig. 9 (detials in Additional file 9 to Additional file 10). The ASDRs of stomach, esophageal, laryngeal cancers, and Hodgkin lymphoma in various countries showed decreasing trends and the increase in ASDR of most of the cancers in most of the countries was less than 25%. However, the rate of pancreatic, ovarian, and testicular cancers increases, which was more than 100%, in more than 20 countries, such as Antigua and Barbuda, the Bahamas, Barbados, Belize, Cuba, and Dominica, and even more than 300% in some countries (shown in the red region at the lower right corner of Fig. 9). The results of ASIRs revealed that most of the cancer groups exhibited increasing trends in all the countries. There was an increase in the ASIR in pancreatic, ovarian, testicular, and brain and nervous system cancers in 20 countries (as mentioned above, such as Antigua and Barbuda, the Bahamas, etc.) was also greater than 100%.

### Predictive analysis of cancers

#### Breast cancer

**Fig. 10:**
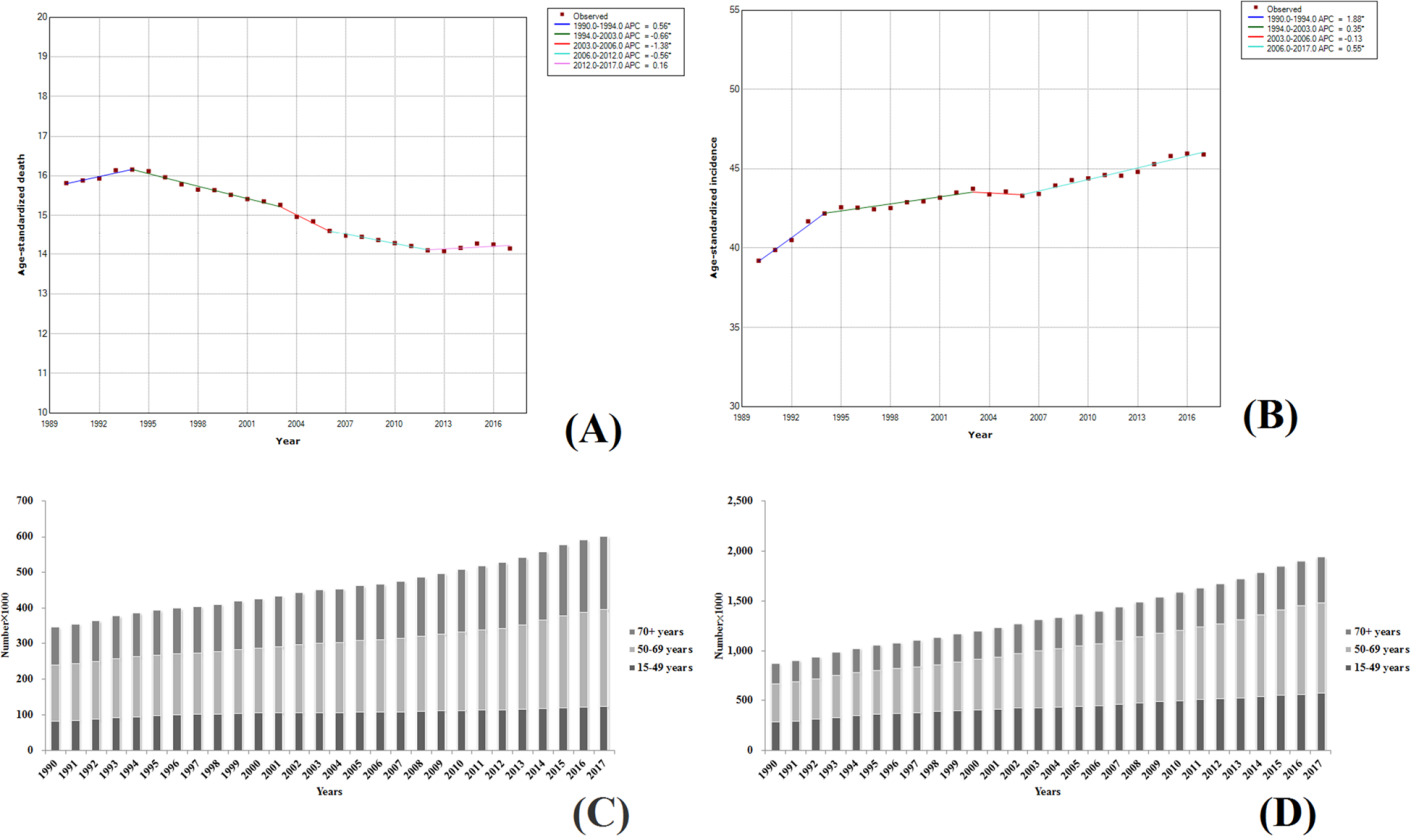
Trends in global breast cancer, ASDR (**a**), ASIR (**b**), death number (**c**), and incidence number (**d**) from 1990 to 2017

Breast cancer is the leading malignancy that is seriously threatening the health of the women [14–16]. In 2017, the ASIR of breast cancer in high SDI countries remained the highest, with up to 40.99 (42.21–39.76), which is about four times higher than those in the low SDI countries. From 1990 to 2017, of the 195 countries, the ASIR of breast cancer in 175 countries showed an increasing trend and the ASIRs in 65 countries were increased by more than 50%. The largest increase was observed in Saudi Arabia, and the ASIR was increased by 226% (the ASIR in 2017 was three times higher than that in 1990), followed by China and Taiwan, with an increase of 189%. The number of deaths of women with breast cancer worldwide was increased from 344,906 (376,023–327,824) to 600,728 (629,932–578,725) and the incidence was increased from 870,183 (927,102–837,709) to 1,937,574 (2,000,363–1,868,019), with an approximate of twofold increase (Fig. 10a, b). The global analysis of morbidity and mortality trends of female breast cancer from 1990 to 2017 showed an upward trend in the ASDR during 1990–1994, with an annual percentage change (APC) of 0.56, followed by a downward trend from 1994 to 2012 and then a stabilization from 2012–2017 with slow growth (APC = 0.16). However, as for the ASIR, the rate was higher from 1990 to 1994 (APC = 1.88), followed by a continuous rise during 2006–2017, with an APC of 0.55. Until 2017, the global ASIR of female breast cancer was 45.91 (47.40–44.24), which was increased by about 20% since 1990. However, the ASDR in 2017 was 14.15 (14.84–13.63), which was slightly lower than that in 1990 [15.82 (17.21–15.06)]. However, there were no patients with breast cancer at the age of 15 during the study conduction. Women aged 50–69 years old demonstrated a higher burden of breast cancer and their incidence number and deaths accounted for about 40% of the total. The results are shown in Fig. 10c, d.

#### Prostate cancer

**Fig. 11:**
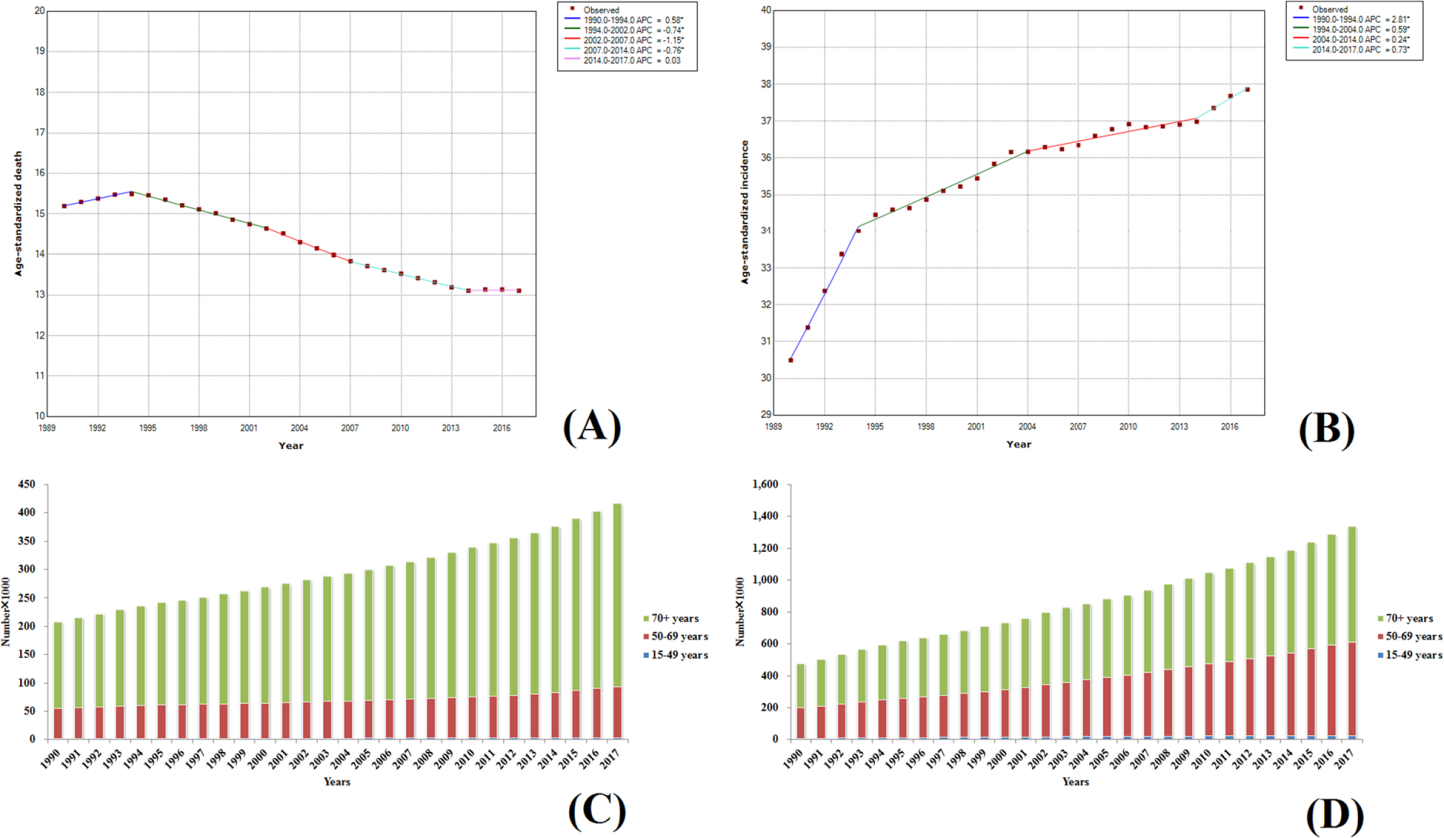
Trends in global prostate cancer, ASDR (**a**), ASIR (**b**), death number (**c**), and incidence number (**d**) from 1990 to 2017

In 2017, the ASIRs of prostate cancer remained the highest in high SDI countries, which was about five times higher than those in the low SDI countries. However, the ASDRs in high SDI countries were slightly lower than those in low SDI countries. For the past 28 years, there were more countries with increased ASIRs of prostate cancer than that of breast cancer. Of the 195 countries, only six countries all over the world showed a downward trend with a maximum declination rate of only 18%. Fifty-four of the 189 countries with increasing trends in the ASIRs showed increased rates of more than 100% and the country with the highest increase rate was Cape Verde (334%). The number of males killed by prostate cancer worldwide was increased from 208,402 (235,155–164734) to 415,910 (489,540–357,283) and the incidence was increased from 478,138 (532,516–359,364) to 13,343,214 (1,697,900–1,170,862) (Fig. 11a, b). The global morbidity and mortality trends of prostate cancer in males from 1990 to 2017 were analyzed. The results were similar to that of female breast cancer. From 1990 to 1994, the ASDRs exhibited an upward trend (APC = 0.58), while showed a declination from 1994 to 2014, followed by a smoother trend during 2014–2017. However, in terms of ASIRs, an upward trend and increased rates were observed to be the largest between 1990 and 1994, with an APC reaching to 2.81. Until 2017, the global male prostate cancer ASIR was 37.86 (47.99–33.03), which was increased by about 20% when compared to those in 1990. Regarding the age at onset, prostate cancer was not observed in patients aged below 15 years, and similar to breast cancer, the main burden of prostate cancer was concentrated in people aged 50 years and older. Their morbidity and mortality accounted for ≥ 98% of the total morbidity and mortality (Fig. 11c, d).

#### Liver cancer

**Fig. 12:**
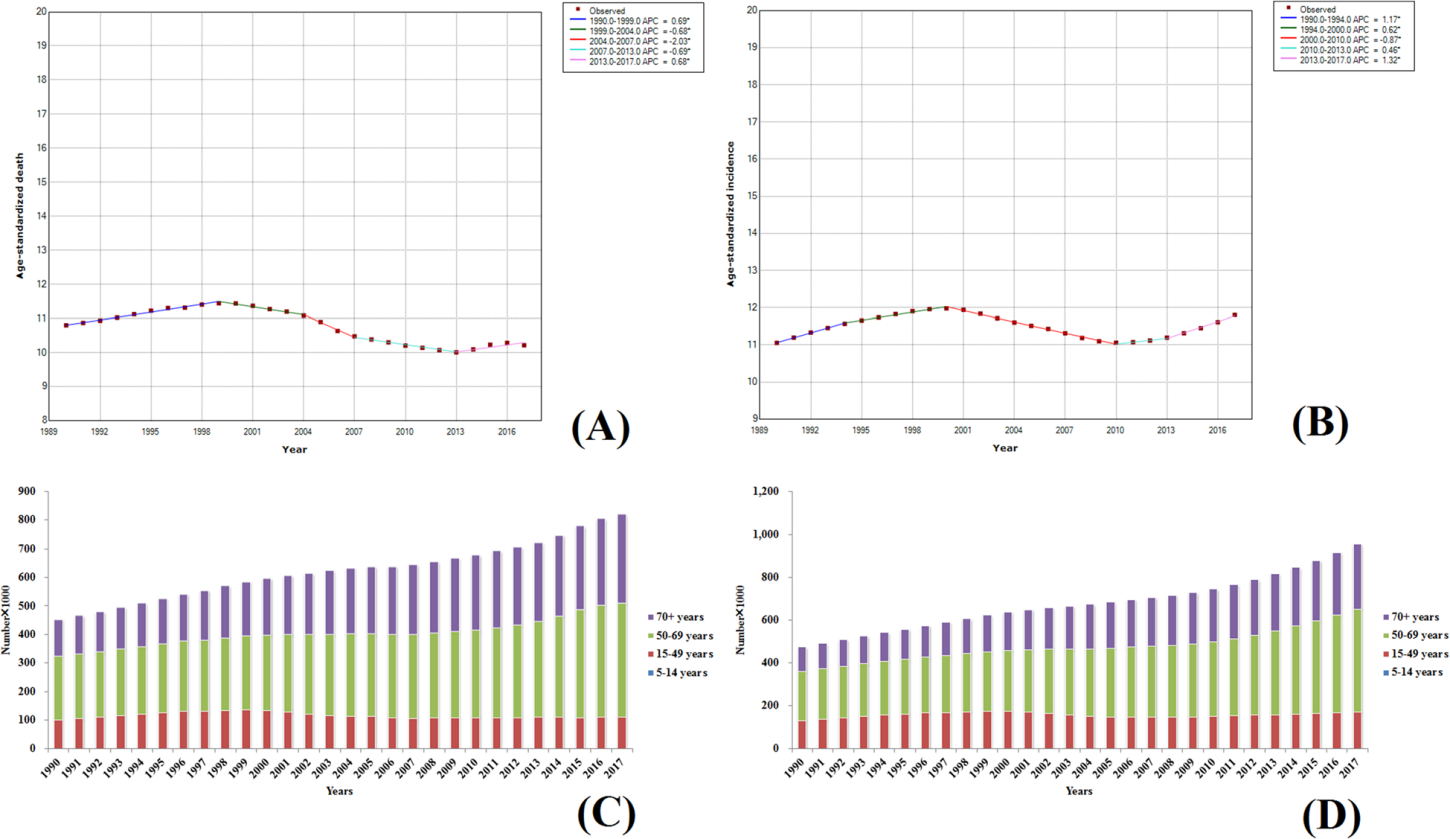
Trends in global liver cancer, ASDR (**a**), ASIR (**b**), death number (**c**), and incidence number (**d**) from 1990 to 2017

Liver cancer is a common and deadly malignancy that caused more than 800,000 deaths worldwide in 2017. Sufficient evidence showed that the incidence of liver cancer varied widely around the world [11], with the highest incidence being in East Asia. This was reported to be more than five times higher than that of eastern Europe or South Asia. In high-middle and middle SDI regions, the ASIR and ASDR of liver cancer were higher than those in the other three SDI regions. The global ASIRs and ASDRs for liver cancer in 2017 were basically the same as those in 1990, but the ASIRs and ASDRs of liver cancer in 195 countries in 2017 were different from those in 1990. The ASIRs of 100 countries showed a downward trend and the ASIRs of Burkina Faso and Sierra Leone were declined by nearly 70%. Among those countries with increasing trends, the ASIRs were increased by 226% and 198% in Seychelles and Moldova, respectively. The results of trend analysis of morbidity and mortality of liver cancer in the population worldwide from 1990 to 2017 are presented in Fig. 12a, b. During 1990–1999, the ASDR demonstrated an upward trend with an APC of 0.69, while from 1999 to 2007, a downward trend was observed, and with a rapid decline from 2004 to 2007, wherein the APC was − 2.03. The ASDRs from 2007 to 2017 were first declined, and then increased. The overall trend of ASIRs showed a Z-shaped change, i.e., it rose at both ends and declined in the middle (2000–2010). However, in general, the change in the global burden of liver cancer showed no significance. The values of ASDR and ASIR were 10.80 (11.68–10.18) and 11.05 (11.97–10.42) in 1990, and 10.21 (10.65–9.85) and 11.80 (12.34–11.35) in 2017, respectively. Regarding the age at onset, data on the morbidity and mortality of liver cancer in individuals below 15 years of age were included in the GBD report (Fig. 12c, d), but the percentage was less than 1% of the total annual morbidity and mortality, showing a downward trend over the years. The age associated with the burden of liver cancer was the individuals with over 50 years age and the increase in morbidity and death in the recent years accounted for more than 80% of the total increase in morbidity and deaths due to liver cancer.

#### TBL cancer

**Fig. 13:**
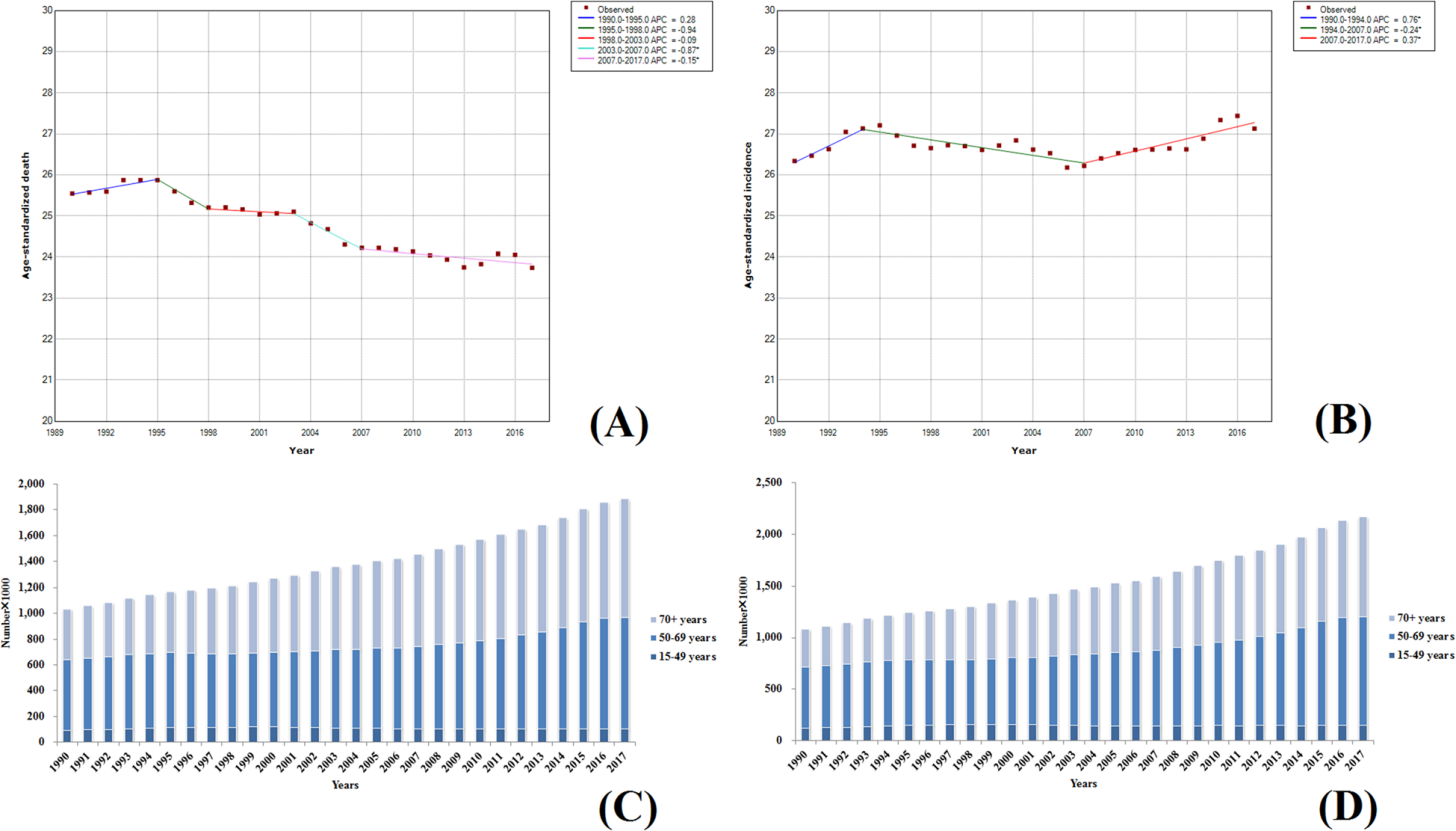
Trends in global TBL cancer, ASDR (**a**), ASIR (**b**), death number (**c**), and incidence number (**d**) from 1990 to 2017

In 2017, the worldwide number of deaths due to TBL cancer reached 1,883,066 (1,922,809–1,844,246), accounting for about one-fifth of the global deaths due to malignant tumors. The mortality rate of TBL remained the highest among all malignant tumors. Since smoking is the leading cause of lung cancer [17], and there are more males who smoke than females [18–20], the global male ASDR due to TBL cancer was 35.38 (36.35–34.39), which was much higher than that of females [13.94 (14.36–13.53)]. TBL cancers in the low-middle and low SDI regions had low ASDRs and ASIRs, which were about one-third of those in the other three regions. Compared with 1990, the ASIR of TBL cancer was slightly decreased and a downward trend was observed by 130 countries around the world. Furthermore, the declination in ASIRs in 13 countries was over 30% and the maximum declination (66%) was found in Bahrain. Among the countries with increasing incidence rates, China showed the largest increase and its ASIR was increased by 53%. Analysis of incidence and mortality trends associated with TBL cancer during the past 28 years revealed that the other four stages (from 1995 to 1998, 1998 to 2003, 2003 to 2007, and 2007 to 2017) showed a downward trend, except for the slow increase from 1990 to 1995 (the APC was 0.28). However, the ASIR showed an upward trend between 1990 and 1994 and between 2007 and 2017 (Fig. 13a, b). Similar to liver cancer, the incidence and mortality rates of male TBL cancer were more than twice that of the females. In 2017, the ASDR and ASIR of TBL cancer in females were 13.94 (14.36–13.53) and 16.26 (16.74–15.77), respectively, while those in males were 35.38 (36.35–34.39) and 39.89 (41.13–38.72), respectively. More than 90% of patients with TBL cancer were over 50 years of age. Along with increasing age, the morbidity and mortality also showed an increasing tendency. The number of patients over 70 years age accounted for about 50% of the total patient number. The statistical results are presented in Fig. 13c, d. TBL cancer did not occur in individuals below 15 years of age. The number of incidences and deaths in patients with liver cancer over 70 years of age in 2017 was 2.3 and 2.6 times higher than that in 1990, which was much higher than 15 to 49 years age group and 50 to 69 years age group.

#### Colon and rectal cancer

**Fig. 14:**
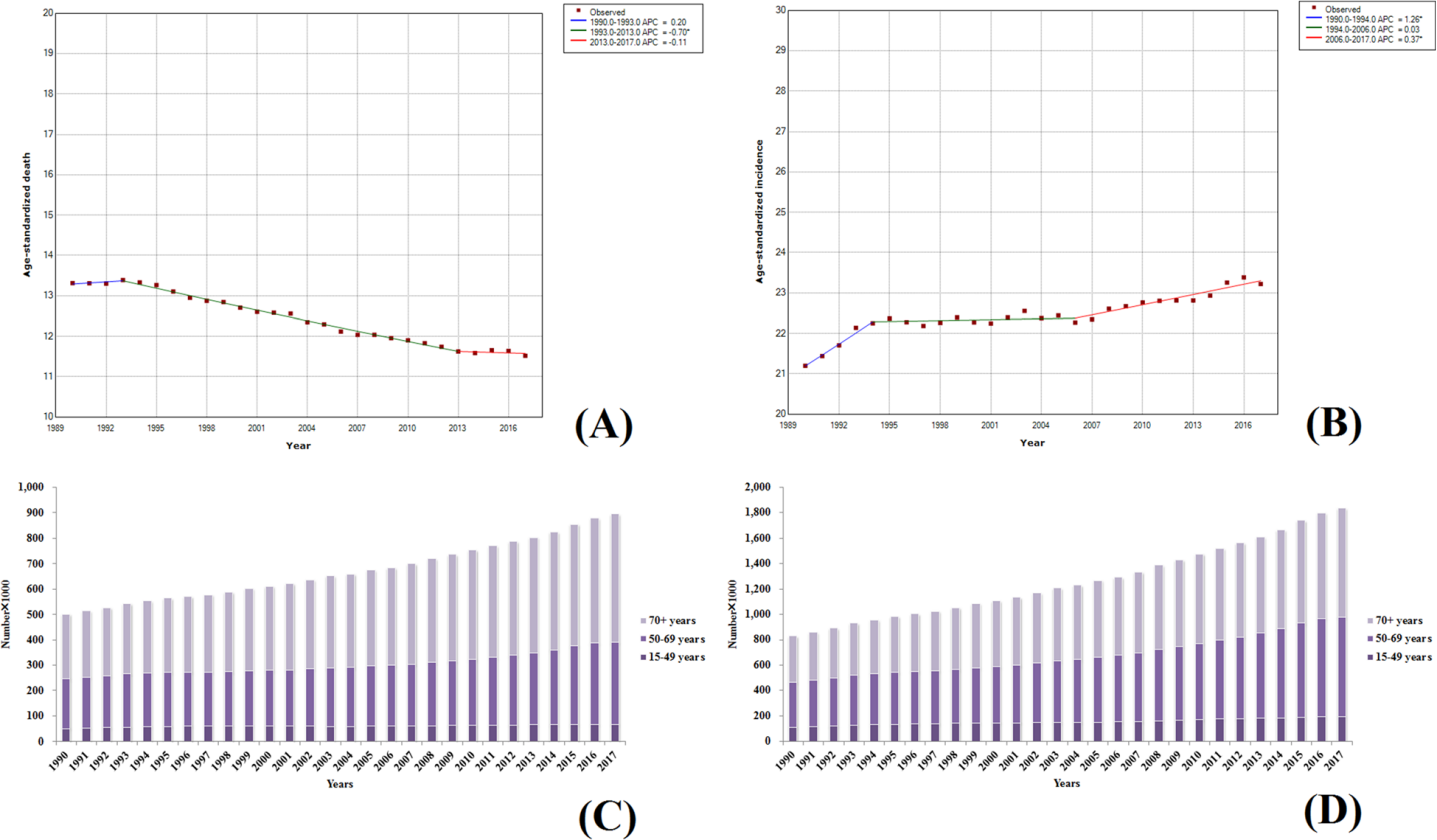
Trends in global colon and rectal cancer, ASDR (**a**), ASIR (**b**), death number (**c**), and incidence number (**d**) from 1990 to 2017

High-fat and high-calorie diets, low fruits and vegetables intake, smoking, drinking, lack of physical exercise, and obesity are the main risk factors of colon and rectal cancer [21–23]. The mortality rate of colon and rectal cancer was shown to be second highest, which was just after TBL cancer. In 2017, its global ASDR was 11.52 (11.77–11.27). The number of deaths was close to 900,000 and the ASIR was 23.22 (23.73–22.7), which was significantly increased when compared to 1990. The ASIRs and ASDRs of colon and rectal cancer in high SDI regions were higher than those in low SDI regions, with obvious regional characteristics. Of the 195 countries worldwide, only 26 countries showed decreased ASIRs and the declination rates were less than one-third. The ASIRs in the other 169 countries showed an increasing trend and the increased rate in 36 countries was more than 50% when compared to 1990. The Philippines had the largest rate with an increase of 182%. The morbidity and mortality trends of colon and rectal cancer from 1990 to 2017 were then analyzed. The ASDR results indicated that, except for the slowly rising trend from 1990 to 1993 (with APC of 0.2), the remaining 24 years demonstrated a downward trend. However, the ASIR showed an upward trend for over 28 years, with the fastest rise from 1990 to 1994 (APC of 1.2), (Fig. 14a, b). Similarly, the morbidity and mortality of colon and rectal cancer in males were higher than those in females. The ASDR and ASIR in males in 2017 were 13.79 (14.22–13.30) and 27.99 (28.87–26.99), respectively, while those of females were 9.65 (9.87–9.35) and 19.17 (19.65–18.60), respectively. Nearly 90% of patients with colon and rectal cancer were aged over 50 years at the age of onset and there were increased proportion of patients at this age. The average age of colon and rectal cancer incidence from 1990 to 2017 was increased from 66.0 to 67.7. The number of patients over 50 years of age who died in 2017 was 92.3% of all death number due to colon and rectal cancer. The statistical results are shown in Fig. 14c, d.

#### Stomach cancer

**Fig. 15:**
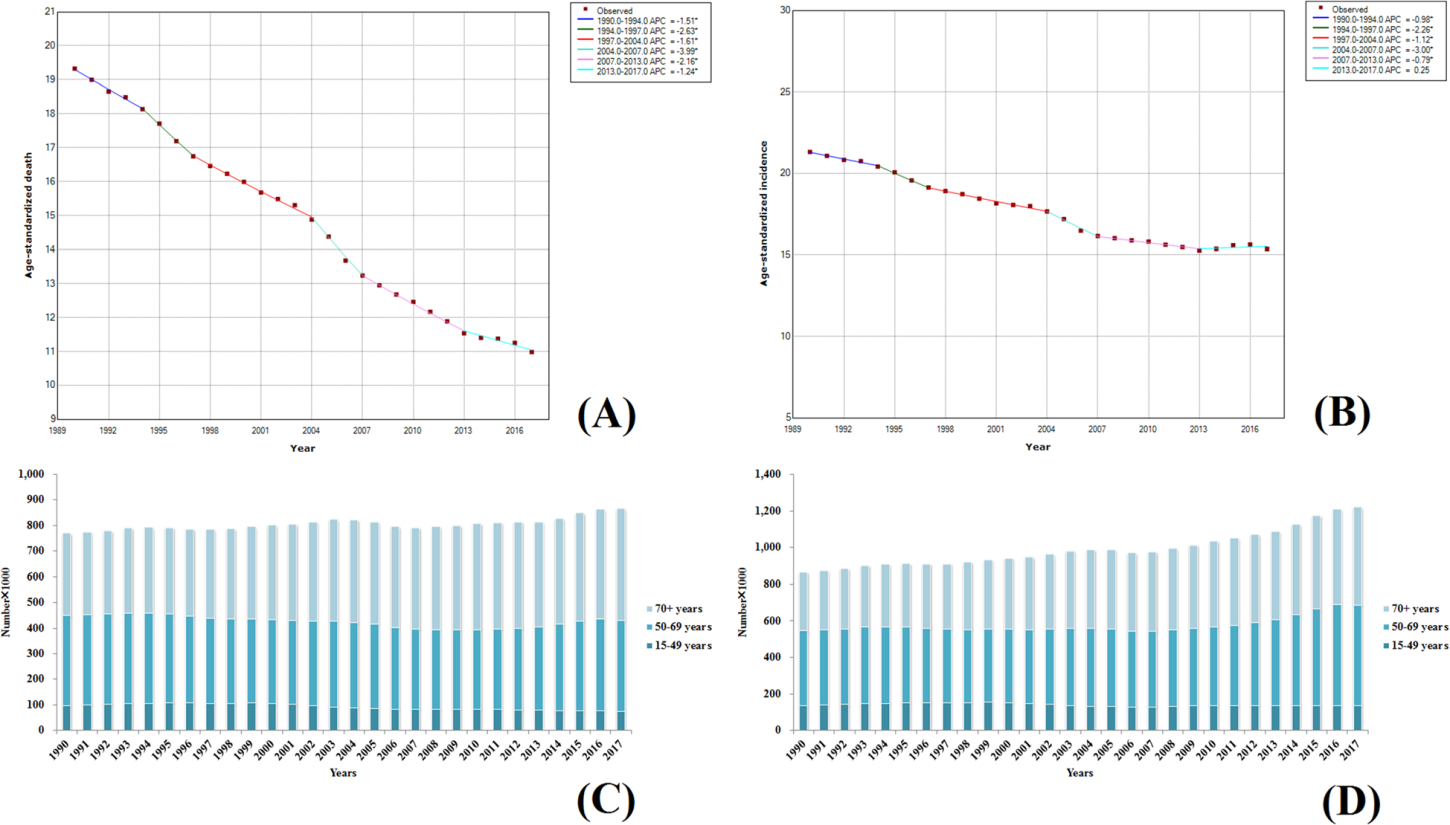
Trends in global gastric cancer, ASDR (**a**), ASIR (**b**), death number (**c**), and incidence number (**d**) from 1990 to 2017

Stomach cancer is also a type of cancer with high morbidity and mortality rates. In 2017, the number of people who died due to stomach cancer worldwide was 864,989 (884,655–848,254) and the incidence was 1,220,662 (1,254,563–1,189,032). The ASDR and ASIR were 10.98 (11.23–10.77) and 15.36 (15.78–14.97), respectively. Compared to 1990, the ASDR was decreased by nearly 50%. The trend analysis results are shown in Fig. 15a, b. The ASDRs of stomach cancer demonstrated a downward trend for over 28 years and the largest declination trend was observed from 2004 to 2007 with an APC of − 3.99. Only four countries worldwide showed increasing ASDRs, with an increasing rate of less than 10%. The ASDRs of the remaining 191 countries were significantly dropped and the decreased rate in 52 countries was more than 50%. In addition to slow growth from 2013 to 2017 (APC of 0.25), the ASIRs exhibited a gradual decrease in the remaining 23 years. The ASIR of stomach cancer has increased in only eight countries worldwide, and the other 186 countries showed a declination. There are 93 countries in which the ASIR of stomach cancer demonstrated a decrease by more than one-third, and Equatorial Guinea showed the largest declination, which was up to 70%. The burden of stomach cancer was also concentrated in people aged over 50, in which the morbidity and mortality accounted for more than 80% of the overall numbers and the proportion of young people (under 50 years old) was decreased annually (Fig. 15c, d). The morbidity and mortality of stomach cancer in males were two times higher than those in females. In 2017, the global male ASDRs and ASIRs were 15.19 (15.67–14.77) and 21.75 (22.59–21.01), respectively, and those of females were 7.46 (7.68–7.25) and 9.89 (10.20–9.58), respectively.

## Discussion

Although non-communicable diseases are currently the major causes of deaths globally, cancer is expected to be the leading cause of death in every country during the twenty-first century [24]. Cancer incidence is related to five factors including air, water, land, the built-in environment, and social population [25]. Poor air quality and the built-in environment are most closely associated with the incidence of cancer [26]. Previous studies have reported that the concentration of particulate matter (PM) 2.5 (PM ≤ 2.5 μm) showed a positive correlation with the incidence of lung cancer [26–28].

People over 50 years of age are associated with heavy cancer burden, and more than 80% of cancer cases and deaths occur in individuals aged over 50. Death due to prostate cancer, colon and rectal cancer, gallbladder and biliary tract cancer, non-melanoma skin cancer, and bladder cancer more likely occurs in those individuals aged over 70 years, the number of deaths was more than 1.5 times that of those aged 50–69, and prostate cancer demonstrated the highest, i.e., about 3.59 times among these types of cancers. The death due to laryngeal cancer, brain and nervous system cancer, cervical cancer, nasopharyngeal cancer, and other pharyngeal cancers more likely occurred in people age 50–69. The number of incidences with these cancers in this age group was also higher than those over age 70. Therefore, it is necessary to strengthen the prevention and examination strategies of different cancers at different ages.

The cancer spectrum in different regions is also different, which is related to environmental characteristics, eating habits, and lifestyles of different regions. For example, lip and oral cavity cancer demonstrated the highest incidence and mortality in South Asia. One-fourth of the oral cancers are caused due to tobacco usage (smoking and/or chewing), 7–19% due to alcohol drinking, 10–15% due to micronutrient deficiency, and more than 50% due to betel quid chewing in areas with high chewing prevalence [29, 30]. Betel nut is commonly used in south Asian countries such as India and Vietnam. Malaysia, Indonesia, and Sri Lanka have high rates of smoking as well as betel nut addiction, which was more than 70%. The ASIR of malignant skin melanoma in Australasia was higher than when compared globally as Oceania is close to the ozone hole over the Antarctic, and this caused high levels of UV exposure in the area. This resulted in higher incidence of skin cancers, such as melanoma [12, 31]. The reason for high incidence of skin cancer but low mortality is that the basal cell carcinoma and squamous cell carcinoma are formed at the bottom of the outer layer of the skin and are rarely spread to the other parts of the body. Thus, they are not considered as deadly as melanoma and can be usually removed by simple surgery. The incidence of cancer burden is greater in high HDI countries when compared with low developed countries, but a greater proportion of global mortality burden is observed in the latter countries, which is associated with a higher mean fatality rate. According to a study, low and medium HDI countries experienced a 100% and 81% increase in the cancer incidence by 2030, respectively [32]. Different cancer profiles were observed at each HDI level, and the dose–response associations between the incidence of cancers [33], of TBL, breast, colon and rectum, pancreatic, brain and nervous system, thyroid cancers, and leukemia showed a positive association with human development in 2017.

The cancer burden in North America was dramatically decreased since 1990, and the ASDR of neoplasms was ranged from 160.51 (161.61–157.32) in 1990 to 127.60 (131.52–124.69) in 2017. ASDR in 25 of 29 cancers showed a decreased trend, and the decrease of stomach cancer, laryngeal cancer, breast cancer, prostate cancer, colon and rectal cancer, nasopharyngeal cancer, gallbladder and biliary tract cancer, and Hodgkin lymphoma was more than 30%. This is closely related to tobacco control, demonstrating effective implementation of cancer screening and early diagnosis and clinical treatment level in North America. Several studies have showed that stomach cancer hospitalizations showed a significant declination in the USA, which is due to the declination in the incidence of stomach cancer and advances in outpatient management. Also the declination of the incidence rates of stomach cancer is correlated with the overall declination and improved management of *Helicobacter pylori* infection [34]. But the healthcare costs and burden of stomach cancer has increased, requiring much attention by the government [35]. Moreover, it should be noted that the ASDR of liver cancer in 2017 was increased by 75% when compared with 1990 in North America, and the ASDR of TBL cancer [34.09(34.99-33.25)] accounted for more than 25% of all cancers in 2017. So, the prevention and treatment of these two cancers should be strengthened.

The ASIR of breast cancer is higher in developed countries (high HDI). The risk factors of breast cancer are related to family history (BRCA1or BRCA2 mutations), influence endogenous estrogen exposure (such as early age at menarche, later age at menopause, late age at first birth, nulliparity, and fewer children), alcohol drinking, physical inactivity, excess body weight. The breastfeeding and physical activity are known to reduce breast cancer risk [24, 36]. The 20% and 50% of breast cancer can be prevented if setting the population wide primary prevention efforts [37]. Primary liver cancer includes hepatocellular carcinoma (HCC) and intrahepatic cholangiocarcinoma and other rare types, and HCC accounted for 75–85% among them. The main risk factors for HCC are chronic infection with hepatitis B virus (HBV) or hepatitis C virus (HCV) [24]. The trend of ASIRs showed a Z-shaped change in liver cancer. Prevention of liver cancer through HBV vaccination is widespread, and the improvement of healthcare systems and medical treatment lead to decreased ASIRs from 2000 to 2010. But population aging and growth, and harmful use of alcohol increased the incidence rates of liver cancer due to HCV and alcohol abuse in recent years [4, 11]. According to a recent study, changes in *H*. *pylori* infection patterns after 1952 birth cohort might contributed to the increasing incidence rates of stomach cancer among the younger generations [38]. The declination in the mortality of stomach cancer may include better food preservation and refrigeration, improvement in environmental conditions, lifestyle changes, and treatment advances [39, 40]. The mortality rate of colon and rectal cancer was second highest in 29 cancers, and also has obvious regional characteristics. The ASDR and ASIR of colon and rectal cancer are serious in high HDI countries compared with low developed countries. The risk factors are attributed to “Western lifestyles,” such as processed meat, alcohol, tobacco, etc. The colon and rectal cancer screening could reduce the mortality in high-income countries effectively [41, 42]. The ASIR of prostate cancer is increasing globally, and the ASDR have been decreasing in many countries, especially in developed countries, such as Northern America, Western Europe, Oceania, etc. It has been due to the earlier diagnosis, improved treatment, postpone of death with metastatic disease. But the increasing ASIR and decreased ASDR lead to the increased disability at the global level [43, 44].

Cancer controlling assists in extending the life expectancy and the ability to live longer and healthier lives. Studies have shown that more than 40% of cancers could be prevented [45], and so early prevention of and health education regarding cancer are considered essential. Raising public awareness regarding the risk factors of cancer, such as smoking, drinking, high-protein and high-fat foods, lack of exercise, and environmental pollution, as well as reduction in exposure to risk factors (such as chewing betel nut, *H*. *pylori* infection, etc.) and performing targeted screening for different diseases in the early stages greatly reduces the cancer risk, relieving cancer burden.

## Conclusions

The results of the 2017 global cancer data have suggested that the incidence of cancer in different countries/regions around the world varied. These differences were due to the combination of population composition and social and economic factors, as well as lifestyle in different regions and countries. For certain types of cancers, the risk factors may also vary between different regions and countries. Therefore, it is necessary to understand and consider the common etiological factors that lead to the occurrence of different cancers. These considerations can guide in the development of control strategies of cancer and more interventions are targeted based on local characteristics and environments.

### Limitations

Although GBD estimates filled the gaps in the actual data burden of disease burden or are unavailable, there are some limitations that should be noted. Firstly, the accuracy of the GBD estimates depends on the quality and quantity of the data sources available to inform these estimates. This is because the low proportion of population in South America, Asia, and Africa is covered by high-quality cancer registries. The estimates are widely uncertain, and it remains crucial to improve data collection by expansion and creation of vital registration systems, cancer registries, health surveys, and other data systems. Secondly, underreporting and failure of diagnosis could be the sources of bias during cancer registration, especially in the less developed countries, and therefore some of the cancers estimates might suffer from underestimation

## Additional files

Additional file 1: **Table S1.** The age standardized of global death and incidence of 29 specified cancer groups in 2017 by gender. **Table S2.** The cancer cases of global death and incidence of 29 specified cancer groups in 2017 by gender. **Table S3.** Age-Specific Global Contributions of Cancer Types to Total Cancer incidence in 2017. (DOCX 32 kb)

Additional file 2: Age-standardized deaths of 29 specified cancer groups for 21 regions in 2017. (PDF 67 kb)

Additional file 3: Age-standardized incidence of 29 specified cancer groups for 21 regions in 2017. (PDF 68 kb)

Additional file 4: Death number of 29 specified cancer groups for 21 regions in 2017. (PDF 70 kb)

Additional file 5: Incidence number of 29 specified cancer groups for 21 regions in 2017. (PDF 71 kb)

Additional file 6: The age standardized of death of cancers for 21 regions compared with the global’s in 2017(%). (PDF 148 kb)

Additional file 7: The age standardized of incidence of cancers for 21 regions compared with the global’s in 2017(%). (PDF 148 kb)

Additional file 8: The number, age standardized of death and incidence of 29 specified cancer groups in 2017 by different HDI regions. (PDF 54 kb)

Additional file 9: The ASDR of 29 specified cancer groups in 2017 and 1990, and relative changes between 1990 and 2017 in 195 countries and territories. (PDF 1561 kb)

Additional file 10: The ASIR of 29 specified cancer groups in 2017 and 1990, and relative changes between 1990 and 2017 in 195 countries and territories. (PDF 1526 kb)

## Data Availability

The datasets generated and/or analysed during the current study are available in the GBD repository, http://ghdx.healthdata.org/gbd-results-tool.

## Abbreviations

APC: Annual percentage change
ASDR: Age-standardized death rates
ASIR: Age-standardized incidence rates
CI5: Cancer Incidence in Five Continents
GBD: Global burden of disease
HBV: Hepatitis B virus
HCC: Hepatocellular carcinoma
HCV: Hepatitis C virus
HDI: Human Development Index
ICD: Classification of Diseases
NCI: National Cancer Institute
NORDCAN: Nordic Cancer Registries database
PM: Particulate matter
SDI: Sociodemographic index
SEER: Surveillance, epidemiology, and end results
TBL: Tracheal, bronchus, and lung
