# Supplementary material for: Incidence and death in 29 cancer groups in 2017 and trend analysis from 1990 to 2017 from the Global Burden of Disease Study
Source: J Hematol Oncol. 2019 Sep 12;12:96. doi: 10.1186/s13045-019-0783-9 (PMC6740016; doi:10.1186/s13045-019-0783-9)
Supplement: Supplementary file 1 — Table S1. The age standardized of global death and incidence of 29 specified cancer groups in 2017 by gender. Table S2. The cancer cases of global death and incidence of 29 specified cancer groups in 2017 by gender. Table S3. Age-Specific Global Contributions of Cancer Types to Total Cancer incidence in 2017. (DOCX 32 kb) [file 13045_2019_783_MOESM1_ESM.docx]

Table S1. The age standardized of global death and incidence of 29 specified cancer groups in 2017 by gender.

| **Tumor types** | **Age-standardized deaths per 100,000 (95% UI)** | | **Age-standardized incidence per 100,000 (95% UI)** | |
| --- | --- | --- | --- | --- |
|  | **Female** | **Male** | **Female** | **Male** |
| Esophageal cancer | 2.93(3.05-2.81) | 8.43(8.7-8.15) | 3.32(3.47-3.17) | 8.87(9.16-8.55) |
| Stomach cancer | 7.46(7.68-7.25) | 15.19(15.67-14.77) | 9.89(10.2-9.58) | 21.75(22.59-21.01) |
| Liver cancer | 5.8(6.03-5.59) | 15.1(16.08-14.37) | 6.19(6.46-5.96) | 17.91(19.06-17.03) |
| Larynx cancer | 0.49(0.51-0.47) | 2.8(2.88-2.72) | 0.77(0.79-0.74) | 4.63(4.74-4.52) |
| Tracheal, bronchus, and lung cancer | 13.94(14.36-13.53) | 35.38(36.35-34.39) | 16.26(16.74-15.77) | 39.9(41.13-38.72) |
| Breast cancer | 14.15(14.84-13.63) | 0.3(0.31-0.29) | 45.91(47.4-44.24) | 0.61(0.64-0.59) |
| Cervical cancer | 6.15(6.37-5.71) |  | 14.52(15.1-13.4) |  |
| Uterine cancer | 2(2.05-1.95) |  | 9.57(9.83-9.33) |  |
| Prostate cancer |  | 13.11(15.33-11.18) |  | 37.86(47.99-33.03) |
| Colon and rectum cancer | 9.65(9.87-9.35) | 13.79(14.22-13.3) | 19.17(19.65-18.6) | 27.99(28.87-26.99) |
| Lip and oral cavity cancer | 1.61(1.7-1.53) | 3.31(3.46-3.1) | 3.6(3.79-3.42) | 6.24(6.5-5.91) |
| Nasopharynx cancer | 0.44(0.46-0.42) | 1.32(1.39-1.25) | 0.69(0.74-0.65) | 2.05(2.18-1.92) |
| Other pharynx cancer | 0.79(0.84-0.74) | 2.17(2.37-1.8) | 1.13(1.2-1.06) | 3.34(3.57-2.9) |
| Gallbladder and biliary tract cancer | 2.39(2.56-2.07) | 2.06(2.27-1.75) | 2.81(3.06-2.43) | 2.58(2.87-2.19) |
| Pancreatic cancer | 5.02(5.13-4.91) | 6.28(6.47-6.09) | 5.03(5.16-4.91) | 6.4(6.59-6.21) |
| Malignant skin melanoma | 0.68(0.86-0.52) | 0.91(1.08-0.56) | 3.63(4.96-2.71) | 4.22(5.12-2.4) |
| Non-melanoma skin cancer | 0.5(0.52-0.49) | 1.29(1.32-1.23) | 77.87(106.99-53.64) | 122.15(170.28-83.87) |
| Ovarian cancer | 4.14(4.26-4.03) |  | 6.83(7.05-6.63) |  |
| Testicular cancer |  | 0.2(0.21-0.19) |  | 1.81(1.88-1.74) |
| Kidney cancer | 1.15(1.19-1.01) | 2.51(2.59-2.38) | 3.68(3.82-3.4) | 6.38(6.58-5.98) |
| Bladder cancer | 1.2(1.24-1.17) | 4.39(4.67-4.24) | 2.61(2.68-2.52) | 10.3(10.8-9.96) |
| Brain and nervous system cancer | 2.6(2.91-1.86) | 3.68(4.11-3.1) | 4.59(5.33-3.3) | 5.79(6.52-4.94) |
| Thyroid cancer | 0.57(0.63-0.54) | 0.47(0.49-0.45) | 4.34(4.73-4.12) | 1.94(2.02-1.86) |
| Mesothelioma | 0.19(0.2-0.19) | 0.61(0.64-0.59) | 0.24(0.25-0.23) | 0.68(0.7-0.65) |
| Hodgkin lymphoma | 0.29(0.35-0.24) | 0.54(0.67-0.44) | 1.03(1.23-0.87) | 1.56(1.91-1.29) |
| Non-Hodgkin lymphoma | 2.49(2.56-2.42) | 3.98(4.08-3.86) | 5(5.12-4.85) | 7.51(7.7-7.29) |
| Multiple myeloma | 1.22(1.37-1.14) | 1.55(1.79-1.3) | 1.65(1.92-1.56) | 2.25(2.66-1.87) |
| Leukemia | 3.69(3.95-3.22) | 5.48(5.8-4.98) | 5.62(6.07-4.85) | 8.09(8.62-7.25) |
| Other malignant neoplasms | 4.18(4.41-3.78) | 5.09(5.3-4.55) | 8.21(8.71-7.47) | 10.25(10.75-9.1) |

Table S2. The cancer cases of global death and incidence of 29 specified cancer groups in 2017 by gender.

| **Tumor types** | **Deaths number** | | **Incidence number** | |
| --- | --- | --- | --- | --- |
|  | **Female** | **Male** | **Female** | **Male** |
| Esophageal cancer | 125226(130421-120125) | 310733(320551-300244) | 141621(147968-135089) | 330904(341987-319159) |
| Stomach cancer | 318548(327854-309796) | 546441(564028-530918) | 421353(434424-408084) | 799309(830413-771025) |
| Liver cancer | 247308(257298-238516) | 572128(609965-543395) | 263537(274789-253684) | 689539(734157-653596) |
| Larynx cancer | 20858(21696-19989) | 105613(108754-102690) | 32609(33693-31537) | 177996(182529-173945) |
| Tracheal, bronchus, and lung cancer | 596287(614200-578640) | 1286779(1322428-1249980) | 694808(715160-673794) | 1468324(1513723-1423592) |
| Breast cancer | 600728(629932-578725) | 10897(11370-10467) | 1937574(2000363-1868019) | 23108(23999-22258) |
| Cervical cancer | 259671(269214-241128) |  | 601186(625402-554455) |  |
| Uterine cancer | 85239(87446-83186) |  | 406793(418006-396709) |  |
| Prostate cancer |  | 415910(489540-357283) |  | 1334315(1697900-1170862) |
| Colon and rectum cancer | 413549(423363-401090) | 482491(497797-464669) | 818868(839422-795122) | 1014583(1046837-977080) |
| Lip and oral cavity cancer | 68618(72311-65225) | 125079(131196-117034) | 151250(159081-143804) | 238509(248532-225742) |
| Nasopharynx cancer | 18557(19395-17766) | 50993(53619-48356) | 28531(30487-26844) | 81249(86583-76112) |
| Other pharynx cancer | 33496(35769-31175) | 83916(91322-69824) | 47829(50689-44874) | 131496(140668-114399) |
| Gallbladder and biliary tract cancer | 102422(109618-88786) | 71552(78997-60221) | 120498(131258-103966) | 90380(99871-76937) |
| Pancreatic cancer | 214985(219733-210618) | 226097(232997-219148) | 215425(221009-210240) | 232240(239167-225056) |
| Malignant skin melanoma | 28713(36452-22006) | 32952(39187-20447) | 151889(207335-113045) | 156795(193565-90729) |
| Non-melanoma skin cancer | 21751(22301-21216) | 43346(44568-41440) | 3313779(4558147-2276388) | 4349809(6035291-2973524) |
| Ovarian cancer | 175982(181198-171384) |  | 286127(295311-278075) |  |
| Testicular cancer |  | 7662(8013-7369) |  | 71348(74442-68780) |
| Kidney cancer | 48907(50647-42921) | 89619(92523-84639) | 152271(157886-140667) | 240771(248694-225639) |
| Bladder cancer | 51841(53331-50309) | 144705(153988-139565) | 111492(114786-107976) | 362308(380149-350363) |
| Brain and nervous system cancer | 106865(119108-76088) | 140278(157736-118009) | 183747(212784-131585) | 221472(250709-189404) |
| Thyroid cancer | 24076(26832-23057) | 17159(17772-16412) | 179401(195543-170401) | 76088(79289-72576) |
| Mesothelioma | 8176(8573-7878) | 21732(22471-21028) | 10038(10685-9548) | 24577(25501-23696) |
| Hodgkin lymphoma | 11840(14110-9576) | 20720(25767-16822) | 40381(48368-34446) | 60751(74882-50470) |
| Non-Hodgkin lymphoma | 104384(107281-101571) | 144252(147944-139829) | 208713(213852-202745) | 279251(286418-271149) |
| Multiple myeloma | 51955(58432-48874) | 55160(63975-46376) | 70345(81930-66568) | 82401(97715-69515) |
| Leukemia | 150313(160485-131440) | 197270(209076-178443) | 223099(239786-193548) | 295386(314881-262628) |
| Other malignant neoplasms | 172771(182167-156106) | 186776(194430-166880) | 332740(352663-302850) | 382806(401138-339943) |
| Total | 4063065 | 5390260 | 11145907 | 13215716 |

Table S3. Age-Specific Global Contributions of Cancer Types to Total Cancer incidence in 2017.

| **Tumor types** | **Deaths number** | | | | | **Incidence number** | | | | |
| --- | --- | --- | --- | --- | --- | --- | --- | --- | --- | --- |
|  | **Under 5** | **5-14 years** | **15-49 years** | **50-69 years** | **70+ years** | **Under 5** | **5-14 years** | **15-49 years** | **50-69 years** | **70+ years** |
| Esophageal cancer |  |  | 29257 | 205000 | 201702 |  |  | 40737 | 234500 | 197287 |
| Stomach cancer |  |  | 76160 | 354528 | 434301 |  |  | 136696 | 550689 | 533277 |
| Liver cancer |  | 1619 | 108824 | 399616 | 309376 |  | 2117 | 164976 | 483538 | 302445 |
| Larynx cancer |  |  | 13207 | 69647 | 43616 |  |  | 24373 | 126337 | 59896 |
| Tracheal, bronchus, and lung cancer |  |  | 105458 | 861533 | 916075 |  |  | 143168 | 1053992 | 965972 |
| Breast cancer |  |  | 123211 | 278263 | 210151 |  |  | 578148 | 919613 | 462920 |
| Cervical cancer |  |  | 70334 | 121165 | 68172 |  |  | 308158 | 225913 | 67114 |
| Uterine cancer |  |  | 7762 | 39115 | 38361 |  |  | 66504 | 235323 | 104966 |
| Prostate cancer |  |  | 3338 | 89895 | 322677 |  |  | 27750 | 586011 | 720553 |
| Colon and rectum cancer |  |  | 68170 | 323185 | 504685 |  |  | 190132 | 787458 | 855861 |
| Lip and oral cavity cancer |  |  | 32028 | 94411 | 67257 |  |  | 90440 | 186900 | 112420 |
| Nasopharynx cancer |  | 468 | 14448 | 36794 | 17839 |  | 2697 | 44327 | 47351 | 15406 |
| Other pharynx cancer |  |  | 17889 | 66356 | 33166 |  |  | 32314 | 109326 | 37686 |
| Gallbladder and biliary tract cancer |  |  | 10079 | 64520 | 99376 |  |  | 14323 | 80870 | 115686 |
| Pancreatic cancer |  |  | 25238 | 178037 | 237808 |  |  | 31310 | 188059 | 228296 |
| Malignant skin melanoma |  |  | 10592 | 23347 | 27727 |  |  | 92147 | 119092 | 97445 |
| Non-melanoma skin cancer |  |  | 4899 | 20906 | 39292 |  |  | 838599 | 3263198 | 3561792 |
| Ovarian cancer |  |  | 25732 | 85413 | 64836 |  |  | 91373 | 128300 | 66453 |
| Testicular cancer |  |  | 5088 | 1351 | 1223 |  |  | 61231 | 7448 | 2669 |
| Kidney cancer | 1887 | 949 | 10554 | 56228 | 68908 | 15147 | 7699 | 67869 | 181288 | 121039 |
| Bladder cancer |  |  | 6754 | 56055 | 133737 |  |  | 37412 | 192649 | 243739 |
| Brain and nervous system cancer | 8408 | 13276 | 59249 | 101474 | 64735 | 27068 | 31495 | 137668 | 134522 | 74466 |
| Thyroid cancer |  | 168 | 6035 | 15467 | 19564 |  | 1772 | 108309 | 105885 | 39522 |
| Mesothelioma |  |  | 2961 | 11320 | 15628 |  |  | 4761 | 14393 | 15462 |
| Hodgkin lymphoma | 703 | 2181 | 12930 | 9875 | 6871 | 2522 | 6442 | 58375 | 24092 | 9701 |
| Non-Hodgkin lymphoma | 5079 | 6136 | 39831 | 89951 | 107639 | 10203 | 13200 | 103520 | 192304 | 168737 |
| Multiple myeloma |  |  | 7472 | 42225 | 57417 |  |  | 15454 | 69523 | 67768 |
| Leukemia | 16075 | 22928 | 73412 | 104600 | 130568 | 52861 | 45509 | 123126 | 149709 | 147280 |
| Other malignant neoplasms | 15895 | 12307 | 65144 | 132377 | 133825 | 51372 | 31581 | 194584 | 242521 | 195488 |
| Total | 48047 | 60034 | 1036053 | 3932657 | 4376534 | 159173 | 142514 | 3827784 | 10640807 | 9591345 |
