# Supplementary material for: Incidence and death in 29 cancer groups in 2017 and trend analysis from 1990 to 2017 from the Global Burden of Disease Study
Source: J Hematol Oncol. 2019 Sep 12;12:96. doi: 10.1186/s13045-019-0783-9 (PMC6740016; doi:10.1186/s13045-019-0783-9)
Supplement: Supplementary file 2 — Age-standardized deaths of 29 specified cancer groups for 21 regions in 2017. (PDF 67 kb) [file 13045_2019_783_MOESM2_ESM.pdf]

Age-standardized deaths (per 100,000) for 21 regions in 2017

Tumor types	Global	Southeast Asia	Southeast Asia	Oceania	Central Asia	Central Europe	Eastern Europe	High-income Asia Pacific	Australasia	Western Europe	Southern Latin America
Esophageal cancer	5.48(5.63-5.34)	11.09(11.59-10.59)	2.6(2.79-2.45)	2.31(2.64-2)	5.99(6.27-5.71)	2.73(2.83-2.63)	2.91(3-2.84)	3.28(3.42-3.14)	3.77(4.15-3.42)	3.41(3.54-3.28)	4.31(4.71-3.99)
Stomach cancer	10.98(11.23-10.77)	18.68(19.48-17.95)	7.02(7.55-6.57)	14(16.15-11.93)	14.34(14.94-13.76)	8.63(8.88-8.38)	12.77(13.13-12.47)	14.2(14.82-13.66)	4.44(4.83-4.08)	6.43(6.67-6.18)	12.29(13.24-11.46)
Liver cancer	10.21(10.65-9.85)	21.47(22.84-20.29)	12.05(12.93-11.07)	10.08(13.22-6.6)	9.28(9.74-8.83)	5.23(5.42-5.05)	3.93(4.15-3.75)	10.3(10.97-9.36)	4.31(4.72-3.91)	5.15(5.33-4.97)	4.5(4.89-4.13)
Larynx cancer	1.57(1.61-1.53)	1(1.05-0.96)	1.38(1.68-1.27)	1.88(2.18-1.65)	1.64(1.73-1.55)	2.44(2.54-2.34)	2.02(2.08-1.96)	0.36(0.38-0.35)	0.6(0.68-0.54)	1.08(1.13-1.03)	1.48(1.64-1.33)
Tracheal, bronchus, and lung cancer	23.74(24.24-23.25)	35.94(37.34-34.41)	21.94(24.18-20.1)	24.59(32.06-20.68)	16.12(16.78-15.47)	34.3(35.35-33.25)	21.1(21.55-20.66)	20.77(21.43-20.1)	22.32(24.23-20.41)	27.2(28.2-26.23)	20.01(21.66-18.53)
Breast cancer	7.65(8.01-7.37)	4.55(4.87-3.81)	8.08(8.77-7.51)	11.59(14.66-9.48)	7.89(8.34-7.44)	9.96(10.37-9.57)	9.72(10.01-9.45)	4.78(5-4.57)	8.91(9.97-7.89)	10(10.47-9.55)	11.1(12.47-9.97)
Cervical cancer	3.21(3.32-2.98)	2.31(2.49-1.58)	4.17(4.64-3.58)	9.97(12.31-7.92)	3.5(3.74-3.28)	3.55(3.73-3.39)	2.61(2.7-2.52)	1.36(1.43-1.3)	1.22(1.4-1.07)	1.4(1.47-1.33)	5.43(6.13-4.82)
Uterine cancer	1.07(1.1-1.05)	0.63(0.68-0.59)	1.1(1.24-0.99)	3.28(3.92-2.74)	1.44(1.53-1.35)	1.81(1.89-1.73)	2.08(2.15-2.01)	0.8(0.85-0.76)	1.26(1.43-1.11)	1.22(1.29-1.16)	1.52(1.71-1.36)
Prostate cancer	5.5(6.46-4.71)	3.06(3.77-2.54)	4.79(5.5-3.88)	8.79(10.12-6.8)	4.09(4.48-2.96)	6.86(7.71-5.25)	4.49(4.81-2.84)	3.17(3.9-2.31)	8.29(10.9-6.72)	7.24(9.75-6.01)	9.35(11.9-7.4)
Colon and rectum cancer	11.52(11.77-11.27)	10.27(10.7-9.75)	10.33(11-9.81)	9.34(12.27-8.14)	9.02(9.39-8.61)	20.92(21.57-20.31)	16.4(16.88-16.01)	14.08(14.74-13.57)	15.2(16.43-14.03)	14.72(15.3-14.14)	16.08(17.38-14.89)
Lip and oral cavity cancer	2.42(2.52-2.31)	1.17(1.23-1.11)	2.44(2.58-2.3)	2.68(3.5-2.29)	1.73(1.82-1.64)	2.7(2.81-2.6)	2.51(2.61-2.43)	1.1(1.16-1.06)	1.68(1.85-1.52)	1.53(1.6-1.46)	1.28(1.41-1.17)
Nasopharynx cancer	0.86(0.89-0.82)	1.42(1.51-1.34)	1.73(1.93-1.57)	2.06(2.49-1.69)	0.36(0.38-0.33)	0.39(0.4-0.37)	0.31(0.33-0.3)	0.34(0.37-0.32)	0.54(0.6-0.48)	0.47(0.49-0.44)	0.18(0.2-0.17)
Other pharynx cancer	1.45(1.53-1.26)	0.3(0.34-0.27)	0.96(1.15-0.86)	1.31(1.61-1.07)	0.8(0.85-0.74)	1.96(2.06-1.85)	1.56(1.63-1.5)	0.68(0.74-0.65)	0.9(1-0.81)	1.18(1.25-1.11)	0.44(0.48-0.4)
Gallbladder and biliary tract cancer	2.23(2.38-1.99)	1.53(1.66-1.16)	2.06(2.25-1.59)	1.37(1.63-1.08)	1.15(1.22-1.06)	3.03(3.23-2.9)	1.33(1.39-1.27)	5.22(5.49-4.97)	1.65(1.83-1.49)	1.97(2.09-1.87)	6.96(7.67-6.44)
Pancreatic cancer	5.62(5.72-5.52)	4.49(4.67-4.3)	3.62(3.89-3.33)	3.53(4.17-3.16)	5.01(5.22-4.8)	9.06(9.32-8.8)	7.33(7.49-7.18)	8.5(8.82-8.22)	7.28(7.9-6.69)	8.68(9.01-8.38)	8.63(9.33-8)
Malignant skin melanoma	0.78(0.89-0.61)	0.28(0.32-0.2)	0.27(0.36-0.24)	0.78(1.16-0.57)	0.64(0.91-0.55)	1.92(2.14-1.43)	1.82(2.21-1.34)	0.24(0.29-0.17)	4.48(5.25-2.85)	1.89(2.07-1.25)	1.12(1.38-0.79)
Non-melanoma skin cancer	0.85(0.87-0.82)	0.89(0.93-0.84)	0.84(0.91-0.79)	1.26(1.43-1.12)	0.83(0.87-0.8)	1.22(1.26-1.18)	0.95(0.98-0.91)	0.34(0.36-0.33)	1.68(1.85-1.54)	0.71(0.74-0.68)	1.1(1.19-1.02)
Ovarian cancer	2.2(2.26-2.14)	1.27(1.35-1.2)	2.2(2.52-1.96)	1.92(2.38-1.62)	2.12(2.24-2.01)	3.8(3.99-3.62)	3.36(3.49-3.23)	1.65(1.74-1.57)	2.64(3-2.33)	3.17(3.34-3)	2.51(2.83-2.23)
Testicular cancer	0.1(0.1-0.09)	0.04(0.04-0.03)	0.05(0.05-0.04)	0.45(0.52-0.37)	0.16(0.18-0.15)	0.26(0.28-0.25)	0.17(0.17-0.16)	0.04(0.04-0.04)	0.1(0.11-0.08)	0.11(0.12-0.1)	0.45(0.51-0.39)
Kidney cancer	1.77(1.82-1.64)	0.97(1.04-0.86)	1.04(1.12-0.89)	0.85(1.08-0.68)	2.24(2.36-2.13)	3.81(3.99-3.32)	3.78(3.9-3.58)	1.91(2.03-1.69)	3.27(3.59-2.97)	3.26(3.43-2.95)	4.28(4.72-3.9)
Bladder cancer	2.57(2.69-2.51)	1.77(2.03-1.68)	2(2.23-1.69)	1.97(2.24-1.59)	2.2(2.34-2.09)	4.5(4.67-4.34)	2.97(3.06-2.88)	2.04(2.12-1.96)	2.81(3.09-2.56)	4.21(4.38-4.02)	2.97(3.22-2.73)
Brain and nervous system cancer	3.12(3.34-2.68)	3.4(3.88-2.84)	2.78(3.14-2.1)	1.81(2.43-1.23)	3.77(4.16-2.89)	5.11(5.45-4.13)	3.55(4.02-3.19)	1.41(1.56-0.95)	4.17(4.69-3.32)	3.98(4.27-3.12)	2.97(3.37-2.65)
Thyroid cancer	0.52(0.56-0.51)	0.38(0.41-0.35)	0.86(1.02-0.79)	0.83(0.99-0.69)	0.37(0.39-0.35)	0.43(0.45-0.41)	0.49(0.51-0.47)	0.57(0.61-0.55)	0.4(0.44-0.36)	0.41(0.44-0.4)	0.52(0.57-0.48)
Mesothelioma	0.38(0.39-0.37)	0.14(0.15-0.13)	0.22(0.24-0.21)	0.31(0.46-0.24)	0.19(0.2-0.18)	0.3(0.32-0.29)	0.26(0.28-0.25)	0.37(0.4-0.35)	1.79(1.98-1.59)	1.11(1.16-1.06)	0.37(0.41-0.34)
Hodgkin lymphoma	0.41(0.48-0.35)	0.15(0.17-0.11)	0.35(0.42-0.28)	0.44(0.56-0.31)	0.44(0.52-0.37)	0.45(0.53-0.39)	0.55(0.71-0.46)	0.08(0.09-0.06)	0.24(0.29-0.19)	0.34(0.44-0.29)	0.42(0.57-0.36)
Non-Hodgkin lymphoma	3.18(3.24-3.11)	2.21(2.31-2.12)	2.78(2.94-2.61)	2.77(3.66-2.31)	1.88(1.97-1.79)	2.88(2.97-2.8)	2.57(2.64-2.51)	3.18(3.31-3.02)	4.6(4.99-4.2)	3.77(3.91-3.63)	3.62(3.93-3.35)
Multiple myeloma	1.37(1.51-1.25)	0.64(0.69-0.56)	0.7(0.83-0.65)	1.1(1.56-0.91)	0.64(0.76-0.59)	1.63(1.75-1.34)	1.41(1.51-1.09)	1.3(1.53-1.14)	2.63(3.06-2.25)	2.33(2.78-2.02)	1.82(2.16-1.6)
Leukemia	4.5(4.73-4.12)	3.79(4.09-3.2)	5.35(5.8-4.39)	5.6(6.62-4.41)	3.66(3.87-3.45)	4.94(5.11-4.66)	4.42(4.59-4.25)	3.13(3.28-2.98)	5.76(6.26-5.25)	5.38(5.59-5.15)	4.66(5.03-4.32)
Other malignant neoplasms	4.61(4.75-4.25)	3.58(3.79-3.11)	4.59(5.29-4.24)	5.56(6.29-4.86)	5.37(5.78-5.12)	4.85(5.08-4.47)	8.59(9.18-6.39)	2.98(3.17-2.5)	3.65(4.03-3.2)	4.13(4.39-3.63)	4.78(6.08-4.2)

High-income North America	Caribbean	Andean Latin America	Central Latin America	Tropical Latin America	North Africa and Middle East	South Asia	Central Sub-Saharan Africa	Eastern Sub-Saharan Africa	Southern Sub-Saharan Africa	Western Sub-Saharan Africa	
3.47(3.56-3.37)	3.4(3.72-3.13)	1.43(1.57-1.29)	1.48(1.54-1.41)	4.81(4.92-4.7)	2.29(2.48-2.14)	4.11(4.36-3.88)	7.67(9.01-6.54)	8.35(9.17-7.72)	10.53(11.02-10.02)	4.24(5-3.71)	
3.56(3.66-3.47)	7.25(7.81-6.77)	17.11(18.55-15.75)	9.32(9.66-8.98)	9.25(9.43-9.07)	8.76(9.17-8.35)	7.45(7.79-7.02)	7.59(8.47-6.7)	6.77(7.26-6.3)	5.52(5.78-5.28)	8.41(9.23-7.7)	
4.93(5.08-4.78)	5.73(6.18-5.23)	7.31(7.98-6.7)	6.16(6.38-5.94)	5.23(5.37-5.1)	5.94(6.46-5.47)	3.46(3.73-3.18)	7.92(11.75-5.39)	7.81(8.59-7.06)	6.68(7.2-6.23)	11.39(13.38-9.9)	
0.92(0.95-0.9)	3.02(3.31-2.74)	0.75(0.83-0.68)	1.11(1.17-1.05)	2.16(2.22-2.1)	1.66(1.74-1.56)	3.22(3.4-3.04)	1.53(1.81-1.29)	1.33(1.5-1.18)	1.63(1.71-1.54)	1.29(1.52-1.11)	
34.09(34.99-33.25)	20.08(21.56-18.64)	9.76(10.67-8.86)	9.97(10.38-9.59)	14.44(14.8-14.1)	15.23(16-14.39)	9.77(10.4-9.16)	9.63(12.06-8.05)	7.83(8.6-7.26)	15.4(16.12-14.7)	8.71(10.14-7.55)	
9.26(9.57-8.97)	10.1(11.49-8.87)	5.93(6.78-5.24)	6.6(6.91-6.29)	8.14(8.34-7.94)	5.88(6.72-5.52)	7.79(9.37-6.68)	9.23(11.51-7.59)	8.58(9.77-7.55)	9.91(10.66-8.94)	12.5(15.99-9.78)	
1.52(1.59-1.46)	6.02(6.91-5.09)	6.29(7.18-5.43)	4.75(5.01-4.51)	4.3(4.47-4.16)	1.29(1.41-1.14)	3.67(4.45-3.33)	13.17(16.31-9.74)	10.37(12.34-8.73)	10.24(11.23-9.16)	8.54(10.32-6.8)	
1.46(1.51-1.41)	2.43(2.7-2.19)	1.71(1.95-1.51)	1.13(1.2-1.07)	1.14(1.17-1.1)	0.63(0.67-0.59)	0.92(1.01-0.84)	1.14(1.54-0.89)	1.21(1.5-1.04)	1.36(1.49-1.24)	1.05(1.2-0.91)	
6.22(9.33-5.65)	17.44(19.37-12.95)	9.5(12.25-8.29)	7.82(9.55-6.09)	9.27(12.82-8.02)	4.63(5.05-3.27)	3.67(4.41-3.01)	9.32(11.24-6.4)	9.99(11.42-7.3)	12.77(14.39-9.89)	21.57(26.78-14.68)	
14.38(14.77-13.98)	13.26(14.33-12.32)	8.17(8.88-7.41)	8.04(8.33-7.74)	10.28(10.56-9.98)	7.97(8.34-7.62)	7.1(7.65-6.36)	8.79(10.32-7.67)	10.28(10.96-9.51)	9.41(10.01-8.63)	8.45(9.84-7.29)	
1.3(1.34-1.26)	2.29(2.47-2.12)	1.07(1.18-0.97)	1.01(1.05-0.97)	2.27(2.34-2.19)	0.78(0.82-0.75)	6.95(7.45-6.41)	2.06(2.34-1.79)	2.23(2.42-2.04)	2.44(2.6-2.29)	1.31(1.49-1.15)	
0.23(0.23-0.22)	0.56(0.61-0.52)	0.17(0.18-0.15)	0.29(0.31-0.28)	0.23(0.23-0.22)	0.52(0.56-0.49)	0.89(0.94-0.85)	0.48(0.56-0.4)	1.04(1.2-0.88)	0.46(0.49-0.43)	0.44(0.54-0.37)	
0.68(0.71-0.66)	1.04(1.18-0.94)	0.66(0.78-0.59)	0.4(0.42-0.38)	1.52(1.6-1.45)	0.33(0.43-0.3)	5.04(5.5-4.01)	0.48(0.61-0.39)	0.77(0.88-0.6)	0.56(0.63-0.46)	0.42(0.48-0.37)	
0.95(1.04-0.92)	1.22(1.73-1.06)	3.56(4.38-3.13)	2.24(2.68-2.12)	2.34(2.41-2.26)	1.42(1.77-1.24)	3.03(3.41-2.36)	1.35(1.69-1.1)	1.37(1.83-1.14)	1.22(1.39-0.99)	1.35(1.8-1.11)	
8.71(8.97-8.49)	4.85(5.22-4.52)	4.58(5-4.16)	4.45(4.61-4.29)	5.63(5.79-5.49)	3.96(4.15-3.75)	2.88(3.05-2.74)	3.32(3.83-2.86)	3.25(3.59-2.89)	5.53(5.81-5.22)	4.52(5.31-3.91)	
2.03(2.63-1.53)	0.56(0.76-0.47)	0.81(1.09-0.69)	0.64(0.84-0.5)	1(1.46-0.74)	0.47(0.75-0.38)	0.23(0.28-0.17)	0.59(0.82-0.47)	0.71(0.92-0.55)	1.18(1.37-0.84)	0.48(0.64-0.39)	
0.7(0.72-0.67)	1.39(1.5-1.29)	0.99(1.07-0.92)	1.23(1.29-1.18)	1.28(1.32-1.24)	0.45(0.48-0.43)	0.8(0.89-0.73)	1.22(1.41-0.96)	1.42(1.65-0.94)	2.32(2.47-2.01)	0.53(0.63-0.4)	
3.06(3.21-2.92)	2.04(2.29-1.85)	1.96(2.22-1.72)	2.18(2.29-2.08)	2.03(2.13-1.96)	1.53(1.63-1.44)	2.1(2.31-1.93)	1.76(2.25-1.33)	2.51(2.84-2.13)	2.42(2.62-2.19)	1.65(2.05-1.32)	
0.12(0.13-0.11)	0.07(0.08-0.06)	0.21(0.24-0.18)	0.3(0.32-0.29)	0.15(0.16-0.15)	0.08(0.09-0.07)	0.12(0.13-0.11)	0.09(0.11-0.06)	0.05(0.06-0.04)	0.13(0.14-0.12)	0.04(0.06-0.04)	
3.11(3.29-2.98)	1.51(1.77-1.39)	1.82(2.03-1.56)	1.95(2.06-1.85)	1.76(1.84-1.67)	1.07(1.15-0.93)	0.62(0.66-0.56)	0.85(1.11-0.68)	0.77(0.88-0.67)	1.13(1.24-1.01)	0.91(1.06-0.79)	
3.34(3.45-3.25)	2.62(2.84-2.43)	1.4(1.6-1.26)	1.36(1.42-1.3)	2.24(2.31-2.18)	3.35(4.17-3.08)	1.67(1.89-1.55)	2.85(4-2.2)	2.47(2.76-2.14)	2.74(2.95-2.44)	2.34(2.69-1.97)	
3.69(4.07-3.25)	2.59(3.25-1.27)	2.7(3.53-1.42)	2.25(2.42-1.75)	4.45(4.77-3.17)	3.66(4.22-3.04)	2.39(2.76-1.98)	1.51(1.85-1.28)	2.12(2.5-1.65)	1.74(2-1.26)	1.54(1.92-1.14)	
0.39(0.4-0.37)	0.53(0.58-0.48)	0.99(1.09-0.86)	0.69(0.72-0.66)	0.5(0.51-0.48)	0.46(0.55-0.43)	0.65(0.71-0.59)	0.39(0.51-0.3)	0.8(0.92-0.69)	0.4(0.45-0.37)	0.28(0.32-0.23)	
0.6(0.63-0.58)	0.17(0.2-0.15)	0.22(0.25-0.2)	0.24(0.26-0.23)	0.37(0.39-0.35)	0.4(0.44-0.36)	0.21(0.26-0.17)	0.16(0.22-0.12)	0.13(0.17-0.09)	0.63(0.7-0.57)	0.14(0.16-0.11)	
0.3(0.41-0.27)	0.52(0.64-0.28)	0.36(0.41-0.29)	0.45(0.55-0.37)	0.32(0.39-0.26)	0.62(0.72-0.47)	0.53(0.68-0.45)	0.49(0.69-0.38)	0.89(1.2-0.66)	0.32(0.37-0.23)	1(1.35-0.75)	
4.74(4.87-4.62)	3.45(3.79-3.2)	4.02(4.37-3.65)	2.66(2.78-2.55)	2.74(2.82-2.66)	2.81(2.99-2.67)	2.68(2.83-2.5)	2.08(2.67-1.47)	5.63(6.27-4.84)	2.99(3.19-2.78)	3.17(3.68-2.79)	
2.66(3.35-2.45)	2.16(2.54-1.95)	1.5(1.9-1.32)	1.3(1.56-1.18)	1.51(1.77-1.28)	1.2(1.56-1.1)	1.01(1.11-0.89)	1.12(1.42-0.83)	1.63(1.78-1.35)	1.86(2.02-1.45)	1.42(1.69-1.18)	
5.38(5.58-5.22)	5.01(5.67-4.58)	4.91(5.43-4.14)	4.75(4.98-4.55)	4.02(4.13-3.91)	5.36(6.12-4.71)	3.57(3.99-3.11)	3.61(4.38-2.68)	4.04(4.69-3.21)	4.03(4.36-3.33)	3.11(3.69-2.52)	
3.54(3.85-3.35)	5.17(6.28-4.6)	4.47(5.57-3.9)	3.9(4.16-3.52)	4.98(5.76-4.67)	4.16(4.81-3.87)	4.6(4.93-4.01)	5.09(6.97-3.45)	8.86(9.99-7.3)	4.52(4.87-4.03)	5.37(6.38-4.57)	
