# Supplementary material for: Incidence and death in 29 cancer groups in 2017 and trend analysis from 1990 to 2017 from the Global Burden of Disease Study
Source: J Hematol Oncol. 2019 Sep 12;12:96. doi: 10.1186/s13045-019-0783-9 (PMC6740016; doi:10.1186/s13045-019-0783-9)
Supplement: Supplementary file 3 — Age-standardized incidence of 29 specified cancer groups for 21 regions in 2017. (PDF 68 kb) [file 13045_2019_783_MOESM3_ESM.pdf]

Age-standardized incidence (per 100,000) for 21 regions in 2017

Tumor types	Global	Southeast Asia	Southeast Asia	Oceania	Central Asia	Central Europe	Eastern Europe	High-income Asia Pacific	Australasia	Western Europe	Southern Latin America
Esophageal cancer	5.9(6.06-5.74)	12.09(12.67-11.51)	2.51(2.69-2.36)	2.21(2.54-1.91)	5.67(5.93-5.4)	2.68(2.79-2.58)	3.46(3.58-3.36)	5.15(5.41-4.87)	4.45(4.9-4.03)	4(4.18-3.82)	4.01(4.41-3.72)
Stomach cancer	15.36(15.78-14.97)	28.6(29.98-27.26)	6.82(7.34-6.37)	13.83(16.09-11.72)	14.12(14.7-13.52)	9.38(9.67-9.09)	17.74(18.29-17.21)	29.49(30.96-28.18)	8.78(9.68-7.91)	10.49(10.98-9.96)	12.35(13.35-11.47)
Liver cancer	11.8(12.34-11.35)	26.2(28.03-24.64)	11.66(12.57-10.69)	9.99(13.11-6.5)	8.9(9.35-8.46)	5.04(5.23-4.86)	3.88(4.11-3.7)	15.14(16.45-13.65)	5.23(5.88-4.65)	6.38(6.66-6.11)	4.17(4.54-3.84)
Larynx cancer	2.59(2.65-2.54)	1.98(2.08-1.89)	1.89(2.23-1.73)	2.05(2.36-1.79)	2.2(2.32-2.07)	4.39(4.59-4.2)	3.67(3.81-3.54)	1.64(1.74-1.56)	2.08(2.38-1.82)	3.19(3.37-3.03)	2.32(2.59-2.08)
Tracheal, bronchus, and lung cancer	27.13(27.75-26.55)	41.54(43.37-39.8)	20.67(22.72-18.93)	23.56(31.02-19.68)	15.63(16.3-14.98)	35.42(36.51-34.3)	26.33(27.21-25.44)	28.92(30.06-27.77)	31.69(34.62-28.91)	34.11(35.49-32.7)	19.12(20.7-17.69)
Breast cancer	24.19(24.96-23.34)	18.41(19.86-15.49)	18.7(20.19-17.22)	20.15(26.69-15.82)	19.66(21-18.43)	32.05(33.7-30.6)	30.64(31.85-29.43)	27.03(28.75-25.31)	44.22(50.73-38.38)	45.41(47.65-43.21)	28.78(32.45-25.69)
Cervical cancer	7.38(7.67-6.81)	5.51(5.98-3.62)	9.15(10.17-7.77)	23.36(30.83-17.05)	7.89(8.6-7.28)	8.01(8.51-7.55)	7.71(8.19-7.28)	5.46(5.92-5.08)	3.24(3.82-2.73)	4.26(4.54-4.01)	14.85(17.29-12.76)
Uterine cancer	5(5.14-4.88)	3.34(3.6-3.11)	2.98(3.29-2.66)	6.42(7.78-5.31)	5.65(6.06-5.24)	10.63(11.23-10.05)	9.1(9.64-8.62)	4.54(4.93-4.19)	7.68(8.88-6.59)	10.02(10.63-9.43)	5.27(5.95-4.67)
Prostate cancer	16.94(21.53-14.84)	7.95(10.06-6.78)	7.78(9.23-6.47)	10.77(12.4-8.28)	6.88(7.61-4.9)	17.69(19.8-13.22)	16.12(18.1-9.94)	15.09(19.63-11.48)	49.38(64.28-37.45)	34.78(49.39-29.47)	20.01(25.83-15.68)
Colon and rectum cancer	23.22(23.73-22.7)	22.81(23.86-21.64)	14.71(15.62-13.97)	11.23(14.77-9.75)	12.35(12.88-11.8)	34.55(35.56-33.52)	30.18(31.19-29.31)	41.93(44.13-40.19)	46.43(50.6-42.48)	38.68(40.31-37.08)	25.47(27.65-23.64)
Lip and oral cavity cancer	4.84(5.02-4.65)	2.71(2.83-2.57)	4.65(4.94-4.36)	4.35(5.68-3.67)	2.96(3.13-2.8)	4.74(4.95-4.56)	4.96(5.19-4.76)	3.5(3.75-3.3)	5.91(6.64-5.24)	4.9(5.16-4.67)	2.72(3-2.46)
Nasopharynx cancer	1.35(1.42-1.28)	2.42(2.66-2.22)	2.54(2.85-2.28)	3.01(3.91-2.3)	0.52(0.64-0.45)	0.51(0.56-0.46)	0.59(0.66-0.52)	0.48(0.53-0.43)	0.77(0.92-0.64)	0.81(0.89-0.75)	0.28(0.33-0.23)
Other pharynx cancer	2.19(2.3-1.96)	0.6(0.66-0.53)	1.3(1.55-1.16)	1.54(1.93-1.23)	1.14(1.22-1.06)	3.44(3.6-3.25)	2.87(3-2.75)	1.61(1.75-1.5)	2.29(2.58-2.02)	3.11(3.31-2.93)	0.7(0.78-0.63)
Gallbladder and biliary tract cancer	2.71(2.89-2.39)	1.77(1.94-1.31)	2.08(2.3-1.59)	1.31(1.56-1.02)	1.07(1.13-0.99)	3.12(3.31-2.94)	1.49(1.62-1.4)	8.73(10.31-7.34)	2.42(3.07-2)	2.98(3.29-2.7)	7.01(7.77-6.45)
Pancreatic cancer	5.69(5.8-5.57)	4.38(4.56-4.18)	3.37(3.63-3.1)	3.33(3.96-2.96)	4.68(4.88-4.48)	8.76(9.02-8.5)	7.59(7.79-7.42)	9.83(10.34-9.33)	8.31(9.09-7.56)	8.97(9.35-8.62)	8(8.65-7.42)
Malignant skin melanoma	3.87(4.58-2.97)	0.88(1.03-0.59)	0.4(0.52-0.35)	0.83(1.24-0.6)	1.37(1.98-1.19)	8.16(9.37-6.21)	7.01(8.91-5.23)	2.01(2.53-1.46)	48.33(58.1-33.54)	16.17(18.22-11.05)	3.19(3.98-2.36)
Non-melanoma skin cancer	97.11(134.21-66.66)	13.13(17.5-9.58)	10.11(15.14-6.85)	17.98(25.53-13.18)	58.43(91.88-33.09)	60.82(89.03-38.98)	63.05(97.47-36.47)	8.92(13.58-5.62)	473.95(665.03-311.73)	83.76(115.1-59.06)	23.97(26.17-21.99)
Ovarian cancer	3.54(3.66-3.44)	2.12(2.25-2)	4.13(4.78-3.66)	3.45(4.49-2.72)	3.34(3.54-3.14)	5.88(6.17-5.6)	5.59(5.89-5.29)	3.66(3.91-3.42)	4.37(5.01-3.81)	5.28(5.55-4.99)	3.99(4.53-3.55)
Testicular cancer	0.9(0.94-0.87)	0.36(0.39-0.33)	0.26(0.3-0.23)	0.73(0.9-0.59)	0.65(0.72-0.59)	4.16(4.59-3.78)	1.16(1.3-1.03)	1.59(1.81-1.37)	3.46(4.24-2.76)	3.89(4.3-3.51)	5.05(6.05-4.19)
Kidney cancer	4.94(5.08-4.66)	2.77(2.98-2.49)	3.32(3.61-2.84)	2.91(3.75-2.27)	6.28(6.67-5.87)	8.65(9.07-7.46)	10.02(10.47-9.49)	4.42(4.83-3.94)	8.79(9.8-7.9)	9.16(9.69-8.27)	11.61(13.02-10.39)
Bladder cancer	6.04(6.27-5.9)	3.98(4.52-3.77)	3.78(4.17-3.24)	3.34(3.77-2.75)	4.48(4.72-4.26)	10.95(11.35-10.54)	7.49(7.76-7.21)	6.34(6.68-5.99)	8.25(9.14-7.43)	12.96(13.6-12.32)	6.67(7.3-6.12)
Brain and nervous system cancer	5.17(5.64-4.46)	7.79(9.41-6.36)	3.31(3.74-2.53)	2.11(2.88-1.42)	4.85(5.41-3.68)	8.41(9.1-6.95)	6.92(8.6-6.25)	5.55(6.22-3.88)	7.43(8.62-6.13)	10.45(11.53-8.58)	3.63(4.22-3.25)
Thyroid cancer	3.15(3.36-3.03)	2.22(2.53-2.06)	3.55(4.33-3.21)	2.14(2.65-1.7)	2.01(2.18-1.85)	3.77(4.06-3.54)	5.03(5.4-4.7)	7.21(8.08-6.5)	5.29(6.04-4.61)	4.57(4.87-4.31)	3.06(3.44-2.75)
Mesothelioma	0.44(0.45-0.42)	0.15(0.16-0.14)	0.28(0.31-0.26)	0.4(0.6-0.3)	0.26(0.28-0.25)	0.42(0.45-0.38)	0.38(0.41-0.35)	0.39(0.43-0.37)	2.13(2.47-1.84)	1.31(1.38-1.24)	0.43(0.49-0.39)
Hodgkin lymphoma	1.29(1.51-1.12)	1.18(1.34-0.86)	0.53(0.65-0.44)	0.54(0.68-0.38)	1.07(1.24-0.89)	2.88(3.51-2.52)	3.99(5.19-3.43)	0.86(1-0.61)	3.26(3.92-2.6)	3.58(4.96-3.18)	0.95(1.33-0.79)
Non-Hodgkin lymphoma	6.18(6.29-6.06)	4.55(4.76-4.32)	3.46(3.67-3.24)	3.11(4.16-2.63)	2.98(3.14-2.83)	7.1(7.4-6.84)	6.14(6.46-5.84)	8.61(9.22-7.99)	15.91(17.54-14.31)	12.67(13.24-12.11)	5.48(5.97-5.08)
Multiple myeloma	1.92(2.17-1.76)	1(1.08-0.84)	0.79(0.94-0.73)	1.15(1.65-0.95)	0.73(0.87-0.67)	2.03(2.2-1.7)	2.1(2.35-1.62)	2.13(2.61-1.84)	5.35(6.42-4.59)	4.11(5.01-3.66)	2.24(2.68-1.98)
Leukemia	6.76(7.16-6.15)	10.54(11.71-8.81)	6.37(6.91-5.28)	6.34(7.65-4.99)	4.74(5.16-4.36)	6.37(6.72-5.95)	6.91(7.55-6.35)	5.27(5.81-4.8)	9.62(11.02-8.33)	8.86(9.35-8.36)	5.29(5.76-4.88)
Other malignant neoplasms	9.16(9.47-8.4)	13.58(14.4-11.93)	5.65(6.43-5.19)	6.01(6.83-5.19)	6.3(6.85-5.94)	10.82(11.68-9.9)	19.24(21.27-15.76)	11.17(12.44-9.49)	15(16.61-13.29)	14.45(16-12.84)	5.82(7.53-5.11)

High-income North America	Caribbean	Andean Latin America	Central Latin America	Tropical Latin America	North Africa and Middle East	South Asia	Central Sub-Saharan Africa	Eastern Sub-Saharan Africa	Southern Sub-Saharan Africa	Western Sub-Saharan Africa
3.88(4-3.77)	3.29(3.6-3.02)	1.31(1.43-1.18)	1.38(1.44-1.31)	4.65(4.76-4.53)	2.18(2.35-2.04)	3.93(4.17-3.71)	7.26(8.51-6.17)	7.8(8.57-7.2)	9.96(10.42-9.47)	3.98(4.69-3.48)
6.49(6.71-6.29)	7.94(8.55-7.41)	16.6(17.95-15.23)	12.93(13.54-12.35)	9.45(9.68-9.25)	8.66(9.08-8.26)	7.15(7.48-6.76)	7.13(7.99-6.28)	6.37(6.84-5.93)	5.19(5.44-4.97)	7.72(8.48-7.05)
6.48(6.73-6.24)	5.44(5.87-4.95)	6.81(7.46-6.22)	5.71(5.91-5.5)	4.94(5.07-4.82)	5.73(6.26-5.25)	3.32(3.57-3.05)	7.53(11.16-5.19)	7.47(8.22-6.75)	6.53(7.08-6.08)	11.1(13.14-9.61)
3.11(3.22-3)	4.64(5.13-4.16)	0.9(0.99-0.81)	1.48(1.57-1.39)	3.03(3.13-2.93)	2.33(2.47-2.19)	3.57(3.77-3.38)	1.55(1.83-1.3)	1.38(1.55-1.23)	1.84(1.95-1.74)	1.33(1.58-1.14)
44.22(45.51-42.92)	19.35(20.86-18)	9.07(9.94-8.21)	9.4(9.79-9.02)	13.64(14.01-13.31)	14.64(15.42-13.83)	9.19(9.81-8.64)	9.1(11.41-7.6)	7.21(7.9-6.68)	14.69(15.39-14.03)	8.06(9.41-6.98)
49.54(51.52-47.62)	27.85(31.29-24.8)	14.77(17.07-12.92)	21.09(22.21-20.01)	22.52(23.28-21.79)	18.06(20.28-16.9)	14.07(16.61-12.17)	13.32(17.01-10.7)	12.95(14.82-11.36)	17.44(18.82-15.75)	20.57(26.95-15.8)
5.03(5.32-4.76)	13.46(15.76-11.13)	12.85(14.79-10.87)	10.65(11.4-10.02)	9.88(10.32-9.47)	2.72(2.99-2.36)	7.37(8.62-6.76)	22.56(27.61-16.52)	17.95(21.31-15.35)	18.87(20.48-16.89)	15.71(19.29-12.27)
14.09(14.73-13.51)	9.39(10.51-8.38)	5.13(5.96-4.47)	4.64(4.92-4.37)	3.6(3.75-3.45)	2.45(2.65-2.26)	1.82(1.98-1.67)	1.7(2.23-1.35)	1.84(2.28-1.57)	2.54(2.77-2.33)	1.71(1.99-1.48)
48.19(71.33-43.17)	40.45(46.17-30.14)	19.95(25.95-16.91)	26.83(32.81-20.78)	22.3(31.42-19.65)	15.24(17.03-10.89)	4.34(5.21-3.58)	9.68(11.67-6.63)	10.9(12.44-7.99)	17.07(19.65-13.47)	23.91(30.24-16.21)
39.09(40.32-37.88)	23.48(25.33-21.84)	14.25(15.64-12.87)	15.22(15.81-14.59)	16.24(16.75-15.74)	12.37(12.94-11.78)	8.1(8.71-7.24)	9.19(10.94-7.94)	10.67(11.35-9.87)	11.11(11.84-10.14)	8.96(10.51-7.71)
5.59(5.79-5.39)	4.09(4.43-3.77)	1.96(2.18-1.76)	1.89(1.97-1.8)	3.96(4.1-3.81)	1.48(1.56-1.41)	10.71(11.5-9.92)	2.69(3.05-2.34)	2.97(3.21-2.74)	3.68(3.92-3.45)	1.85(2.1-1.62)
0.51(0.53-0.48)	0.77(0.87-0.68)	0.24(0.29-0.2)	0.42(0.45-0.39)	0.4(0.43-0.36)	0.78(0.87-0.71)	1.17(1.26-1.1)	0.6(0.73-0.49)	1.32(1.53-1.12)	0.57(0.63-0.52)	0.58(0.76-0.47)
2.51(2.62-2.4)	1.48(1.69-1.34)	0.87(1.02-0.77)	0.56(0.6-0.54)	2.17(2.28-2.06)	0.47(0.61-0.43)	5.84(6.35-4.74)	0.52(0.66-0.43)	0.85(0.97-0.66)	0.68(0.77-0.55)	0.45(0.52-0.4)
2.08(2.3-1.95)	1.18(1.68-1.02)	3.34(4.08-2.94)	2.12(2.56-2.01)	2.21(2.28-2.14)	1.36(1.67-1.18)	2.83(3.19-2.21)	1.23(1.54-1.02)	1.26(1.66-1.04)	1.11(1.26-0.9)	1.23(1.65-1.01)
9.84(10.16-9.55)	4.55(4.91-4.22)	4.23(4.61-3.84)	4.15(4.3-4)	5.23(5.39-5.1)	3.73(3.91-3.53)	2.66(2.82-2.53)	3.09(3.55-2.66)	3.01(3.33-2.67)	5.13(5.4-4.83)	4.19(4.91-3.61)
17.29(24.45-14.04)	1.41(1.94-1.18)	1.49(2.08-1.25)	1.5(1.99-1.15)	2.24(3.36-1.7)	1.17(1.94-0.94)	0.32(0.38-0.24)	0.66(0.93-0.52)	0.85(1.11-0.66)	1.59(1.85-1.1)	0.6(0.8-0.47)
839.49(1160.4-573.7)	21.98(28.55-16.43)	43.25(64.22-28.13)	117.05(179.2-69.91)	144.54(213.67-90.3)	18.58(24.25-14.09)	5.19(7.95-3.24)	27.61(41.85-16.47)	20.45(31.33-13)	101.39(157.35-56.7)	11.44(17.29-6.92)
5.03(5.32-4.73)	3.3(3.78-2.96)	3.29(3.77-2.86)	3.82(4.02-3.64)	3.32(3.48-3.18)	2.54(2.7-2.39)	3.18(3.61-2.89)	2.31(2.92-1.78)	3.47(3.94-2.93)	3.4(3.68-3.07)	2.31(2.9-1.83)
3.19(3.47-2.94)	0.54(0.66-0.46)	0.99(1.21-0.81)	2.36(2.65-2.16)	1.07(1.21-0.97)	0.7(0.83-0.6)	0.29(0.33-0.26)	0.15(0.19-0.1)	0.08(0.09-0.07)	0.24(0.27-0.22)	0.07(0.1-0.06)
12.15(13.19-11.55)	4.67(5.67-4.13)	5.05(5.64-4.39)	5.68(6.02-5.4)	5.04(5.26-4.79)	3.09(3.31-2.68)	1.87(1.97-1.7)	2.66(3.27-2.19)	2.47(2.87-2.11)	3.28(3.63-2.93)	2.92(3.41-2.52)
10.05(10.38-9.76)	5.49(6.01-5.07)	2.54(2.88-2.26)	2.79(2.92-2.67)	4.42(4.56-4.29)	7.82(9.27-7.16)	2.53(2.87-2.35)	3.79(5.19-2.99)	3.32(3.71-2.87)	4.51(4.85-3.93)	3.26(3.76-2.74)
7.58(8.57-6.95)	3.32(4.2-1.68)	3.16(4.16-1.68)	2.77(2.99-2.22)	5.18(5.59-3.94)	4.96(5.8-4.17)	2.69(3.15-2.25)	1.7(2.11-1.44)	2.4(2.86-1.88)	1.9(2.2-1.35)	1.78(2.21-1.31)
5.44(5.65-5.22)	2.86(3.16-2.58)	4.12(4.7-3.56)	3.44(3.65-3.27)	2.28(2.38-2.17)	3.19(3.65-2.93)	2.29(2.58-2.03)	0.74(1.04-0.59)	1.84(2.15-1.59)	1.16(1.32-1.05)	0.55(0.66-0.47)
0.62(0.64-0.59)	0.22(0.26-0.19)	0.27(0.3-0.24)	0.3(0.32-0.29)	0.46(0.49-0.43)	0.47(0.52-0.41)	0.25(0.31-0.21)	0.2(0.28-0.15)	0.16(0.21-0.11)	0.71(0.8-0.65)	0.18(0.21-0.15)
3.23(4.84-2.69)	1.16(1.45-0.61)	0.55(0.67-0.46)	0.88(1.1-0.75)	0.65(0.79-0.54)	1.45(1.65-1.03)	0.68(0.87-0.57)	0.54(0.75-0.42)	1.04(1.41-0.77)	0.4(0.46-0.28)	1.24(1.69-0.91)
15.24(15.75-14.77)	5.35(5.83-4.96)	5.09(5.56-4.62)	3.82(4.03-3.66)	3.77(3.89-3.65)	4.34(4.63-4.12)	2.96(3.13-2.76)	2.16(2.71-1.57)	5.96(6.67-5.1)	3.25(3.47-3.01)	3.56(4.13-3.11)
4.72(6.13-4.38)	2.6(3.06-2.35)	1.67(2.11-1.45)	1.55(1.88-1.4)	1.73(2.06-1.48)	1.45(1.84-1.32)	1.03(1.13-0.91)	1.09(1.37-0.81)	1.57(1.72-1.3)	1.94(2.11-1.51)	1.4(1.68-1.16)
7.17(7.57-6.89)	5.82(6.71-5.25)	5.97(6.77-4.93)	5.89(6.24-5.63)	4.65(4.86-4.48)	6.37(7.19-5.63)	3.99(4.47-3.46)	3.87(4.6-2.9)	4.48(5.21-3.52)	4.1(4.44-3.35)	3.4(4.01-2.76)
11.19(12.24-10.51)	6.54(8.04-5.75)	4.79(6.07-4.15)	4.69(5.03-4.27)	5.66(6.48-5.38)	5.33(6-4.99)	4.81(5.16-4.18)	5.27(7.38-3.81)	9.48(10.72-7.76)	4.86(5.25-4.28)	5.91(6.93-5.04)
