# Supplementary material for: Incidence and death in 29 cancer groups in 2017 and trend analysis from 1990 to 2017 from the Global Burden of Disease Study
Source: J Hematol Oncol. 2019 Sep 12;12:96. doi: 10.1186/s13045-019-0783-9 (PMC6740016; doi:10.1186/s13045-019-0783-9)
Supplement: Supplementary file 4 — Death number of 29 specified cancer groups for 21 regions in 2017. (PDF 70 kb) [file 13045_2019_783_MOESM4_ESM.pdf]

**Death number for 21 regions in 2017**

| Tumor types | Global | Southeast Asia | Southeast Asia | Oceania | Central Asia | Central Europe | Eastern Europe | High-income Asia Pacific | Australasia |
|---|---|---|---|---|---|---|---|---|---|
| Esophageal cancer | 435959(447580-424994) | 221066(231096-210944) | 14818(15905-13909) | 140(165-119) | 4219(4423-4012) | 5645(5865-5431) | 10032(10333-9787) | 14765(15353-14124) | 1855(2032-1682) |
| Stomach cancer | 864989(884655-848254) | 371288(387201-356398) | 38871(41869-36283) | 913(1077-758) | 10331(10769-9891) | 18570(19135-18014) | 43943(45173-42870) | 68042(71099-65688) | 2233(2429-2052) |
| Liver cancer | 819435(855505-789721) | 443133(472122-418580) | 70453(75990-64453) | 689(908-444) | 6699(7039-6363) | 11225(11624-10834) | 13438(14196-12834) | 46032(49194-41218) | 2060(2248-1870) |
| Larynx cancer | 126471(129853-123375) | 20430(21360-19560) | 8008(9782-7311) | 120(140-103) | 1236(1305-1168) | 5013(5215-4812) | 6876(7091-6672) | 1694(1769-1623) | 293(329-262) |
| Tracheal, bronchus, and lung cancer | 1883066(1922809-1844246) | 722056(751266-690734) | 123245(135238-112824) | 1516(2043-1257) | 11871(12397-11374) | 73025(75251-70732) | 73239(74771-71709) | 100625(103722-97463) | 10957(11886-10041) |
| Breast cancer | 611625(640680-589197) | 93555(100095-78460) | 50881(55081-47153) | 844(1148-650) | 6164(6543-5803) | 20536(21373-19735) | 32586(33542-31689) | 18580(19405-17800) | 4131(4613-3666) |
| Cervical cancer | 259671(269214-241128) | 48100(51945-32269) | 25675(28766-21854) | 815(1041-616) | 2848(3054-2662) | 6902(7258-6578) | 8344(8647-8075) | 5304(5573-5094) | 553(632-486) |
| Uterine cancer | 85239(87446-83186) | 13018(13958-12214) | 6487(7254-5765) | 224(276-182) | 1081(1150-1013) | 3939(4126-3760) | 7245(7508-7005) | 3528(3705-3362) | 630(715-555) |
| Prostate cancer | 415910(489540-357283) | 55114(68690-46129) | 22668(26091-18370) | 337(395-263) | 2416(2651-1743) | 15866(17863-12154) | 16037(17174-10198) | 17239(20825-12344) | 4455(5825-3617) |
| Colon and rectum cancer | 896040(915720-876279) | 199982(208686-189925) | 55681(59309-52786) | 546(760-457) | 6105(6372-5822) | 45941(47394-44622) | 57216(58856-55845) | 68204(71047-65810) | 7614(8224-7036) |
| Lip and oral cavity cancer | 193696(201641-184723) | 23647(24772-22499) | 13523(14356-12743) | 174(242-142) | 1298(1364-1230) | 5389(5595-5189) | 8391(8719-8116) | 5133(5384-4952) | 798(879-724) |
| Nasopharynx cancer | 69550(72270-66872) | 29636(31591-27970) | 10741(11998-9736) | 163(203-130) | 291(312-273) | 721(753-689) | 984(1032-940) | 1360(1449-1283) | 250(278-223) |
| Other pharynx cancer | 117412(124476-102082) | 6265(6954-5538) | 5861(6977-5209) | 93(119-72) | 620(665-576) | 3819(4009-3597) | 5240(5479-5038) | 2920(3129-2772) | 422(469-380) |
| Gallbladder and biliary tract cancer | 173974(184875-154232) | 29742(32501-22670) | 11109(12134-8552) | 80(96-61) | 786(833-721) | 6709(7152-6421) | 4672(4888-4465) | 27063(28309-25357) | 845(936-764) |
| Pancreatic cancer | 441083(448960-432833) | 89755(93552-85883) | 19909(21437-18283) | 206(252-181) | 3463(3617-3315) | 19596(20168-19036) | 25356(25886-24870) | 40359(41673-39113) | 3618(3918-3329) |
| Malignant skin melanoma | 61665(70323-47910) | 5448(6217-3814) | 1580(2061-1394) | 49(77-34) | 454(649-393) | 3821(4226-2811) | 5873(7057-4251) | 995(1198-714) | 2110(2470-1311) |
| Non-melanoma skin cancer | 65097(66459-63091) | 16709(17458-15848) | 4487(4849-4164) | 64(76-54) | 537(563-511) | 2683(2774-2593) | 3225(3353-3109) | 1889(1988-1803) | 910(995-837) |
| Ovarian cancer | 175982(181198-171384) | 26531(28056-25040) | 13559(15639-12026) | 141(181-112) | 1652(1747-1566) | 7782(8178-7426) | 11274(11716-10849) | 6554(6876-6233) | 1263(1437-1114) |
| Testicular cancer | 7662(8013-7369) | 694(747-641) | 322(358-285) | 31(39-24) | 131(143-121) | 371(392-353) | 414(436-392) | 100(107-92) | 32(36-28) |
| Kidney cancer | 138526(142522-128656) | 18634(19987-16488) | 5975(6474-5097) | 59(74-46) | 1704(1791-1611) | 8099(8503-7044) | 12953(13374-12178) | 8974(9523-7762) | 1588(1739-1436) |
| Bladder cancer | 196546(205835-191549) | 32750(37532-31029) | 10133(11301-8544) | 102(116-83) | 1446(1532-1373) | 10120(10483-9762) | 10528(10835-10202) | 11147(11566-10756) | 1505(1651-1373) |
| Brain and nervous system cancer | 247143(265001-212969) | 63261(72156-52664) | 17437(19683-13243) | 175(243-115) | 3212(3564-2431) | 9402(10073-7406) | 10265(11271-9017) | 4514(5010-2852) | 1741(1969-1354) |
| Thyroid cancer | 41235(44139-39911) | 7248(7919-6827) | 4821(5704-4458) | 50(62-41) | 260(275-246) | 919(961-880) | 1665(1730-1612) | 2774(2935-2651) | 192(212-173) |
| Mesothelioma | 29909(30613-29134) | 2928(3102-2709) | 1338(1448-1244) | 23(35-17) | 143(150-136) | 624(664-588) | 884(925-847) | 1702(1831-1614) | 901(1000-806) |
| Hodgkin lymphoma | 32560(38086-27644) | 2900(3298-2003) | 2186(2639-1787) | 42(53-30) | 370(437-316) | 783(936-681) | 1478(1853-1248) | 298(333-207) | 101(117-79) |
| Non-Hodgkin lymphoma | 248636(253064-243475) | 43353(45322-41529) | 16459(17455-15377) | 215(303-180) | 1484(1559-1409) | 5806(5985-5633) | 8200(8378-8008) | 14795(15379-14191) | 2246(2434-2056) |
| Multiple myeloma | 107114(118911-98521) | 13049(13940-11419) | 4050(4797-3732) | 68(103-55) | 471(558-431) | 3536(3799-2901) | 4894(5226-3727) | 6266(7294-5497) | 1326(1528-1127) |
| Leukemia | 347583(364877-317256) | 63510(68517-53813) | 31823(34581-26195) | 491(614-385) | 2950(3134-2770) | 9758(10099-9205) | 13492(13923-13062) | 12211(12672-11728) | 2649(2889-2414) |
| Other malignant neoplasms | 359548(370791-331393) | 67676(72001-58561) | 26985(31032-24863) | 450(528-380) | 4093(4425-3890) | 9552(9991-8789) | 27516(29510-19946) | 12372(13131-10252) | 1678(1851-1451) |

| Western Europe | Southern Latin America | High-income North America | Caribbean | Andean Latin America | Central Latin America | Tropical Latin America | North Africa and Middle East | South Asia | Central Sub-Saharan Africa |
|---|---|---|---|---|---|---|---|---|---|
| 30228(31378-29195) | 3584(3919-3328) | 21211(21780-20594) | 1736(1900-1598) | 760(834-687) | 3358(3497-3205) | 11256(11508-10993) | 9193(9938-8552) | 53633(56781-50604) | 3670(4279-3113) |
| 62213(64525-59851) | 10203(10988-9515) | 22159(22750-21591) | 3684(3966-3438) | 9130(9901-8408) | 21226(22007-20432) | 21140(21550-20734) | 34530(36201-32838) | 96652(101052-91276) | 3551(3990-3110) |
| 47386(49047-45781) | 3726(4052-3428) | 30076(30960-29164) | 2907(3134-2657) | 3906(4271-3569) | 13976(14476-13460) | 12027(12355-11718) | 24703(27033-22582) | 45851(49414-42145) | 3877(5762-2733) |
| 9258(9660-8888) | 1210(1348-1092) | 5655(5825-5481) | 1541(1686-1399) | 398(440-360) | 2530(2665-2375) | 5083(5226-4954) | 6771(7139-6343) | 44258(46791-41776) | 770(915-631) |
| 241323(250115-232785) | 16476(17837-15273) | 212191(217684-207062) | 10216(10973-9486) | 5198(5688-4715) | 22611(23534-21734) | 33172(34016-32398) | 60971(64094-57608) | 126519(134597-118809) | 4573(5802-3778) |
| 88977(93164-84917) | 9041(10150-8131) | 55418(57196-53745) | 5149(5853-4528) | 3260(3732-2881) | 15682(16453-14949) | 19216(19702-18734) | 27709(31151-25885) | 108966(131457-93488) | 5083(6576-3970) |
| 11762(12368-11212) | 4306(4861-3826) | 8431(8777-8098) | 3048(3494-2577) | 3466(3961-2997) | 11363(11976-10797) | 10200(10578-9861) | 5808(6368-5054) | 52921(63949-48233) | 7591(9381-5637) |
| 11657(12265-11091) | 1265(1424-1129) | 9116(9449-8795) | 1234(1373-1114) | 918(1044-809) | 2608(2766-2463) | 2602(2685-2518) | 2597(2773-2426) | 12210(13440-11146) | 539(711-426) |
| 78373(103800-64317) | 8016(10196-6334) | 41463(61970-37818) | 8842(9809-6574) | 4965(6396-4327) | 16904(20659-13165) | 20102(27783-17376) | 15289(16726-10863) | 38058(45834-31411) | 3090(3714-2109) |
| 144456(149986-138821) | 13402(14491-12431) | 89893(92289-87480) | 6735(7276-6263) | 4352(4733-3952) | 18272(18925-17589) | 23374(24019-22683) | 31342(32855-29922) | 86712(93391-77540) | 3885(4744-3296) |
| 13050(13613-12521) | 1049(1153-957) | 7982(8234-7740) | 1170(1260-1084) | 578(635-523) | 2309(2406-2217) | 5322(5496-5134) | 3234(3394-3092) | 92468(99403-84929) | 1018(1155-882) |
| 3932(4158-3737) | 145(160-133) | 1251(1295-1214) | 288(310-263) | 92(102-83) | 687(717-658) | 544(566-523) | 2375(2544-2216) | 12692(13310-12081) | 292(341-241) |
| 9193(9757-8689) | 353(389-319) | 4106(4267-3946) | 532(602-479) | 357(418-316) | 932(984-884) | 3628(3809-3455) | 1402(1796-1283) | 69025(75214-55435) | 258(326-212) |
| 19550(20707-18513) | 5779(6370-5350) | 6016(6571-5805) | 621(881-541) | 1891(2329-1666) | 5068(6052-4798) | 5308(5466-5131) | 5486(6700-4758) | 38087(42667-29570) | 566(705-467) |
| 81449(84439-78708) | 7185(7754-6658) | 54590(56194-53258) | 2467(2654-2296) | 2437(2655-2212) | 10118(10467-9742) | 12830(13198-12509) | 15636(16414-14791) | 35820(37929-34115) | 1496(1700-1295) |
| 15895(17445-10142) | 905(1107-636) | 11830(14846-8605) | 284(387-237) | 442(595-372) | 1492(1946-1159) | 2311(3410-1720) | 1904(2987-1572) | 3071(3651-2266) | 310(443-242) |
| 7545(7844-7252) | 934(1011-866) | 4547(4722-4398) | 717(772-668) | 533(576-493) | 2744(2860-2629) | 2863(2950-2768) | 1624(1736-1546) | 8753(9819-8076) | 491(564-415) |
| 28329(29811-26799) | 2025(2286-1806) | 18619(19464-17793) | 1039(1166-942) | 1075(1220-943) | 5199(5455-4943) | 4780(4997-4596) | 6810(7227-6418) | 29549(32610-27074) | 936(1181-720) |
| 619(663-575) | 317(359-278) | 478(507-448) | 36(40-32) | 123(142-107) | 786(824-748) | 362(381-342) | 451(510-406) | 1983(2239-1767) | 78(100-55) |
| 30325(31837-27097) | 3495(3857-3186) | 19048(20091-18298) | 762(896-696) | 982(1093-844) | 4542(4801-4308) | 4029(4211-3809) | 4497(4820-3911) | 8316(8799-7511) | 494(595-414) |
| 43838(45622-42016) | 2510(2723-2316) | 21937(22678-21349) | 1331(1443-1239) | 738(844-664) | 3000(3130-2880) | 4950(5105-4812) | 12580(15208-11562) | 18870(21249-17526) | 1077(1447-864) |
| 29135(31176-21904) | 2283(2569-2017) | 19994(21743-17280) | 1274(1597-613) | 1532(1998-811) | 5419(5828-4232) | 10211(10960-7237) | 17741(20620-14777) | 36657(42760-30612) | 1275(1662-1005) |
| 3861(4046-3685) | 429(468-396) | 2383(2453-2299) | 268(293-247) | 528(582-459) | 1565(1639-1499) | 1131(1165-1088) | 1917(2283-1799) | 8932(9841-8181) | 186(248-147) |
| 10303(10771-9862) | 300(330-274) | 3788(3945-3639) | 86(100-76) | 121(133-109) | 577(602-552) | 864(905-808) | 1669(1848-1480) | 2756(3482-2337) | 85(119-64) |
| 2441(3057-2013) | 322(434-277) | 1512(2045-1371) | 257(318-139) | 201(231-163) | 1074(1315-899) | 747(919-609) | 3196(3641-2369) | 8536(10987-7260) | 361(489-288) |
| 34809(36047-33501) | 2906(3162-2698) | 29276(30041-28523) | 1725(1887-1605) | 2203(2394-1998) | 6255(6537-5996) | 6278(6449-6097) | 12508(13324-11851) | 37348(39553-34825) | 1273(1542-975) |
| 22678(26512-19544) | 1504(1788-1327) | 16701(20949-15272) | 1096(1290-994) | 804(1015-704) | 3001(3618-2727) | 3475(4111-2964) | 4852(6179-4431) | 13054(14347-11429) | 522(654-391) |
| 48524(50340-46559) | 3614(3917-3338) | 31902(32959-30999) | 2451(2755-2250) | 2826(3140-2375) | 11484(12054-11000) | 8908(9158-8683) | 25096(28531-22072) | 52412(58699-45681) | 2693(3222-1956) |
| 35115(37136-29940) | 3784(4817-3329) | 19943(21680-18813) | 2573(3117-2296) | 2468(3092-2155) | 9181(9810-8281) | 11276(13062-10551) | 18758(21394-17482) | 64960(69881-56367) | 3289(4879-2579) |

| Eastern Sub-Saharan Africa | Southern Sub-Saharan Africa | Western Sub-Saharan Africa |
|---|---|---|
| 12100(13251-11150) | 5639(5903-5349) | 7050(8363-6148) |
| 10087(10828-9391) | 2910(3052-2779) | 13311(14701-12153) |
| 12356(13629-11190) | 3768(4062-3505) | 21149(24999-18311) |
| 2155(2427-1928) | 911(965-861) | 2260(2678-1935) |
| 10942(12057-10145) | 8367(8759-7982) | 13974(16392-12100) |
| 15068(17331-13192) | 5469(5883-4944) | 25311(32925-19602) |
| 18656(22249-15779) | 5876(6406-5243) | 17702(21539-14000) |
| 1831(2267-1560) | 700(767-638) | 1811(2089-1566) |
| 12058(13743-8745) | 5932(6684-4577) | 28683(36014-19399) |
| 14245(15152-13187) | 4824(5138-4423) | 13259(15582-11407) |
| 3472(3772-3182) | 1360(1453-1272) | 2329(2651-2039) |
| 1891(2166-1609) | 267(284-249) | 949(1162-795) |
| 1301(1492-1008) | 324(364-265) | 763(874-675) |
| 1894(2450-1568) | 612(689-497) | 2090(2810-1715) |
| 4638(5153-4104) | 2871(3022-2704) | 7283(8582-6271) |
| 1295(1684-990) | 642(745-458) | 955(1264-754) |
| 1812(2092-1269) | 1171(1252-1002) | 859(1027-649) |
| 4297(4880-3625) | 1348(1458-1220) | 3218(4011-2567) |
| 126(147-111) | 79(86-72) | 130(172-106) |
| 1467(1696-1259) | 627(685-559) | 1956(2265-1696) |
| 3171(3561-2726) | 1357(1466-1199) | 3457(3974-2906) |
| 5758(6977-4494) | 1096(1269-784) | 4761(5943-3423) |
| 1412(1635-1213) | 217(240-200) | 478(554-411) |
| 215(283-142) | 337(376-306) | 266(317-220) |
| 2459(3392-1800) | 213(244-152) | 3083(4161-2193) |
| 11656(13234-9877) | 1714(1836-1585) | 8125(9336-7181) |
| 2360(2583-1955) | 1008(1095-782) | 2401(2889-1993) |
| 10133(12012-7899) | 2298(2490-1892) | 8355(9864-6749) |
| 19934(22688-16138) | 2773(3007-2438) | 15173(17844-12757) |
