# Supplementary material for: Incidence and death in 29 cancer groups in 2017 and trend analysis from 1990 to 2017 from the Global Burden of Disease Study
Source: J Hematol Oncol. 2019 Sep 12;12:96. doi: 10.1186/s13045-019-0783-9 (PMC6740016; doi:10.1186/s13045-019-0783-9)
Supplement: Supplementary file 5 — Incidence number of 29 specified cancer groups for 21 regions in 2017. (PDF 71 kb) [file 13045_2019_783_MOESM5_ESM.pdf]

Incidence number for 21 regions in 2017

Tumor types	Global	Southeast Asia	Southeast Asia	Oceania	Central Asia	Central Europe	Eastern Europe	High-income Asia Pacific
Esophageal cancer	472525(485294-459485)	244509(256226-232910)	14755(15840-13824)	145(171-123)	4115(4321-3915)	5462(5679-5250)	11804(12183-11452)	22145(23212-20956)
Stomach cancer	1220662(1254563-1189032)	583758(612688-554933)	39191(42265-36559)	988(1177-815)	10513(10965-10059)	19794(20462-19194)	59809(61663-57983)	131636(138437-125691)
Liver cancer	953076(997047-916543)	545623(584189-513237)	70240(75833-64069)	735(971-473)	6639(6990-6306)	10627(11024-10257)	13062(13827-12457)	64061(69767-57200)
Larynx cancer	210606(215539-206424)	41890(44004-39973)	11458(13564-10463)	141(166-122)	1744(1845-1640)	8693(9074-8318)	12272(12760-11815)	6861(7260-6472)
Tracheal, bronchus, and lung cancer	2163132(2212890-2117035)	845753(883106-809404)	119498(130961-109301)	1549(2089-1274)	11832(12373-11334)	74369(76602-71946)	90382(93561-87330)	135164(140269-129852)
Breast cancer	1960682(2023170-1891447)	386508(417015-326128)	125591(135934-115233)	1715(2379-1274)	16634(17814-15527)	60364(63445-57627)	97209(100843-93529)	91895(97839-85833)
Cervical cancer	601186(625402-554455)	113036(122613-73551)	62047(69261-52363)	2325(3172-1583)	7101(7760-6527)	13320(14105-12602)	21930(23132-20770)	15490(16613-14526)
Uterine cancer	406793(418006-396709)	71683(77456-66853)	19490(21498-17328)	521(652-413)	4743(5124-4378)	21171(22395-19990)	30433(32241-28746)	14836(16040-13803)
Prostate cancer	1334315(1697900-1170862)	155879(198274-134186)	40035(47700-33326)	482(565-371)	4510(4983-3187)	39634(44403-29716)	56976(64119-35077)	72562(92261-54490)
Colon and rectum cancer	1833451(1873464-1791865)	462088(483591-438223)	85149(90557-80680)	745(1031-617)	8977(9410-8558)	72984(75162-70812)	103116(106623-100177)	183789(193063-175950)
Lip and oral cavity cancer	389760(404404-374456)	55187(57791-52417)	27650(29504-25860)	328(451-267)	2364(2505-2223)	9241(9641-8866)	15878(16610-15254)	14504(15635-13563)
Nasopharynx cancer	109781(115555-104443)	49492(54427-45351)	16913(19065-15126)	279(376-201)	452(552-383)	863(946-803)	1618(1796-1445)	1525(1664-1403)
Other pharynx cancer	179326(188621-160343)	12773(14103-11256)	8282(9754-7349)	120(157-92)	927(994-863)	6525(6833-6143)	9481(9913-9089)	6135(6645-5746)
Gallbladder and biliary tract cancer	210878(225429-186150)	34677(38062-25715)	11569(12831-8848)	82(100-63)	756(801-690)	6834(7283-6449)	5140(5569-4815)	43936(53057-35907)
Pancreatic cancer	447665(456296-438598)	88159(91988-84111)	19111(20603-17530)	209(254-182)	3342(3493-3200)	18689(19249-18148)	25865(26509-25301)	46240(48931-43540)
Malignant skin melanoma	308684(365866-237650)	16768(19608-11279)	2515(3316-2214)	59(95-40)	1076(1565-940)	14459(16378-10837)	20492(25680-15045)	6302(7819-4534)
Non-melanoma skin cancer	7663589(10570278-5251106)	260981(351789-190967)	62813(95211-41949)	997(1407-725)	44728(67278-26202)	126618(190062-79475)	211209(334281-118485)	37208(58697-21844)
Ovarian cancer	286127(295311-278075)	43755(46253-41183)	27407(31860-24128)	314(429-235)	2829(3008-2652)	10865(11392-10351)	17393(18246-16516)	12100(12798-11337)
Testicular cancer	71348(74442-68780)	6217(6748-5711)	1822(2109-1566)	71(91-53)	608(673-550)	4791(5243-4387)	2673(2976-2392)	2807(3204-2423)
Kidney cancer	393043(404595-371162)	52291(56228-46830)	20831(22722-17706)	266(354-205)	5185(5520-4844)	17167(18046-14768)	32267(33740-30307)	16893(18246-14907)
Bladder cancer	473800(491763-462151)	78486(89371-74207)	21375(23597-18310)	218(253-181)	3279(3449-3105)	23446(24342-22589)	25657(26586-24652)	29913(31392-28449)
Brain and nervous system cancer	405218(442624-351030)	125571(150246-102730)	21221(24008-16237)	223(319-144)	4288(4796-3220)	12309(13246-9927)	16679(19922-15090)	15010(17146-9703)
Thyroid cancer	255489(272471-245709)	44769(50710-41549)	23883(29124-21430)	185(235-145)	1744(1903-1607)	6503(6948-6106)	14495(15530-13608)	20618(22712-18892)
Mesothelioma	34615(35697-33530)	3055(3233-2830)	1785(1967-1623)	32(51-23)	206(221-193)	827(898-766)	1233(1340-1148)	1698(1844-1594)
Hodgkin lymphoma	101133(118746-87968)	19018(21638-13550)	3496(4318-2901)	56(70-39)	981(1144-815)	3302(4017-2914)	8495(10726-7410)	1912(2199-1333)
Non-Hodgkin lymphoma	487964(496904-478850)	88039(92084-83583)	21304(22665-19922)	267(380-225)	2500(2638-2359)	12625(13081-12183)	18131(19029-17217)	33829(36179-31576)
Multiple myeloma	152746(172662-140564)	20545(22148-17475)	4748(5610-4380)	77(118-62)	569(677-519)	4242(4580-3547)	7138(7956-5479)	9551(11547-8251)
Leukemia	518485(548018-472240)	147726(161336-124458)	38894(42316-32374)	623(802-483)	4027(4408-3676)	11075(11569-10405)	19717(21322-18142)	17764(19509-16084)
Other malignant neoplasms	715547(740043-656333)	237880(253350-206823)	34636(39341-31741)	545(651-455)	5109(5592-4795)	17227(18461-15626)	49258(54450-39632)	34531(38063-28824)

Australasia	Western Europe	Southern Latin America	High-income North America	Caribbean	Andean Latin America	Central Latin America	Tropical Latin America	North Africa and Middle East
2165(2382-1961)	34183(35615-32753)	3315(3632-3074)	23420(24124-22732)	1684(1844-1546)	699(767-631)	3162(3297-3017)	10981(11257-10709)	9080(9833-8456)
4263(4692-3845)	95266(100111-90462)	10145(10956-9436)	39247(40539-37998)	4033(4342-3769)	8925(9658-8194)	29601(31008-28255)	21823(22331-21369)	35755(37539-33988)
2387(2679-2121)	55100(57629-52731)	3431(3727-3156)	38190(39686-36882)	2761(2979-2514)	3668(4024-3351)	13113(13578-12619)	11500(11799-11198)	24644(26906-22354)
953(1087-836)	25275(26635-24062)	1881(2102-1688)	18625(19280-17949)	2377(2626-2131)	483(531-437)	3435(3637-3213)	7247(7487-7023)	10059(10634-9448)
15325(16737-13946)	292856(304685-280870)	15637(16925-14469)	273198(280843-265265)	9857(10623-9160)	4859(5330-4400)	21527(22432-20669)	31657(32499-30913)	60442(63764-57175)
18761(21387-16363)	341848(358885-324867)	22659(25512-20245)	276898(287822-266909)	14186(15935-12637)	8293(9587-7251)	51422(54158-48742)	54359(56239-52537)	91173(100561-85206)
1152(1344-984)	26183(27712-24707)	10926(12650-9423)	23037(24240-21875)	6705(7824-5560)	7347(8479-6216)	26756(28660-25148)	24126(25222-23143)	14577(16180-12548)
3441(3968-2955)	80410(85307-75826)	4223(4765-3743)	82183(85883-78827)	4808(5395-4295)	2836(3303-2470)	11336(12026-10661)	8594(8960-8254)	11456(12406-10582)
24244(31635-18374)	312252(439750-262883)	16776(21636-13149)	300861(446835-269548)	20591(23495-15325)	10505(13669-8905)	60663(74224-47022)	50673(71397-44684)	57631(64375-41497)
22266(24232-20408)	347288(361454-332898)	20898(22657-19394)	234927(241844-228060)	11943(12868-11109)	7635(8372-6901)	35294(36661-33818)	37656(38850-36473)	52224(54659-49748)
2652(2985-2363)	38705(40634-36917)	2186(2412-1984)	32585(33705-31474)	2084(2257-1923)	1082(1203-972)	4443(4650-4239)	9397(9740-9038)	6704(7050-6368)
314(370-266)	5692(6206-5278)	207(241-176)	2479(2599-2365)	385(436-340)	139(167-114)	1020(1120-943)	948(1034-867)	4101(4617-3655)
1002(1127-885)	22762(24192-21496)	555(614-501)	14417(15057-13796)	760(865-687)	478(559-421)	1346(1423-1277)	5254(5521-4986)	2167(2721-1983)
1213(1571-993)	28274(31515-25549)	5762(6400-5303)	13031(14482-12202)	600(856-521)	1785(2182-1569)	4872(5852-4610)	5084(5230-4908)	5461(6549-4724)
4129(4521-3748)	82839(86522-79435)	6613(7157-6140)	61111(63147-59322)	2317(2501-2150)	2263(2466-2051)	9543(9873-9196)	12047(12405-11750)	15214(16007-14377)
19967(24122-13498)	109014(120940-72958)	2454(3055-1804)	90398(121618-70626)	714(976-593)	834(1173-696)	3642(4848-2796)	5333(8033-4044)	5530(9066-4462)
213705(304261-139206)	714328(1002483-493434)	19535(21315-17947)	5023455(7011079-3401638)	11151(14478-8375)	23507(34571-15456)	275571(418756-167648)	336024(494366-211225)	80098(105474-61115)
1888(2160-1653)	40412(42586-38326)	3096(3508-2753)	27677(29115-26203)	1669(1910-1498)	1880(2160-1637)	9504(9993-9021)	7947(8323-7602)	12775(13627-12013)
976(1190-783)	16335(17912-14799)	3423(4090-2846)	11262(12170-10387)	264(319-221)	619(759-502)	6332(7105-5794)	2533(2857-2289)	4498(5339-3824)
3873(4320-3494)	72675(76756-65478)	9096(10168-8140)	68843(74202-65664)	2310(2813-2052)	2830(3162-2458)	13764(14577-13075)	11698(12216-11105)	15083(16227-12976)
4122(4556-3718)	119675(125309-114239)	5516(6025-5064)	62548(64613-60778)	2797(3063-2584)	1361(1542-1214)	6410(6717-6136)	10107(10427-9813)	32895(37628-29965)
2629(3046-2108)	62542(68161-49280)	2669(3070-2397)	34051(37897-30349)	1598(2002-799)	1839(2425-986)	6793(7318-5481)	11746(12699-8771)	26521(31230-22420)
2025(2313-1768)	30262(32025-28579)	2313(2595-2085)	28283(29353-27186)	1445(1598-1302)	2348(2681-2021)	8516(9034-8088)	5537(5768-5266)	17467(19886-15986)
1065(1243-914)	11729(12369-11138)	346(390-310)	3815(3966-3664)	112(134-96)	148(166-132)	723(761-686)	1091(1160-1015)	2094(2390-1809)
983(1179-785)	16797(22728-14900)	681(951-567)	12445(18154-10569)	567(707-296)	323(401-271)	2226(2779-1882)	1510(1839-1248)	8481(9789-5975)
6923(7643-6234)	96872(100951-92637)	4261(4636-3955)	86797(89558-84016)	2664(2890-2469)	2851(3111-2580)	9218(9723-8814)	8718(8999-8441)	21134(22636-20031)
2559(3039-2181)	36301(43535-31810)	1822(2168-1608)	28638(36732-26591)	1326(1562-1200)	903(1147-785)	3658(4444-3316)	4044(4844-3463)	6248(7813-5665)
4208(4871-3602)	75694(80241-71159)	3932(4260-3626)	39552(41478-38013)	2797(3200-2538)	3531(4023-2892)	14489(15395-13845)	10170(10602-9861)	32334(36500-28685)
5853(6482-5110)	96683(103707-83738)	4414(5671-3876)	49598(53731-46604)	3196(3923-2817)	2709(3450-2351)	11300(12102-10286)	12720(14626-12073)	26612(29545-24959)

South Asia	Central Sub-Saharan Africa	Eastern Sub-Saharan Africa	Southern Sub-Saharan Africa	Western Sub-Saharan Africa
53022(56177-50092)	3653(4260-3100)	11842(13029-10911)	5484(5746-5205)	6901(8208-6007)
96577(101062-91436)	3555(4009-3106)	10056(10804-9361)	2839(2979-2706)	12890(14193-11719)
45336(48744-41617)	3901(5792-2773)	12432(13728-11264)	3825(4151-3544)	21798(25965-18826)
50557(53439-47883)	820(977-666)	2334(2602-2092)	1060(1125-1000)	2441(2922-2069)
122603(130725-115074)	4525(5748-3756)	10488(11521-9701)	8169(8572-7796)	13442(15798-11616)
210811(248931-182317)	8148(10695-6236)	25615(29467-22320)	10482(11381-9508)	46110(60972-35073)
117544(136180-107899)	16071(20128-11496)	39062(46432-33250)	12498(13588-11079)	39955(49933-31011)
25923(28312-23879)	915(1176-722)	3091(3828-2638)	1415(1547-1298)	3284(3858-2824)
49583(59427-41201)	3571(4273-2453)	14105(16061-10287)	8554(9922-6787)	34228(43476-23085)
104958(113041-93845)	4416(5434-3711)	16007(17000-14839)	6002(6404-5469)	15089(17883-12862)
152348(164393-140437)	1452(1640-1258)	5169(5580-4762)	2141(2287-2005)	3659(4189-3191)
18140(19684-16788)	432(551-332)	2893(3412-2446)	363(404-328)	1526(2071-1189)
83240(90658-67903)	296(373-245)	1517(1740-1183)	408(458-331)	883(1018-780)
36859(41294-28578)	548(680-451)	1826(2337-1511)	578(646-466)	1992(2688-1634)
34252(36377-32651)	1468(1662-1268)	4495(4996-3976)	2740(2892-2582)	7021(8278-6035)
4710(5541-3413)	388(551-300)	1743(2275-1329)	933(1101-644)	1354(1814-1036)
77115(119919-46938)	17739(26562-10767)	43066(68412-25503)	57018(86979-34006)	26721(39371-16358)
48752(56104-43896)	1441(1832-1106)	7024(8030-5868)	2097(2274-1892)	5302(6693-4179)
5318(6023-4710)	145(187-99)	237(276-207)	170(190-152)	250(331-200)
27980(29678-25445)	2186(2726-1756)	6912(8386-5656)	2057(2285-1832)	8838(10332-7399)
31700(35700-29473)	1658(2193-1332)	4766(5336-4111)	2444(2640-2127)	5428(6267-4570)
42878(50522-36064)	1639(2224-1203)	7477(9116-5842)	1254(1468-880)	6279(7768-4492)
37969(43030-33683)	479(679-384)	4585(5463-3906)	758(862-679)	1304(1563-1108)
3474(4452-2926)	116(170-86)	287(393-191)	400(451-361)	378(457-310)
11510(14709-9677)	439(588-351)	3212(4447-2346)	281(326-196)	4419(6015-3078)
43547(46287-40473)	1480(1823-1166)	14129(16260-11889)	1965(2106-1816)	10709(12457-9271)
13844(15245-12137)	540(678-408)	2409(2636-1999)	1089(1184-846)	2495(3033-2062)
62007(69680-53796)	3402(4400-2400)	13301(16009-10190)	2491(2709-2014)	10753(12661-8701)
71210(76872-61808)	3975(6290-3081)	24601(28125-19699)	3154(3428-2747)	20335(24494-16714)
