# Supplementary material for: Incidence and death in 29 cancer groups in 2017 and trend analysis from 1990 to 2017 from the Global Burden of Disease Study
Source: J Hematol Oncol. 2019 Sep 12;12:96. doi: 10.1186/s13045-019-0783-9 (PMC6740016; doi:10.1186/s13045-019-0783-9)
Supplement: Supplementary file 6 — The age standardized of death of cancers for 21 regions compared with the global’s in 2017(%). (PDF 148 kb) [file 13045_2019_783_MOESM6_ESM.pdf]

The age standardized of death of cancers for 21 regions compared with the global's in 2017(%).

Tumor types	East Asia	Southeast Asia	Oceania	Central Asia	Central Europe	Eastern Europe	High-income Asia Pacific	Australasia	Western Europe	Southern Latin America	High-income North America	Caribbean	Andean Latin America	Central Latin America	Tropical Latin America
Esophageal cancer	202.34	47.47	42.17	109.30	49.76	53.06	59.91	68.87	62.14	78.59	63.25	62.02	26.08	27.02	87.83
Stomach cancer	170.13	63.94	127.54	130.64	78.60	116.36	129.32	40.45	58.53	111.95	32.45	66.07	155.81	84.90	84.28
Liver cancer	210.25	117.97	98.73	90.86	51.23	38.46	100.90	42.21	50.41	44.02	48.31	56.09	71.58	60.35	51.19
Larynx cancer	63.94	88.18	119.82	104.57	155.86	128.63	23.06	38.58	68.86	94.19	58.99	192.69	47.64	71.14	137.72
Tracheal, bronchus, and lung cancer	151.38	92.43	103.58	67.90	144.49	88.89	87.47	94.02	114.56	84.28	143.57	84.58	41.13	42.00	60.80
Breast cancer	59.49	105.57	151.57	103.10	130.19	127.09	62.49	116.46	130.68	145.10	121.02	132.01	77.52	86.22	106.41
Cervical cancer	72.07	129.92	310.69	109.01	110.71	81.32	42.38	38.03	43.66	169.31	47.52	187.56	195.95	147.98	134.17
Uterine cancer	59.04	102.59	304.72	133.79	168.28	193.32	74.66	117.01	113.62	141.37	135.59	225.60	159.15	105.33	105.88
Prostate cancer	55.58	87.10	159.75	74.40	124.75	81.57	57.59	150.71	131.65	170.01	113.00	317.18	172.68	142.26	168.59
Colon and rectum cancer	89.12	89.63	81.10	78.26	181.56	142.37	122.22	131.90	127.74	139.54	124.82	115.06	70.88	69.82	89.20
Lip and oral cavity cancer	48.45	100.89	110.69	71.57	111.75	103.84	45.64	69.44	63.09	53.08	53.89	94.80	44.31	41.70	93.91
Nasopharynx cancer	165.57	202.06	240.71	41.59	45.12	36.23	40.20	62.57	54.30	21.45	26.43	65.87	19.39	34.26	26.36
Other pharynx cancer	20.98	66.35	90.52	55.15	135.85	107.76	47.35	62.36	81.61	30.31	47.24	71.94	45.75	27.56	105.10
Gallbladder and biliary tract cancer	68.26	92.33	61.34	51.49	135.69	59.71	233.75	73.92	88.35	311.39	42.52	54.65	159.17	100.08	104.58
Pancreatic cancer	79.78	64.42	62.76	89.07	161.07	130.27	151.18	129.45	154.29	153.48	154.77	86.25	81.49	79.16	100.17
Malignant skin melanoma	35.85	34.70	99.61	81.49	245.16	232.08	30.10	571.04	241.05	143.09	259.09	71.25	103.87	81.84	126.89
Non-melanoma skin cancer	104.81	99.32	148.95	98.16	143.83	111.56	40.45	198.16	83.66	129.60	82.04	163.52	117.03	145.30	151.09
Ovarian cancer	57.89	100.11	87.59	96.36	172.76	152.81	75.31	120.08	144.36	114.00	139.34	92.74	89.05	99.41	92.60
Testicular cancer	38.82	51.03	459.48	169.21	271.05	170.18	42.11	99.06	111.66	462.46	122.92	74.58	214.23	312.56	158.30
Kidney cancer	54.79	58.74	48.30	126.89	215.18	213.77	107.87	185.06	184.52	241.72	175.53	85.56	102.84	110.31	99.64
Bladder cancer	68.90	77.58	76.54	85.59	175.04	115.57	79.20	109.27	163.43	115.33	129.79	101.76	54.45	52.75	87.13
Brain and nervous system cancer	108.98	89.07	58.18	120.82	163.80	113.70	45.24	133.70	127.57	95.15	118.42	82.89	86.62	72.10	142.52
Thyroid cancer	71.81	163.52	159.16	70.35	82.42	93.27	109.15	76.10	79.08	99.25	73.72	100.55	188.35	131.01	94.93
Mesothelioma	38.09	57.76	82.72	50.25	79.88	69.20	97.07	469.04	291.66	97.18	158.62	44.20	58.92	64.26	97.27
Hodgkin lymphoma	36.61	83.50	106.52	105.34	108.57	132.41	20.29	59.01	81.08	100.57	72.12	124.63	86.48	108.12	77.15
Non-Hodgkin lymphoma	69.64	87.41	87.28	59.24	90.64	80.99	99.90	144.61	118.71	113.74	149.27	108.52	126.56	83.82	86.29
Multiple myeloma	47.22	51.57	80.83	46.82	119.17	103.51	94.98	192.80	170.88	133.31	194.76	157.89	109.95	95.06	110.62
Leukemia	84.12	118.84	124.50	81.24	109.73	98.22	69.61	127.89	119.58	103.59	119.49	111.28	108.99	105.57	89.23
Other malignant neoplasms	77.63	99.77	120.64	116.64	105.29	186.58	64.65	79.27	89.63	103.78	76.81	112.18	97.00	84.65	108.05

North Africa and Middle East	South Asia	Central Sub-Saharan Africa	Eastern Sub-Saharan Africa	Southern Sub-Saharan Africa	Western Sub-Saharan Africa
41.82	75.08	139.96	152.39	192.20	77.43
79.83	67.87	69.15	61.63	50.24	76.62
58.15	33.92	77.55	76.49	65.45	111.51
105.74	205.31	97.82	84.60	103.74	82.38
64.13	41.14	40.57	33.00	64.88	36.68
76.86	101.80	120.68	112.16	129.61	163.40
40.19	114.41	410.53	323.14	319.11	266.17
58.52	85.63	106.20	112.91	126.30	97.60
84.17	66.76	169.49	181.72	232.11	392.11
69.16	61.67	76.32	89.28	81.73	73.32
32.34	287.68	85.26	92.28	100.99	54.08
60.92	104.31	56.60	121.80	53.61	51.35
22.73	348.90	33.32	52.98	38.66	29.07
63.65	135.39	60.24	61.29	54.49	60.32
70.37	51.12	59.07	57.86	98.38	80.44
59.95	29.21	75.49	89.99	150.22	61.42
53.46	93.91	143.34	167.01	272.63	62.81
69.73	95.76	80.02	114.26	110.12	75.02
83.58	122.46	90.77	51.53	133.42	45.11
60.45	35.16	47.98	43.54	64.13	51.68
130.19	64.75	110.89	95.94	106.49	90.88
117.44	76.72	48.38	67.89	55.76	49.43
87.69	124.00	73.40	152.04	76.43	52.49
104.90	54.04	42.04	34.66	165.07	36.06
150.63	128.36	119.06	214.97	77.74	241.84
88.50	84.22	65.42	177.18	94.11	99.83
88.16	74.33	82.14	119.11	136.09	103.87
119.19	79.38	80.12	89.81	89.59	69.20
90.39	99.99	110.44	192.48	98.19	116.52
