# Supplementary material for: Incidence and death in 29 cancer groups in 2017 and trend analysis from 1990 to 2017 from the Global Burden of Disease Study
Source: J Hematol Oncol. 2019 Sep 12;12:96. doi: 10.1186/s13045-019-0783-9 (PMC6740016; doi:10.1186/s13045-019-0783-9)
Supplement: Supplementary file 7 — The age standardized of incidence of cancers for 21 regions compared with the global’s in 2017(%). (PDF 148 kb) [file 13045_2019_783_MOESM7_ESM.pdf]

The age standardized of incidence of cancers for 21 regions compared with the global's in 2017(%).

Tumor types	East Asia	Southeast Asia	Oceania	Central Asia	Central Europe	Eastern Europe	High-income Asia Pacific	Australasia	Western Europe	Southern Latin America
Esophageal cancer	204.73	42.43	37.48	95.98	45.46	58.64	87.16	75.28	67.71	67.99
Stomach cancer	186.25	44.39	90.06	91.94	61.06	115.52	192.02	57.17	68.30	80.45
Liver cancer	222.08	98.83	84.68	75.39	42.71	32.92	128.29	44.33	54.04	35.37
Larynx cancer	76.68	72.98	79.07	84.87	169.77	141.69	63.48	80.41	123.19	89.63
Tracheal, bronchus, and lung cancer	153.16	76.20	86.85	57.61	130.58	97.06	106.61	116.84	125.75	70.50
Breast cancer	76.08	77.29	83.28	81.28	132.46	126.65	111.74	182.78	187.72	118.95
Cervical cancer	74.70	123.96	316.55	106.92	108.53	104.52	74.00	43.86	57.70	201.27
Uterine cancer	66.71	59.57	128.32	112.94	212.51	182.02	90.69	153.55	200.29	105.35
Prostate cancer	46.91	45.94	63.57	40.63	104.43	95.15	89.09	291.45	205.32	118.10
Colon and rectum cancer	98.21	63.36	48.35	53.18	148.80	129.96	180.59	199.97	166.57	109.70
Lip and oral cavity cancer	55.86	96.03	89.79	61.20	97.96	102.44	72.26	122.13	101.23	56.15
Nasopharynx cancer	179.50	188.20	223.10	38.61	37.50	43.50	35.23	57.19	60.20	20.50
Other pharynx cancer	27.39	59.43	70.40	51.99	157.24	131.06	73.40	104.63	141.87	32.00
Gallbladder and biliary tract cancer	65.32	76.79	48.44	39.61	115.16	55.23	322.68	89.62	110.09	259.05
Pancreatic cancer	76.90	59.28	58.46	82.21	153.92	133.38	172.69	145.98	157.57	140.61
Malignant skin melanoma	22.60	10.27	21.42	35.38	210.69	181.03	51.96	1247.97	417.48	82.30
Non-melanoma skin cancer	13.52	10.41	18.52	60.17	62.63	64.93	9.18	488.06	86.26	24.68
Ovarian cancer	59.94	116.67	97.44	94.18	165.83	157.84	103.32	123.20	148.93	112.72
Testicular cancer	40.00	29.06	81.44	72.49	461.70	129.07	176.45	384.18	432.15	560.12
Kidney cancer	56.13	67.25	59.02	127.24	175.19	203.13	89.61	178.13	185.54	235.29
Bladder cancer	65.86	62.51	55.29	74.15	181.12	123.97	104.97	136.58	214.50	110.37
Brain and nervous system cancer	150.84	64.00	40.76	93.90	162.78	133.86	107.51	143.76	202.35	70.26
Thyroid cancer	70.55	112.73	67.85	63.64	119.68	159.41	228.58	167.78	145.11	97.11
Mesothelioma	34.44	63.96	91.84	59.87	95.22	86.56	89.72	489.17	300.14	99.06
Hodgkin lymphoma	91.79	41.40	41.88	82.83	224.15	310.52	66.82	253.33	278.21	73.83
Non-Hodgkin lymphoma	73.61	55.94	50.29	48.29	114.96	99.40	139.35	257.53	205.17	88.75
Multiple myeloma	51.99	41.27	59.90	37.97	105.64	109.03	110.62	278.16	213.67	116.57
Leukemia	155.84	94.21	93.73	70.18	94.25	102.21	78.02	142.25	131.13	78.19
Other malignant neoplasms	148.30	61.64	65.58	68.76	118.12	210.02	121.98	163.75	157.75	63.53

High-income North America	Caribbean	Andean Latin America	Central Latin America	Tropical Latin America	North Africa and Middle East	South Asia	Central Sub-Saharan Africa	Eastern Sub-Saharan Africa	Southern Sub-Saharan Africa	Western Sub-Saharan Africa
65.75	55.74	22.12	23.33	78.71	36.88	66.55	122.87	132.10	168.73	67.39
42.27	51.70	108.07	84.19	61.55	56.41	46.57	46.44	41.48	33.82	50.28
54.90	46.09	57.71	48.42	41.90	48.57	28.10	63.84	63.32	55.38	94.08
120.24	179.37	34.62	57.32	116.94	90.15	137.96	60.03	53.22	71.09	51.52
163.02	71.33	33.43	34.64	50.27	53.97	33.88	33.55	26.56	54.14	29.72
204.79	115.11	61.04	87.18	93.07	74.66	58.17	55.07	53.52	72.07	85.04
68.19	182.44	174.14	144.38	133.82	36.82	99.87	305.66	243.18	255.73	212.83
281.71	187.64	102.59	92.68	71.91	48.97	36.32	34.02	36.86	50.78	34.15
284.45	238.76	117.77	158.35	131.64	89.96	25.59	57.13	64.35	100.74	141.15
168.35	101.11	61.37	65.53	69.96	53.26	34.90	39.59	45.96	47.83	38.58
115.44	84.39	40.48	38.95	81.70	30.64	221.24	55.50	61.40	75.95	38.11
37.62	56.94	17.83	30.81	29.41	57.97	87.09	44.79	97.69	42.40	43.21
114.66	67.78	39.78	25.79	99.06	21.63	266.83	23.82	38.83	31.22	20.76
76.86	43.62	123.34	78.53	81.75	50.32	104.58	45.42	46.49	41.21	45.54
172.94	79.98	74.36	72.99	91.96	65.56	46.71	54.27	52.95	90.10	73.57
446.59	36.53	38.42	38.71	57.89	30.26	8.27	17.05	21.87	41.01	15.49
864.49	22.64	44.53	120.54	148.85	19.14	5.35	28.43	21.06	104.41	11.78
141.97	93.12	92.72	107.95	93.69	71.64	89.84	65.25	97.98	95.91	65.26
354.14	60.42	109.76	261.83	118.99	78.05	32.61	16.23	8.76	26.68	8.22
246.26	94.61	102.32	115.16	102.12	62.57	37.82	54.00	50.06	66.47	59.21
166.32	90.84	41.96	46.18	73.07	129.37	41.80	62.68	54.91	74.66	53.96
146.73	64.35	61.26	53.65	100.28	96.10	52.09	32.92	46.41	36.68	34.46
172.64	90.71	130.81	109.05	72.40	101.10	72.64	23.40	58.35	36.92	17.55
141.90	50.32	61.90	69.01	105.04	107.41	56.39	46.09	37.00	163.86	40.31
251.14	90.31	42.68	68.62	50.55	112.35	52.81	42.16	81.21	31.02	96.69
246.72	86.63	82.42	61.90	60.98	70.20	47.91	34.91	96.53	52.60	57.57
245.17	135.16	86.58	80.52	89.71	75.54	53.67	56.76	81.76	100.73	72.94
106.12	86.11	88.30	87.19	68.80	94.25	59.05	57.26	66.20	60.65	50.32
122.11	71.42	52.31	51.18	61.81	58.18	52.49	57.53	103.46	53.03	64.52
