# Supplementary material for: Incidence and death in 29 cancer groups in 2017 and trend analysis from 1990 to 2017 from the Global Burden of Disease Study
Source: J Hematol Oncol. 2019 Sep 12;12:96. doi: 10.1186/s13045-019-0783-9 (PMC6740016; doi:10.1186/s13045-019-0783-9)
Supplement: Supplementary file 8 — The number, age standardized of death and incidence of 29 specified cancer groups in 2017 by different HDI regions. (PDF 54 kb) [file 13045_2019_783_MOESM8_ESM.pdf]

The number, age standardized of death and incidence of 29 specified cancer groups in 2017 by different HDI regions.

Tumor types	Death							
	Number(95% UI)				Age-standardized per 100,000 (95% UI)			
	High Income	Upper Middle Income	Lower Middle Income	Low Income	High Income	Upper Middle Income	Lower Middle Income	Low Income
Esophageal cancer	76575(78420-74748)	255312(265345-245424)	79512(83132-76111)	20861(22812-19223)	3.45(3.54-3.37)	7.91(8.21-7.6)	3.6(3.77-3.44)	7.35(8.04-6.79)
Stomach cancer	176252(181667-171684)	482416(498634-467802)	169287(175562-163168)	30714(33263-28521)	7.44(7.66-7.25)	15.03(15.52-14.57)	7.78(8.08-7.5)	10.87(11.78-10.12)
Liver cancer	143659(148380-138233)	501743(524827-479356)	130012(136795-122915)	36566(54723-30963)	6.39(6.59-6.17)	15.17(15.85-14.51)	5.74(6.03-5.43)	11.98(17.75-10.16)
Larynx cancer	20762(21338-20262)	42782(43932-41621)	57788(60796-55020)	4719(5182-4318)	0.96(0.98-0.94)	1.3(1.33-1.27)	2.49(2.62-2.37)	1.56(1.73-1.43)
Tracheal, bronchus, and lu	631726(642834-620360)	930852(960812-898682)	278350(296220-263744)	29574(33439-26933)	27.92(28.41-27.42)	28.62(29.54-27.65)	12.71(13.56-12.03)	10.64(11.98-9.72)
Breast cancer	186783(192053-181859)	195244(202768-178514)	199690(225902-181448)	27974(31601-25600)	8.58(8.82-8.35)	5.95(6.18-5.44)	8.29(9.44-7.52)	8.47(9.59-7.79)
Cervical cancer	32444(33445-31577)	94333(98598-78096)	98309(112286-90179)	33591(37319-28515)	1.57(1.62-1.53)	2.86(2.99-2.39)	4.02(4.6-3.69)	9.89(11.11-8.34)
Uterine cancer	28671(29597-27816)	29228(30280-28234)	23464(24917-22056)	3585(4002-3137)	1.24(1.29-1.21)	0.9(0.93-0.87)	1.05(1.11-0.98)	1.23(1.38-1.07)
Prostate cancer	158628(209802-130766)	137454(161392-117586)	96274(108026-75512)	22196(25150-15990)	6.16(8.22-5.11)	4.61(5.4-3.93)	5.36(6.01-4.22)	9.61(10.87-6.93)
Colon and rectum cancer	354707(363414-345039)	340618(349862-329094)	171356(179622-161361)	25601(28376-23559)	14.99(15.36-14.58)	10.76(11.05-10.4)	8.26(8.65-7.78)	9.45(10.34-8.77)
Lip and oral cavity cancer	33432(34259-32614)	44495(45944-43008)	108909(115946-101114)	6391(6838-5942)	1.55(1.59-1.51)	1.37(1.41-1.32)	4.8(5.11-4.47)	2.16(2.32-2.01)
Nasopharynx cancer	8308(8636-8010)	33955(36077-32198)	23984(25527-22639)	2785(3072-2466)	0.41(0.42-0.39)	1.02(1.08-0.97)	0.99(1.05-0.93)	0.81(0.9-0.72)
Other pharynx cancer	20260(21045-19528)	17449(18831-16499)	77048(83449-63538)	2510(2963-2073)	0.99(1.02-0.95)	0.52(0.56-0.49)	3.3(3.57-2.71)	0.81(0.96-0.67)
Gallbladder and biliary tra	62392(65096-59162)	56933(60548-47713)	49937(55987-40267)	4164(5378-3625)	2.55(2.68-2.43)	1.8(1.91-1.51)	2.35(2.67-1.92)	1.57(2.06-1.35)
Pancreatic cancer	198919(203210-194894)	160284(164030-156131)	71310(75198-67989)	8894(9529-8112)	8.57(8.76-8.4)	4.98(5.09-4.85)	3.36(3.55-3.21)	3.23(3.47-2.94)
Malignant skin melanoma	34124(39538-23453)	17634(20581-14796)	7784(9172-6405)	2000(2524-1599)	1.61(1.94-1.13)	0.56(0.65-0.47)	0.34(0.41-0.28)	0.6(0.77-0.49)
Non-melanoma skin canc	17181(17682-16685)	29771(30594-28740)	15142(16558-14118)	2688(3055-1997)	0.68(0.7-0.66)	0.98(1.01-0.95)	0.78(0.85-0.72)	1.06(1.21-0.76)
Ovarian cancer	62110(64157-60032)	56069(57647-54485)	50857(54699-47423)	6417(7058-5643)	2.83(2.92-2.74)	1.69(1.74-1.65)	2.13(2.29-1.99)	1.98(2.19-1.73)
Testicular cancer	1618(1681-1556)	2986(3087-2892)	2735(3009-2494)	308(354-269)	0.11(0.12-0.11)	0.1(0.11-0.1)	0.1(0.1-0.09)	0.07(0.07-0.06)
Kidney cancer	68682(70896-63020)	46014(48106-43072)	20517(21488-18672)	2937(3418-2457)	3.02(3.12-2.81)	1.44(1.51-1.35)	0.9(0.94-0.82)	0.84(0.98-0.7)
Bladder cancer	87730(90143-85237)	62388(68977-60362)	38992(41560-36479)	6782(7549-6090)	3.49(3.59-3.39)	2.03(2.24-1.97)	1.98(2.13-1.85)	2.72(3.02-2.43)
Brain and nervous system	63338(66387-50545)	105691(114705-90365)	67157(75319-56607)	9808(11303-7777)	3.38(3.56-2.81)	3.42(3.72-2.93)	2.54(2.84-2.13)	2.09(2.42-1.66)
Thyroid cancer	10397(10765-10069)	13757(14853-13230)	14858(16403-14033)	2081(2325-1819)	0.45(0.47-0.44)	0.44(0.47-0.42)	0.65(0.73-0.62)	0.64(0.72-0.56)
Mesothelioma	17318(17998-16664)	7284(7568-6856)	4703(5531-4209)	538(755-393)	0.75(0.78-0.72)	0.22(0.23-0.21)	0.2(0.24-0.18)	0.17(0.22-0.12)
Hodgkin lymphoma	5165(6359-4404)	7871(8954-6440)	15982(19458-13116)	3475(4686-2692)	0.28(0.35-0.25)	0.26(0.29-0.21)	0.58(0.71-0.48)	0.73(0.98-0.57)
Non-Hodgkin lymphoma	89146(91048-87145)	75490(78055-73406)	67458(70747-64008)	15691(17321-13330)	3.93(4.02-3.84)	2.38(2.46-2.32)	2.8(2.94-2.66)	4.07(4.47-3.48)
Multiple myeloma	51370(60707-45251)	30408(33082-27913)	21401(23192-19646)	3646(4005-3221)	2.18(2.61-1.95)	0.93(1.02-0.86)	0.97(1.05-0.9)	1.28(1.41-1.13)
Leukemia	106180(108544-103593)	119277(125460-105735)	102355(111707-89222)	18509(20642-15263)	4.91(5.02-4.78)	4.11(4.33-3.64)	4.05(4.4-3.53)	4.16(4.63-3.41)
Other malignant neoplasm	79862(83049-71202)	132621(138537-116932)	118356(124364-109196)	27398(30127-23729)	3.83(4.03-3.48)	4.29(4.47-3.81)	4.88(5.13-4.55)	6.72(7.41-5.78)

Incidence							
Number(95% UI)				Age-standardized per 100,000 (95% UI)			
**High Income**	**Upper Middle Income**	**Lower Middle Income**	**Low Income**	**High Income**	**Upper Middle Income**	**Lower Middle Income**	**Low Income**
91384(93922-88993)	277632(288899-266281)	78909(82526-75693)	20521(22453-18891)	4.22(4.34-4.11)	8.5(8.85-8.16)	3.45(3.61-3.3)	6.94(7.59-6.4)
296469(307159-286902)	710403(739481-681401)	173358(179794-167254)	30637(33266-28369)	13.06(13.52-12.66)	21.7(22.58-20.84)	7.68(7.97-7.41)	10.3(11.2-9.58)
180407(187920-172668)	596470(626621-566251)	129919(136740-122902)	37169(55940-31426)	8.37(8.7-8.03)	17.87(18.76-16.99)	5.55(5.83-5.26)	11.62(17.31-9.85)
59074(60823-57381)	76659(79167-74384)	68882(72117-65795)	5170(5635-4714)	2.86(2.95-2.78)	2.28(2.35-2.21)	2.87(3.01-2.75)	1.65(1.8-1.51)
785324(801746-769057)	058512(1097016-102070§	275999(293793-261757)	28703(32557-26097)	35.38(36.13-34.66)	32.23(33.39-31.09)	12.22(13.04-11.58)	9.96(11.22-9.09)
796788(819104-772924)	689825(720886-623878)	418989(465690-383021)	47622(54030-43276)	41.02(42.17-39.81)	20.61(21.53-18.63)	16.2(18.06-14.8)	13.12(14.91-11.97)
78737(81254-76288)	225604(236566-184133)	224030(252242-205900)	70476(77786-60264)	4.79(4.95-4.62)	6.89(7.22-5.65)	8.2(9.27-7.54)	17.58(19.47-14.99)
201118(208330-194449)	137439(143934-131706)	60227(63500-56870)	6529(7314-5740)	9.96(10.32-9.63)	4.04(4.23-3.87)	2.45(2.58-2.32)	2.01(2.24-1.76)
761824(1066802-658180)	398840(466153-338937)	143674(160573-114763)	26253(29751-18991)	33.92(47.73-29.34)	12.49(14.6-10.59)	7.28(8.14-5.82)	10.59(12-7.66)
868019(889450-843585)	697695(719327-672213)	229417(239986-216357)	29870(33382-27319)	39.12(40.09-38.04)	21.42(22.07-20.62)	10.38(10.85-9.8)	10.26(11.3-9.47)
102719(105693-99708)	92003(95154-88657)	184451(197268-172014)	9517(10106-8866)	5.05(5.2-4.91)	2.81(2.9-2.71)	7.61(8.11-7.11)	2.95(3.14-2.75)
12541(13231-11926)	56119(61397-51769)	35981(38576-33545)	4279(4850-3720)	0.72(0.76-0.68)	1.7(1.86-1.57)	1.35(1.45-1.27)	1.07(1.2-0.94)
51243(53293-49363)	30118(32585-28269)	94788(102579-78922)	2899(3416-2409)	2.63(2.74-2.54)	0.88(0.96-0.83)	3.9(4.21-3.24)	0.89(1.05-0.74)
96173(106666-86904)	61300(65624-49809)	48734(54538-39210)	4035(5219-3530)	4.02(4.42-3.66)	1.92(2.05-1.56)	2.22(2.51-1.8)	1.45(1.9-1.26)
212477(217771-207049)	156132(160093-151978)	68788(72647-65639)	8623(9226-7880)	9.26(9.49-9.04)	4.81(4.93-4.68)	3.13(3.31-2.99)	3.01(3.23-2.74)
238119(286766-172808)	53274(62624-45164)	14288(16900-11813)	2580(3264-2058)	13.14(16.54-9.79)	1.67(1.97-1.42)	0.57(0.68-0.48)	0.7(0.89-0.57)
084717(8424688-4204200	205464(1720999-795775	308828(470940-189443)	58905(89942-36260)	285.22(390.4-197.62)	37.69(53.36-24.87)	12.91(19.81-7.73)	15.44(23.15-9.74)
93179(96420-90098)	92881(95614-90058)	88730(96373-82080)	10461(11494-9337)	4.81(4.98-4.66)	2.83(2.91-2.74)	3.38(3.65-3.14)	2.81(3.09-2.47)
37178(39199-35263)	24852(26616-23630)	8579(9458-7895)	601(691-524)	3.13(3.31-2.97)	0.88(0.95-0.84)	0.28(0.3-0.26)	0.11(0.13-0.1)
181463(187564-173334)	128605(134700-122195)	68774(72607-62342)	13138(15507-10946)	9.04(9.35-8.69)	4.05(4.23-3.86)	2.66(2.8-2.43)	2.76(3.25-2.32)
238709(245918-231289)	147324(162340-142134)	75641(79421-69681)	10562(11779-9510)	10.43(10.75-10.1)	4.58(5.05-4.42)	3.44(3.63-3.21)	3.8(4.22-3.42)
126505(134629-103026)	181321(207927-152319)	82605(92564-69863)	12571(14441-9957)	8.11(8.68-6.92)	6.36(7.34-5.36)	2.98(3.32-2.51)	2.37(2.74-1.89)
92035(96161-88841)	87997(98167-83741)	67888(74753-62333)	6703(7685-5842)	5.31(5.57-5.11)	2.69(3-2.56)	2.45(2.7-2.27)	1.56(1.79-1.37)
19117(19994-18351)	8552(8923-8008)	6126(7245-5432)	749(1094-545)	0.85(0.89-0.82)	0.26(0.27-0.24)	0.25(0.3-0.22)	0.21(0.3-0.15)
36086(47583-32925)	35763(40017-28671)	24439(29047-20036)	4483(6065-3478)	2.79(3.74-2.54)	1.27(1.41-1.03)	0.83(0.99-0.68)	0.86(1.15-0.68)
241885(248091-235721)	141173(146138-136405)	84239(88392-79812)	18980(21138-16115)	12.12(12.45-11.82)	4.47(4.63-4.32)	3.3(3.46-3.14)	4.38(4.81-3.74)
82780(100878-74966)	42308(45817-38325)	23477(25509-21587)	3746(4122-3304)	3.75(4.62-3.43)	1.28(1.38-1.16)	1.02(1.11-0.94)	1.26(1.38-1.11)
151265(157122-145576)	216085(230331-188309)	124722(136465-109582)	23728(26527-19660)	7.51(7.77-7.24)	8.15(8.8-7.05)	4.64(5.06-4.06)	4.63(5.14-3.81)
208997(220385-189062)	325467(342770-287024)	143198(151260-132983)	33720(37166-29199)	12.43(13.43-11.46)	10.98(11.57-9.77)	5.55(5.86-5.18)	7.21(7.93-6.24)
