# Supplementary material for: Incidence and death in 29 cancer groups in 2017 and trend analysis from 1990 to 2017 from the Global Burden of Disease Study
Source: J Hematol Oncol. 2019 Sep 12;12:96. doi: 10.1186/s13045-019-0783-9 (PMC6740016; doi:10.1186/s13045-019-0783-9)
Supplement: Supplementary file 9 — The ASDR of 29 specified cancer groups in 2017 and 1990, and relative changes between 1990 and 2017 in 195 countries and territories. (PDF 1561 kb) [file 13045_2019_783_MOESM9_ESM.pdf]

Age-standardized Death in 2017

	Total	Esophageal cancer	Stomach cancer	Liver cancer	Larynx cancer	Tracheal, bronchus, and lung cancer	Breast cancer	Cervical cancer	Uterine cancer	Prostate cancer	Colon and rectum cancer	Lip and oral cavity cancer	Nasopharynx cancer	Other pharynx cancer	Gallbladder and biliary tract cancer	Pancreatic cancer	Malignant skin melanoma	Non-melanoma skin cancer	Ovarian cancer	Testicular cancer
China	137.35	11.25	18.83	21.30	1.00	36.29	4.49	2.29	0.62	3.03	10.10	1.09	1.40	0.27	1.51	4.48	0.28	0.90	1.26	0.04
North Korea	133.05	8.41	20.56	25.91	1.12	23.77	6.75	3.78	1.11	3.44	9.51	1.12	1.54	0.32	1.73	3.12	0.34	0.69	1.38	0.06
Taiwan	139.51	5.97	9.17	25.09	0.74	27.51	6.18	2.11	0.87	3.82	17.66	5.75	2.17	2.32	2.00	5.83	0.45	0.50	1.67	0.05
Cambodia	113.91	2.68	10.24	8.42	1.88	20.61	8.90	6.29	1.57	5.89	11.24	2.48	1.91	0.92	1.95	3.29	0.31	0.88	2.34	0.05
Indonesia	97.07	0.84	8.03	8.24	1.48	18.62	7.42	4.26	1.30	5.54	7.60	1.38	1.67	0.72	1.61	3.73	0.27	0.89	2.13	0.00
Laos	111.67	2.61	9.09	9.86	1.74	21.69	7.71	5.40	1.32	6.20	10.24	2.00	1.72	0.83	1.79	3.34	0.33	0.87	2.13	0.04
Malaysia	109.55	2.78	7.15	6.89	1.26	19.75	9.82	4.11	1.17	4.71	16.63	2.29	3.44	0.84	1.23	3.55	0.35	0.85	2.11	0.12
Maldives	56.31	1.49	2.37	4.78	0.81	7.27	4.09	1.80	0.27	3.18	5.09	3.53	0.40	0.26	0.88	2.69	0.37	0.45	1.84	0.00
Myanmar	135.08	9.52	10.67	10.25	1.80	18.56	12.98	7.13	1.92	6.67	12.49	2.36	1.95	0.90	2.13	4.44	0.38	0.89	3.32	0.05
Philippines	109.79	1.29	4.19	11.64	1.07	18.45	12.94	3.57	1.43	7.13	13.44	2.29	1.96	0.57	0.71	3.87	0.39	0.71	2.97	0.12
Sri Lanka	69.58	3.94	4.06	3.21	0.79	7.07	5.42	1.69	0.80	3.08	5.77	4.08	1.01	1.56	1.99	2.78	0.19	1.19	1.60	0.04
Thailand	106.03	2.88	4.38	22.65	1.25	20.18	5.68	3.25	0.50	3.36	8.81	2.91	1.33	0.66	4.81	3.54	0.22	0.86	1.74	0.05
Timor-Leste	104.88	2.24	8.83	8.81	1.62	18.72	5.99	5.03	1.26	8.00	8.63	2.03	1.85	0.68	1.88	3.11	0.33	1.09	1.89	0.05
Vietnam	133.02	3.49	8.94	15.26	1.51	40.57	6.03	4.34	0.70	3.07	13.93	3.82	1.80	2.11	1.23	3.26	0.21	0.72	1.88	0.08
Fiji	105.48	2.59	5.85	8.58	1.17	8.18	16.70	10.35	2.83	8.63	9.40	2.59	0.49	1.10	1.22	3.72	0.49	0.73	0.98	0.47
Kiribati	138.18	4.46	11.85	11.52	1.05	16.18	12.21	27.41	3.61	5.01	9.56	7.52	0.68	1.42	1.47	2.93	0.47	0.49	1.02	1.25
Marshall Islands	159.28	3.41	15.75	14.43	2.19	28.08	13.49	8.81	4.24	10.99	14.13	2.72	2.42	1.49	1.40	5.48	0.90	1.47	2.78	0.24
Federated States of Micronesia	134.40	2.22	12.05	11.39	1.63	24.91	13.44	7.37	3.87	8.30	11.59	2.47	1.77	1.23	1.25	4.56	0.71	1.29	2.73	0.18
Papua New Guinea	134.17	2.17	16.15	9.65	2.10	27.55	10.34	10.11	3.31	8.13	8.65	2.59	2.32	1.35	1.42	3.10	0.78	1.26	1.89	0.27
Samoa	87.13	1.61	10.81	4.76	0.61	7.82	6.79	4.70	2.76	5.73	8.84	1.28	1.24	0.26	1.14	3.31	1.37	1.34	2.29	0.23
Solomon Islands	118.85	2.26	13.03	10.30	1.70	21.34	9.57	8.44	3.17	8.60	8.01	2.10	1.85	1.09	1.24	3.14	0.69	1.23	1.91	0.19
Tonga	164.36	2.54	14.44	24.65	1.19	27.17	18.23	9.38	2.75	13.68	7.55	1.96	1.27	0.89	0.98	4.89	0.70	2.67	2.14	6.46
Vanuatu	166.28	2.22	14.00	16.62	2.19	25.47	13.06	8.90	3.86	17.25	11.62	3.50	2.67	1.35	1.98	4.57	1.46	2.18	2.51	0.23
Armenia	139.01	1.67	12.65	9.97	2.41	29.12	12.61	3.85	2.02	6.03	12.77	1.18	0.21	0.38	1.56	9.28	0.50	1.00	2.77	0.16
Azerbaijan	120.07	8.63	20.19	8.32	2.16	19.90	6.81	2.60	1.19	5.58	9.02	1.25	0.20	0.51	1.49	5.88	0.48	0.92	1.71	0.03
Georgia	137.22	2.63	14.33	7.52	3.19	25.53	13.54	4.46	2.95	5.04	10.69	2.66	0.32	1.19	2.21	5.53	1.49	1.77	3.32	0.64
Kazakhstan	108.20	6.87	13.00	5.77	1.39	18.74	8.00	3.71	1.63	3.36	11.29	2.09	0.38	0.85	1.14	5.70	0.96	0.65	2.98	0.21
Kyrgyzstan	82.83	3.61	14.22	7.20	0.74	11.21	5.53	4.29	1.26	2.52	6.55	1.42	0.29	0.65	0.97	4.06	0.52	0.88	2.12	0.12
Mongolia	238.41	20.52	37.65	100.14	1.35	26.53	4.88	5.81	1.05	2.66	6.83	1.96	0.25	0.54	2.30	4.76	0.13	0.42	1.93	0.01
Tajikistan	85.61	5.23	17.83	5.63	0.70	7.84	5.28	1.63	1.25	3.93	6.79	0.85	0.49	0.50	0.58	3.91	0.42	0.53	1.68	0.00
Turkmenistan	89.95	9.08	9.79	5.71	0.94	9.95	7.62	4.08	0.47	3.51	7.53	2.14	0.30	1.02	1.09	3.77	0.47	0.55	1.84	0.23
Uzbekistan	85.45	4.99	10.93	6.03	1.61	10.44	7.04	3.33	1.05	4.13	7.45	1.70	0.44	0.97	0.65	3.78	0.41	0.73	1.36	0.13
Albania	105.39	1.42	10.63	7.25	2.08	24.21	5.74	1.47	0.84	7.97	7.08	1.28	0.37	0.67	1.16	5.53	0.68	1.15	1.23	0.26
Bosnia and Herzegovina	150.71	2.16	9.69	7.86	2.42	36.61	9.92	2.88	1.54	7.49	20.39	1.63	0.23	1.12	3.48	9.04	1.52	0.98	3.64	0.20
Bulgaria	132.30	1.81	9.08	5.51	2.74	27.07	10.14	3.83	2.17	6.26	19.24	2.01	0.34	1.21	1.64	8.56	1.38	1.10	3.52	0.36
Croatia	151.82	2.56	8.91	5.66	2.18	31.43	11.15	2.18	1.80	8.19	23.74	2.34	0.22	1.81	2.99	8.44	2.35	1.27	3.52	0.23
Czech Republic	137.94	2.99	5.98	4.27	1.28	27.12	8.52	2.62	1.78	7.03	20.62	2.22	0.22	1.58	4.24	10.88	2.02	0.98	3.81	0.24
Hungary	168.28	3.53	8.28	4.61	2.81	45.30	10.99	2.64	1.51	5.99	26.15	4.42	0.53	3.44	3.57	10.43	2.01	1.08	3.51	0.27
Macedonia	130.63	1.31	11.38	6.87	3.06	29.67	9.29	2.42	1.79	7.51	15.01	1.31	0.23	0.65	1.77	7.43	2.57	1.05	2.63	0.32
Montenegro	141.02	2.47	6.18	5.68	3.98	43.79	11.00	2.70	1.18	7.58	14.54	1.95	0.13	0.74	2.19	8.35	1.34	1.03	2.67	0.21
Poland	155.74	3.02	8.85	3.32	2.46	38.66	9.27	3.33	2.03	7.05	21.63	2.54	0.33	1.56	3.53	8.80	2.20	1.14	4.55	0.26
Romania	143.01	2.38	9.48	8.57	2.97	29.53	9.62	5.55	1.36	5.82	17.56	3.30	0.70	2.86	1.66	8.38	1.33	1.49	3.17	0.26
Serbia	170.08	2.52	8.12	5.84	2.70	40.98	14.74	4.69	2.13	7.74	23.91	2.56	0.34	1.62	3.21	9.65	2.26	1.99	4.03	0.30
Slovakia	146.84	3.37	7.93	4.66	1.89	25.31	10.29	3.00	2.49	7.10	24.45	3.51	0.32	3.09	4.00	9.52	2.11	0.94	3.68	0.26
Slovenia	136.30	2.63	8.21	4.91	1.25	27.44	9.13	1.76	1.43	9.27	18.97	1.68	0.19	2.27	3.32	9.26	2.46	0.91	3.17	0.16
Belarus	116.99	2.68	13.27	2.84	2.16	19.69	7.36	3.04	1.18	5.62	14.97	2.80	0.31	1.70	1.42	6.25	1.82	0.98	3.19	0.12
Estonia	128.88	2.52	10.82	3.51	1.38	24.80	8.70	2.55	1.56	7.91	15.31	2.08	0.25	1.39	1.68	9.15	2.27	0.97	3.87	0.11
Latvia	139.71	3.32	12.11	3.60	2.15	24.46	10.32	2.34	2.66	7.89	16.61	2.72	0.25	1.62	1.26	9.54	2.11	1.35	5.08	0.18
Lithuania	140.42	3.83	12.81	3.50	2.56	24.77	9.68	3.30	2.27	7.99	16.02	2.82	0.26	2.06	1.74	8.57	2.09	0.98	5.31	0.14
Moldova	111.34	1.63	8.89	8.91	2.70	18.27	9.02	3.16	1.54	3.90	15.65	2.29	0.62	2.49	0.74	6.81	1.29	0.82	2.23	0.15
Russian Federation	125.62	3.01	13.10	4.05	1.85	21.32	9.67	2.52	2.13	3.85	16.41	2.23	0.25	1.32	1.34	7.37	1.68	0.94	3.23	0.13

Ukraine	128.89	2.71	12.18	3.48	2.43	20.52	10.45	2.68	2.13	5.57	16.82	3.34	0.48	2.19	1.30	7.23	2.26	0.94	3.62	0.29
Brunei	154.73	2.25	10.90	11.40	0.84	31.66	9.72	4.97	1.36	5.90	21.70	3.09	2.47	1.84	3.30	5.75	0.62	0.61	4.57	0.10
Japan	107.57	3.66	14.22	8.33	0.32	20.24	4.96	1.40	0.92	3.17	14.67	1.19	0.29	0.75	5.08	8.93	0.23	0.32	1.77	0.05
South Korea	109.60	2.16	14.09	15.70	0.51	22.97	4.34	1.39	0.43	3.11	11.99	0.79	0.30	0.49	5.95	7.10	0.27	0.53	1.36	0.02
Singapore	80.44	1.28	5.09	8.42	0.38	18.62	5.59	1.13	0.89	3.42	12.67	0.67	2.26	0.37	0.95	4.14	0.23	0.29	1.86	0.01
Australia	119.45	3.82	4.42	4.38	0.62	22.08	8.65	1.23	1.20	8.27	14.37	1.78	0.56	0.95	1.68	7.36	4.43	1.65	2.60	0.08
New Zealand	126.80	3.52	4.56	3.94	0.50	23.57	10.32	1.17	1.54	8.41	19.68	1.14	0.40	0.62	1.51	6.88	4.77	1.87	2.86	0.20
Andorra	122.80	3.12	5.78	2.81	0.66	26.06	9.77	1.10	1.04	9.70	14.56	1.38	0.37	0.67	1.63	7.96	1.95	0.77	3.43	0.10
Austria	118.46	2.21	5.80	5.22	0.83	24.46	9.62	1.47	1.13	6.58	12.51	1.70	0.42	1.43	2.16	9.89	2.14	0.78	3.12	0.12
Belgium	130.53	3.77	5.26	4.02	1.03	32.48	11.01	1.41	1.20	7.45	14.44	1.80	0.52	1.12	1.43	8.24	1.78	0.75	3.20	0.06
Cyprus	106.40	1.08	6.19	4.04	0.85	21.78	9.36	1.21	1.25	8.55	11.13	1.02	0.29	0.23	1.63	6.56	1.34	0.89	2.69	0.01
Denmark	149.56	3.95	4.92	3.85	0.87	35.39	11.73	1.68	1.23	11.10	19.05	1.67	0.45	1.61	1.82	9.58	2.92	0.80	4.00	0.13
Finland	105.55	2.18	4.66	3.95	0.38	19.69	8.08	0.94	1.40	7.51	10.77	1.16	0.20	0.58	2.03	9.68	2.00	0.45	3.16	0.09
France	128.41	3.35	4.98	6.59	1.38	29.42	10.08	1.46	1.24	6.95	13.78	1.84	0.55	1.73	1.41	8.64	1.74	0.74	3.03	0.11
Germany	131.74	3.40	6.85	4.49	0.98	28.22	11.04	1.52	1.08	7.34	15.31	1.69	0.38	1.80	2.45	9.90	1.90	0.58	3.37	0.16
Greece	128.27	1.17	7.46	6.00	1.63	33.08	10.11	1.57	1.21	7.02	11.88	1.01	0.65	0.33	1.98	8.33	1.24	0.91	3.03	0.14
Iceland	119.77	3.72	5.00	3.01	0.49	26.85	7.30	1.02	0.77	10.83	11.50	1.29	0.28	0.45	1.27	8.68	1.84	0.39	2.67	0.07
Ireland	129.60	5.46	6.24	3.58	0.86	27.47	10.57	1.48	1.12	8.86	15.68	1.29	0.39	0.75	1.59	8.47	2.18	1.14	4.09	0.08
Israel	111.50	1.31	5.75	3.31	0.79	19.50	10.82	1.35	1.29	5.10	14.03	0.83	0.33	0.23	1.20	9.33	2.30	1.12	2.94	0.07
Italy	118.14	1.50	7.70	7.11	1.12	23.52	9.20	1.19	1.08	5.37	13.28	1.32	0.48	0.71	2.69	8.39	1.60	0.68	2.77	0.11
Luxembourg	132.55	3.06	5.35	4.97	1.06	29.56	10.85	1.24	1.75	6.80	15.93	1.64	0.56	1.20	1.55	9.87	2.19	0.72	4.11	0.10
Malta	115.57	2.23	6.15	2.50	1.07	21.95	11.99	1.09	1.61	5.15	14.37	1.42	0.93	0.59	1.21	9.32	1.32	0.67	3.90	0.16
Netherlands	149.86	6.25	6.90	3.35	0.76	36.06	12.04	1.28	1.44	8.62	18.81	1.35	0.47	0.80	2.12	10.16	2.82	0.56	3.78	0.11
Norway	119.97	2.10	4.51	2.23	0.42	22.41	8.01	1.29	1.19	11.32	17.88	1.07	0.27	0.55	1.31	8.29	3.55	0.59	3.54	0.11
Portugal	121.59	3.01	11.54	4.92	1.89	19.60	8.64	1.75	1.35	8.06	17.83	1.91	0.56	1.51	1.71	6.67	1.30	0.93	2.09	0.09
Spain	115.14	2.27	6.79	5.52	1.64	24.72	7.21	1.23	1.33	6.04	16.07	1.53	0.49	1.01	1.68	7.07	1.21	0.73	2.36	0.07
Sweden	110.76	2.46	3.78	3.44	0.33	18.61	9.02	1.42	1.13	10.22	14.33	1.03	0.20	0.62	2.29	8.24	3.23	0.48	3.00	0.08
Switzerland	101.30	2.84	3.81	4.34	0.56	20.33	8.64	1.15	0.81	7.66	10.23	1.27	0.36	0.91	1.48	7.76	2.04	0.66	2.69	0.11
United Kingdom	137.61	6.86	5.99	3.78	0.71	30.45	11.33	1.50	1.47	9.01	15.13	1.43	0.52	0.85	1.52	8.43	2.32	0.85	4.10	0.08
Argentina	135.27	4.41	9.83	4.13	1.73	21.18	12.48	5.90	1.58	8.59	17.12	1.42	0.20	0.43	5.37	9.12	1.16	1.03	2.60	0.42
Chile	127.42	3.80	17.85	5.80	0.73	15.48	7.18	4.25	1.27	10.66	12.46	0.83	0.11	0.36	10.43	6.71	0.96	1.30	2.14	0.54
Uruguay	172.31	5.94	13.51	2.75	2.35	30.06	14.66	6.02	1.92	11.66	21.10	2.00	0.33	0.94	7.72	12.12	1.50	1.03	3.13	0.32
Canada	124.98	3.53	6.28	3.75	0.71	31.41	9.17	1.52	1.19	7.20	14.49	1.40	0.25	0.56	2.09	8.01	1.79	0.87	2.88	0.09
United States	125.33	3.46	3.22	5.08	0.95	34.41	9.27	1.52	1.49	6.09	14.35	1.29	0.22	0.70	0.81	8.79	2.06	0.68	3.08	0.12
Antigua and Barbuda	119.17	2.77	8.71	5.59	1.56	7.97	12.42	4.92	2.12	26.25	11.57	1.51	0.33	0.90	1.29	4.68	0.97	1.03	3.01	0.09
The Bahamas	144.73	4.93	8.12	5.55	2.60	13.35	18.36	6.08	2.75	25.76	15.69	2.73	0.51	1.46	1.82	4.26	1.01	0.90	3.68	0.01
Barbados	139.86	4.10	8.34	4.44	1.48	8.20	15.82	6.43	3.35	26.03	18.22	1.80	0.53	1.14	1.39	5.89	0.58	0.64	3.10	0.04
Belize	103.91	2.28	8.63	8.65	1.74	12.23	5.51	7.65	1.99	16.76	8.25	1.47	0.38	0.66	1.33	5.37	0.49	0.93	1.31	0.07
Cuba	138.77	4.74	5.21	4.83	5.02	31.62	9.31	3.80	3.09	15.33	15.41	2.93	0.49	1.14	1.09	5.20	0.58	1.95	1.89	0.11
Dominica	165.77	4.26	15.87	6.32	2.42	13.91	12.53	7.63	2.10	39.69	11.65	2.83	0.51	1.74	1.69	6.21	0.60	0.84	1.65	0.03
Dominican Republic	106.35	1.95	7.13	7.81	1.57	13.10	8.34	5.42	1.17	19.68	9.20	2.23	0.80	1.40	0.98	4.73	0.30	1.36	1.38	0.02
Grenada	143.29	5.69	8.41	5.60	1.94	12.58	13.62	7.19	4.70	24.01	12.97	2.62	0.47	1.02	1.48	6.65	0.68	0.96	3.62	0.03
Guyana	102.16	1.70	6.83	5.23	1.16	7.47	9.77	9.07	2.99	17.92	10.16	1.44	0.28	0.55	1.31	4.24	0.54	0.67	3.28	0.09
Haiti	157.23	3.66	16.22	6.78	3.11	12.94	12.43	13.67	2.87	30.48	11.88	2.48	0.74	1.03	2.40	3.30	0.87	1.17	2.06	0.06
Jamaica	134.65	3.19	9.21	3.91	1.77	19.46	13.54	7.41	3.22	21.61	14.22	1.28	0.50	0.70	1.49	4.31	0.54	0.53	2.80	0.06
Saint Lucia	128.59	3.88	10.19	4.19	2.20	10.93	11.21	6.94	2.28	25.43	9.81	2.63	0.71	1.01	1.07	6.01	0.79	0.67	3.03	0.17
Saint Vincent and the Grenadines	140.27	2.41	9.58	5.65	2.91	9.76	12.46	9.29	3.10	29.32	11.58	3.64	0.57	1.38	1.19	4.97	0.76	1.21	2.50	0.07
Suriname	117.44	1.38	6.38	7.90	1.03	14.44	8.69	7.79	1.28	16.07	14.12	1.49	0.89	0.56	1.17	5.71	0.60	0.64	3.07	0.14
Trinidad and Tobago	105.74	1.45	4.60	3.45	1.14	9.93	11.25	5.59	2.98	20.28	12.32	1.25	0.32	0.61	1.18	5.02	0.34	0.40	3.18	0.05
Bolivia	145.00	2.45	27.10	9.22	1.33	12.09	7.93	9.82	2.53	14.06	10.50	1.47	0.25	1.00	5.32	5.74	1.24	1.40	2.32	0.21
Ecuador	108.95	1.45	17.97	8.02	0.77	8.96	5.87	6.15	2.13	10.84	8.66	1.07	0.17	0.70	3.15	4.50	0.93	1.35	2.10	0.25
Peru	91.83	1.15	14.16	6.48	0.58	9.55	5.41	5.37	1.29	7.88	7.36	0.97	0.14	0.55	3.30	4.34	0.65	0.78	1.79	0.19
Colombia	84.40	1.63	10.68	4.18	1.00	10.34	6.18	3.88	0.96	6.85	8.17	0.97	0.26	0.33	2.31	3.94	0.77	1.08	2.07	0.21
Costa Rica	115.52	1.86	17.24	6.65	1.27	9.53	8.49	3.80	1.53	10.74	13.19	1.19	0.60	0.69	2.25	6.37	1.10	1.41	1.96	0.26
El Salvador	97.64	1.53	13.49	6.86	0.91	9.22	6.61	7.90	2.05	7.30	8.61	1.21	0.52	0.69	3.06	4.56	0.24	0.95	2.07	0.09
Guatemala	100.74	1.70	19.39	16.03	0.82	6.54	4.75	7.60	1.54	9.36	6.58	0.96	0.44	0.53	1.86	3.42	0.38	1.51	1.26	0.23
Honduras	88.06	1.56	7.27	2.56	1.01	8.10	6.63	2.33	4.26	7.05	7.11	1.39	0.69	1.30	3.59	4.23	0.38	1.14	3.05	0.02
Mexico	84.35	1.30	7.28	6.63	1.00	8.66	6.45	4.40	0.88	7.15	7.62	0.92	0.23	0.30	2.29	4.69	0.67	1.22	2.33	0.43

Nicaragua	70.55	0.75	7.96	7.49	0.77	6.26	5.15	6.60	0.63	5.69	6.60	0.65	0.22	0.28	2.05	3.35	0.26	0.68	1.40	0.12
Panama	87.42	1.42	8.74	4.86	1.12	9.60	6.83	4.82	1.47	9.43	9.29	1.11	0.45	0.70	1.48	4.11	0.46	0.69	1.75	0.13
Venezuela	107.37	1.84	9.68	4.90	2.02	17.16	8.57	6.46	1.44	12.16	9.31	1.32	0.37	0.57	1.70	4.82	0.53	1.76	2.23	0.19
Brazil	108.43	4.84	9.27	5.27	2.17	14.43	8.12	4.20	1.13	9.25	10.28	2.29	0.23	1.53	2.34	5.64	1.00	1.28	2.04	0.15
Paraguay	106.03	3.72	8.44	3.41	1.48	14.91	9.04	8.59	1.57	10.39	10.37	1.60	0.17	0.94	2.07	5.42	0.68	1.35	1.95	0.22
Algeria	65.47	1.02	4.56	2.14	1.33	8.66	6.20	2.00	0.35	3.58	6.13	0.74	1.38	0.68	3.63	2.88	0.25	0.36	1.06	0.05
Bahrain	67.40	1.26	3.95	3.12	0.80	13.29	6.45	0.88	0.77	4.29	7.04	0.82	0.23	0.26	0.76	4.03	0.14	0.33	1.84	0.01
Egypt	79.43	1.84	3.74	20.47	0.82	7.39	5.14	0.77	0.54	3.38	5.31	0.64	0.11	0.30	1.12	2.77	0.17	0.21	1.86	0.02
Iran	89.80	4.36	14.91	3.91	1.93	11.28	5.71	1.02	0.47	5.91	8.50	0.71	0.17	0.22	1.13	4.14	0.50	0.45	1.56	0.09
Iraq	56.25	0.67	2.91	3.44	1.25	9.78	5.50	0.67	0.67	2.45	4.55	0.56	0.12	0.27	0.56	2.94	0.12	0.29	1.02	0.06
Jordan	78.03	1.06	4.32	2.76	0.67	12.62	7.52	0.80	0.84	5.15	10.26	1.12	0.46	0.35	1.42	4.05	0.21	0.52	1.33	0.09
Kuwait	49.53	1.07	2.05	4.26	0.54	7.18	3.58	0.41	0.56	3.62	5.65	0.51	0.20	0.23	0.76	3.24	0.11	0.25	0.80	0.01
Lebanon	128.12	1.19	6.00	3.17	1.98	22.63	15.70	1.33	1.23	5.09	13.09	1.13	0.42	0.34	2.32	4.96	0.55	0.32	3.84	0.13
Libya	119.46	1.72	6.41	6.14	2.89	21.33	7.57	1.99	0.95	4.97	13.24	1.18	1.63	0.33	2.81	6.80	0.26	0.51	2.35	0.09
Morocco	76.82	1.31	4.09	1.89	2.38	13.93	9.22	3.00	0.65	3.88	6.72	1.03	1.48	0.46	1.08	2.92	0.40	0.39	1.97	0.07
Palestine	91.71	0.99	5.38	5.86	0.75	14.86	10.22	1.17	1.55	5.04	12.57	0.70	0.32	0.22	0.86	4.76	0.17	0.44	1.82	0.01
Oman	64.82	2.34	6.83	4.56	0.65	6.82	3.88	1.05	0.30	3.87	6.53	1.13	0.29	0.34	0.97	3.32	0.20	0.56	1.01	0.02
Qatar	85.25	2.42	5.15	9.42	1.16	13.16	6.16	0.76	0.54	7.36	9.59	0.89	0.19	0.39	1.07	4.81	0.21	0.45	1.33	0.01
Saudi Arabia	76.92	2.09	4.74	7.01	0.80	9.65	3.77	0.59	0.48	6.67	9.28	1.20	0.92	0.39	1.59	4.18	0.18	0.48	0.95	0.06
Syria	68.98	1.02	4.27	3.85	0.78	9.60	4.76	0.72	0.34	5.26	6.40	0.64	0.13	0.24	0.67	3.25	0.18	0.35	0.90	0.01
Tunisia	79.20	0.89	5.01	1.79	2.24	17.47	6.58	1.19	0.56	3.76	7.88	1.19	1.23	0.46	2.35	2.87	0.32	0.40	1.42	0.06
Turkey	105.38	1.29	9.24	3.92	1.71	31.31	5.07	0.92	0.97	4.79	9.91	0.62	0.44	0.26	1.17	6.11	0.99	0.66	1.79	0.21
United Arab Emirates	154.70	8.89	8.76	6.00	2.84	16.82	6.06	2.08	0.34	4.80	13.07	1.37	0.45	0.58	1.36	6.90	0.44	0.45	1.14	0.09
Yemen	90.88	4.11	19.57	3.16	2.37	10.55	5.51	1.93	0.41	5.31	8.34	0.97	0.40	0.29	1.52	2.23	0.42	0.43	0.98	0.07
Afghanistan	138.97	8.30	33.56	5.95	3.66	12.30	9.15	4.61	0.79	6.01	11.98	1.31	0.68	0.44	2.29	2.75	0.59	0.49	1.54	0.12
Bangladesh	82.69	2.77	6.53	2.36	2.42	21.17	5.40	2.79	0.47	3.28	7.13	4.92	0.71	2.86	1.98	1.87	0.16	0.64	0.98	0.07
Bhutan	80.07	4.56	6.07	4.06	2.42	7.66	5.86	2.58	0.59	4.39	7.11	5.11	0.78	3.59	2.81	3.11	0.22	0.79	1.40	0.08
India	86.44	3.83	7.75	3.34	3.11	7.99	7.15	3.92	0.77	3.61	6.97	6.13	0.90	5.44	2.97	3.02	0.20	0.81	1.84	0.10
Nepal	86.10	5.20	8.14	3.42	3.09	11.20	6.88	3.98	0.66	3.80	6.44	4.94	0.80	3.53	3.13	2.81	0.20	0.83	1.42	0.07
Pakistan	143.77	8.20	5.54	5.92	5.17	13.95	16.59	2.24	3.03	4.78	8.46	16.85	1.08	3.93	4.88	2.67	0.54	0.87	5.91	0.30
Angola	111.47	8.84	7.17	8.49	1.68	11.45	8.76	11.66	1.10	10.13	9.67	2.20	0.49	0.59	1.47	4.19	0.61	1.31	1.79	0.08
Central African Republic	123.48	10.23	10.88	8.25	2.12	11.05	11.99	17.64	1.31	8.51	9.71	2.61	0.65	0.56	1.46	3.24	0.62	1.16	1.78	0.14
Congo	132.53	9.42	7.80	8.30	1.79	12.59	13.61	14.50	1.48	11.78	12.14	2.47	0.61	0.65	1.58	5.19	0.70	1.49	2.63	0.08
Democratic Republic of the Congo	99.94	6.94	7.59	7.65	1.43	8.61	8.80	13.45	1.12	8.91	8.09	1.93	0.47	0.42	1.28	2.79	0.58	1.17	1.66	0.09
Equatorial Guinea	111.12	10.50	4.84	9.88	1.24	11.11	10.25	9.06	1.14	9.37	10.33	2.16	0.39	0.66	1.41	5.99	0.58	1.10	2.29	0.05
Gabon	120.27	8.95	6.07	8.48	1.77	13.78	10.76	8.52	1.07	10.43	11.96	2.63	0.52	0.74	1.40	5.91	0.64	1.38	2.18	0.08
Burundi	108.68	9.57	7.92	5.93	1.75	7.54	7.81	12.08	1.29	10.52	8.35	2.47	1.12	0.92	1.09	2.66	0.69	1.22	2.02	0.06
Comoros	123.33	8.64	6.61	6.45	1.36	8.50	11.21	12.75	1.65	12.39	11.43	2.20	1.07	0.75	1.17	3.77	0.82	1.50	3.32	0.05
Djibouti	130.07	8.75	6.47	7.67	1.57	10.59	9.66	10.40	1.52	14.32	13.59	2.39	1.17	0.87	1.15	4.57	0.78	1.74	3.02	0.06
Eritrea	158.72	10.38	9.57	8.80	1.87	9.20	14.71	19.85	2.46	11.75	13.86	2.99	1.59	1.09	1.64	4.29	1.28	1.47	4.33	0.08
Ethiopia	101.03	5.34	5.79	4.27	0.88	8.63	8.62	8.32	0.84	6.20	11.45	2.67	0.92	0.43	1.82	3.11	0.38	1.51	2.89	0.04
Kenya	98.37	8.43	9.55	5.86	1.65	6.43	7.40	6.43	0.64	8.88	8.68	2.03	1.47	0.93	1.83	3.37	0.45	1.48	1.75	0.06
Madagascar	104.18	7.81	7.00	5.49	1.32	7.28	8.26	12.59	1.35	9.54	9.58	1.97	1.02	0.72	1.00	2.54	0.77	1.18	2.25	0.05
Malawi	120.64	23.85	3.58	5.47	0.73	5.71	6.20	11.28	1.06	6.24	5.86	1.95	0.48	0.23	0.89	2.77	1.70	1.32	1.43	0.17
Mauritius	85.24	2.26	6.55	3.84	1.06	11.61	10.94	2.82	1.67	5.56	11.05	2.44	0.72	0.58	1.11	4.57	0.20	0.41	2.45	0.05
Mozambique	138.99	8.02	7.22	30.51	1.91	10.23	10.61	14.93	1.67	4.35	14.07	2.55	0.13	0.70	1.22	3.07	0.84	1.39	2.68	0.01
Rwanda	106.17	7.18	5.95	6.38	1.32	7.40	8.81	10.73	1.30	11.19	7.78	2.16	0.91	0.80	1.15	3.28	0.75	1.29	2.71	0.03
Seychelles	154.90	5.17	5.54	6.09	4.62	15.27	10.96	8.45	1.19	22.28	21.87	6.63	1.69	3.74	1.32	6.28	0.64	0.61	4.08	0.15
Somalia	136.62	11.17	9.24	8.04	1.73	8.28	9.07	17.63	1.80	12.88	12.81	2.36	1.31	0.79	1.36	3.16	0.86	1.54	2.72	0.07
Tanzania	107.66	4.68	6.13	6.55	1.33	7.38	8.08	10.36	1.35	12.40	9.72	1.38	0.97	0.82	1.14	3.44	0.76	1.42	2.65	0.01
Uganda	137.54	14.12	5.51	8.96	1.27	6.71	9.59	9.27	1.75	21.05	9.04	2.70	1.56	1.48	0.66	3.41	1.01	1.19	2.53	0.07
Zambia	127.90	6.33	7.31	9.32	1.67	8.61	9.83	12.31	1.69	12.81	12.03	2.05	1.25	1.04	1.24	4.40	0.83	1.58	3.07	0.05
Botswana	114.28	8.16	4.99	7.23	1.33	12.20	10.40	9.77	1.43	14.56	9.08	2.47	0.48	0.56	1.07	5.58	1.23	2.00	2.49	0.08
Lesotho	153.39	14.42	10.17	10.43	2.62	16.16	13.03	17.54	2.18	12.32	10.25	3.76	0.83	0.87	1.46	5.81	1.51	2.14	3.11	0.20
Namibia	107.07	2.99	3.46	3.25	2.53	7.46	13.83	7.59	1.15	13.27	7.77	5.14	0.49	1.32	0.84	3.64	2.65	2.31	1.81	0.13
South Africa	111.87	10.04	4.63	5.24	1.47	16.39	9.51	9.33	1.20	11.82	9.15	2.37	0.41	0.54	1.19	5.45	1.06	2.31	2.28	0.13
Swaziland	150.33	15.51	7.80	13.51	2.23	15.44	11.57	13.77	1.79	12.34	12.18	3.56	0.74	0.88	1.34	6.92	1.47	2.06	3.05	0.14
Zimbabwe	165.78	14.45	11.14	15.51	2.21	10.65	10.50	14.90	2.21	19.33	11.08	1.96	0.67	0.44	1.42	6.16	1.46	2.40	3.18	0.11

Benin	115.13	6.57	12.45	14.47	1.30	9.44	8.18	10.31	1.45	14.33	7.61	1.47	0.34	0.48	1.40	4.57	0.35	0.53	1.45	0.04
Burkina Faso	123.76	6.78	13.71	15.69	1.49	7.91	10.83	12.05	1.47	12.11	13.05	1.43	0.37	0.55	1.46	3.78	0.36	0.51	1.36	0.07
Cameroon	129.02	7.12	12.63	15.99	1.65	11.25	9.05	9.55	1.47	16.66	9.08	1.83	0.38	0.65	1.49	5.94	0.39	0.60	1.73	0.05
Cape Verde	119.86	10.58	17.14	10.08	0.93	11.56	6.70	5.86	1.75	15.26	7.40	2.89	0.25	0.90	0.89	7.27	0.25	0.38	1.36	0.03
Chad	119.25	5.54	14.62	15.11	1.59	11.95	6.76	11.60	1.24	15.67	8.10	1.60	0.39	0.52	1.49	3.38	0.36	0.57	1.10	0.06
Cote d'Ivoire	102.18	1.33	5.88	8.91	1.36	6.73	10.01	4.47	0.95	27.88	5.67	1.61	0.68	0.66	1.49	2.69	0.41	0.65	1.83	0.06
The Gambia	110.69	2.18	6.24	39.20	0.74	7.96	6.69	7.32	0.99	5.94	6.26	1.47	0.49	0.41	1.53	3.08	0.31	0.64	1.38	0.09
Ghana	114.74	4.04	8.36	16.34	1.54	7.25	9.93	9.05	2.27	12.84	8.66	1.60	0.14	0.84	1.31	8.30	0.23	0.42	1.80	0.01
Guinea	144.32	2.39	15.29	36.40	1.66	9.90	9.00	15.37	1.62	10.70	7.53	4.19	0.65	1.32	1.55	2.98	1.00	0.61	1.72	0.19
Guinea-Bissau	138.85	8.32	16.21	18.13	1.74	10.77	10.65	14.32	1.84	13.08	10.56	1.89	0.47	0.62	1.63	4.98	0.40	0.54	1.74	0.06
Liberia	110.67	6.04	11.68	15.49	1.24	7.81	7.98	9.36	1.25	15.12	8.73	1.36	0.32	0.43	1.20	3.87	0.32	0.54	1.26	0.04
Mali	118.22	2.42	15.40	32.22	0.87	7.09	7.36	6.61	0.69	7.16	7.31	1.32	0.43	0.25	1.12	3.05	0.72	0.59	0.79	0.09
Mauritania	117.70	5.27	10.50	12.80	1.14	9.80	8.92	9.03	1.47	18.83	8.72	1.55	0.32	0.45	1.35	5.30	0.43	0.63	1.71	0.04
Niger	111.06	4.50	13.00	22.33	1.19	8.30	5.85	11.57	1.22	13.56	6.49	1.29	0.31	0.39	1.34	2.83	0.34	0.49	1.07	0.05
Nigeria	112.83	3.75	4.49	3.84	1.16	8.41	16.65	7.30	0.60	28.69	8.58	0.86	0.51	0.21	1.29	4.40	0.56	0.51	1.83	0.03
Sao Tome and Principe	122.52	4.41	17.10	2.03	1.02	15.48	9.26	11.19	2.35	8.29	12.75	0.90	0.15	0.58	2.19	2.36	0.21	0.22	2.20	0.03
Senegal	125.60	5.21	13.06	14.91	1.47	11.55	9.09	10.34	1.41	19.47	8.01	1.59	0.36	0.49	1.52	4.55	0.42	0.66	1.53	0.05
Sierra Leone	116.71	5.23	13.32	13.99	1.48	10.29	8.99	10.91	1.38	14.31	9.01	1.50	0.36	0.55	1.36	3.86	0.34	0.55	1.38	0.05
Togo	106.65	5.04	12.26	11.74	1.30	9.14	9.18	10.07	1.42	12.19	7.35	1.51	0.35	0.48	1.29	4.03	0.35	0.49	1.52	0.06
American Samoa	146.91	1.41	12.90	10.10	1.19	25.33	14.39	3.54	5.20	18.80	12.66	1.78	1.52	2.06	1.05	5.49	0.90	1.32	6.71	0.04
Bermuda	123.07	4.23	4.95	3.30	1.93	21.19	9.08	1.90	1.50	15.28	16.14	2.12	0.40	1.00	0.80	8.23	1.63	1.11	3.02	0.01
Greenland	216.48	11.43	11.71	6.20	1.73	76.18	7.84	4.01	0.45	5.61	26.50	3.29	4.78	2.69	2.52	17.36	1.00	0.74	5.12	0.01
Guam	132.18	2.55	4.95	11.39	1.39	37.32	9.64	2.86	2.27	7.57	13.28	2.71	3.31	1.43	0.85	6.20	0.66	0.92	3.08	0.19
Northern Mariana Islands	123.65	1.73	6.59	7.50	1.82	31.57	9.37	4.58	2.96	9.33	9.62	6.51	1.62	2.16	0.77	6.04	0.95	1.38	1.79	0.10
Puerto Rico	89.33	2.22	4.26	5.46	1.06	10.79	8.41	2.19	1.26	8.72	12.93	1.34	0.33	0.62	0.71	5.10	0.54	0.85	2.09	0.12
Virgin Islands, U.S.	168.12	4.02	8.56	4.55	2.37	21.56	14.45	4.45	1.97	30.08	23.33	2.36	0.79	1.48	1.64	8.29	1.80	0.89	4.81	0.00
South Sudan	126.56	12.27	8.44	8.42	1.66	10.51	7.19	12.81	1.41	12.77	10.84	2.08	1.23	0.88	1.22	3.40	0.74	1.51	2.11	0.06
Sudan	81.08	4.72	14.91	3.60	1.99	8.34	4.61	1.37	0.34	5.66	7.13	0.96	0.33	0.27	1.31	2.46	0.38	0.41	0.84	0.06

Age-standardized Death in 1990

	Total	Esophageal cancer	Stomach cancer	Liver cancer	Larynx cancer	Tracheal, bronchus, and lung cancer	Breast cancer	Cervical cancer	Uterine cancer	Prostate cancer	Colon and rectum cancer	Lip and oral cavity cancer	Nasopharynx cancer	Other pharynx cancer	Gallbladder and biliary tract cancer	Pancreatic cancer	Malignant skin melanoma	Non-melanoma skin cancer	Ovarian cancer	Testicular cancer
China	162.96	20.53	34.19	26.72	1.34	28.30	4.38	2.73	1.34	2.97	9.33	0.84	2.74	0.32	1.48	3.04	0.31	0.70	0.81	0.08
North Korea	132.69	9.04	21.32	27.27	1.07	23.60	5.56	3.54	1.15	3.59	8.90	1.04	1.45	0.38	1.91	2.98	0.32	0.56	1.19	0.06
Taiwan	126.57	5.13	16.30	21.96	1.19	22.69	4.09	5.65	0.62	3.44	12.17	2.13	4.04	1.04	2.21	3.44	0.33	1.07	0.96	0.04
Cambodia	137.25	4.67	20.29	9.59	2.64	24.24	7.94	9.97	2.17	5.19	11.84	2.65	2.34	1.00	2.46	2.76	0.32	0.87	1.89	0.08
Indonesia	93.07	0.96	11.09	6.84	1.58	16.07	8.02	5.33	1.54	3.99	7.11	1.41	1.65	0.72	1.70	2.50	0.24	0.65	1.69	0.01
Laos	140.45	4.83	19.28	11.99	2.79	25.98	8.00	8.96	2.05	4.58	11.45	2.78	2.51	1.13	2.39	2.85	0.35	0.79	1.85	0.10
Malaysia	122.17	3.09	12.04	5.42	1.83	19.84	9.45	7.11	1.43	4.96	18.23	3.01	4.99	0.92	1.58	2.37	0.38	0.83	1.69	0.16
Maldives	92.72	3.70	7.96	6.63	2.02	13.83	5.27	6.25	0.54	4.68	6.80	3.84	0.91	0.49	1.41	2.58	0.55	0.63	1.86	0.01
Myanmar	169.39	12.86	23.05	11.75	2.98	22.87	14.80	11.82	2.71	5.27	13.44	2.86	2.64	1.13	2.79	3.60	0.40	0.86	2.91	0.10
Philippines	93.40	1.46	7.03	12.25	1.13	16.11	7.05	2.01	1.70	4.20	5.60	3.00	1.74	0.66	1.18	2.62	0.28	0.47	1.95	0.10
Sri Lanka	78.39	4.70	8.58	1.93	0.83	7.55	4.53	1.87	0.68	4.34	4.88	4.32	1.38	2.23	4.37	2.00	0.20	1.94	1.14	0.03
Thailand	137.28	3.20	9.14	23.33	2.40	29.34	5.92	7.32	0.73	3.40	10.07	4.71	1.57	0.89	6.31	3.40	0.29	1.76	1.86	0.02
Timor-Leste	100.66	3.24	13.57	8.14	1.79	16.22	5.01	6.90	1.60	5.00	6.87	2.05	1.79	0.69	2.02	2.15	0.27	0.80	1.41	0.07
Vietnam	134.79	3.83	18.34	14.60	1.53	35.11	6.30	5.30	0.83	3.21	12.15	3.80	1.72	1.62	1.34	1.88	0.19	0.74	1.61	0.10
Fiji	100.11	1.99	6.76	7.32	0.99	8.45	14.09	12.13	2.42	8.71	8.02	2.58	0.50	0.85	1.29	2.90	0.46	0.74	0.73	0.48
Kiribati	139.90	3.85	15.54	11.68	1.06	16.28	10.81	31.25	3.46	5.89	9.01	5.91	0.66	1.20	1.54	2.25	0.43	0.44	0.74	1.23
Marshall Islands	144.37	3.37	19.19	13.18	2.00	25.44	10.08	10.07	3.78	7.93	12.63	2.30	2.13	1.26	1.54	3.73	0.83	1.05	1.55	0.26
Federated States of Micronesia	138.75	2.51	15.85	12.42	1.84	25.02	10.44	10.16	3.92	8.71	11.08	2.39	1.93	1.25	1.62	3.55	0.76	1.14	1.65	0.20
Papua New Guinea	131.80	2.34	18.91	9.33	2.03	25.30	9.49	12.47	3.27	6.76	8.29	2.30	2.25	1.28	1.54	2.37	0.74	0.99	1.24	0.29
Samoa	91.56	1.56	12.10	4.81	0.72	8.04	7.32	5.18	2.59	6.46	8.80	1.59	1.48	0.27	1.22	2.98	1.50	1.12	1.95	0.36
Solomon Islands	122.77	2.44	17.04	10.76	1.85	23.58	7.19	10.18	2.80	8.81	7.18	1.89	1.96	1.00	1.42	2.46	0.75	1.06	1.08	0.23
Tonga	170.72	2.42	18.54	23.91	1.19	30.01	17.26	12.42	2.53	15.33	7.18	1.62	1.18	0.78	1.07	3.97	0.65	2.78	1.31	7.26
Vanuatu	157.31	2.19	16.48	21.62	2.06	25.72	9.78	9.74	3.06	13.48	10.48	2.87	2.30	1.16	1.93	3.26	1.27	1.63	1.38	0.23
Armenia	143.79	2.39	20.30	9.57	3.80	28.68	11.78	4.89	1.47	2.73	11.18	1.28	0.24	0.54	0.86	7.37	0.39	0.27	2.73	0.27
Azerbaijan	112.36	8.46	23.63	7.34	2.42	18.75	6.38	2.56	1.22	3.23	7.85	0.91	0.15	0.32	1.44	3.71	0.49	0.64	1.40	0.04
Georgia	102.23	1.65	14.91	5.16	3.23	19.63	12.13	4.83	1.56	2.64	8.16	1.68	0.40	0.69	1.25	3.06	1.35	0.46	1.65	0.23
Kazakhstan	165.16	19.14	29.60	9.61	3.11	35.78	7.43	5.60	2.38	2.82	11.91	2.57	0.42	1.34	1.50	1.41	1.21	0.32	2.54	0.48
Kyrgyzstan	125.46	7.88	28.33	6.84	2.03	22.87	7.47	5.97	1.34	2.72	9.89	2.59	0.35	0.67	1.37	4.04	0.74	1.12	2.26	0.20
Mongolia	260.37	28.65	62.84	61.12	1.02	38.22	4.31	9.34	1.20	2.83	7.73	4.43	0.25	0.80	3.12	3.03	0.16	0.39	1.22	0.00
Tajikistan	100.21	10.42	23.75	5.51	1.14	12.69	4.40	2.76	0.80	3.39	6.43	0.89	0.53	0.56	0.70	3.41	0.38	0.49	1.54	0.00
Turkmenistan	117.99	32.36	19.68	3.82	2.23	13.30	5.11	3.63	1.13	3.00	6.21	2.24	0.30	1.13	2.56	1.50	0.78	0.45	1.73	0.32
Uzbekistan	93.65	15.53	18.00	4.56	1.65	14.04	5.07	3.15	0.93	2.71	5.87	1.30	0.35	0.60	0.59	1.95	0.34	0.51	0.79	0.15
Albania	106.47	2.03	14.07	10.11	3.09	24.78	4.00	1.69	0.94	6.06	6.36	1.81	0.58	0.93	1.09	3.03	0.79	1.18	0.87	0.29
Bosnia and Herzegovina	130.75	2.61	12.72	5.44	3.96	33.95	6.42	3.38	1.16	5.02	13.38	1.86	0.11	1.20	5.89	7.01	1.27	0.33	2.20	0.12
Bulgaria	124.05	2.25	17.37	6.34	2.45	23.63	8.91	3.58	2.02	5.38	16.65	1.54	0.21	0.76	1.59	5.93	0.96	0.88	2.53	0.56
Croatia	174.20	3.89	20.96	3.28	4.08	36.68	12.17	4.43	1.94	7.54	21.98	4.09	0.43	2.42	4.12	8.10	1.93	2.02	4.29	0.25
Czech Republic	206.60	3.07	17.86	5.76	2.56	45.42	13.20	5.12	3.05	7.18	33.41	2.75	0.40	1.28	8.32	11.72	2.28	1.47	5.02	0.52
Hungary	211.96	4.05	19.91	6.72	4.27	45.90	15.09	4.88	3.04	8.11	29.93	4.98	0.49	2.63	7.31	9.88	2.14	1.28	4.52	0.59
Macedonia	125.55	1.34	19.11	7.28	3.19	23.00	9.47	2.94	1.53	5.32	12.06	1.49	0.29	0.62	2.33	5.58	2.75	0.76	2.22	0.48
Montenegro	138.99	2.43	7.61	5.58	4.53	41.14	10.78	3.21	1.28	7.04	13.70	2.03	0.15	0.77	2.46	7.01	1.37	0.99	2.46	0.32
Poland	173.32	3.75	17.59	3.73	3.98	40.65	10.22	6.13	1.92	5.58	21.53	2.32	0.34	1.26	5.01	9.50	1.66	1.66	4.48	0.39
Romania	120.92	1.59	14.15	3.77	2.93	23.02	8.76	6.55	1.69	4.48	12.18	2.10	0.30	1.20	2.60	6.02	1.01	1.71	2.85	0.33
Serbia	169.39	2.61	12.68	6.34	3.86	39.11	12.65	6.24	1.74	7.38	21.94	3.02	0.39	1.68	3.88	7.64	2.22	1.75	3.33	0.41
Slovakia	171.85	3.72	16.81	4.72	3.78	36.83	11.04	3.79	3.28	6.36	22.92	5.58	0.50	2.17	4.83	7.94	1.92	1.32	3.77	0.41
Slovenia	168.42	4.01	20.08	3.73	2.77	33.86	12.59	3.78	2.21	7.78	21.90	3.42	0.37	2.54	5.21	8.66	2.35	0.68	4.66	0.27
Belarus	142.27	2.59	30.31	2.51	3.43	28.68	8.63	4.19	1.52	3.37	14.53	4.03	0.26	0.86	1.48	5.56	1.10	0.85	4.23	0.16
Estonia	158.42	2.90	25.56	3.05	2.53	36.12	10.87	5.44	1.81	4.59	16.91	2.64	0.48	1.14	2.16	8.63	1.77	1.05	4.91	0.23
Latvia	150.44	2.69	23.87	2.74	2.80	32.61	10.64	4.10	2.06	4.36	16.60	2.65	0.46	0.94	1.61	8.95	1.38	0.80	5.57	0.23
Lithuania	151.00	2.75	24.10	2.30	3.01	31.74	9.69	5.59	1.72	4.78	15.15	2.94	0.41	0.92	2.22	8.14	1.29	0.75	6.59	0.18

Moldova	128.16	2.24	17.70	2.98	2.97	25.19	9.73	5.33	1.58	3.06	15.40	2.88	0.50	1.71	1.66	6.29	1.12	1.10	3.03	0.14
Russian Federation	149.65	4.48	27.92	4.50	3.13	31.33	8.94	3.70	2.92	2.75	15.64	2.42	0.31	1.20	1.68	7.32	1.34	0.82	3.83	0.19
Ukraine	141.87	2.97	23.42	2.15	3.42	31.91	10.88	5.23	1.63	3.03	16.42	2.37	0.33	1.27	1.34	4.95	1.51	0.72	3.04	0.18
Brunei	175.27	3.72	22.80	13.92	2.67	38.09	7.61	7.58	1.56	5.18	20.57	4.08	2.88	1.64	4.05	4.69	0.49	1.05	3.19	0.18
Japan	135.53	4.47	29.94	14.02	0.59	22.11	4.15	2.00	1.12	2.99	15.86	1.01	0.25	0.43	7.59	8.14	0.23	0.35	2.02	0.09
South Korea	164.44	5.12	47.08	29.08	2.28	20.60	4.05	2.97	1.76	2.39	9.85	0.98	0.41	0.35	9.55	6.78	0.22	1.26	0.86	0.03
Singapore	140.36	3.73	17.67	13.10	1.32	36.49	7.59	3.53	1.05	3.38	20.26	1.27	5.44	0.51	1.66	3.65	0.26	0.54	2.56	0.06
Australia	154.95	3.96	8.16	1.98	1.24	30.16	12.54	2.32	1.48	10.03	21.98	2.52	0.77	1.25	2.50	6.95	4.42	1.56	3.43	0.15
New Zealand	166.77	4.46	9.86	2.30	1.03	32.83	16.33	3.03	2.03	10.14	28.23	1.64	0.66	0.86	2.12	6.83	4.57	1.23	4.23	0.28
Andorra	145.91	3.54	9.38	2.28	1.07	33.51	10.98	1.46	1.02	10.58	18.13	1.70	0.50	0.81	2.10	8.07	1.88	0.83	3.64	0.20
Austria	158.39	2.18	15.31	4.62	1.68	27.55	14.55	3.79	1.91	7.76	21.83	1.97	0.43	0.98	4.40	9.18	2.13	0.83	5.38	0.15
Belgium	183.49	3.39	12.25	3.12	2.54	45.68	18.19	2.71	1.46	10.95	22.37	1.81	0.83	1.06	2.81	8.52	1.73	0.82	5.28	0.15
Cyprus	119.96	1.09	8.30	3.36	1.40	20.21	12.25	2.00	1.72	9.35	13.09	1.21	0.31	0.26	2.68	4.77	1.34	1.32	3.22	0.12
Denmark	176.63	4.02	8.79	2.91	1.68	42.32	19.07	4.18	1.72	10.39	16.90	1.66	0.64	1.25	2.60	8.85	2.74	0.81	5.93	0.30
Finland	140.10	2.80	12.84	2.96	0.74	27.69	11.30	1.77	1.82	8.12	13.46	1.21	0.36	0.58	3.55	9.79	1.98	0.59	4.55	0.13
France	172.19	7.04	10.91	6.55	3.99	30.35	14.58	2.79	1.68	10.43	20.49	3.72	1.16	3.25	3.02	7.40	1.70	0.86	4.35	0.22
Germany	165.14	3.10	14.45	3.31	1.59	30.84	15.62	3.55	1.75	7.75	23.85	2.23	0.64	1.49	5.39	8.71	1.90	0.72	5.61	0.33
Greece	137.69	1.66	11.11	12.99	2.22	34.32	10.02	2.06	0.96	7.06	9.83	0.94	0.61	0.31	1.59	6.77	0.81	0.96	2.41	0.18
Iceland	150.70	4.44	13.20	2.38	0.80	32.40	12.26	2.16	1.21	11.56	13.91	1.40	0.52	0.50	2.18	8.88	1.54	0.33	4.76	0.10
Ireland	174.18	6.71	13.11	2.70	1.52	37.30	17.03	2.36	1.39	10.52	23.62	2.07	0.61	0.74	2.40	9.48	1.83	1.69	5.21	0.21
Israel	132.81	1.68	10.66	2.93	0.95	20.95	15.09	1.82	1.03	7.01	17.89	0.76	0.86	0.39	3.03	8.68	2.55	0.95	4.14	0.08
Italy	166.50	2.78	19.25	9.73	2.78	35.00	13.63	1.30	0.63	6.45	17.51	2.14	0.62	1.05	3.76	8.29	1.62	0.75	3.41	0.16
Luxembourg	175.57	4.16	12.23	4.48	2.33	37.91	16.48	2.41	2.42	9.19	22.76	2.49	0.95	1.61	2.80	8.86	2.19	0.90	5.74	0.26
Malta	137.49	2.62	11.78	2.39	1.89	24.62	16.76	1.89	1.79	6.38	15.56	1.69	1.27	0.61	1.93	8.12	1.17	0.81	4.64	0.22
Netherlands	176.42	3.94	13.27	1.58	1.30	45.55	17.51	1.93	1.50	9.94	21.40	1.27	0.62	0.67	3.32	8.66	2.04	0.72	5.25	0.17
Norway	146.15	2.35	11.08	1.64	0.75	23.88	12.28	2.65	1.66	12.44	21.94	1.50	0.43	0.66	1.95	8.41	3.29	0.62	4.88	0.20
Portugal	153.22	4.40	26.26	4.20	3.19	18.76	13.36	3.35	1.94	9.92	19.63	2.45	0.68	0.85	2.68	6.84	0.93	1.32	2.42	0.15
Spain	144.20	3.35	14.57	7.07	3.84	27.48	11.61	2.01	1.48	7.92	15.96	2.30	0.92	1.09	3.02	6.26	1.14	1.39	2.74	0.12
Sweden	134.87	2.47	8.93	3.99	0.56	19.41	12.07	2.12	1.45	11.41	17.10	1.19	0.28	0.60	3.37	8.76	2.94	0.55	4.85	0.14
Switzerland	135.99	3.83	9.08	4.30	1.28	28.99	16.19	2.88	1.14	10.77	9.07	1.69	0.65	0.88	2.51	8.18	2.06	0.70	3.93	0.33
United Kingdom	178.17	6.38	12.62	1.89	1.07	43.64	19.06	3.25	1.30	9.23	21.71	1.43	0.77	0.75	2.15	8.25	2.01	0.91	5.68	0.22
Argentina	161.42	7.04	15.94	4.44	2.83	25.91	13.56	6.57	2.23	8.70	16.63	1.49	0.36	0.91	8.82	8.34	0.79	1.10	2.76	0.46
Chile	163.97	7.63	32.71	6.45	1.35	17.49	8.60	9.94	1.30	8.93	10.72	1.04	0.16	0.55	18.43	6.28	0.91	1.03	2.47	0.84
Uruguay	199.19	9.21	20.83	1.97	4.23	37.10	17.19	7.44	2.54	11.23	21.65	2.26	0.48	1.41	9.97	9.56	0.96	1.20	2.63	0.52
Canada	166.82	3.63	10.30	2.40	1.37	44.09	14.69	2.20	1.57	11.23	19.94	2.14	0.37	0.68	3.16	9.02	1.73	0.79	3.91	0.13
United States	158.20	3.48	5.51	2.86	1.41	46.95	14.20	1.94	1.51	8.74	20.61	1.79	0.33	0.83	1.25	8.38	2.07	0.67	3.93	0.14
Antigua and Barbuda	120.81	3.29	14.37	6.61	1.79	8.56	10.65	6.24	1.54	23.73	10.24	1.82	0.29	1.42	3.73	1.53	0.96	0.24	0.69	0.01
The Bahamas	149.09	6.07	12.59	6.66	2.84	15.58	16.26	7.59	2.42	22.11	14.92	3.40	0.45	2.14	4.53	1.73	1.02	0.87	1.17	0.01
Barbados	144.91	5.56	15.26	6.00	1.55	9.16	14.97	9.59	2.96	21.01	15.67	2.15	0.43	1.70	4.27	1.36	0.56	0.62	0.72	0.01
Belize	94.09	1.90	12.12	7.61	1.44	10.22	4.57	10.19	2.08	12.59	6.52	1.28	0.27	0.65	3.15	1.68	0.35	0.74	0.42	0.01
Cuba	139.54	3.70	7.57	6.18	4.16	31.69	9.25	5.55	2.43	13.87	14.76	2.70	0.40	1.41	3.61	1.72	0.53	1.90	0.57	0.02
Dominica	149.04	4.12	21.92	6.83	1.98	12.33	11.61	10.16	1.75	25.26	9.44	2.66	0.34	1.90	4.63	1.53	0.48	0.70	0.45	0.01
Dominican Republic	81.12	1.59	7.30	4.50	1.77	9.74	4.93	5.52	2.00	11.76	6.59	2.18	0.37	1.06	2.01	1.47	0.28	0.95	0.49	0.02
Grenada	158.43	6.45	14.11	8.45	1.92	12.37	12.43	12.77	4.24	21.39	12.84	2.83	0.60	1.60	5.35	1.52	0.92	0.21	0.92	0.01
Guyana	99.53	1.85	10.67	5.63	1.20	6.66	7.58	10.68	2.30	16.82	9.39	1.54	0.22	0.69	3.38	1.43	0.46	0.21	0.76	0.02
Haiti	170.84	4.69	24.38	7.94	3.86	16.49	10.56	20.79	2.98	23.06	10.64	2.59	0.76	1.38	3.93	1.84	0.84	0.80	1.04	0.04
Jamaica	109.51	3.11	13.85	4.10	1.23	14.46	10.15	8.95	1.67	13.68	10.59	1.41	0.27	0.71	2.40	1.43	0.41	0.40	0.59	0.01
Saint Lucia	149.42	4.94	17.99	6.12	2.56	12.32	12.93	11.71	2.37	23.76	10.37	3.36	0.64	1.56	3.67	1.59	0.86	0.51	0.93	0.03
Saint Vincent and the Grenadines	132.28	2.14	13.91	5.81	2.31	8.82	12.73	13.89	3.05	18.69	10.48	3.56	0.47	1.58	3.39	1.55	0.86	0.25	0.77	0.01
Suriname	106.42	1.39	9.36	7.99	0.94	12.55	7.42	9.98	1.05	11.78	11.23	1.50	0.66	0.69	3.25	1.33	0.52	0.43	0.73	0.02
Trinidad and Tobago	126.43	2.42	10.90	6.81	1.63	11.25	12.64	8.95	2.75	19.54	13.87	1.79	0.28	1.01	3.82	1.37	0.41	0.45	0.56	0.01
Bolivia	170.92	3.22	45.13	9.11	2.15	12.90	7.35	17.67	3.35	11.95	9.79	1.63	0.33	1.55	7.58	3.42	1.23	1.12	1.37	0.16
Ecuador	114.45	2.38	28.96	7.73	1.16	7.99	4.66	8.35	3.35	9.98	6.82	1.08	0.18	1.19	5.37	1.71	0.97	1.09	0.57	0.05
Peru	107.50	1.72	20.81	6.06	1.38	12.86	5.40	7.89	2.11	7.92	6.69	1.37	0.18	1.41	4.60	2.12	0.64	0.84	0.77	0.06
Colombia	124.10	3.72	24.38	7.15	2.33	13.97	7.48	6.99	1.67	8.41	8.49	1.66	0.54	0.73	4.64	5.00	0.77	1.53	2.19	0.19
Costa Rica	130.26	2.83	34.37	5.76	1.86	11.37	7.38	7.55	1.31	8.63	9.39	1.50	0.60	0.83	4.78	3.43	0.87	1.70	1.11	0.07
El Salvador	80.89	1.34	13.86	4.14	0.95	6.80	4.43	7.48	2.46	5.88	5.03	1.52	0.55	1.13	3.53	1.71	0.23	0.55	0.70	0.02
Guatemala	90.25	1.44	22.67	8.48	1.33	7.70	3.92	5.77	2.03	6.22	4.68	1.17	0.36	0.81	5.15	1.40	0.21	1.57	0.37	0.01

Honduras	77.39	1.25	8.38	1.96	1.13	7.62	5.47	3.22	3.20	6.12	5.15	1.21	0.68	1.23	3.17	2.30	0.31	1.03	1.34	0.03
Mexico	100.72	1.78	11.55	7.26	1.87	13.43	5.85	10.49	1.07	7.25	5.79	1.09	0.32	0.42	4.34	4.96	0.50	1.41	1.82	0.31
Nicaragua	81.44	1.10	12.89	6.94	1.38	6.82	4.14	12.57	0.91	6.20	5.52	0.81	0.27	0.45	3.87	1.76	0.29	0.34	0.55	0.02
Panama	99.98	1.63	13.34	4.13	2.15	13.25	6.34	8.73	1.30	8.56	8.73	1.85	0.52	1.02	2.99	2.73	0.50	0.68	1.10	0.03
Venezuela	118.94	2.48	18.23	6.63	2.49	16.95	7.24	9.49	2.25	9.59	8.41	1.51	0.37	0.81	4.09	1.52	0.45	1.91	0.51	0.02
Brazil	120.47	6.01	16.94	5.07	2.65	15.84	8.25	6.72	1.39	9.25	8.75	2.43	0.21	1.64	3.59	5.00	0.87	1.21	1.93	0.14
Paraguay	94.09	3.48	11.37	3.65	1.40	11.21	6.81	10.24	2.30	7.70	6.50	1.69	0.17	0.83	2.77	2.48	0.52	1.24	0.90	0.04
Algeria	71.97	1.15	7.58	1.73	2.05	10.39	5.33	3.33	0.41	3.66	6.07	0.88	1.61	0.71	4.74	1.85	0.25	0.34	0.83	0.08
Bahrain	131.85	3.87	11.22	4.09	2.53	39.57	9.03	1.84	1.01	6.24	9.70	1.68	0.58	0.57	1.32	5.57	0.21	0.62	2.76	0.01
Egypt	69.63	1.58	4.94	13.79	0.89	5.92	4.28	0.97	0.57	3.36	4.39	0.60	0.13	0.27	1.22	1.79	0.16	0.21	1.44	0.02
Iran	86.72	5.28	21.88	3.16	2.68	10.26	4.06	1.40	0.38	4.11	6.93	0.62	0.23	0.22	1.11	2.23	0.68	0.28	0.84	0.05
Iraq	96.62	1.54	7.48	4.70	3.30	17.51	9.83	1.58	0.84	3.43	7.14	1.14	0.56	0.55	1.15	3.64	0.20	0.49	1.22	0.13
Jordan	85.12	1.27	7.55	2.45	1.88	12.59	7.86	1.58	1.08	3.17	9.96	1.14	0.75	0.39	1.81	2.58	0.25	0.51	1.27	0.22
Kuwait	66.53	1.57	4.39	3.72	1.08	10.64	5.57	1.09	0.69	2.88	5.40	0.95	0.43	0.43	1.75	3.19	0.16	0.25	1.52	0.08
Lebanon	145.53	1.44	12.06	3.58	4.17	26.15	13.71	2.57	1.53	5.84	13.72	1.28	0.71	0.44	3.24	3.70	0.57	0.30	3.10	0.14
Libya	110.11	1.38	7.26	5.45	3.27	21.94	4.86	2.31	0.88	4.39	11.39	1.09	1.90	0.35	3.32	4.86	0.23	0.42	1.52	0.08
Morocco	75.75	1.38	5.56	1.73	2.78	13.38	8.25	4.69	0.74	3.44	5.64	1.00	1.76	0.46	1.24	1.93	0.37	0.31	1.51	0.08
Palestine	101.29	1.32	9.25	6.47	1.25	17.41	9.47	1.72	1.69	5.19	13.06	0.79	0.40	0.25	1.00	3.89	0.18	0.44	1.38	0.01
Oman	76.67	2.75	13.85	4.91	1.15	8.20	3.56	2.16	0.40	3.73	6.10	1.44	0.49	0.42	1.39	2.09	0.22	0.61	0.96	0.04
Qatar	129.70	5.31	11.08	14.43	1.35	20.72	8.77	1.51	0.61	6.78	13.80	1.29	0.23	0.41	3.46	6.60	0.36	0.43	1.35	0.01
Saudi Arabia	66.37	2.22	7.64	6.68	1.12	8.08	2.53	0.76	0.40	5.31	5.54	1.13	1.11	0.37	1.66	1.98	0.17	0.39	0.62	0.06
Syria	72.41	1.02	6.37	4.23	1.01	9.48	4.28	1.16	0.44	4.40	5.84	0.66	0.16	0.24	0.86	2.04	0.18	0.29	0.66	0.01
Tunisia	82.84	0.90	7.23	1.72	2.88	17.92	5.65	1.74	0.61	3.88	7.43	1.21	1.67	0.47	2.88	2.00	0.31	0.37	1.09	0.08
Turkey	145.89	2.25	20.67	4.37	3.43	38.38	5.89	2.17	1.59	5.18	11.88	1.43	0.81	0.41	2.05	5.97	1.25	0.90	2.19	0.35
United Arab Emirates	138.85	6.50	10.17	4.71	2.79	14.85	5.74	3.51	0.41	3.96	11.40	1.33	0.50	0.69	1.75	4.16	0.42	0.34	1.05	0.05
Yemen	87.23	5.02	22.01	3.29	2.50	10.74	4.07	2.64	0.45	3.22	6.58	0.93	0.52	0.34	1.80	1.36	0.38	0.29	0.65	0.07
Afghanistan	141.89	9.74	36.92	6.73	4.01	13.58	7.62	4.98	0.73	5.83	10.15	1.38	0.93	0.49	2.39	2.24	0.58	0.42	1.04	0.09
Bangladesh	107.36	4.32	12.93	2.51	5.20	22.36	4.08	7.55	0.89	4.43	9.30	3.89	1.30	3.77	2.66	1.63	0.21	0.93	0.89	0.02
Bhutan	96.64	7.78	10.44	3.38	4.26	8.38	6.76	7.81	1.07	3.69	6.57	6.86	1.14	3.67	2.81	1.93	0.22	0.76	1.14	0.18
India	80.91	4.29	11.07	2.13	4.26	7.29	4.97	5.24	0.85	3.19	5.53	5.84	0.98	4.70	2.21	1.59	0.17	0.75	1.11	0.18
Nepal	92.94	6.36	11.65	2.22	4.57	11.59	6.17	8.40	0.93	3.24	5.70	5.94	1.07	3.23	2.79	1.56	0.20	0.82	0.94	0.19
Pakistan	115.93	7.36	5.77	4.56	5.33	12.41	10.45	2.54	2.46	3.82	5.99	13.46	1.08	3.06	4.24	1.54	0.42	0.78	3.40	0.34
Angola	137.37	12.94	14.01	13.93	2.40	14.74	7.20	17.43	1.28	9.91	9.64	2.22	0.72	0.63	1.71	3.25	0.64	1.37	1.38	0.13
Central African Republic	142.89	14.16	15.46	12.86	2.64	12.90	10.10	21.62	1.46	8.56	9.73	2.71	0.81	0.65	1.73	3.20	0.66	1.20	1.63	0.14
Congo	151.75	14.80	13.63	11.97	2.56	15.18	11.80	20.68	1.77	8.76	12.01	2.60	0.82	0.80	1.92	4.30	0.69	1.23	2.24	0.13
Democratic Republic of the Congo	114.04	9.81	10.45	11.51	1.80	10.37	7.13	14.74	1.16	9.94	8.01	1.95	0.57	0.46	1.42	2.82	0.57	1.27	1.46	0.09
Equatorial Guinea	141.12	15.65	15.41	12.39	2.53	11.75	8.88	21.62	1.54	8.49	9.99	2.33	0.79	0.61	1.69	3.24	0.64	1.15	1.55	0.15
Gabon	130.45	11.84	10.14	10.07	2.28	14.47	9.96	13.65	1.35	8.16	11.64	2.78	0.67	0.88	1.88	4.54	0.64	1.16	1.95	0.09
Burundi	158.20	19.61	13.73	9.18	3.04	9.89	10.60	22.42	2.37	10.53	8.80	3.15	1.70	1.46	1.47	3.04	0.90	1.24	2.82	0.10
Comoros	151.13	16.05	11.17	8.58	2.21	11.23	9.45	19.37	2.19	12.39	11.69	2.38	1.45	1.01	1.38	3.38	0.92	1.56	2.94	0.09
Djibouti	143.88	13.63	9.72	8.39	2.10	10.84	8.82	16.87	2.18	12.09	13.30	2.37	1.36	1.07	1.39	3.50	0.87	1.51	2.98	0.10
Eritrea	181.81	19.16	16.53	10.56	3.13	10.83	11.50	27.87	2.87	11.81	13.10	3.02	2.04	1.31	1.71	3.28	1.24	1.43	3.32	0.14
Ethiopia	146.04	10.54	14.58	5.93	1.59	13.19	10.30	19.48	1.45	4.52	14.48	3.19	1.40	0.57	2.36	3.09	0.41	1.30	3.13	0.05
Kenya	91.90	9.24	10.45	4.38	1.68	6.81	6.13	8.03	0.66	6.87	7.73	2.00	1.27	0.83	1.76	2.34	0.40	1.48	1.48	0.05
Madagascar	124.24	11.94	9.70	6.60	1.87	8.53	7.89	15.90	1.66	10.25	9.90	2.27	1.24	0.88	1.06	2.42	0.84	1.25	2.30	0.08
Malawi	125.28	22.62	5.69	5.45	0.90	6.34	5.69	14.98	1.28	6.42	5.05	1.98	0.64	0.22	1.05	2.32	1.67	1.49	1.42	0.16
Mauritius	88.03	3.16	13.60	3.49	2.04	13.26	5.91	5.22	2.55	3.85	7.37	2.33	0.94	0.51	1.61	4.06	0.16	0.44	1.64	0.05
Mozambique	138.56	7.89	8.91	25.59	2.31	9.35	9.10	19.47	1.96	5.24	12.56	2.50	0.13	0.62	1.40	2.23	0.98	1.56	2.43	0.02
Rwanda	156.70	16.59	13.72	9.94	2.95	10.15	9.74	21.93	2.11	12.84	8.45	2.86	1.66	1.37	1.47	3.18	0.91	1.45	2.80	0.08
Seychelles	135.01	6.15	11.41	1.90	5.94	15.56	8.42	6.62	1.61	13.84	11.96	6.01	2.27	2.75	1.70	5.96	0.59	0.58	3.08	0.20
Somalia	145.79	17.13	12.50	8.76	2.25	9.18	7.77	20.30	2.09	12.58	11.39	2.17	1.42	0.87	1.32	2.75	0.81	1.43	2.34	0.08
Tanzania	121.32	6.05	9.21	8.13	1.92	7.71	7.63	15.34	1.82	12.47	10.37	1.47	1.16	0.95	1.28	2.82	0.82	1.38	2.52	0.01
Uganda	134.58	13.18	8.13	8.98	1.57	5.75	9.21	14.50	2.02	18.84	7.74	2.30	1.45	1.21	0.90	2.01	0.87	1.18	2.64	0.05
Zambia	167.86	8.51	12.65	12.47	2.69	10.28	10.73	21.97	2.47	17.31	13.25	2.31	1.73	1.32	1.56	4.07	1.06	2.00	3.34	0.07
Botswana	123.43	12.26	8.33	7.32	2.20	15.65	7.79	11.54	1.27	13.38	8.59	3.17	0.64	0.66	1.33	4.13	1.34	1.97	1.83	0.14
Lesotho	125.66	13.72	11.04	9.22	2.64	12.94	7.70	14.80	1.47	10.88	7.21	3.17	0.71	0.61	1.29	3.53	1.31	1.87	1.75	0.17
Namibia	108.26	3.70	6.05	2.96	3.52	8.91	10.35	10.89	1.61	8.94	7.63	5.12	0.74	1.39	1.15	2.68	2.83	2.37	1.92	0.22
South Africa	122.14	12.63	6.59	9.14	1.96	17.98	8.78	11.17	0.98	11.16	8.39	2.92	0.52	0.51	1.32	4.69	0.98	2.53	1.92	0.14

Swaziland	147.93	18.37	10.45	8.80	2.82	17.06	8.38	15.91	1.70	13.01	10.26	3.84	0.79	0.82	1.52	5.24	1.48	2.00	2.36	0.14
Zimbabwe	140.49	13.49	11.11	16.44	2.20	12.19	6.12	11.33	1.41	16.72	8.96	1.45	0.52	0.34	1.51	4.34	1.10	2.49	1.86	0.07
Benin	113.02	3.75	15.50	18.88	1.34	10.17	6.70	11.10	1.24	10.03	6.57	1.45	0.45	0.43	1.74	2.56	0.39	0.42	1.13	0.10
Burkina Faso	161.65	6.60	16.60	49.09	1.41	8.39	10.94	14.79	1.44	9.51	12.40	1.53	0.54	0.50	1.94	2.28	0.43	0.44	1.23	0.12
Cameroon	127.12	4.63	15.66	21.97	1.53	11.01	8.12	11.91	1.47	10.16	8.36	1.78	0.53	0.60	1.90	3.52	0.43	0.44	1.40	0.09
Cape Verde	104.41	8.24	26.49	12.96	1.34	8.60	5.53	8.87	1.37	4.52	3.55	2.31	0.22	0.81	0.83	3.80	0.17	0.25	0.90	0.06
Chad	113.66	3.00	14.06	28.63	1.14	8.87	6.27	12.15	1.18	8.40	6.49	1.42	0.43	0.39	1.65	1.86	0.37	0.37	0.97	0.09
Cote d'Ivoire	95.51	1.09	7.17	9.48	1.49	7.74	8.28	5.49	0.92	22.16	5.63	1.54	0.74	0.69	1.71	2.00	0.39	0.43	1.46	0.08
The Gambia	100.79	1.93	7.87	36.90	0.81	7.97	4.31	7.23	0.86	4.63	5.70	1.23	0.49	0.38	1.60	1.95	0.26	0.43	0.88	0.13
Ghana	118.79	3.56	11.96	16.54	1.27	6.57	7.85	9.58	2.15	17.58	7.75	1.53	0.26	0.78	1.38	5.32	0.20	0.39	1.26	0.01
Guinea	137.37	1.81	14.96	36.43	1.20	8.81	7.17	18.73	1.60	9.43	6.05	3.49	0.70	1.25	1.80	2.09	0.96	0.44	1.39	0.18
Guinea-Bissau	153.64	5.43	22.83	29.82	2.03	13.43	8.43	16.12	1.57	10.04	11.31	1.94	0.67	0.64	2.05	3.06	0.45	0.46	1.27	0.14
Liberia	111.00	3.96	13.62	21.27	1.34	8.82	6.12	9.52	1.05	10.27	7.71	1.45	0.43	0.44	1.55	2.49	0.37	0.43	0.94	0.09
Mali	140.74	2.63	23.75	40.65	1.20	7.43	7.53	11.26	0.96	5.66	7.50	1.33	0.70	0.29	1.39	2.41	0.74	0.41	0.74	0.16
Mauritania	122.05	4.37	15.52	19.35	1.25	10.37	8.74	13.25	1.72	8.65	8.84	1.58	0.53	0.48	1.80	3.17	0.47	0.40	1.52	0.12
Niger	131.51	3.28	15.66	42.57	1.24	8.60	5.48	12.29	1.18	9.24	6.76	1.37	0.44	0.38	1.69	1.93	0.39	0.39	0.90	0.14
Nigeria	105.32	2.69	6.18	4.06	1.56	8.63	13.89	9.38	0.60	22.15	8.51	0.79	0.66	0.23	1.41	3.30	0.52	0.35	1.31	0.06
Sao Tome and Principe	96.60	2.37	15.79	1.65	0.81	12.23	5.57	11.83	1.91	5.70	8.34	0.64	0.14	0.41	2.04	1.31	0.17	0.17	1.41	0.06
Senegal	117.95	3.66	15.57	19.30	1.38	12.12	6.92	10.11	1.19	10.61	9.18	1.47	0.43	0.43	1.69	2.73	0.42	0.44	1.09	0.12
Sierra Leone	135.43	3.21	14.50	45.27	1.36	10.65	6.50	9.69	1.06	8.86	8.13	1.40	0.41	0.46	1.58	2.21	0.35	0.40	0.98	0.10
Togo	108.51	3.25	14.40	17.56	1.29	9.43	7.42	11.40	1.25	9.08	6.47	1.49	0.46	0.45	1.64	2.39	0.39	0.40	1.22	0.11
American Samoa	139.98	1.25	16.58	8.06	1.51	27.47	9.66	3.76	3.60	20.55	13.03	1.44	1.51	1.60	1.47	4.01	0.99	1.19	3.55	0.02
Bermuda	175.11	6.61	11.34	5.57	2.77	31.26	16.66	4.99	2.35	15.24	22.02	3.39	0.44	1.98	3.71	1.88	2.18	0.97	4.04	0.00
Greenland	302.19	14.02	20.71	5.23	1.98	112.91	13.99	8.16	0.63	4.90	37.01	4.88	8.67	3.80	3.25	19.75	1.33	0.64	6.29	0.05
Guam	120.28	2.01	6.15	7.33	1.44	36.49	7.57	3.17	2.20	8.46	12.63	1.96	3.05	1.10	1.18	4.05	0.67	0.71	1.21	0.08
Northern Mariana Islands	132.36	1.53	10.55	6.74	2.16	39.58	8.56	5.95	2.48	8.07	11.53	4.60	1.87	2.02	1.04	4.56	0.86	1.40	1.13	0.04
Puerto Rico	115.93	6.29	10.70	6.92	2.65	15.25	8.76	3.14	1.38	12.27	12.17	2.95	0.47	1.59	1.34	2.00	0.56	0.83	0.75	0.03
Virgin Islands, U.S.	154.66	3.58	11.35	4.31	2.25	18.32	13.86	6.37	1.97	27.47	20.58	2.13	0.69	1.46	3.10	3.63	1.51	0.42	2.63	0.00
South Sudan	141.34	18.10	11.31	10.42	2.18	10.70	7.30	15.15	1.85	12.88	10.50	2.13	1.38	1.04	1.39	3.11	0.78	1.46	2.13	0.07
Sudan	84.89	5.11	18.58	3.87	2.34	8.78	4.27	2.20	0.41	4.37	6.27	1.01	0.50	0.32	1.63	1.50	0.39	0.32	0.67	0.06

Age-standardized Death change from 1990 to 2017(%)

	Total	Esophageal cancer	Stomach cancer	Liver cancer	Larynx cancer	Tracheal, bronchus, and lung cancer	Breast cancer	Cervical cancer	Uterine cancer	Prostate cancer	Colon and rectum cancer	Lip and oral cavity cancer	Nasopharynx cancer	Other pharynx cancer	Gallbladder and biliary tract cancer	Pancreatic cancer	Malignant skin melanoma	Non-melanoma skin cancer	Ovarian cancer	Testicular cancer
China	-15.72	-45.21	-44.92	-20.27	-25.06	28.24	2.46	-16.30	-53.65	1.76	8.20	29.02	-48.81	-16.17	2.04	47.51	-10.51	29.19	55.39	-54.58
North Korea	0.27	-6.98	-3.59	-5.00	4.74	0.72	21.53	6.96	-3.61	-4.26	6.83	8.24	5.97	-14.75	-9.34	4.82	5.19	23.52	15.82	7.66
Taiwan	10.23	16.33	-43.77	14.22	-37.82	21.23	51.17	-62.60	40.62	10.85	45.09	169.94	-46.18	123.20	-9.60	69.82	37.05	-53.11	72.89	33.61
Cambodia	-17.00	-42.60	-49.51	-12.23	-28.73	-14.97	12.20	-36.89	-27.71	13.51	-5.04	-6.66	-18.67	-8.89	-20.98	19.18	-2.35	1.10	23.61	-38.93
Indonesia	4.29	-12.63	-27.61	20.51	-6.32	15.83	-7.59	-20.11	-15.44	38.67	6.89	-2.18	0.84	-0.01	-5.44	49.27	12.99	36.37	26.01	-41.21
Laos	-20.49	-45.99	-52.86	-17.79	-37.65	-16.52	-3.63	-39.75	-35.46	35.27	-10.54	-28.32	-31.44	-26.29	-25.29	17.42	-6.08	10.72	15.35	-54.66
Malaysia	-10.33	-10.22	-40.62	27.09	-31.27	-0.41	3.95	-42.27	-18.46	-4.90	-8.79	-23.75	-31.13	-9.21	-22.41	49.78	-7.26	2.46	24.88	-21.12
Maldives	-39.27	-59.82	-70.28	-27.90	-60.10	-47.43	-22.36	-71.26	-50.65	-32.04	-25.10	-8.14	-55.85	-45.89	-37.41	4.05	-33.37	-28.61	-0.97	-15.74
Myanmar	-20.26	-25.97	-53.72	-12.79	-39.61	-18.83	-12.31	-39.62	-29.19	26.60	-7.03	-17.53	-25.92	-20.46	-23.47	23.08	-4.67	3.93	14.01	-49.76
Philippines	17.54	-11.96	-40.32	-5.05	-4.96	14.52	83.56	78.02	-16.06	69.93	139.82	-23.91	12.71	-13.57	-40.32	47.94	38.46	50.15	52.19	19.00
Sri Lanka	-11.25	-16.28	-52.60	66.37	-4.69	-6.29	19.61	-9.19	18.45	-29.14	18.42	-5.61	-26.63	-30.12	-54.61	39.29	-4.60	-38.30	39.96	25.57
Thailand	-22.76	-10.19	-52.08	-2.91	-47.71	-31.22	-4.01	-55.64	-31.02	-1.12	-12.54	-38.26	-15.46	-25.98	-23.74	4.19	-25.71	-50.91	-6.19	226.76
Timor-Leste	4.20	-30.92	-34.96	8.30	-9.87	15.40	19.53	-27.14	-21.12	59.86	25.62	-0.97	3.09	-0.52	-6.92	44.70	21.01	35.25	33.62	-27.23
Vietnam	-1.32	-8.90	-51.25	4.48	-1.25	15.53	-4.30	-18.02	-16.16	-4.19	14.62	0.60	4.68	30.21	-7.59	73.30	6.26	-2.17	16.88	-17.07
Fiji	5.36	29.74	-13.52	17.18	18.13	-3.17	18.51	-14.63	16.80	-0.91	17.15	0.65	-2.84	28.92	-4.91	27.99	7.63	-1.38	33.95	-2.00
Kiribati	-1.23	15.91	-23.77	-1.42	-0.91	-0.62	12.97	-12.29	4.57	-15.06	6.08	27.13	1.83	18.39	-4.56	29.94	9.51	9.66	38.52	1.50
Marshall Islands	10.33	1.31	-17.96	9.51	9.40	10.34	33.88	-12.51	12.13	38.61	11.84	18.16	13.61	17.42	-9.27	46.87	8.86	39.89	79.38	-4.68
Federated States of Micronesia	-3.13	-11.54	-23.94	-8.34	-11.42	-0.43	28.73	-27.49	-1.19	-4.68	4.63	3.47	-8.70	-1.22	-22.90	28.41	-6.53	13.57	65.33	-9.14
Papua New Guinea	1.80	-7.16	-14.59	3.43	3.61	8.88	8.94	-18.94	0.96	20.23	4.27	12.77	3.35	5.57	-7.54	31.06	5.36	27.46	52.26	-7.99
Samoa	-4.84	2.69	-10.67	-0.93	-15.36	-2.70	-7.22	-9.23	6.53	-11.34	0.41	-19.72	-16.16	-3.86	-6.25	10.91	-8.52	19.68	17.07	-37.45
Solomon Islands	-3.19	-7.18	-23.52	-4.21	-8.20	-9.49	33.06	-17.06	13.12	-2.36	11.55	11.46	-5.34	8.70	-12.15	27.62	-7.60	16.69	77.28	-16.08
Tonga	-3.73	4.75	-22.08	3.07	-0.02	-9.46	5.68	-24.50	8.34	-10.74	5.17	21.07	8.13	13.48	-9.10	23.24	6.88	-4.01	63.40	-11.02
Vanuatu	5.70	1.55	-15.05	-23.12	6.05	-0.99	33.58	-8.65	26.15	27.93	10.92	21.92	16.11	16.52	2.81	40.17	15.25	33.92	81.68	0.72
Armenia	-3.32	-30.00	-37.68	4.19	-36.54	1.56	7.11	-21.35	37.63	120.68	14.30	-7.68	-11.94	-29.50	81.07	25.99	28.74	272.55	1.22	-40.64
Azerbaijan	6.87	2.00	-14.55	13.29	-10.93	6.14	6.63	1.81	-3.03	72.71	14.87	37.26	28.86	60.35	3.42	58.76	-2.60	43.94	22.05	-27.16
Georgia	34.22	59.77	-3.88	45.62	-1.20	30.09	11.64	-7.67	89.69	91.15	30.96	58.23	-20.07	71.49	77.23	81.01	10.27	288.12	101.78	177.34
Kazakhstan	-34.49	-64.12	-56.09	-39.91	-55.17	-47.64	7.65	-33.80	-31.57	19.08	-5.18	-18.67	-8.63	-36.59	-23.96	302.80	-20.99	100.85	17.47	-56.30
Kyrgyzstan	-33.98	-54.15	-49.80	5.33	-63.46	-51.00	-26.04	-28.12	-6.07	-7.28	-33.82	-45.33	-16.73	-3.16	-28.90	0.44	-29.55	-21.51	-6.56	-41.79
Mongolia	-8.43	-28.37	-40.08	63.83	32.84	-30.59	13.39	-37.86	-12.91	-6.09	-11.70	-55.67	0.48	-33.46	-26.25	56.98	-14.50	8.72	58.13	69.37
Tajikistan	-14.57	-49.81	-24.91	2.30	-38.70	-38.19	19.92	-40.75	55.75	15.97	5.52	-4.82	-7.67	-9.77	-16.80	14.89	9.13	7.86	9.16	9.30
Turkmenistan	-23.77	-71.94	-50.24	49.62	-57.75	-25.20	49.19	12.41	-58.28	17.23	21.15	-4.21	0.20	-10.07	-57.47	151.01	-39.71	20.95	6.38	-26.57
Uzbekistan	-8.76	-67.88	-39.26	32.33	-2.39	-25.64	38.74	5.66	12.71	52.26	26.78	30.52	24.78	61.00	11.07	94.06	18.23	42.91	72.68	-17.05
Albania	-1.01	-30.02	-24.41	-28.27	-32.59	-2.32	43.69	-12.98	-10.36	31.63	11.20	-29.16	-37.28	-28.38	6.58	82.28	-13.02	-2.12	41.89	-9.38
Bosnia and Herzegovina	15.26	-17.35	-23.83	44.53	-38.70	7.82	54.63	-14.56	32.42	49.15	52.42	-12.48	97.86	-6.52	-40.82	28.98	19.33	199.11	65.42	63.01
Bulgaria	6.65	-19.32	-47.75	-13.09	11.47	14.54	13.86	6.92	7.46	16.40	15.51	29.91	64.78	59.45	3.19	44.28	44.15	25.83	38.92	-36.06
Croatia	-12.85	-34.21	-57.51	72.75	-46.50	-14.30	-8.37	-50.84	-7.52	8.65	8.01	-42.84	-48.91	-25.06	-27.41	4.22	21.74	-37.24	-18.04	-8.28
Czech Republic	-33.23	-2.73	-66.52	-25.73	-49.79	-40.28	-35.41	-48.82	-41.81	-2.06	-38.26	-19.43	-45.26	23.65	-49.04	-7.16	-11.38	-32.82	-24.10	-54.34
Hungary	-20.61	-12.76	-58.43	-31.40	-34.13	-1.32	-27.14	-45.83	-50.44	-26.22	-12.64	-11.29	8.08	31.20	-51.10	5.59	-6.46	-15.88	-22.32	-53.42
Macedonia	4.04	-2.47	-40.45	-5.63	-4.07	28.98	-1.88	-17.67	17.35	41.08	24.44	-11.90	-20.36	4.57	-23.85	33.19	-6.55	38.45	18.54	-32.86
Montenegro	1.46	1.77	-18.82	1.75	-12.14	6.44	2.08	-15.96	-7.81	7.60	6.12	-3.99	-11.45	-3.40	-10.93	19.12	-2.19	3.77	8.47	-33.31
Poland	-10.15	-19.44	-49.72	-10.86	-38.29	-4.90	-9.32	-45.67	5.68	26.35	0.45	9.68	-2.19	23.03	-29.65	-7.39	32.83	-31.23	1.50	-34.72
Romania	18.27	50.13	-33.05	127.57	1.34	28.27	9.81	-15.28	-19.99	29.95	44.13	56.75	131.66	137.96	-36.26	39.24	30.93	-13.13	11.12	-21.10
Serbia	0.41	-3.51	-35.99	-7.84	-30.03	4.77	16.55	-24.72	22.43	4.90	8.98	-15.16	-13.89	-3.18	-17.31	26.24	1.99	13.58	21.11	-26.42
Slovakia	-14.55	-9.47	-52.81	-1.25	-49.96	-31.27	-6.80	-20.79	-24.11	11.62	6.67	-37.05	-35.93	42.69	-17.04	19.85	9.69	-28.84	-2.20	-36.20
Slovenia	-19.07	-34.34	-59.14	31.74	-54.82	-18.98	-27.46	-53.36	-35.32	19.10	-13.36	-50.75	-48.77	-10.49	-36.18	6.87	4.54	35.30	-31.90	-38.25
Belarus	-17.77	3.31	-56.22	13.43	-36.97	-31.35	-14.69	-27.56	-21.91	66.82	3.03	-30.56	16.22	99.19	-3.76	12.40	65.86	16.11	-24.63	-25.50
Estonia	-18.64	-13.20	-57.67	15.14	-45.45	-31.33	-19.98	-53.04	-13.73	72.49	-9.47	-21.26	-47.17	22.05	-22.13	6.03	28.30	-8.00	-21.32	-53.52
Latvia	-7.13	23.49	-49.28	31.52	-23.18	-24.98	-2.99	-42.90	29.17	80.87	0.07	2.90	-44.63	72.59	-21.71	6.59	52.24	69.26	-8.76	-21.57
Lithuania	-7.01	39.10	-46.83	52.39	-14.92	-21.97	-0.11	-40.93	32.10	67.04	5.71	-4.27	-36.54	123.90	-21.56	5.25	61.35	30.72	-19.42	-20.80

Moldova	-13.12	-27.22	-49.77	199.38	-9.18	-27.47	-7.31	-40.68	-2.29	27.29	1.64	-20.49	23.36	45.79	-55.30	8.29	16.03	-25.64	-26.55	3.77
Russian Federation	-16.06	-32.81	-53.08	-9.84	-40.95	-31.97	8.16	-31.85	-27.12	40.13	4.97	-7.93	-18.21	9.29	-19.92	0.63	25.26	14.59	-15.67	-33.42
Ukraine	-9.15	-8.77	-48.01	61.68	-28.78	-35.68	-3.91	-48.69	30.69	83.77	2.48	40.81	46.35	71.67	-2.79	46.14	49.88	31.48	19.08	58.45
Brunei	-11.71	-39.53	-52.21	-18.12	-68.69	-16.90	27.70	-34.44	-13.07	14.03	5.53	-24.29	-14.05	11.99	-18.47	22.66	26.32	-42.07	43.46	-44.15
Japan	-20.63	-18.05	-52.51	-40.57	-44.71	-8.43	19.46	-29.66	-18.00	5.94	-7.52	18.10	13.04	74.58	-33.09	9.79	-1.11	-6.86	-12.40	-39.98
South Korea	-33.35	-57.70	-70.08	-46.01	-77.57	11.50	7.36	-53.15	-75.54	30.24	21.80	-19.42	-27.54	42.06	-37.72	4.76	26.35	-58.13	58.38	-46.66
Singapore	-42.69	-65.66	-71.22	-35.72	-70.90	-48.97	-26.35	-67.89	-15.00	1.30	-37.46	-47.22	-58.44	-27.94	-42.90	13.57	-10.85	-46.07	-27.30	-74.91
Australia	-22.91	-3.37	-45.87	121.46	-49.45	-26.78	-31.04	-46.96	-18.65	-17.55	-34.61	-29.44	-27.42	-23.65	-32.76	5.83	0.22	6.13	-24.34	-46.38
New Zealand	-23.97	-21.04	-53.78	71.23	-51.54	-28.20	-36.82	-61.28	-24.06	-17.05	-30.28	-30.25	-38.68	-28.28	-29.00	0.78	4.25	52.21	-32.53	-29.24
Andorra	-15.84	-12.00	-38.46	23.03	-38.18	-22.21	-11.02	-24.97	1.35	-8.26	-19.72	-18.85	-25.83	-17.07	-22.02	-1.41	3.78	-7.06	-5.70	-50.61
Austria	-25.21	1.29	-62.12	13.04	-50.80	-11.21	-33.88	-61.30	-40.76	-15.19	-42.71	-13.55	-1.67	45.78	-50.93	7.79	0.47	-5.64	-41.95	-17.03
Belgium	-28.86	10.99	-57.07	29.07	-59.60	-28.90	-39.47	-48.20	-17.53	-31.93	-35.45	-0.42	-37.26	5.99	-49.21	-3.31	2.87	-8.61	-39.42	-56.51
Cyprus	-11.30	-1.20	-25.42	20.14	-39.71	7.79	-23.53	-39.54	-27.07	-8.62	-14.96	-15.68	-4.21	-9.88	-39.21	37.48	0.11	-32.15	-16.60	-91.57
Denmark	-15.33	-1.64	-44.03	32.53	-48.14	-16.37	-38.47	-59.72	-28.50	6.87	12.66	0.42	-29.39	28.17	-30.05	8.27	6.49	-0.84	-32.60	-57.36
Finland	-24.66	-22.14	-63.73	33.40	-47.75	-28.91	-28.51	-47.09	-22.77	-7.52	-19.96	-3.36	-44.18	0.25	-42.79	-1.12	0.89	-24.16	-30.51	-34.77
France	-25.43	-52.36	-54.35	0.70	-65.41	-3.05	-30.85	-47.63	-25.97	-33.39	-32.75	-50.62	-52.15	-46.73	-53.24	16.74	2.46	-14.06	-30.38	-47.33
Germany	-20.23	9.65	-52.60	35.69	-38.45	-8.48	-29.35	-57.17	-38.15	-5.30	-35.81	-24.40	-40.35	20.62	-54.57	13.61	-0.04	-19.97	-39.92	-51.90
Greece	-6.84	-29.66	-32.85	-53.83	-26.45	-3.61	0.86	-24.20	27.01	-0.62	20.90	8.35	7.14	7.45	24.50	22.97	52.10	-5.65	25.85	-19.98
Iceland	-20.52	-16.19	-62.12	26.53	-38.53	-17.13	-40.41	-52.49	-36.05	-6.36	-17.33	-7.76	-45.36	-8.87	-41.71	-2.26	19.38	19.05	-43.78	-27.83
Ireland	-25.60	-18.75	-52.40	32.37	-43.35	-26.37	-37.89	-37.30	-19.24	-15.80	-33.63	-37.56	-36.01	1.46	-33.94	-10.72	18.95	-32.75	-21.46	-62.56
Israel	-16.05	-21.85	-46.11	12.81	-16.57	-6.92	-28.32	-25.51	24.84	-27.23	-21.57	10.12	-62.19	-41.23	-60.25	7.51	-10.16	17.81	-28.94	-14.88
Italy	-29.05	-46.12	-60.00	-26.87	-59.61	-32.81	-32.51	-8.44	71.36	-16.66	-24.18	-38.26	-22.47	-32.26	-28.45	1.19	-0.87	-9.63	-18.73	-30.43
Luxembourg	-24.50	-26.43	-56.24	10.99	-54.59	-22.04	-34.15	-48.59	-27.59	-26.02	-30.00	-34.08	-40.92	-25.80	-44.76	11.46	0.40	-20.28	-28.46	-60.71
Malta	-15.94	-15.09	-47.75	4.20	-43.38	-10.85	-28.45	-42.22	-9.85	-19.25	-7.69	-15.89	-26.56	-4.15	-37.30	14.84	13.15	-17.21	-15.96	-24.04
Netherlands	-15.06	58.64	-48.04	111.37	-41.36	-20.83	-31.26	-33.62	-3.87	-13.29	-12.09	6.17	-25.32	19.14	-36.10	17.34	38.16	-22.73	-27.95	-36.66
Norway	-17.92	-10.75	-59.31	36.34	-44.63	-6.18	-34.80	-51.47	-28.46	-8.97	-18.52	-28.27	-36.35	-16.74	-32.71	-1.44	7.72	-5.11	-27.38	-47.56
Portugal	-20.64	-31.53	-56.05	17.01	-40.83	4.48	-35.28	-47.61	-30.51	-18.72	-9.21	-22.03	-17.01	77.74	-36.03	-2.42	38.71	-29.49	-13.67	-44.56
Spain	-20.15	-32.18	-53.37	-21.85	-57.39	-10.04	-37.90	-39.02	-9.78	-23.67	0.68	-33.49	-46.26	-6.48	-44.37	12.89	6.88	-47.03	-13.66	-43.51
Sweden	-17.88	-0.29	-57.64	-13.91	-41.14	-4.13	-25.25	-32.94	-21.69	-10.43	-16.23	-13.03	-25.61	2.33	-32.09	-6.01	9.92	-12.96	-38.05	-44.22
Switzerland	-25.51	-25.65	-58.09	0.89	-56.33	-29.87	-46.68	-60.25	-28.88	-28.81	12.85	-24.93	-43.82	3.34	-40.86	-5.21	-0.76	-5.17	-31.42	-66.31
United Kingdom	-22.77	7.56	-52.55	99.68	-33.23	-30.21	-40.53	-53.71	13.40	-2.39	-30.30	0.48	-32.86	12.77	-29.48	2.11	15.40	-6.50	-27.91	-61.61
Argentina	-16.20	-37.35	-38.31	-6.99	-38.85	-18.28	-7.97	-10.18	-28.84	-1.28	2.92	-4.16	-44.14	-53.11	-39.15	9.35	47.30	-5.87	-5.50	-7.35
Chile	-22.29	-50.23	-45.44	-10.04	-46.43	-11.48	-16.57	-57.24	-2.18	19.36	16.25	-20.42	-31.70	-33.62	-43.41	6.86	5.40	27.16	-13.32	-35.58
Uruguay	-13.49	-35.53	-35.12	40.12	-44.43	-18.97	-14.74	-19.09	-24.32	3.84	-2.53	-11.71	-30.57	-33.61	-22.59	26.76	57.01	-14.04	18.76	-38.71
Canada	-25.08	-2.77	-39.06	55.95	-47.78	-28.75	-37.58	-30.81	-24.58	-35.90	-27.31	-34.42	-33.33	-18.34	-33.95	-11.19	3.39	10.32	-26.40	-32.44
United States	-20.78	-0.74	-41.47	77.61	-32.87	-26.71	-34.75	-21.49	-1.31	-30.32	-30.35	-27.86	-32.09	-15.67	-35.51	4.83	-0.20	0.58	-21.49	-10.13
Antigua and Barbuda	-1.36	-15.88	-39.37	-15.39	-12.88	-6.86	16.58	-21.14	36.98	10.62	13.02	-17.14	14.70	-36.69	-65.34	205.13	0.96	327.26	335.27	509.28
The Bahamas	-2.93	-18.92	-35.45	-16.67	-8.24	-14.35	12.90	-19.93	13.43	16.52	5.20	-19.49	14.93	-31.76	-59.71	146.02	-0.84	2.79	214.93	138.81
Barbados	-3.48	-26.36	-45.36	-26.04	-4.68	-10.51	5.67	-32.97	13.17	23.86	16.31	-16.31	24.72	-33.16	-67.35	331.62	3.09	2.50	328.43	445.43
Belize	10.44	20.29	-28.78	13.69	20.58	19.70	20.47	-24.89	-4.54	33.08	26.59	15.46	40.12	1.68	-57.74	220.29	41.91	26.46	210.66	504.39
Cuba	-0.56	27.97	-31.22	-21.74	20.53	-0.21	0.69	-31.53	27.25	10.50	4.42	8.48	22.70	-19.24	-69.93	202.49	7.97	2.61	233.42	502.49
Dominica	11.22	3.41	-27.63	-7.38	21.78	12.79	7.88	-24.92	19.69	57.13	23.46	6.68	52.21	-8.77	-63.45	304.85	24.45	19.96	265.73	434.37
Dominican Republic	31.11	22.45	-2.40	73.51	-11.11	34.50	69.19	-1.80	-41.60	67.38	39.67	2.27	118.78	31.66	-51.29	222.34	6.62	43.24	181.84	14.48
Grenada	-9.56	-11.75	-40.37	-33.78	0.60	1.71	9.54	-43.71	10.83	12.25	1.01	-7.62	-21.16	-36.61	-72.34	337.45	-26.33	368.35	292.17	80.68
Guyana	2.65	-7.97	-36.03	-7.25	-3.27	12.14	28.96	-15.05	30.26	6.53	8.26	-5.98	29.10	-20.72	-61.08	195.53	18.73	219.65	330.91	431.80
Haiti	-7.97	-21.95	-33.46	-14.61	-19.37	-21.51	17.71	-34.22	-3.72	32.18	11.68	-4.18	-2.99	-25.76	-39.02	79.45	3.03	45.48	98.75	53.83
Jamaica	22.96	2.77	-33.50	-4.62	44.23	34.58	33.34	-17.21	92.95	57.98	34.32	-8.89	82.87	-1.13	-37.75	201.87	31.27	33.40	375.52	345.61
Saint Lucia	-13.94	-21.51	-43.35	-31.52	-13.93	-11.33	-13.29	-40.75	-3.81	7.03	-5.41	-21.93	10.76	-35.13	-70.89	277.26	-7.49	29.64	227.24	524.32
Saint Vincent and the Grenadines	6.04	12.61	-31.09	-2.73	25.97	10.61	-2.16	-33.13	1.84	56.87	10.49	2.29	22.17	-12.95	-64.79	220.57	-11.81	384.29	224.86	598.90
Suriname	10.36	-0.80	-31.86	-1.05	9.67	15.07	17.10	-21.97	22.70	36.49	25.77	-0.46	33.74	-19.57	-63.94	328.14	16.52	47.31	317.85	495.56
Trinidad and Tobago	-16.36	-40.15	-57.78	-49.44	-29.75	-11.75	-10.97	-37.61	8.02	3.76	-11.20	-30.27	12.20	-39.26	-69.18	266.04	-16.95	-11.23	464.61	289.75
Bolivia	-15.17	-23.92	-39.94	1.22	-38.25	-6.26	7.95	-44.45	-24.38	17.66	7.24	-9.71	-23.39	-35.24	-29.73	67.79	1.18	25.60	69.82	36.45
Ecuador	-4.80	-39.13	-37.97	3.80	-33.89	12.15	25.96	-26.33	-36.20	8.58	26.99	-1.01	-4.18	-41.46	-41.32	162.31	-4.64	23.91	268.21	437.92
Peru	-14.57	-33.04	-31.96	6.85	-58.19	-25.75	0.11	-31.84	-38.70	-0.46	9.96	-29.15	-22.15	-60.90	-28.30	104.51	1.90	-7.35	132.86	230.67
Colombia	-31.99	-56.08	-56.19	-41.44	-57.30	-25.93	-17.37	-44.41	-42.59	-18.51	-3.72	-41.83	-50.95	-55.64	-50.26	-21.34	0.32	-29.40	-5.80	14.78
Costa Rica	-11.32	-34.21	-49.82	15.43	-32.08	-16.20	15.06	-49.68	17.42	24.56	40.50	-21.04	-1.26	-16.97	-52.92	85.89	26.78	-17.50	77.37	277.01
El Salvador	20.72	14.11	-2.68	65.55	-4.08	35.58	49.27	5.60	-16.54	24.11	71.04	-20.67	-5.35	-39.13	-13.17	166.49	1.54	72.43	194.42	338.03
Guatemala	11.62	17.49	-14.51	89.16	-38.72	-15.15	21.12	31.63	-24.16	50.54	40.48	-18.22	21.90	-35.09	-63.95	143.60	83.06	-3.74	239.31	1508.96

Honduras	13.78	24.97	-13.27	30.63	-10.40	6.29	21.13	-27.80	32.98	15.29	38.15	15.27	0.94	5.59	13.10	84.16	22.24	11.07	127.66	-33.13
Mexico	-16.25	-27.35	-37.02	-8.67	-46.59	-35.52	10.24	-58.07	-17.31	-1.41	31.56	-15.30	-28.06	-28.91	-47.24	-5.54	34.46	-13.83	27.78	39.06
Nicaragua	-13.37	-31.53	-38.28	8.01	-44.18	-8.12	24.47	-47.52	-31.18	-8.14	19.42	-20.49	-19.79	-37.14	-47.10	90.61	-7.23	101.81	153.21	692.89
Panama	-12.56	-12.91	-34.53	17.74	-47.95	-27.53	7.75	-44.81	13.09	10.18	6.44	-40.07	-14.68	-30.66	-50.62	50.49	-8.99	1.23	60.13	363.49
Venezuela	-9.73	-25.83	-46.90	-26.13	-18.72	1.24	18.45	-31.94	-35.94	26.74	10.70	-12.12	0.09	-28.73	-58.46	216.38	17.43	-7.80	339.64	842.44
Brazil	-10.00	-19.45	-45.25	3.95	-17.85	-8.91	-1.56	-37.48	-18.57	-0.04	17.42	-6.00	8.63	-6.35	-34.74	12.74	15.56	6.02	5.38	5.51
Paraguay	12.69	6.83	-25.78	-6.53	5.31	32.97	32.64	-16.14	-31.98	34.90	59.49	-5.71	-1.88	13.04	-25.37	118.53	29.83	8.72	116.67	404.39
Algeria	-9.04	-11.49	-39.79	23.68	-35.22	-16.66	16.40	-39.88	-15.61	-2.12	0.92	-15.76	-14.07	-4.78	-23.27	55.26	2.75	6.93	27.93	-38.70
Bahrain	-48.88	-67.45	-64.84	-23.90	-68.27	-66.41	-28.58	-52.45	-24.51	-31.29	-27.38	-51.31	-61.01	-54.73	-42.52	-27.73	-30.53	-46.59	-33.33	-47.33
Egypt	14.08	16.52	-24.26	48.46	-8.34	24.88	20.01	-21.28	-5.89	0.72	20.77	6.39	-14.68	12.60	-8.59	54.16	6.72	0.52	29.28	-6.18
Iran	3.56	-17.39	-31.87	23.51	-28.04	9.99	40.61	-27.06	24.18	43.93	22.60	16.10	-27.81	0.62	2.02	85.72	-25.36	63.10	85.90	88.61
Iraq	-41.79	-56.13	-61.07	-26.82	-62.25	-44.19	-44.08	-57.94	-19.78	-28.61	-36.29	-50.44	-78.56	-51.87	-50.99	-19.26	-38.61	-41.13	-16.24	-56.78
Jordan	-8.33	-16.00	-42.80	12.63	-64.47	0.25	-4.33	-49.45	-21.96	62.26	3.04	-1.82	-38.61	-9.39	-21.50	56.78	-14.35	1.92	4.47	-58.22
Kuwait	-25.56	-31.87	-53.19	14.35	-49.68	-32.48	-35.67	-62.88	-18.91	25.68	4.60	-46.84	-52.43	-47.84	-56.80	1.49	-30.62	1.65	-47.49	-86.44
Lebanon	-11.96	-17.25	-50.26	-11.69	-52.55	-13.46	14.55	-48.09	-20.06	-12.88	-4.60	-11.51	-41.62	-22.16	-28.63	34.29	-3.09	4.97	23.72	-11.63
Libya	8.49	24.52	-11.58	12.75	-11.60	-2.76	55.84	-13.77	7.56	13.17	16.20	8.88	-14.28	-5.99	-15.26	39.79	12.14	22.14	54.28	9.08
Morocco	1.41	-4.81	-26.44	9.40	-14.32	4.14	11.84	-35.98	-11.47	12.68	19.27	3.88	-15.64	-0.27	-12.40	51.63	10.10	26.90	30.96	-17.62
Palestine	-9.46	-25.38	-41.80	-9.41	-39.75	-14.65	7.88	-31.81	-8.12	-2.88	-3.73	-10.58	-20.48	-14.03	-14.02	22.26	-6.39	0.27	32.17	7.61
Oman	-15.45	-14.97	-50.69	-7.11	-43.28	-16.81	8.88	-51.32	-24.71	3.68	7.00	-21.65	-42.20	-18.80	-30.20	59.31	-10.84	-8.74	5.18	-48.29
Qatar	-34.27	-54.42	-53.55	-34.71	-14.12	-36.48	-29.75	-49.69	-11.54	8.54	-30.52	-30.61	-16.56	-3.44	-69.06	-27.02	-40.03	4.93	-1.81	63.06
Saudi Arabia	15.90	-5.92	-37.97	4.99	-28.32	19.43	48.81	-22.46	18.61	25.63	67.42	6.56	-17.43	5.08	-3.84	111.08	10.57	21.26	53.68	-0.28
Syria	-4.74	-0.83	-32.93	-9.03	-22.81	1.23	11.30	-37.59	-22.93	19.56	9.64	-2.75	-18.49	-2.04	-22.36	59.21	-3.03	19.23	35.39	-15.33
Tunisia	-4.40	-0.30	-30.71	3.89	-22.41	-2.48	16.49	-31.62	-8.50	-3.13	6.11	-2.29	-26.24	-1.17	-18.37	43.86	1.84	10.37	29.53	-22.35
Turkey	-27.77	-42.47	-55.28	-10.29	-50.07	-18.43	-13.99	-57.58	-39.06	-7.35	-16.59	-56.75	-45.75	-37.67	-42.79	2.38	-21.36	-26.53	-18.06	-41.64
United Arab Emirates	11.42	36.75	-13.86	27.40	2.05	13.23	5.59	-40.79	-16.79	21.17	14.65	3.30	-9.48	-15.59	-22.64	65.78	4.44	31.72	8.85	75.32
Yemen	4.18	-18.08	-11.09	-3.74	-5.15	-1.80	35.30	-27.09	-7.40	64.75	26.80	4.56	-24.03	-13.31	-15.30	63.80	9.15	49.17	51.76	6.81
Afghanistan	-2.06	-14.76	-9.12	-11.57	-8.84	-9.48	20.00	-7.32	9.34	3.12	17.97	-5.42	-26.49	-8.89	-3.97	22.65	1.07	17.39	47.76	22.12
Bangladesh	-22.97	-35.74	-49.51	-6.17	-53.43	-5.35	32.31	-63.05	-47.56	-25.98	-23.26	26.50	-45.06	-24.00	-25.45	15.20	-25.20	-30.85	9.81	290.96
Bhutan	-17.15	-41.37	-41.88	20.18	-43.28	-8.69	-13.34	-66.91	-45.23	18.88	8.29	-25.56	-31.30	-2.13	0.00	61.17	-0.25	4.70	22.32	-56.54
India	6.83	-10.76	-30.01	56.71	-26.92	9.66	44.02	-25.15	-8.98	12.88	26.02	4.87	-8.41	15.63	34.18	89.68	21.56	8.05	66.37	-45.45
Nepal	-7.36	-18.23	-30.14	54.30	-32.41	-3.41	11.51	-52.62	-29.20	17.23	13.11	-16.78	-25.64	9.08	12.21	80.42	2.62	0.24	51.67	-63.97
Pakistan	24.02	11.47	-4.12	29.85	-2.90	12.35	58.82	-11.76	23.24	24.98	41.13	25.16	0.00	28.31	15.27	73.53	28.31	12.72	73.51	-11.79
Angola	-18.85	-31.70	-48.82	-39.08	-30.08	-22.30	21.63	-33.10	-13.97	2.21	0.27	-1.00	-32.72	-6.62	-14.28	28.98	-4.36	-4.34	30.30	-38.46
Central African Republic	-13.58	-27.73	-29.59	-35.82	-19.58	-14.35	18.73	-18.42	-9.99	-0.57	-0.18	-3.74	-18.94	-13.92	-15.59	1.21	-6.31	-3.30	9.05	2.31
Congo	-12.66	-36.31	-42.78	-30.64	-30.19	-17.09	15.39	-29.88	-16.45	34.46	1.02	-4.93	-26.47	-17.96	-17.88	20.90	0.53	21.20	17.52	-36.97
Democratic Republic of the Congo	-12.36	-29.22	-27.34	-33.56	-20.76	-16.96	23.38	-8.77	-3.79	-10.34	0.89	-0.92	-18.08	-8.09	-10.03	-1.07	1.08	-8.12	13.86	-5.09
Equatorial Guinea	-21.26	-32.90	-68.59	-20.25	-51.03	-5.44	15.51	-58.09	-26.00	10.40	3.39	-7.61	-50.36	8.94	-16.27	84.72	-8.87	-3.83	47.49	-65.29
Gabon	-7.80	-24.37	-40.12	-15.75	-22.55	-4.76	8.07	-37.58	-20.89	27.87	2.72	-5.25	-21.92	-16.28	-25.71	30.26	-1.22	19.24	11.88	-18.31
Burundi	-31.30	-51.21	-42.32	-35.40	-42.43	-23.82	-26.36	-46.13	-45.37	-0.03	-5.14	-21.43	-33.95	-37.22	-25.67	-12.46	-23.75	-1.70	-28.61	-46.14
Comoros	-18.39	-46.15	-40.84	-24.82	-38.20	-24.37	18.66	-34.18	-24.56	0.01	-2.20	-7.94	-25.99	-25.72	-15.52	11.46	-10.58	-3.89	12.88	-44.10
Djibouti	-9.60	-35.79	-33.44	-8.52	-25.30	-2.31	9.49	-38.34	-30.04	18.48	2.18	0.98	-13.66	-18.87	-17.48	30.47	-9.47	15.40	1.27	-41.25
Eritrea	-12.70	-45.80	-42.09	-16.66	-40.12	-15.01	27.90	-28.76	-14.31	-0.50	5.81	-1.16	-21.90	-16.26	-3.94	30.92	2.94	2.69	30.73	-39.38
Ethiopia	-30.82	-49.30	-60.27	-28.00	-44.58	-34.59	-16.34	-57.29	-41.78	37.09	-20.93	-16.19	-34.05	-25.05	-22.71	0.65	-8.88	15.85	-7.88	-11.09
Kenya	7.04	-8.84	-8.64	33.74	-1.35	-5.66	20.60	-19.91	-2.96	29.36	12.37	1.36	15.73	12.98	4.03	44.16	10.82	0.53	17.89	43.52
Madagascar	-16.15	-34.57	-27.79	-16.85	-29.61	-14.64	4.74	-20.83	-18.77	-6.89	-3.30	-13.47	-17.55	-18.74	-6.46	4.93	-8.96	-5.21	-2.36	-41.48
Malawi	-3.71	5.42	-37.20	0.46	-18.53	-9.94	8.90	-24.71	-17.27	-2.93	15.89	-1.26	-24.89	3.40	-15.06	19.27	1.72	-11.72	0.27	6.42
Mauritius	-3.17	-28.36	-51.81	9.97	-48.13	-12.41	85.13	-45.97	-34.54	44.18	49.87	4.45	-23.50	12.73	-31.42	12.47	25.50	-6.80	49.43	3.78
Mozambique	0.31	1.74	-19.04	19.22	-17.37	9.38	16.64	-23.35	-14.78	-17.01	12.05	1.96	-5.73	12.22	-12.98	37.61	-14.19	-11.17	10.22	-14.40
Rwanda	-32.25	-56.74	-56.60	-35.88	-55.28	-27.14	-9.56	-51.08	-38.29	-12.85	-7.94	-24.34	-45.02	-41.57	-21.52	3.07	-17.30	-11.07	-3.48	-54.21
Seychelles	14.74	-15.88	-51.46	219.77	-22.29	-1.86	30.07	27.53	-26.34	60.95	82.87	10.46	-25.55	35.84	-22.60	5.39	8.08	6.07	32.70	-27.51
Somalia	-6.29	-34.77	-26.08	-8.28	-23.46	-9.82	16.78	-13.15	-13.87	2.41	12.46	8.45	-8.08	-9.24	3.28	15.06	6.69	7.59	16.39	-17.54
Tanzania	-11.26	-22.67	-33.43	-19.37	-30.77	-4.26	5.95	-32.45	-25.78	-0.51	-6.28	-5.95	-16.88	-14.14	-10.98	22.16	-6.98	2.57	4.97	-19.35
Uganda	2.20	7.16	-32.23	-0.24	-19.23	16.71	4.18	-36.07	-13.24	11.72	16.79	17.53	7.78	22.20	-26.52	69.49	15.98	0.62	-4.10	38.89
Zambia	-23.80	-25.66	-42.25	-25.29	-37.98	-16.28	-8.36	-43.96	-31.63	-25.95	-9.17	-11.46	-27.63	-21.77	-20.56	8.11	-22.17	-21.15	-7.90	-23.10
Botswana	-7.41	-33.43	-40.17	-1.35	-39.46	-22.02	33.45	-15.35	12.96	8.77	5.69	-22.21	-25.19	-14.94	-19.13	35.01	-8.17	1.21	36.13	-42.70
Lesotho	22.07	5.04	-7.87	13.14	-0.61	24.91	69.30	18.52	48.58	13.27	42.10	18.57	15.48	41.05	12.61	64.48	15.28	14.70	78.21	20.47
Namibia	-1.10	-19.16	-42.76	9.56	-28.32	-16.25	33.61	-30.29	-28.49	48.45	1.73	0.39	-33.45	-5.10	-26.55	35.55	-6.58	-2.70	-5.47	-39.46
South Africa	-8.41	-20.54	-29.75	-42.72	-24.78	-8.81	8.34	-16.48	22.31	5.93	8.98	-19.03	-21.07	5.33	-9.79	16.12	8.69	-8.49	19.10	-10.24

Swaziland	1.62	-15.57	-25.34	53.58	-20.74	-9.50	38.14	-13.47	5.22	-5.09	18.72	-7.37	-5.67	7.11	-11.79	32.01	-0.71	3.29	29.40	1.56
Zimbabwe	18.00	7.12	0.26	-5.69	0.78	-12.66	71.49	31.47	56.72	15.63	23.62	35.25	28.09	31.01	-6.12	42.13	32.25	-3.68	70.96	60.06
Benin	1.87	75.12	-19.67	-23.33	-2.81	-7.24	22.11	-7.14	16.83	42.83	15.86	1.72	-22.83	11.45	-19.46	78.05	-9.90	24.89	28.68	-55.58
Burkina Faso	-23.44	2.73	-17.45	-68.05	5.70	-5.68	-1.00	-18.50	1.75	27.32	5.27	-6.52	-30.61	10.31	-24.83	66.19	-16.79	16.92	10.89	-39.86
Cameroon	1.49	53.97	-19.35	-27.21	8.13	2.16	11.46	-19.80	-0.35	63.93	8.65	2.85	-28.55	7.73	-21.54	68.92	-10.99	35.94	23.21	-50.10
Cape Verde	14.80	28.37	-35.31	-22.21	-30.75	34.45	21.22	-33.94	27.59	237.96	108.48	24.98	13.76	10.89	7.91	91.16	49.19	54.95	51.29	-50.92
Chad	4.92	84.87	4.04	-47.21	38.70	34.78	7.82	-4.52	5.04	86.54	24.80	12.94	-9.62	33.28	-9.62	81.51	-1.30	55.50	14.02	-27.75
Cote d'Ivoire	6.98	21.92	-17.94	-6.00	-8.77	-13.04	20.99	-18.63	3.15	25.82	0.59	4.91	-8.83	-4.31	-13.07	34.36	6.38	51.56	25.11	-18.88
The Gambia	9.82	13.21	-20.70	6.23	-8.81	-0.17	55.31	1.21	15.97	28.14	9.90	20.22	0.87	7.24	-4.17	58.45	17.39	51.57	57.52	-33.14
Ghana	-3.41	13.25	-30.10	-1.19	21.13	10.46	26.40	-5.47	5.27	-26.97	11.70	4.67	-44.67	7.01	-5.42	55.90	13.72	9.28	43.10	13.07
Guinea	5.06	31.85	2.20	-0.08	38.59	12.35	25.55	-17.94	1.59	13.47	24.58	20.10	-6.79	5.23	-13.53	42.95	4.55	38.98	23.79	10.61
Guinea-Bissau	-9.63	53.11	-29.01	-39.18	-14.37	-19.85	26.27	-11.14	17.30	30.25	-6.65	-2.67	-30.55	-1.90	-20.46	62.87	-12.21	18.37	37.05	-57.24
Liberia	-0.30	52.75	-14.22	-27.14	-7.66	-11.51	30.27	-1.74	19.27	47.29	13.24	-6.30	-26.28	-2.49	-22.59	55.65	-11.61	24.44	33.27	-52.51
Mali	-16.00	-8.10	-35.15	-20.75	-27.22	-4.58	-2.17	-41.28	-28.28	26.35	-2.48	-0.77	-38.92	-11.19	-19.50	26.86	-3.81	44.84	7.03	-41.38
Mauritania	-3.56	20.51	-32.37	-33.85	-9.15	-5.49	2.04	-31.87	-14.67	117.59	-1.38	-2.01	-40.27	-7.51	-25.23	67.12	-7.40	60.47	12.63	-66.18
Niger	-15.55	37.17	-17.02	-47.53	-4.00	-3.48	6.91	-5.83	3.76	46.72	-4.06	-5.68	-30.39	2.50	-20.88	47.14	-13.39	25.63	18.24	-65.50
Nigeria	7.14	39.49	-27.39	-5.22	-25.85	-2.61	19.86	-22.11	-1.13	29.51	0.74	9.18	-22.79	-7.74	-8.65	33.10	6.52	44.27	39.49	-55.96
Sao Tome and Principe	26.83	85.83	8.28	23.04	25.55	26.51	66.26	-5.43	23.24	45.31	52.91	39.79	11.55	41.41	7.31	79.95	22.22	28.37	56.76	-40.72
Senegal	6.48	42.24	-16.14	-22.76	7.06	-4.67	31.42	2.30	18.24	83.48	-12.74	8.06	-15.87	13.89	-10.46	66.79	-0.04	49.16	39.53	-55.33
Sierra Leone	-13.82	63.10	-8.12	-69.09	9.22	-3.41	38.27	12.50	30.44	61.50	10.82	6.69	-14.05	21.25	-14.26	74.75	-2.75	38.55	40.51	-51.09
Togo	-1.71	55.17	-14.90	-33.13	1.26	-3.09	23.67	-11.66	14.08	34.27	13.63	1.41	-25.35	7.23	-20.99	68.25	-10.82	21.69	25.01	-44.11
American Samoa	4.95	12.41	-22.17	25.42	-21.07	-7.77	48.93	-5.76	44.46	-8.48	-2.83	23.23	0.68	28.62	-28.97	36.90	-8.63	10.62	88.88	128.68
Bermuda	-29.72	-36.06	-56.34	-40.87	-30.46	-32.22	-45.52	-61.85	-36.15	0.30	-26.69	-37.68	-10.93	-49.31	-78.41	336.72	-25.28	14.41	-25.24	100.35
Greenland	-28.36	-18.48	-43.45	18.52	-12.80	-32.53	-43.93	-50.82	-29.77	14.39	-28.41	-32.46	-44.80	-29.19	-22.37	-12.08	-24.32	15.35	-18.52	-70.74
Guam	9.90	26.87	-19.41	55.44	-3.80	2.28	27.29	-9.71	3.11	-10.56	5.12	37.96	8.36	30.61	-28.18	53.01	-1.58	29.73	154.17	135.03
Northern Mariana Islands	-6.58	12.94	-37.56	11.22	-15.95	-20.25	9.46	-22.99	19.26	15.61	-16.59	41.40	-13.10	6.62	-26.55	32.36	11.01	-1.33	58.43	131.85
Puerto Rico	-22.94	-64.75	-60.16	-21.08	-59.94	-29.26	-4.04	-30.38	-8.79	-28.91	6.19	-54.47	-30.18	-60.80	-47.06	154.60	-4.60	2.53	180.15	381.85
Virgin Islands, U.S.	8.70	12.40	-24.59	5.45	5.51	17.67	4.21	-30.21	-0.21	9.49	13.33	11.12	14.01	1.05	-47.01	128.11	19.16	113.98	83.18	14.87
South Sudan	-10.46	-32.21	-25.41	-19.16	-23.75	-1.82	-1.53	-15.43	-23.51	-0.91	3.22	-2.64	-10.52	-15.59	-12.54	9.31	-5.40	2.97	-1.08	-16.95
Sudan	-4.49	-7.61	-19.78	-7.07	-14.62	-5.04	8.07	-37.78	-19.00	29.70	13.82	-4.85	-33.56	-15.84	-19.67	63.39	-2.16	28.90	25.19	-7.30

Age-standardized Death in 2017

	Kidney cancer	Bladder cancer	Brain and nervous system cancer	Thyroid cancer	Mesothelioma	Hodgkin lymphoma	Non-Hodgkin lymphoma	Multiple myeloma	Leukemia	Other malignant neoplasms
China	0.94	1.75	3.43	0.37	0.14	0.15	2.18	0.64	3.76	3.56
North Korea	1.02	1.79	3.10	0.42	0.17	0.27	2.17	0.53	4.97	3.93
Taiwan	2.26	2.71	1.94	0.53	0.12	0.09	3.59	1.07	3.39	3.96
Cambodia	1.00	2.55	2.43	0.87	0.23	0.53	3.02	0.83	6.10	4.53
Indonesia	1.02	2.69	2.87	0.69	0.19	0.40	2.91	0.72	5.51	4.35
Laos	1.08	2.65	2.64	0.78	0.19	0.56	3.18	0.78	6.37	4.53
Malaysia	1.22	2.53	1.93	0.85	0.17	0.28	3.46	0.85	5.10	4.10
Maldives	0.88	2.39	1.44	1.01	0.41	0.09	1.96	0.80	3.32	2.45
Myanmar	1.33	2.98	3.11	0.92	0.46	0.58	3.51	0.96	7.31	5.50
Philippines	1.24	1.10	2.61	1.55	0.18	0.20	2.68	0.66	5.35	5.49
Sri Lanka	2.21	1.30	2.00	0.53	0.32	0.36	2.18	0.84	5.05	4.52
Thailand	0.80	1.58	2.75	0.51	0.25	0.19	1.64	0.60	5.41	3.22
Timor-Leste	0.95	2.68	2.45	0.73	0.17	0.51	2.99	0.71	6.66	5.00
Vietnam	0.66	1.21	3.04	1.15	0.18	0.36	3.37	0.61	3.63	5.86
Fiji	0.77	1.80	1.47	1.30	0.30	0.26	2.28	1.21	4.86	5.16
Kiribati	1.32	1.59	1.05	0.42	0.16	0.21	2.04	0.98	4.27	6.04
Marshall Islands	1.46	2.50	2.18	1.00	0.35	0.37	3.49	1.67	5.82	6.02
Federated States of Micronesia	1.10	2.33	1.81	0.84	0.37	0.32	2.76	1.40	5.21	5.30
Papua New Guinea	0.74	1.89	1.86	0.75	0.32	0.49	2.63	0.95	5.77	5.62
Samoa	0.74	1.53	1.47	0.76	0.20	0.38	4.31	1.40	4.34	5.12
Solomon Islands	0.84	1.86	1.49	0.70	0.30	0.38	2.35	1.10	4.99	4.93
Tonga	1.35	1.94	1.74	0.69	0.19	0.20	5.12	1.72	3.66	4.22
Vanuatu	1.24	3.32	2.28	0.92	0.42	0.64	3.62	1.10	8.56	8.52
Armenia	2.57	4.85	5.31	0.64	0.53	0.21	1.70	0.66	4.02	8.39
Azerbaijan	2.93	2.48	4.02	0.39	0.10	0.55	1.85	0.70	4.63	5.55
Georgia	2.51	3.78	4.56	0.53	0.40	0.91	2.74	0.81	5.32	6.66
Kazakhstan	2.85	2.07	2.54	0.47	0.24	0.41	1.41	0.64	3.38	5.49
Kyrgyzstan	1.56	1.40	2.32	0.36	0.10	0.24	1.22	0.47	2.62	4.38
Mongolia	1.90	1.11	2.80	0.50	0.12	0.16	2.09	0.51	2.67	6.83
Tajikistan	1.84	1.30	5.42	0.18	0.17	0.22	2.22	0.61	3.26	5.30
Turkmenistan	2.74	1.40	3.76	0.34	0.12	0.46	1.52	0.96	3.36	5.18
Uzbekistan	1.46	1.61	3.91	0.21	0.09	0.46	2.10	0.59	3.50	4.37
Albania	2.00	1.03	5.85	0.41	0.13	0.45	1.52	0.71	4.12	8.14
Bosnia and	3.13	3.98	6.84	0.46	0.14	0.42	2.22	0.93	4.41	5.37
Bulgaria	2.33	3.75	5.58	0.38	0.08	0.59	2.22	0.82	4.27	4.31
Croatia	3.76	4.55	6.39	0.42	0.78	0.34	3.54	2.01	4.92	4.16
Czech Republic	5.57	3.89	4.14	0.39	0.25	0.46	3.10	1.98	5.32	4.40
Hungary	4.11	4.56	3.91	0.43	0.21	0.33	3.11	1.64	5.37	3.56
Macedonia	1.62	4.27	5.73	0.31	0.05	0.49	1.75	0.72	4.15	5.26
Montenegro	2.33	3.39	6.02	0.55	0.15	0.78	1.92	0.83	4.96	2.38
Poland	4.58	5.37	5.12	0.45	0.44	0.45	3.09	2.05	5.17	4.50
Romania	2.66	4.04	5.07	0.43	0.21	0.42	2.46	1.11	4.41	6.23
Serbia	3.32	5.01	6.42	0.49	0.24	0.58	2.96	1.47	5.21	5.04
Slovakia	3.85	3.73	4.43	0.43	0.17	0.47	3.51	2.03	4.91	5.39
Slovenia	3.40	3.98	3.55	0.38	0.72	0.33	4.07	2.28	5.36	3.79
Belarus	3.89	2.54	3.28	0.54	0.18	0.50	2.33	1.44	4.66	6.21
Estonia	4.46	3.44	3.97	0.52	0.19	0.34	3.13	2.01	5.47	4.50
Latvia	4.45	4.80	4.54	0.57	0.24	0.43	2.84	1.80	5.32	5.14
Lithuania	4.77	4.01	4.61	0.47	0.19	0.39	2.77	1.99	5.80	4.72
Moldova	2.31	2.62	3.44	0.39	0.10	0.64	2.41	0.77	3.14	4.41
Russian Federation	3.72	2.87	3.15	0.48	0.27	0.45	2.59	1.54	4.22	9.92

Ukraine	3.97	3.20	4.78	0.50	0.29	0.88	2.58	0.99	4.96	6.11
Brunei	2.95	2.88	3.60	0.91	0.38	0.52	6.58	2.78	5.69	5.40
Japan	1.93	2.06	1.30	0.48	0.43	0.08	3.29	1.30	3.14	3.03
South Korea	1.79	1.97	1.77	0.87	0.15	0.08	2.50	1.21	2.96	2.81
Singapore	1.24	1.07	1.36	0.29	0.30	0.10	2.14	0.76	2.49	2.39
Australia	3.28	2.80	4.18	0.41	1.89	0.24	4.56	2.61	5.78	3.57
New Zealand	3.24	2.85	4.12	0.37	1.23	0.25	4.80	2.75	5.63	4.09
Andorra	2.72	4.51	4.01	0.35	1.95	0.30	4.17	2.51	5.62	3.81
Austria	3.09	3.24	3.68	0.42	0.66	0.25	3.70	2.11	5.33	4.37
Belgium	3.20	4.34	3.89	0.43	1.15	0.30	3.76	2.42	5.84	4.24
Cyprus	1.64	4.06	3.56	0.41	0.69	0.29	3.85	2.19	6.00	3.59
Denmark	3.68	5.16	5.09	0.34	1.15	0.35	3.72	2.43	6.68	4.19
Finland	3.62	2.27	3.88	0.47	0.81	0.29	4.59	2.39	4.32	4.00
France	3.25	4.25	3.70	0.38	1.25	0.31	3.77	2.34	5.62	4.52
Germany	3.76	3.59	4.19	0.50	0.86	0.28	3.70	2.24	5.60	4.56
Greece	2.77	5.10	6.13	0.36	0.33	0.88	2.14	1.89	6.49	3.40
Iceland	5.23	3.58	5.36	1.02	0.87	0.21	3.24	3.01	5.31	4.50
Ireland	3.36	3.17	4.24	0.34	0.58	0.32	4.26	2.76	5.60	3.68
Israel	2.72	3.77	4.57	0.65	0.38	0.38	5.25	2.55	6.14	3.50
Italy	2.89	4.43	3.56	0.42	1.16	0.41	3.73	2.33	5.41	3.98
Luxembourg	1.86	4.07	5.25	0.61	0.87	0.26	3.86	2.43	6.44	4.39
Malta	2.91	4.09	3.92	0.55	1.02	0.48	4.02	1.97	4.94	4.06
Netherlands	3.94	4.67	3.71	0.42	1.77	0.32	4.35	2.76	5.66	4.59
Norway	3.99	3.86	4.36	0.43	0.79	0.24	4.03	3.35	4.62	3.68
Portugal	1.97	3.94	4.56	0.43	0.30	0.31	3.78	2.24	4.76	3.93
Spain	2.61	5.25	3.73	0.38	0.52	0.31	3.10	2.00	4.56	3.68
Sweden	3.73	3.58	3.73	0.38	0.76	0.21	3.83	2.68	4.33	3.61
Switzerland	2.38	3.02	3.15	0.41	1.06	0.22	3.47	2.23	4.50	3.17
United Kingdom	3.61	4.40	4.16	0.33	2.13	0.35	4.39	2.55	5.43	3.91
Argentina	4.32	3.00	3.21	0.48	0.42	0.45	3.52	1.51	4.73	4.90
Chile	3.93	2.58	2.33	0.63	0.28	0.32	3.48	2.37	4.19	4.47
Uruguay	5.54	4.26	3.44	0.48	0.25	0.51	5.29	2.55	6.03	4.89
Canada	2.95	3.49	3.68	0.36	0.93	0.29	5.02	2.64	5.12	3.31
United States	3.12	3.32	3.70	0.39	0.56	0.30	4.71	2.66	5.41	3.56
Antigua and Barbuda	1.59	2.25	2.21	0.58	0.09	0.15	3.10	2.37	4.66	4.49
The Bahamas	1.88	1.81	2.15	0.60	0.22	0.34	3.73	4.03	4.86	5.51
Barbados	2.28	2.24	2.15	0.57	0.18	0.33	4.90	4.32	5.56	5.82
Belize	1.59	1.84	2.15	0.36	0.49	0.45	1.99	1.12	3.86	4.37
Cuba	1.75	3.61	3.61	0.52	0.12	0.59	3.45	2.11	4.18	5.10
Dominica	2.14	3.12	1.66	0.57	0.31	0.50	6.12	4.21	7.04	7.64
Dominican Republic	0.92	1.20	2.12	0.44	0.09	0.24	1.96	1.46	4.41	4.94
Grenada	1.35	2.38	2.33	0.80	0.24	0.25	6.63	3.19	5.84	6.07
Guyana	1.49	1.40	1.28	0.57	0.30	0.38	2.12	1.16	3.91	4.83
Haiti	1.46	3.08	2.04	0.77	0.33	0.98	4.35	1.94	7.23	6.92
Jamaica	1.15	2.15	1.58	0.66	0.17	0.25	5.00	3.12	5.75	5.05
Saint Lucia	1.45	2.95	1.94	0.70	0.17	0.46	4.57	3.31	4.88	5.01
Saint Vincent and the Grenadines	1.51	2.29	2.07	0.79	0.17	0.56	6.28	2.10	5.90	6.25
Suriname	1.54	1.70	3.70	0.59	0.18	0.55	3.43	2.11	4.17	6.14
Trinidad and Tobago	1.65	1.59	1.43	0.47	0.14	0.30	3.25	2.61	4.28	4.68
Bolivia	2.06	2.32	2.78	1.33	0.41	0.63	5.49	1.74	6.15	6.09
Ecuador	1.68	1.29	2.97	1.27	0.19	0.47	4.07	1.49	5.60	4.87
Peru	1.82	1.23	2.55	0.77	0.19	0.22	3.60	1.44	4.23	3.85
Colombia	1.19	1.24	2.53	0.66	0.19	0.36	2.77	1.36	4.56	3.71
Costa Rica	1.82	2.21	2.77	0.59	0.14	0.71	4.47	2.33	6.28	4.08
El Salvador	1.21	1.24	2.97	0.53	0.13	0.51	2.31	1.17	6.33	3.36
Guatemala	1.16	0.93	1.88	0.54	0.11	0.38	1.83	0.78	5.19	3.07
Honduras	1.16	1.56	1.96	0.80	0.26	0.15	2.29	1.93	9.82	4.45
Mexico	2.43	1.34	2.21	0.76	0.33	0.50	2.69	1.21	4.54	3.92

Nicaragua	1.06	0.92	1.63	0.52	0.08	0.25	1.76	0.62	3.53	3.25
Panama	1.49	1.22	2.72	0.51	0.14	0.26	2.38	1.42	4.15	4.67
Venezuela	2.30	1.74	1.98	0.58	0.13	0.51	2.75	1.54	4.25	4.56
Brazil	1.77	2.26	4.52	0.49	0.37	0.32	2.75	1.52	4.00	4.97
Paraguay	1.58	1.39	1.66	0.78	0.20	0.40	2.67	0.90	4.74	5.41
Algeria	0.66	1.90	1.73	0.58	0.13	0.81	3.41	1.35	3.42	4.46
Bahrain	1.26	3.00	1.50	0.45	0.37	0.28	2.38	1.35	3.95	2.61
Egypt	0.83	6.18	2.98	0.33	0.22	1.17	1.86	0.82	4.08	4.38
Iran	1.15	2.53	4.28	0.41	0.73	0.37	2.03	1.01	6.31	3.98
Iraq	0.89	3.36	4.16	0.42	0.16	0.21	1.26	0.61	4.28	3.06
Jordan	1.02	3.11	2.40	0.48	0.22	0.11	3.78	1.06	6.66	3.65
Kuwait	0.96	2.36	1.85	0.39	0.23	0.14	2.67	0.75	3.15	2.01
Lebanon	1.34	8.69	3.83	0.61	0.10	1.00	9.02	2.56	7.63	7.94
Libya	1.69	5.30	5.27	0.50	0.28	1.38	5.72	1.91	7.17	7.04
Morocco	0.65	1.77	2.32	0.64	0.16	1.08	4.78	0.87	2.44	5.21
Palestine	1.36	2.81	6.03	0.48	0.07	0.42	2.22	1.29	6.41	2.93
Oman	1.01	1.97	1.91	0.44	0.34	0.43	4.38	1.76	4.10	3.80
Qatar	1.37	3.20	2.52	0.36	0.15	0.14	2.69	1.06	5.63	3.04
Saudi Arabia	1.31	1.97	2.41	0.75	0.08	0.49	4.92	1.38	4.66	3.92
Syria	0.74	1.84	4.12	0.19	0.13	0.09	1.65	0.68	13.72	2.47
Tunisia	0.82	4.19	2.06	0.44	0.12	0.63	3.82	1.40	3.72	4.33
Turkey	1.57	3.20	5.06	0.42	0.75	0.30	2.75	1.84	5.29	2.81
United Arab Emirates	1.95	4.45	6.02	0.55	0.23	0.46	3.56	1.43	5.94	47.67
Yemen	0.68	3.25	3.03	0.47	0.19	0.71	2.08	0.74	6.54	4.61
Afghanistan	1.07	3.62	5.00	0.79	0.58	1.54	3.10	1.00	9.74	6.02
Bangladesh	0.47	1.08	3.11	0.43	0.10	0.37	1.53	0.78	2.93	3.37
Bhutan	0.72	1.57	2.30	0.55	0.17	0.41	2.23	1.07	3.45	4.42
India	0.63	1.40	2.19	0.60	0.22	0.42	2.39	0.96	3.45	4.37
Nepal	0.62	1.48	1.85	0.58	0.16	0.47	1.99	0.99	3.40	4.02
Pakistan	0.73	5.20	3.13	1.34	0.15	1.53	6.45	1.91	4.78	7.64
Angola	1.12	3.34	1.57	0.41	0.17	0.46	2.48	1.35	3.64	5.25
Central African	0.85	2.63	1.73	0.44	0.23	0.79	2.20	1.09	4.12	5.50
Congo	1.28	4.52	2.19	0.46	0.27	0.51	2.75	1.32	4.12	6.32
Democratic Republic of the Congo	0.70	2.53	1.41	0.37	0.14	0.49	1.86	1.02	3.54	4.91
Equatorial Guinea	1.57	2.59	1.63	0.42	0.13	0.26	2.82	1.74	2.97	4.64
Gabon	1.43	4.86	2.20	0.40	0.20	0.40	2.81	1.57	3.60	5.50
Burundi	0.65	2.01	1.68	0.64	0.14	0.83	4.88	1.57	3.23	8.06
Comoros	0.78	2.58	2.04	0.60	0.14	0.80	5.06	1.75	3.85	10.09
Djibouti	1.04	3.40	2.27	0.63	0.14	0.75	5.81	1.98	3.84	9.91
Eritrea	0.97	3.43	2.92	0.87	0.25	1.34	6.83	1.67	5.39	13.83
Ethiopia	0.87	2.43	2.63	1.45	0.13	1.25	5.59	1.17	5.84	5.57
Kenya	0.52	1.08	1.62	0.26	0.13	0.47	3.33	1.99	2.81	8.83
Madagascar	0.58	1.96	1.59	0.54	0.10	0.77	4.22	1.31	3.18	8.21
Malawi	0.84	6.63	1.10	0.55	0.11	0.46	11.79	1.22	2.35	14.69
Mauritius	1.12	1.88	1.67	0.40	0.09	0.25	1.81	0.95	3.64	3.55
Mozambique	0.79	2.51	3.02	0.71	0.14	1.01	4.42	1.87	4.59	3.84
Rwanda	0.72	1.99	1.82	0.57	0.12	0.65	5.05	1.76	3.48	8.88
Seychelles	1.79	4.58	4.23	0.30	0.19	0.43	5.18	1.84	6.53	3.26
Somalia	0.81	2.85	1.86	0.71	0.17	1.30	5.56	1.57	4.49	10.49
Tanzania	0.84	2.31	2.23	0.59	0.13	0.71	5.44	1.84	3.68	9.31
Uganda	0.68	2.21	1.43	0.74	0.10	0.84	8.24	2.08	2.56	16.78
Zambia	0.91	3.47	2.68	0.64	0.20	0.87	5.93	1.92	3.90	9.97
Botswana	1.01	2.27	1.58	0.41	0.54	0.24	2.72	1.64	3.53	5.24
Lesotho	1.15	2.38	1.99	0.59	1.22	0.62	3.19	1.58	4.90	6.96
Namibia	0.81	1.65	1.72	0.39	0.73	0.52	4.43	1.27	2.76	11.17
South Africa	1.21	2.32	1.63	0.32	0.67	0.25	2.21	1.67	4.02	3.05
Swaziland	1.43	2.60	2.05	0.55	0.75	0.50	3.33	1.78	4.50	6.53
Zimbabwe	0.71	6.30	2.13	0.85	0.18	0.61	7.10	3.33	3.85	10.96

Benin	1.18	2.54	1.32	0.31	0.17	0.37	2.85	1.38	3.44	4.84
Burkina Faso	0.87	2.26	2.69	0.30	0.16	0.39	2.77	1.31	3.39	4.66
Cameroon	1.49	3.08	1.79	0.36	0.23	0.39	3.62	1.65	3.69	5.23
Cape Verde	1.64	2.30	2.75	0.19	0.07	0.08	2.50	1.26	3.39	4.20
Chad	0.81	2.74	1.19	0.29	0.14	0.45	2.67	1.12	3.48	4.68
Cote d'Ivoire	0.78	2.91	0.85	0.33	0.22	0.44	5.03	1.87	2.79	3.68
The Gambia	0.89	1.85	0.84	0.33	0.17	0.43	4.47	0.81	3.13	4.84
Ghana	0.94	2.73	2.80	0.20	0.17	0.13	4.60	1.47	3.18	3.60
Guinea	0.92	4.83	0.95	0.43	0.23	0.63	2.62	0.88	3.09	5.68
Guinea-Bissau	1.30	2.94	1.50	0.38	0.31	0.52	3.36	1.51	3.91	5.18
Liberia	1.05	2.43	1.11	0.29	0.15	0.33	2.60	1.28	3.13	4.25
Mali	0.79	8.28	0.65	0.47	0.13	0.54	2.22	1.14	2.84	5.67
Mauritania	1.25	3.08	1.41	0.31	0.16	0.28	2.90	1.51	3.52	5.03
Niger	0.69	1.74	0.98	0.25	0.11	0.38	2.20	1.07	3.20	4.32
Nigeria	0.84	1.27	1.51	0.23	0.09	1.71	3.00	1.48	2.91	6.14
Sao Tome and Principe	0.97	5.02	0.82	0.30	0.13	0.07	3.07	1.29	3.13	15.02
Senegal	1.08	3.18	1.39	0.30	0.20	0.41	2.90	1.34	3.85	5.27
Sierra Leone	0.92	2.60	1.34	0.31	0.18	0.37	2.87	1.23	3.34	4.69
Togo	0.88	2.42	1.33	0.28	0.18	0.35	2.46	1.24	3.22	4.50
American Samoa	0.92	2.36	1.89	0.92	0.38	0.14	2.47	2.06	2.83	6.55
Bermuda	2.28	3.89	2.65	0.60	0.41	0.42	3.66	2.56	5.08	3.71
Greenland	5.16	3.63	2.11	0.59	1.13	0.26	3.64	1.45	4.43	4.91
Guam	1.70	2.25	1.68	0.62	0.27	0.18	3.63	1.66	4.44	3.20
Northern Mariana	1.50	1.74	1.72	0.47	0.11	0.11	2.20	2.24	4.16	3.03
Puerto Rico	1.55	2.02	1.92	0.34	0.18	0.39	3.49	2.49	4.54	3.41
Virgin Islands, U.S.	3.35	2.10	3.05	0.46	0.62	0.23	4.80	4.94	5.97	5.21
South Sudan	0.93	2.40	1.73	0.65	0.10	0.95	5.78	1.74	3.66	9.09
Sudan	0.84	3.36	2.93	0.45	0.13	0.59	2.21	0.84	6.01	4.04

Age-standardized Death in 1990

	Kidney cancer	Bladder cancer	Brain and nervous system cancer	Thyroid cancer	Mesothelioma	Hodgkin lymphoma	Non-Hodgkin lymphoma	Multiple myeloma	Leukemia	Other malignant neoplasms
China	0.64	2.29	3.72	0.38	0.13	0.58	1.90	0.60	6.16	4.41
North Korea	0.85	1.85	2.71	0.42	0.10	0.29	2.08	0.51	4.83	4.12
Taiwan	0.78	2.93	1.79	0.59	0.13	0.14	2.94	0.69	3.50	4.58
Cambodia	0.77	2.58	2.55	1.11	0.28	1.05	3.13	0.75	6.80	5.31
Indonesia	0.62	2.17	2.76	0.74	0.14	0.63	2.55	0.58	5.31	4.47
Laos	0.81	2.84	2.91	1.11	0.29	1.13	3.53	0.73	7.13	5.30
Malaysia	0.83	2.97	1.86	0.94	0.15	0.55	3.28	0.70	6.12	5.43
Maldives	0.94	3.54	2.18	1.69	0.99	0.32	3.18	0.84	5.44	3.65
Myanmar	0.96	3.31	3.28	1.26	0.91	1.24	3.70	0.86	8.47	6.56
Philippines	0.80	1.16	2.35	1.12	0.17	0.34	1.99	0.57	4.09	10.25
Sri Lanka	2.66	1.34	1.33	0.78	0.37	0.83	1.83	0.73	6.29	4.76
Thailand	0.66	2.58	2.20	0.81	0.44	0.46	2.51	0.64	6.95	4.37
Timor-Leste	0.62	2.10	2.22	0.82	0.15	0.86	2.65	0.62	6.17	4.86
Vietnam	0.54	1.31	2.59	1.00	0.17	0.59	3.73	0.56	3.59	6.49
Fiji	0.66	1.47	1.33	1.48	0.20	0.25	1.65	0.97	5.32	5.39
Kiribati	0.94	1.43	1.06	0.42	0.12	0.22	1.85	0.93	4.38	5.30
Marshall Islands	1.10	1.92	1.64	0.90	0.25	0.44	2.97	1.45	5.62	5.75
Federated States of Micronesia	0.95	2.24	1.66	0.83	0.31	0.46	2.73	1.24	5.96	5.93
Papua New Guinea	0.65	1.60	1.69	0.71	0.27	0.60	2.50	0.86	6.03	5.68
Samoa	0.65	1.65	1.59	0.77	0.19	0.57	4.24	1.32	4.95	5.59
Solomon Islands	0.73	1.64	1.39	0.65	0.27	0.48	2.31	0.98	5.39	5.27
Tonga	0.99	1.81	1.40	0.60	0.16	0.22	4.69	1.62	3.66	4.13
Vanuatu	0.94	2.51	1.83	0.76	0.30	0.64	3.12	1.00	7.84	7.72
Armenia	0.52	5.95	4.71	0.22	0.54	0.15	1.45	0.50	5.01	13.99
Azerbaijan	2.62	2.29	3.60	0.39	0.09	0.77	1.40	0.55	4.11	5.57
Georgia	1.62	2.42	2.29	0.30	0.09	1.39	1.34	0.60	4.13	3.40
Kazakhstan	2.89	2.74	0.89	0.86	0.23	0.62	2.45	0.88	4.27	10.17
Kyrgyzstan	0.93	1.91	1.44	0.55	0.07	0.53	1.60	0.49	3.86	5.38
Mongolia	1.18	2.17	1.68	0.48	0.30	0.28	2.94	0.55	2.91	17.23
Tajikistan	1.51	1.35	4.72	0.18	0.15	0.26	2.16	0.57	4.00	5.53
Turkmenistan	1.96	1.14	0.98	0.45	0.10	0.58	1.19	0.67	3.68	5.79
Uzbekistan	0.89	1.26	2.55	0.14	0.10	0.56	1.71	0.44	4.00	3.90
Albania	1.32	0.94	4.51	0.51	0.10	0.76	1.52	0.54	4.20	8.39
Bosnia and	1.86	2.91	5.04	0.49	0.17	0.60	1.49	0.54	3.90	5.73
Bulgaria	1.04	3.13	4.12	0.49	0.07	0.87	1.63	0.60	4.11	4.46
Croatia	1.31	4.21	5.04	0.77	0.29	0.80	2.89	1.51	6.33	6.45
Czech Republic	4.13	4.81	4.19	0.83	0.30	1.32	3.86	2.15	7.56	7.04
Hungary	4.67	5.25	5.32	0.87	0.22	1.01	3.51	1.59	6.78	7.02
Macedonia	0.74	3.78	4.67	0.46	0.08	0.75	1.75	0.65	4.25	6.67
Montenegro	2.05	3.30	6.13	0.60	0.12	1.22	1.90	0.76	5.46	2.60
Poland	3.65	4.68	4.90	0.91	0.19	1.19	2.52	1.21	5.72	6.63
Romania	1.73	3.47	3.60	0.50	0.18	0.85	1.99	0.80	4.31	6.23
Serbia	2.57	4.64	6.35	0.48	0.19	0.99	2.59	1.02	5.93	5.77
Slovakia	1.55	4.17	4.22	0.65	0.22	0.88	2.80	1.58	5.27	8.83
Slovenia	2.03	4.14	3.66	0.61	0.47	0.73	3.20	1.84	6.13	4.75
Belarus	1.03	3.13	2.49	0.45	0.14	0.85	1.64	0.81	6.15	7.27
Estonia	1.28	3.48	3.56	0.54	0.13	0.83	2.51	1.36	6.15	5.78
Latvia	1.25	3.91	3.51	0.49	0.19	0.71	1.98	1.27	6.33	5.77
Lithuania	1.50	4.05	3.63	0.49	0.28	0.77	1.73	1.50	7.00	5.79

Moldova	1.76	3.17	3.09	0.45	0.11	1.15	2.25	0.79	5.30	5.48
Russian Federation	3.39	3.03	2.97	0.50	0.25	0.83	2.13	0.99	4.54	6.59
Ukraine	2.97	3.04	3.22	0.44	0.19	0.76	1.69	0.71	6.09	5.99
Brunei	2.29	3.49	3.16	0.99	0.39	0.45	6.14	1.77	5.75	4.88
Japan	1.63	2.28	0.85	0.60	0.34	0.11	3.24	1.39	4.37	3.39
South Korea	0.88	2.59	2.52	0.42	0.14	0.11	1.94	0.85	4.93	4.47
Singapore	1.16	1.77	1.13	0.54	0.54	0.21	2.65	0.93	4.12	3.23
Australia	3.31	3.67	4.57	0.36	2.02	0.45	5.92	2.50	9.76	5.00
New Zealand	3.05	3.90	4.44	0.42	1.56	0.43	5.51	2.69	7.13	4.97
Andorra	2.69	5.70	4.30	0.42	2.08	0.48	4.86	2.77	6.48	4.44
Austria	4.60	4.42	3.41	0.79	0.72	0.83	3.49	1.86	5.45	6.20
Belgium	3.40	6.01	5.54	0.88	1.13	0.67	4.04	2.34	8.04	5.77
Cyprus	0.99	4.83	4.03	0.66	1.08	0.71	5.27	2.30	7.03	5.04
Denmark	3.20	6.91	5.90	0.32	1.06	0.59	4.33	2.31	10.46	4.79
Finland	3.72	3.38	4.40	0.61	0.94	0.60	5.27	2.60	8.02	4.32
France	3.40	5.38	3.47	0.66	1.09	0.63	4.57	2.43	7.71	8.39
Germany	3.63	5.01	3.96	0.75	0.89	0.75	3.58	1.99	7.14	4.60
Greece	2.19	5.61	5.73	0.45	0.33	1.10	2.16	1.43	6.69	5.17
Iceland	4.78	4.58	5.81	1.26	0.71	0.44	3.37	2.76	7.42	5.07
Ireland	2.82	3.99	4.90	0.55	0.88	0.71	4.80	3.00	7.13	4.89
Israel	2.64	4.12	3.39	0.65	0.54	0.65	5.05	2.33	8.33	3.65
Italy	2.93	6.32	4.07	0.65	1.35	0.82	4.35	2.29	7.06	5.80
Luxembourg	2.06	5.15	5.31	0.96	0.96	0.55	3.96	2.31	8.36	5.80
Malta	2.73	5.69	3.55	0.73	0.91	0.84	3.90	1.74	5.88	5.37
Netherlands	3.50	5.52	3.33	0.45	1.91	0.60	5.37	2.83	6.41	5.85
Norway	3.37	5.09	4.71	0.49	0.89	0.47	4.84	3.42	5.95	4.32
Portugal	1.97	4.67	4.13	0.80	0.28	0.65	3.30	1.74	6.28	6.06
Spain	2.00	6.16	3.58	0.47	0.56	0.70	3.35	1.84	6.07	5.22
Sweden	4.41	3.78	4.32	0.47	1.00	0.37	5.13	2.75	5.03	5.44
Switzerland	2.38	3.65	2.92	0.50	1.13	0.41	5.08	2.35	5.74	3.39
United Kingdom	3.13	6.09	3.97	0.40	2.23	0.65	4.78	2.48	6.98	5.14
Argentina	4.78	4.19	2.85	0.70	0.25	0.77	3.61	1.53	5.76	8.10
Chile	3.76	2.65	1.73	0.73	0.37	0.65	3.52	1.83	4.81	7.08
Uruguay	5.53	5.12	3.08	0.38	0.22	0.84	4.76	1.99	7.19	7.70
Canada	2.45	4.38	4.02	0.39	1.14	0.56	6.35	2.86	7.08	4.24
United States	3.21	3.41	3.83	0.35	0.70	0.55	6.19	2.75	6.69	3.92
Antigua and Barbuda	1.97	2.22	0.58	0.50	0.14	0.20	3.50	2.19	5.39	6.39
The Bahamas	2.35	1.74	0.74	0.59	0.25	0.49	4.13	3.80	5.45	7.19
Barbados	3.16	2.24	0.51	0.59	0.21	0.51	5.64	3.97	6.29	8.22
Belize	1.40	1.63	0.58	0.30	0.39	0.57	1.77	0.86	3.94	4.87
Cuba	2.21	3.59	0.74	0.43	0.15	0.16	4.38	2.17	5.88	7.81
Dominica	2.16	2.63	0.46	0.47	0.22	0.67	5.58	3.40	6.41	8.94
Dominican Republic	1.12	1.17	0.82	0.45	0.08	0.14	1.58	1.01	4.31	5.90
Grenada	1.66	2.41	0.61	1.16	0.46	0.53	9.80	2.70	7.23	10.93
Guyana	1.70	1.38	0.49	0.50	0.31	0.23	2.15	0.92	4.10	6.26
Haiti	1.78	3.06	1.50	0.84	0.44	1.33	4.99	1.82	8.25	8.23
Jamaica	1.54	2.54	0.52	0.37	0.17	0.32	3.52	1.76	3.54	5.80
Saint Lucia	2.03	3.39	0.55	0.84	0.23	0.65	5.94	3.33	6.39	7.85
Saint Vincent and the Grenadines	1.99	2.13	0.58	0.76	0.18	0.32	6.06	1.79	6.10	8.12
Suriname	1.80	1.59	0.75	0.53	0.18	0.61	3.55	1.79	4.63	8.17
Trinidad and Tobago	2.82	1.98	0.52	0.56	0.16	0.29	4.14	2.07	5.77	7.62
Bolivia	1.55	2.35	2.01	1.34	0.66	1.27	5.15	1.72	6.84	7.06
Ecuador	1.31	1.48	0.82	0.59	0.16	0.76	2.86	1.43	4.72	6.71
Peru	1.73	1.56	1.26	0.71	0.84	0.43	3.57	1.66	4.83	6.06
Colombia	0.98	2.05	2.18	0.95	0.23	0.73	3.20	1.46	6.46	4.02
Costa Rica	1.13	2.27	1.41	0.68	0.17	1.26	4.47	2.02	6.83	4.69
El Salvador	0.84	1.14	1.11	1.07	0.13	1.06	1.50	1.01	6.44	4.24
Guatemala	1.03	1.19	0.59	0.61	0.14	0.76	1.30	0.76	3.95	4.61

Honduras	0.76	1.30	1.58	0.79	0.18	0.24	1.83	1.49	9.61	4.81
Mexico	1.85	1.53	1.71	0.70	0.27	0.89	2.29	1.01	4.93	4.02
Nicaragua	1.04	1.03	0.96	0.40	0.06	0.41	1.62	0.68	4.15	4.25
Panama	0.88	1.55	1.57	0.49	0.14	0.29	2.83	1.58	5.36	5.71
Venezuela	2.45	1.87	0.56	0.47	0.15	0.71	3.39	1.59	5.33	7.50
Brazil	1.29	2.61	2.52	0.60	0.45	0.60	2.73	1.10	4.77	5.93
Paraguay	1.48	1.32	1.04	0.62	0.15	0.49	2.03	0.77	4.98	5.89
Algeria	0.51	2.08	1.62	0.53	0.05	1.39	3.36	1.05	3.62	4.77
Bahrain	1.85	6.85	2.19	0.60	0.22	1.09	3.24	1.89	6.69	4.81
Egypt	0.59	6.67	2.46	0.31	0.08	1.91	1.63	0.66	4.34	4.44
Iran	0.80	2.19	3.71	0.32	0.26	0.43	1.67	0.75	6.89	3.29
Iraq	1.07	5.01	5.49	0.64	0.28	0.72	3.57	0.82	7.16	5.44
Jordan	0.63	2.96	2.43	0.67	0.30	0.24	4.84	1.36	8.12	5.26
Kuwait	0.92	3.41	1.90	0.60	0.12	0.33	3.57	0.98	5.35	3.55
Lebanon	0.88	11.23	3.97	0.68	0.09	2.15	7.35	2.47	9.67	8.77
Libya	1.18	4.98	4.02	0.46	0.08	1.83	5.45	1.71	6.79	6.69
Morocco	0.45	1.63	1.98	0.66	0.07	1.70	4.41	0.68	2.54	5.40
Palestine	1.12	3.09	6.15	0.54	0.08	0.50	2.73	1.30	7.39	3.21
Oman	0.56	2.24	1.88	0.45	0.20	0.97	4.96	1.46	5.19	4.27
Qatar	2.71	4.50	3.47	1.36	0.07	0.24	4.16	1.03	8.77	4.88
Saudi Arabia	0.55	1.77	1.76	0.51	0.05	0.76	3.70	0.92	4.48	4.10
Syria	0.53	1.50	3.57	0.21	0.12	0.13	2.62	0.60	17.19	2.60
Tunisia	0.66	4.24	1.85	0.45	0.04	1.02	3.98	1.26	4.51	4.79
Turkey	1.52	4.20	7.98	0.62	1.48	0.74	3.88	1.99	8.04	4.29
United Arab Emirates	1.20	3.91	3.62	0.55	0.07	0.64	3.85	1.24	6.09	43.36
Yemen	0.47	2.72	2.72	0.47	0.09	1.03	2.13	0.65	6.11	4.00
Afghanistan	1.03	3.59	5.08	0.79	0.43	1.93	3.47	1.04	9.29	5.39
Bangladesh	0.50	1.58	3.15	0.58	0.22	1.35	1.85	0.79	4.41	4.06
Bhutan	0.58	1.55	2.37	0.64	0.18	1.16	2.05	0.91	4.18	4.16
India	0.44	1.23	1.95	0.50	0.17	0.84	1.79	0.75	3.58	3.35
Nepal	0.41	1.46	1.98	0.58	0.16	1.09	1.61	0.76	3.78	3.54
Pakistan	0.45	3.97	2.26	1.05	0.10	1.86	5.04	1.55	4.00	6.64
Angola	0.91	3.69	1.49	0.47	0.21	0.81	2.91	1.21	4.13	5.98
Central African	0.83	2.79	1.47	0.53	0.26	0.86	2.67	1.20	4.04	6.04
Congo	1.05	4.19	1.73	0.57	0.37	0.75	3.15	1.33	4.17	6.57
Democratic Republic of the Congo	0.73	3.04	1.26	0.41	0.16	0.61	2.29	1.07	3.55	5.36
Equatorial Guinea	0.97	2.63	1.47	0.55	0.27	0.89	2.84	1.29	4.05	5.79
Gabon	1.05	4.44	1.48	0.50	0.24	0.55	3.20	1.51	3.54	5.78
Burundi	0.74	3.26	1.94	0.80	0.32	1.36	7.25	1.86	3.65	10.96
Comoros	0.73	2.87	1.80	0.62	0.17	1.28	6.42	1.83	3.95	12.03
Djibouti	0.75	3.21	1.81	0.58	0.13	1.03	6.34	1.92	3.62	11.41
Eritrea	0.77	3.60	2.14	0.80	0.30	1.83	7.20	1.71	4.69	13.96
Ethiopia	0.98	2.49	3.41	2.24	0.30	2.53	7.60	1.20	6.74	6.99
Kenya	0.44	0.97	1.44	0.25	0.12	0.49	2.81	1.62	2.55	7.65
Madagascar	0.56	2.95	1.66	0.53	0.12	1.17	5.47	1.50	3.41	10.27
Malawi	0.79	7.35	0.99	0.57	0.12	0.62	12.42	1.05	2.35	13.64
Mauritius	0.78	3.14	1.15	0.44	0.12	0.35	1.28	0.60	4.60	3.35
Mozambique	0.64	3.51	2.53	0.63	0.16	1.51	4.23	1.75	5.18	4.17
Rwanda	0.76	3.02	1.97	0.74	0.25	1.33	7.43	1.85	3.82	11.31
Seychelles	0.91	5.18	4.49	0.46	0.23	0.63	3.38	1.01	5.96	6.19
Somalia	0.73	2.89	1.51	0.62	0.19	1.29	5.90	1.64	3.61	10.27
Tanzania	0.69	2.77	1.97	0.59	0.13	0.91	5.88	1.83	3.31	10.17
Uganda	0.49	2.03	0.93	0.56	0.11	0.95	7.93	1.78	2.34	14.92
Zambia	0.95	4.05	3.00	0.75	0.19	1.27	7.95	2.15	4.35	13.42
Botswana	0.75	2.34	1.30	0.41	0.66	0.45	2.59	1.46	4.04	5.87
Lesotho	0.70	1.88	1.25	0.46	0.95	0.55	2.44	1.46	4.13	5.82
Namibia	0.71	1.86	1.67	0.43	1.00	0.91	4.33	1.28	3.27	9.82
South Africa	0.99	2.52	1.39	0.34	0.62	0.33	2.14	1.51	4.48	3.51

Swaziland	1.07	2.67	1.49	0.56	0.83	0.56	3.25	1.85	4.46	6.26
Zimbabwe	0.61	5.81	1.41	0.64	0.09	0.40	4.46	2.34	3.19	7.89
Benin	0.74	3.71	0.91	0.34	0.19	0.52	2.91	0.97	3.42	5.36
Burkina Faso	0.64	4.01	2.56	0.39	0.25	0.57	3.00	1.01	3.56	5.48
Cameroon	1.09	3.70	1.10	0.43	0.29	0.50	3.93	1.34	3.55	5.68
Cape Verde	0.93	1.66	2.11	0.17	0.06	0.11	1.86	0.75	2.54	3.40
Chad	0.54	2.76	0.75	0.31	0.20	0.48	2.43	0.87	3.02	4.57
Cote d'Ivoire	0.56	2.56	0.65	0.33	0.26	0.54	4.57	1.62	2.59	3.36
The Gambia	0.63	1.52	0.53	0.31	0.18	0.54	3.80	0.69	2.80	4.27
Ghana	0.88	3.92	2.22	0.19	0.32	0.16	5.78	0.96	4.05	4.36
Guinea	0.69	3.98	0.74	0.40	0.24	0.78	2.59	0.75	3.05	5.69
Guinea-Bissau	0.91	3.99	1.08	0.46	0.42	0.75	3.67	1.13	3.87	5.67
Liberia	0.85	3.82	0.90	0.35	0.16	0.49	3.19	1.01	3.26	5.10
Mali	0.64	8.71	0.54	0.59	0.27	1.03	2.36	0.88	3.05	5.95
Mauritania	0.89	3.84	0.98	0.38	0.31	0.46	3.09	1.16	3.41	5.37
Niger	0.62	2.67	0.87	0.32	0.18	0.61	2.77	0.86	3.44	5.25
Nigeria	0.72	1.25	0.97	0.26	0.15	2.55	3.01	1.42	2.87	5.81
Sao Tome and Principe	0.65	3.46	0.53	0.26	0.11	0.09	2.57	0.76	2.82	12.82
Senegal	0.77	3.72	0.91	0.33	0.21	0.50	2.90	0.98	3.41	5.36
Sierra Leone	0.64	3.75	0.89	0.32	0.15	0.49	2.92	0.88	3.19	5.09
Togo	0.65	3.62	0.94	0.33	0.24	0.48	2.75	0.93	3.29	5.17
American Samoa	0.89	1.92	1.46	1.01	0.28	0.17	2.09	1.56	3.35	6.00
Bermuda	4.03	5.07	0.79	0.87	0.96	0.49	6.26	3.37	8.71	7.15
Greenland	5.31	4.45	2.47	0.56	1.11	0.68	5.85	1.67	6.05	5.86
Guam	1.61	1.77	1.09	0.41	0.18	0.19	3.87	1.46	5.04	3.19
Northern Mariana	1.41	1.57	1.54	0.56	0.11	0.13	2.01	2.23	4.99	3.13
Puerto Rico	1.78	2.30	0.77	0.60	0.22	0.66	4.54	2.46	6.03	6.52
Virgin Islands, U.S.	3.00	1.92	1.37	0.41	0.49	0.22	4.84	4.09	6.55	6.13
South Sudan	0.82	2.55	1.40	0.59	0.09	0.99	6.33	1.78	3.20	9.70
Sudan	0.60	3.94	3.00	0.47	0.06	0.96	2.34	0.72	6.28	3.92

Age-standardized Death change from 1990 to 2017(%)

	Kidney cancer	Bladder cancer	Brain and nervous system cancer	Thyroid cancer	Mesothelioma	Hodgkin lymphoma	Non-Hodgkin lymphoma	Multiple myeloma	Leukemia	Other malignant neoplasms
China	45.94	-23.53	-7.87	-1.08	13.48	-74.01	14.87	6.81	-38.90	-19.26
North Korea	20.57	-3.46	14.57	1.04	68.22	-8.10	4.69	4.43	2.82	-4.61
Taiwan	188.36	-7.55	8.41	-9.66	-4.12	-36.65	21.96	55.56	-3.12	-13.63
Cambodia	29.04	-0.90	-4.99	-22.00	-17.83	-49.66	-3.69	10.40	-10.35	-14.64
Indonesia	64.43	23.69	4.05	-6.43	36.08	-36.37	14.21	24.26	3.79	-2.63
Laos	33.68	-6.49	-9.31	-30.19	-33.96	-50.38	-9.87	6.09	-10.72	-14.60
Malaysia	46.97	-14.99	4.02	-8.87	12.86	-48.37	5.32	21.61	-16.69	-24.47
Maldives	-6.23	-32.43	-33.82	-40.36	-58.14	-73.06	-38.50	-4.17	-38.97	-32.94
Myanmar	38.23	-10.02	-5.26	-26.88	-49.45	-53.51	-5.11	11.68	-13.63	-16.22
Philippines	54.12	-5.32	11.00	38.90	1.79	-40.99	34.45	15.77	30.78	-46.49
Sri Lanka	-16.95	-3.16	49.91	-31.27	-13.24	-57.16	19.32	15.38	-19.67	-5.10
Thailand	21.71	-38.74	24.72	-36.30	-43.88	-58.65	-34.67	-5.65	-22.12	-26.34
Timor-Leste	54.20	28.07	10.42	-10.44	15.84	-40.48	12.80	14.63	8.01	2.82
Vietnam	20.80	-7.69	17.25	14.67	5.86	-39.13	-9.76	9.61	0.98	-9.83
Fiji	16.28	22.65	10.97	-11.89	50.35	6.95	38.31	25.01	-8.48	-4.22
Kiribati	39.73	11.50	-1.27	-0.15	37.18	-5.00	10.34	5.87	-2.54	13.92
Marshall Islands	32.57	30.26	32.66	10.40	38.71	-15.77	17.48	15.69	3.64	4.72
Federated States of Micronesia	16.66	3.88	8.52	0.75	20.58	-31.40	1.19	13.11	-12.52	-10.68
Papua New Guinea	13.73	17.90	10.52	5.26	15.49	-19.00	5.29	10.61	-4.27	-1.08
Samoa	14.19	-7.28	-7.35	-0.39	6.10	-32.72	1.63	6.11	-12.30	-8.40
Solomon Islands	15.44	13.67	7.73	8.16	10.49	-19.92	1.88	12.06	-7.34	-6.51
Tonga	35.83	7.24	23.66	14.08	18.26	-10.66	9.20	5.60	0.08	1.98
Vanuatu	32.40	32.22	24.66	21.34	39.95	-0.62	15.93	11.03	9.15	10.26
Armenia	396.58	-18.59	12.56	184.57	-2.25	35.09	17.17	30.73	-19.87	-40.04
Azerbaijan	11.77	8.34	11.72	0.34	18.04	-28.29	31.80	27.77	12.55	-0.45
Georgia	54.78	56.07	99.12	74.14	360.96	-34.43	104.74	33.92	28.73	95.59
Kazakhstan	-1.42	-24.50	185.56	-45.62	3.35	-33.48	-42.17	-27.35	-20.98	-46.02
Kyrgyzstan	67.95	-26.86	60.41	-34.61	46.19	-54.91	-23.67	-5.20	-32.14	-18.46
Mongolia	61.20	-48.94	67.07	4.57	-59.52	-40.42	-28.69	-6.19	-8.31	-60.37
Tajikistan	22.14	-3.23	14.66	0.57	15.85	-15.60	2.81	7.28	-18.44	-4.15
Turkmenistan	40.18	23.55	285.63	-25.26	25.89	-20.66	27.63	42.85	-8.56	-10.47
Uzbekistan	63.81	27.80	53.26	47.41	-7.61	-17.68	22.77	33.31	-12.46	12.11
Albania	52.22	9.98	29.62	-18.56	29.65	-41.11	0.13	31.58	-1.94	-2.95
Bosnia and	68.33	36.61	35.74	-5.65	-18.70	-30.33	49.41	71.28	13.04	-6.14
Bulgaria	124.48	19.68	35.42	-20.91	15.10	-32.01	36.18	37.27	3.96	-3.19
Croatia	186.75	7.89	26.75	-45.38	168.55	-57.12	22.33	33.59	-22.33	-35.58
Czech Republic	34.96	-19.20	-1.24	-53.01	-17.04	-64.99	-19.70	-7.79	-29.70	-37.41
Hungary	-11.91	-13.19	-26.49	-49.89	-7.87	-67.67	-11.44	2.87	-20.87	-49.36
Macedonia	120.40	13.00	22.84	-32.56	-29.59	-35.59	-0.02	10.44	-2.31	-21.19
Montenegro	13.41	2.82	-1.85	-7.96	21.85	-35.93	0.95	9.75	-9.05	-8.33
Poland	25.64	14.73	4.50	-50.26	134.23	-62.26	22.25	69.34	-9.77	-32.07
Romania	53.57	16.52	40.73	-14.31	18.63	-51.06	23.56	38.12	2.37	0.14
Serbia	29.27	8.06	1.13	0.89	27.38	-40.97	14.65	43.75	-12.24	-12.75
Slovakia	148.41	-10.62	4.92	-34.12	-22.04	-46.52	25.40	28.10	-6.87	-38.98
Slovenia	67.53	-3.81	-3.08	-37.69	52.60	-54.95	27.11	24.23	-12.55	-20.16
Belarus	277.53	-18.88	31.44	18.00	35.32	-41.50	41.93	77.09	-24.16	-14.61
Estonia	247.37	-0.96	11.74	-3.53	53.54	-59.19	24.62	47.28	-10.97	-22.17
Latvia	256.33	22.75	29.45	17.25	23.58	-38.72	43.76	41.81	-15.96	-10.81
Lithuania	219.35	-0.97	26.82	-4.05	-33.39	-49.00	59.94	32.61	-17.08	-18.41

Moldova	31.49	-17.14	11.11	-14.34	-7.10	-44.49	7.04	-2.91	-40.74	-19.57
Russian Federation	9.66	-5.18	6.01	-2.46	6.01	-45.14	21.39	55.91	-7.03	50.38
Ukraine	33.56	5.57	48.28	12.96	49.79	15.91	51.97	38.90	-18.50	2.07
Brunei	28.71	-17.51	13.95	-8.24	-3.26	16.00	7.31	56.62	-1.02	10.54
Japan	18.64	-9.64	53.43	-20.09	27.26	-21.99	1.69	-5.86	-28.07	-10.47
South Korea	103.95	-23.86	-29.94	109.17	5.36	-28.84	28.89	42.60	-40.01	-37.18
Singapore	6.97	-39.41	21.17	-46.62	-45.69	-50.89	-19.30	-18.55	-39.65	-25.85
Australia	-1.03	-23.60	-8.61	11.83	-6.76	-46.02	-23.01	4.44	-40.79	-28.65
New Zealand	6.15	-26.88	-7.16	-12.21	-20.94	-41.88	-12.90	2.10	-21.03	-17.74
Andorra	0.98	-20.82	-6.68	-17.25	-6.20	-37.62	-14.26	-9.54	-13.33	-14.31
Austria	-32.69	-26.72	7.91	-46.45	-8.66	-69.85	6.04	13.52	-2.15	-29.48
Belgium	-5.98	-27.82	-29.89	-51.37	2.57	-55.40	-6.77	3.35	-27.32	-26.60
Cyprus	64.88	-15.87	-11.71	-38.09	-36.54	-59.24	-26.90	-4.73	-14.67	-28.70
Denmark	15.16	-25.39	-13.86	6.84	9.34	-41.23	-13.95	5.53	-36.19	-12.36
Finland	-2.69	-32.76	-11.94	-22.26	-13.82	-52.07	-12.89	-8.03	-46.14	-7.32
France	-4.42	-21.02	6.62	-42.41	13.78	-50.66	-17.40	-3.84	-27.09	-46.19
Germany	3.60	-28.33	5.74	-33.41	-3.20	-62.00	3.15	12.54	-21.51	-0.77
Greece	26.85	-9.05	6.98	-18.52	1.05	-20.15	-1.15	32.25	-2.99	-34.28
Iceland	9.33	-21.82	-7.69	-18.91	21.92	-51.01	-3.96	9.09	-28.49	-11.27
Ireland	19.23	-20.52	-13.45	-38.46	-34.37	-54.22	-11.23	-7.95	-21.52	-24.71
Israel	3.10	-8.50	34.68	-0.80	-31.07	-42.04	3.87	9.47	-26.31	-4.06
Italy	-1.39	-29.93	-12.61	-35.11	-13.86	-50.21	-14.22	1.72	-23.37	-31.45
Luxembourg	-9.60	-20.93	-1.15	-36.38	-9.69	-52.99	-2.53	5.45	-22.97	-24.30
Malta	6.77	-28.24	10.33	-25.43	11.40	-43.18	3.13	13.11	-16.06	-24.53
Netherlands	12.74	-15.54	11.47	-7.33	-6.92	-47.03	-18.90	-2.67	-11.76	-21.52
Norway	18.58	-24.15	-7.42	-12.59	-11.43	-49.49	-16.83	-2.03	-22.25	-14.93
Portugal	-0.41	-15.74	10.32	-46.63	6.77	-52.14	14.75	28.85	-24.13	-35.07
Spain	30.90	-14.85	4.06	-19.70	-6.32	-55.20	-7.46	8.51	-24.87	-29.44
Sweden	-15.47	-5.20	-13.53	-18.23	-24.22	-42.57	-25.39	-2.54	-13.87	-33.61
Switzerland	0.19	-17.07	8.10	-17.09	-6.39	-45.31	-31.69	-5.04	-21.60	-6.28
United Kingdom	15.41	-27.74	4.86	-16.15	-4.62	-46.23	-8.16	2.85	-22.26	-23.95
Argentina	-9.53	-28.46	12.74	-31.48	69.59	-41.28	-2.41	-1.45	-17.93	-39.49
Chile	4.55	-2.78	34.16	-13.94	-24.52	-51.61	-1.14	29.27	-12.85	-36.84
Uruguay	0.19	-16.78	11.59	24.77	16.81	-38.99	11.23	27.84	-16.02	-36.45
Canada	20.10	-20.17	-8.52	-5.91	-18.99	-47.55	-20.88	-7.72	-27.63	-21.99
United States	-2.65	-2.57	-3.43	9.80	-19.81	-45.49	-23.86	-3.20	-19.18	-9.00
Antigua and Barbuda	-19.47	1.15	279.57	16.68	-37.52	-27.12	-11.46	8.43	-13.48	-29.73
The Bahamas	-19.99	3.88	189.42	1.81	-12.45	-30.01	-9.57	6.06	-10.72	-23.37
Barbados	-27.85	-0.04	319.53	-3.01	-12.73	-35.39	-13.05	8.72	-11.54	-29.24
Belize	13.61	12.52	268.45	22.15	25.22	-22.09	12.31	29.21	-2.00	-10.29
Cuba	-20.75	0.39	385.21	21.80	-20.58	266.75	-21.18	-2.75	-28.91	-34.80
Dominica	-1.09	18.59	258.84	22.73	41.76	-25.74	9.66	23.79	9.80	-14.50
Dominican Republic	-17.30	2.29	159.52	-2.98	12.40	70.08	24.26	44.99	2.14	-16.27
Grenada	-18.32	-1.59	279.63	-31.52	-47.73	-52.42	-32.30	17.91	-19.23	-44.41
Guyana	-12.75	1.61	160.72	15.31	-4.92	67.13	-1.24	26.63	-4.62	-22.92
Haiti	-18.36	0.61	36.14	-8.11	-25.36	-26.05	-12.93	6.67	-12.43	-15.91
Jamaica	-25.60	-15.13	201.76	75.82	0.22	-21.61	42.07	76.78	62.50	-12.93
Saint Lucia	-28.82	-12.89	253.15	-16.93	-25.37	-29.23	-23.10	-0.40	-23.62	-36.20
Saint Vincent and the Grenadines	-23.80	7.78	259.03	4.24	-6.72	75.80	3.72	17.67	-3.31	-23.02
Suriname	-14.44	6.87	394.24	9.74	-4.87	-9.46	-3.44	17.82	-10.01	-24.87
Trinidad and Tobago	-41.39	-19.95	173.64	-16.06	-6.72	1.62	-21.59	26.00	-25.69	-38.54
Bolivia	32.58	-1.12	38.39	-0.35	-38.14	-50.28	6.59	1.33	-10.06	-13.69
Ecuador	28.08	-12.43	260.81	117.23	15.29	-37.63	42.17	4.29	18.60	-27.46
Peru	5.09	-21.43	102.01	7.09	-77.20	-48.27	0.73	-13.06	-12.43	-36.56
Colombia	21.11	-39.21	15.80	-30.31	-15.63	-51.02	-13.40	-6.72	-29.36	-7.73
Costa Rica	60.35	-2.82	97.24	-13.44	-13.56	-43.84	-0.07	15.27	-8.07	-13.03
El Salvador	44.25	8.82	166.28	-50.55	0.72	-52.14	54.44	15.62	-1.68	-20.82
Guatemala	12.96	-22.51	217.62	-11.65	-16.98	-50.30	40.48	2.35	31.47	-33.33

Honduras	53.36	19.94	24.25	1.24	45.58	-35.26	24.96	29.10	2.17	-7.58
Mexico	31.94	-12.31	28.96	8.05	22.95	-43.79	17.72	18.77	-8.03	-2.58
Nicaragua	2.39	-10.62	70.53	28.50	28.26	-38.31	8.72	-9.09	-14.98	-23.59
Panama	69.57	-21.04	73.30	5.01	-1.54	-12.02	-16.02	-9.71	-22.60	-18.11
Venezuela	-6.29	-7.21	252.31	24.84	-13.93	-27.95	-18.91	-2.69	-20.13	-39.24
Brazil	37.14	-13.23	79.46	-18.14	-17.07	-47.09	0.58	38.31	-16.09	-16.19
Paraguay	6.93	5.60	58.84	26.25	37.91	-18.79	31.90	17.59	-4.89	-8.14
Algeria	29.68	-8.48	7.31	8.88	133.95	-41.62	1.41	28.71	-5.52	-6.57
Bahrain	-31.83	-56.16	-31.35	-24.61	69.92	-74.35	-26.59	-28.70	-40.97	-45.69
Egypt	40.93	-7.32	21.14	7.20	158.47	-38.51	14.28	23.79	-6.02	-1.52
Iran	44.52	15.09	15.21	28.49	176.68	-15.37	21.94	35.37	-8.39	20.95
Iraq	-16.19	-32.83	-24.10	-34.71	-42.31	-71.43	-64.62	-25.39	-40.19	-43.68
Jordan	62.14	4.98	-1.30	-28.67	-27.48	-56.17	-21.95	-22.33	-17.97	-30.57
Kuwait	4.16	-30.78	-2.93	-35.29	87.41	-56.85	-25.28	-23.44	-41.15	-43.34
Lebanon	51.33	-22.61	-3.52	-9.49	18.77	-53.74	22.60	3.54	-21.09	-9.49
Libya	42.87	6.50	30.92	10.08	237.95	-24.55	4.98	11.87	5.47	5.32
Morocco	42.29	8.66	17.08	-2.78	151.31	-36.56	8.16	27.00	-3.71	-3.55
Palestine	21.58	-8.99	-2.06	-10.82	-20.56	-15.82	-18.71	-0.94	-13.30	-8.83
Oman	82.11	-11.87	1.50	-1.33	73.72	-55.66	-11.67	20.17	-21.02	-11.06
Qatar	-49.42	-28.83	-27.27	-73.82	102.65	-41.68	-35.36	3.07	-35.84	-37.81
Saudi Arabia	138.68	10.85	37.09	46.93	83.55	-34.84	32.86	49.52	3.91	-4.30
Syria	40.23	22.17	15.52	-8.33	8.66	-32.66	-36.87	13.31	-20.21	-5.29
Tunisia	24.68	-1.13	11.32	-2.81	171.63	-38.69	-4.07	10.58	-17.38	-9.62
Turkey	3.07	-23.84	-36.52	-31.75	-49.02	-59.67	-29.14	-7.55	-34.14	-34.46
United Arab Emirates	63.34	13.97	66.12	0.03	248.16	-27.60	-7.52	14.86	-2.50	9.95
Yemen	43.94	19.42	11.45	0.22	116.80	-30.39	-2.24	14.04	7.01	15.37
Afghanistan	4.01	1.06	-1.65	1.16	34.76	-20.45	-10.50	-3.74	4.78	11.54
Bangladesh	-5.38	-31.52	-1.29	-26.19	-55.08	-72.37	-17.60	-0.91	-33.63	-16.98
Bhutan	24.71	0.99	-2.87	-14.37	-6.17	-64.29	8.62	17.07	-17.46	6.23
India	42.07	14.37	12.35	20.80	31.41	-50.05	33.65	27.67	-3.68	30.34
Nepal	49.84	1.54	-6.48	0.43	1.56	-56.46	23.22	31.28	-10.00	13.60
Pakistan	62.73	31.12	38.75	28.36	46.53	-17.92	27.88	22.73	19.52	15.15
Angola	22.99	-9.49	5.37	-13.82	-21.29	-42.70	-14.70	11.35	-11.88	-12.28
Central African	3.26	-5.87	17.08	-18.00	-9.82	-8.29	-17.58	-9.09	1.84	-9.06
Congo	21.59	7.89	26.26	-19.14	-26.59	-31.76	-12.68	-1.08	-1.20	-3.70
Democratic Republic of the Congo	-3.43	-16.84	11.41	-10.76	-9.14	-19.28	-18.69	-4.99	-0.48	-8.46
Equatorial Guinea	61.94	-1.51	11.09	-24.32	-52.28	-70.25	-0.83	34.14	-26.57	-19.81
Gabon	36.67	9.66	49.19	-20.85	-17.40	-27.79	-12.29	4.25	1.90	-4.87
Burundi	-12.87	-38.24	-13.70	-20.61	-55.79	-39.41	-32.75	-15.63	-11.32	-26.47
Comoros	6.39	-10.07	13.44	-3.42	-15.49	-37.20	-21.12	-4.17	-2.52	-16.12
Djibouti	39.20	5.78	25.58	8.32	8.14	-26.75	-8.31	2.98	6.09	-13.15
Eritrea	25.05	-4.70	36.59	8.53	-17.89	-26.66	-5.18	-2.53	15.03	-0.87
Ethiopia	-11.86	-2.40	-22.69	-35.52	-56.68	-50.55	-26.42	-2.93	-13.30	-20.33
Kenya	17.77	12.19	12.44	4.63	16.13	-4.74	18.64	23.16	10.51	15.40
Madagascar	3.26	-33.73	-3.84	2.45	-19.81	-34.34	-22.80	-12.28	-6.74	-20.04
Malawi	6.81	-9.82	11.68	-3.07	-5.42	-25.89	-5.09	15.61	-0.10	7.66
Mauritius	43.48	-40.13	44.45	-8.47	-25.67	-29.03	41.00	58.45	-20.95	5.90
Mozambique	22.63	-28.40	19.22	12.76	-9.94	-33.28	4.51	6.77	-11.46	-7.79
Rwanda	-5.56	-34.00	-7.81	-23.16	-50.44	-50.74	-32.07	-4.52	-8.96	-21.56
Seychelles	95.83	-11.65	-5.77	-34.96	-19.11	-31.12	53.24	81.93	9.63	-47.31
Somalia	11.32	-1.46	23.17	15.15	-8.98	0.92	-5.73	-4.67	24.16	2.17
Tanzania	22.29	-16.53	13.32	0.10	-6.40	-22.35	-7.44	0.77	11.26	-8.49
Uganda	39.13	8.47	52.98	33.43	-10.99	-12.05	3.92	16.46	9.49	12.50
Zambia	-3.51	-14.29	-10.87	-13.83	4.44	-31.42	-25.46	-10.54	-10.26	-25.71
Botswana	34.03	-3.18	21.51	-0.88	-18.48	-45.56	4.82	12.61	-12.68	-10.71
Lesotho	64.44	26.79	59.51	27.40	28.81	11.57	30.87	8.74	18.72	19.61
Namibia	15.18	-11.58	2.59	-8.57	-26.72	-42.49	2.17	-0.68	-15.69	13.76
South Africa	22.21	-7.98	17.62	-6.15	8.17	-24.37	3.15	11.27	-10.44	-13.03

Swaziland	32.87	-2.56	37.80	-1.13	-9.04	-9.93	2.69	-4.16	0.73	4.27
Zimbabwe	17.03	8.55	50.79	33.52	88.42	50.00	59.22	42.72	20.60	38.81
Benin	58.78	-31.50	45.32	-9.18	-10.32	-29.83	-1.78	42.04	0.50	-9.81
Burkina Faso	36.83	-43.73	4.80	-23.36	-36.99	-32.63	-7.83	30.11	-4.85	-15.04
Cameroon	36.41	-16.96	62.81	-16.79	-19.52	-23.00	-7.87	22.94	4.00	-7.87
Cape Verde	76.07	38.44	30.35	9.51	10.85	-23.14	34.61	68.65	33.25	23.52
Chad	51.03	-0.44	58.78	-7.92	-26.72	-7.56	9.98	27.81	15.24	2.45
Cote d'Ivoire	40.42	13.81	30.23	0.04	-12.59	-18.10	10.11	15.78	7.82	9.38
The Gambia	41.35	21.96	60.76	6.76	-6.05	-21.11	17.70	17.52	11.98	13.34
Ghana	6.06	-30.39	26.00	2.71	-44.81	-20.06	-20.34	52.40	-21.46	-17.45
Guinea	32.26	21.30	28.16	5.35	-4.09	-19.19	1.15	16.91	1.37	-0.14
Guinea-Bissau	42.79	-26.28	39.30	-17.23	-25.89	-30.89	-8.48	33.59	1.07	-8.72
Liberia	24.39	-36.42	23.67	-17.64	-11.99	-33.05	-18.67	26.89	-3.85	-16.59
Mali	24.67	-5.02	20.32	-19.65	-52.72	-47.10	-5.76	29.25	-7.06	-4.66
Mauritania	39.73	-19.85	43.67	-19.92	-48.38	-39.00	-5.88	30.47	3.13	-6.37
Niger	11.89	-34.69	13.06	-21.16	-40.53	-38.10	-20.54	24.31	-6.94	-17.75
Nigeria	16.70	1.04	54.95	-10.96	-38.97	-33.02	-0.42	4.16	1.41	5.75
Sao Tome and Principe	50.16	44.90	54.80	15.54	19.22	-15.39	19.42	69.04	10.97	17.20
Senegal	39.51	-14.48	53.49	-8.50	-5.28	-18.51	0.07	36.25	12.94	-1.74
Sierra Leone	44.18	-30.68	51.11	-3.45	23.91	-24.40	-2.03	39.96	5.01	-7.79
Togo	34.70	-33.27	42.55	-17.28	-22.31	-26.90	-10.60	33.44	-2.03	-12.88
American Samoa	3.75	22.77	29.68	-9.08	35.36	-18.90	17.86	31.76	-15.51	9.13
Bermuda	-43.51	-23.30	235.38	-30.92	-57.47	-13.65	-41.44	-23.91	-41.67	-48.13
Greenland	-2.95	-18.37	-14.40	6.00	1.80	-62.14	-37.77	-13.13	-26.85	-16.28
Guam	5.61	27.05	53.36	52.04	54.30	-4.81	-6.09	13.57	-11.90	0.07
Northern Mariana	6.03	11.21	11.66	-14.95	-0.14	-20.87	9.14	0.70	-16.57	-3.23
Puerto Rico	-12.84	-12.44	150.39	-44.28	-15.39	-41.54	-23.00	1.04	-24.67	-47.63
Virgin Islands, U.S.	11.65	9.60	122.01	11.36	26.90	5.62	-0.73	20.64	-8.82	-15.00
South Sudan	14.50	-5.76	23.54	10.06	4.41	-4.24	-8.77	-2.31	14.40	-6.29
Sudan	40.67	-14.85	-2.30	-4.88	116.28	-38.42	-5.76	16.05	-4.21	3.02
