# Supplementary material for: Incidence and death in 29 cancer groups in 2017 and trend analysis from 1990 to 2017 from the Global Burden of Disease Study
Source: J Hematol Oncol. 2019 Sep 12;12:96. doi: 10.1186/s13045-019-0783-9 (PMC6740016; doi:10.1186/s13045-019-0783-9)
Supplement: Supplementary file 10 — The ASIR of 29 specified cancer groups in 2017 and 1990, and relative changes between 1990 and 2017 in 195 countries and territories. (PDF 1526 kb) [file 13045_2019_783_MOESM10_ESM.pdf]

Age-standardized Incidence in 1990

	Total	Esophageal cancer	Stomach cancer	Liver cancer	Larynx cancer	Tracheal, bronchus, and lung cancer	Breast cancer	Cervical cancer	Uterine cancer	Prostate cancer	Colon and rectum cancer	Lip and oral cavity cancer	Nasopharynx cancer	Other pharynx cancer	Gallbladder and biliary tract cancer	Pancreatic cancer	Malignant skin melanoma	Non-melanoma skin cancer	Ovarian cancer
China	191.00	19.38	34.06	27.16	1.52	27.41	9.36	4.98	2.87	3.66	12.18	1.36	4.42	0.38	1.40	2.91	0.42	7.09	1.42
North Korea	165.07	8.67	20.82	27.60	1.46	22.81	11.79	8.40	3.71	6.06	12.21	2.12	2.42	0.49	1.89	2.85	0.40	2.16	2.43
China Taiwan	186.99	5.49	19.08	22.38	2.10	22.64	10.92	13.35	2.19	7.26	19.85	5.69	5.87	1.60	2.64	3.34	0.47	4.57	2.03
Cambodia	160.71	4.50	19.41	9.33	2.70	23.36	11.17	18.17	3.23	5.14	12.42	3.71	3.06	1.07	2.30	2.62	0.34	5.74	2.91
Indonesia	127.84	0.92	10.56	6.65	1.69	15.46	13.40	10.48	2.73	4.29	7.85	2.24	2.26	0.80	1.59	2.37	0.27	16.10	2.82
Laos	164.07	4.66	18.49	11.68	2.86	25.03	11.11	16.42	2.94	4.45	11.91	3.84	3.37	1.20	2.22	2.70	0.37	5.80	2.87
Malaysia	153.67	2.86	10.99	5.11	2.12	18.29	16.69	12.48	2.98	6.03	20.52	5.14	6.71	1.07	1.48	2.17	0.45	6.52	2.71
Maldives	120.15	3.56	7.50	6.29	2.19	12.96	9.17	14.01	1.27	5.02	7.84	5.83	1.12	0.55	1.30	2.43	0.59	6.10	3.30
Myanmar	202.32	12.32	22.23	11.47	3.07	22.23	21.62	22.16	4.02	5.20	14.15	4.04	3.63	1.22	2.60	3.42	0.43	5.86	4.50
Philippines	117.86	1.39	6.57	12.32	1.29	15.73	12.09	4.37	3.92	4.96	6.52	4.95	2.44	0.76	1.11	2.47	0.33	3.57	3.51
Sri Lanka	102.48	4.49	7.95	1.80	1.03	7.15	8.68	3.88	1.60	5.75	5.98	7.47	1.70	2.63	4.13	1.86	0.26	3.86	1.99
Thailand	172.08	3.00	8.69	22.86	2.85	28.39	10.80	15.72	1.80	4.47	12.36	8.31	2.27	1.09	5.97	3.19	0.40	8.54	3.23
Timor-Leste	118.35	3.07	12.78	7.84	1.82	15.44	6.95	12.13	2.38	4.92	7.13	2.87	2.34	0.73	1.87	2.02	0.28	5.92	2.13
Vietnam	157.30	3.67	17.82	14.50	1.75	33.41	10.34	10.42	1.52	3.81	13.83	6.11	2.35	1.90	1.24	1.77	0.22	4.40	2.50
Fiji	145.99	1.85	6.30	7.09	1.09	7.91	23.73	25.06	5.32	10.41	9.30	4.53	0.70	0.96	1.21	2.71	0.50	10.57	1.23
Kiribati	185.70	3.63	14.81	11.65	1.05	15.48	14.61	55.98	5.11	5.92	9.48	8.69	0.87	1.27	1.42	2.10	0.43	9.78	1.15
Marshall Islands	191.55	3.20	18.24	12.92	2.14	24.31	15.74	20.84	6.95	8.89	14.14	3.72	2.82	1.40	1.44	3.51	0.86	19.58	2.60
Federated States of Micronesia	176.15	2.38	15.03	12.11	1.89	23.67	14.86	19.48	6.40	9.10	11.87	3.59	2.56	1.34	1.50	3.32	0.76	16.21	2.53
Papua New Guinea	170.14	2.31	18.91	9.49	2.12	24.70	14.45	27.85	5.47	6.90	9.27	3.59	3.23	1.44	1.48	2.29	0.77	7.20	2.16
Samoa	141.54	1.48	11.51	4.74	0.77	7.65	12.35	12.21	5.03	7.63	10.23	2.72	2.14	0.31	1.13	2.84	1.64	26.24	3.26
Solomon Islands	162.44	2.35	16.53	10.65	1.91	22.54	10.93	22.26	4.92	9.07	7.87	2.89	2.65	1.11	1.33	2.33	0.76	16.70	1.82
Tonga	235.20	2.24	17.20	23.39	1.27	27.81	27.74	23.66	4.99	17.57	7.85	2.66	1.50	0.86	0.99	3.67	0.68	35.07	2.12
Vanuatu	200.84	2.07	15.56	21.28	2.10	24.25	15.12	20.93	5.44	14.26	11.46	4.56	3.00	1.27	1.82	3.07	1.30	18.28	2.43
Armenia	237.16	2.25	19.99	8.87	4.48	28.72	25.43	10.98	4.02	3.61	14.73	2.00	0.39	0.67	0.80	6.90	0.69	55.85	3.96
Azerbaijan	195.19	8.16	23.05	7.09	2.82	18.88	12.40	5.18	2.82	3.99	9.89	1.38	0.26	0.39	1.36	3.52	0.72	57.76	2.31
Georgia	208.53	1.61	14.80	5.08	4.16	19.85	29.04	11.56	5.32	4.11	11.69	2.79	0.70	0.95	1.20	2.96	2.68	57.26	2.71
Kazakhstan	261.53	17.97	29.08	9.52	3.74	35.83	15.37	10.80	6.24	3.96	15.48	3.92	0.60	1.72	1.43	1.31	1.94	56.34	3.85
Kyrgyzstan	182.69	7.55	28.28	6.76	2.35	22.88	14.33	11.77	3.18	3.35	12.41	3.80	0.49	0.83	1.29	3.90	1.16	30.60	3.35
Mongolia	323.92	25.71	59.47	59.36	0.97	35.80	6.64	15.65	2.17	2.90	8.47	5.79	0.31	0.79	2.94	2.85	0.18	57.36	1.70
Tajikistan	169.72	9.97	23.25	5.52	1.24	12.57	7.41	5.03	1.55	3.49	7.51	1.25	0.66	0.65	0.65	3.15	0.47	55.73	2.14
Turkmenistan	196.43	31.09	19.14	3.78	2.47	13.39	9.31	7.18	2.29	3.36	7.45	3.18	0.48	1.35	2.45	1.39	1.07	58.95	2.91
Uzbekistan	169.10	14.89	17.75	4.51	1.93	14.07	9.94	6.38	2.24	3.16	7.41	2.11	0.59	0.77	0.55	1.83	0.51	57.22	1.25
Albania	186.31	1.94	13.65	9.63	3.44	24.00	8.58	4.20	2.87	7.91	8.13	2.58	0.74	1.13	1.03	2.91	1.31	58.81	1.55
Bosnia and Herzegovina	211.06	2.48	12.21	5.21	4.36	33.21	12.70	6.65	2.76	5.88	16.18	2.54	0.14	1.48	5.51	6.61	2.06	56.02	3.40
Bulgaria	260.02	2.20	16.85	6.10	3.68	24.03	24.72	9.63	9.76	7.30	24.07	3.15	0.31	1.24	1.58	5.89	2.48	75.50	4.31
Croatia	329.98	3.82	22.73	3.10	6.25	39.86	32.04	12.70	9.91	14.05	34.40	6.89	0.86	3.91	4.61	8.33	5.21	52.95	7.96
Czech Republic	363.20	2.99	17.78	5.45	3.75	45.78	34.32	11.71	11.33	14.72	48.64	4.60	0.54	1.87	8.55	11.24	8.64	52.55	7.97
Hungary	329.94	4.11	18.92	6.35	5.94	45.73	34.56	12.44	9.46	12.50	40.76	7.95	0.78	3.89	6.89	9.40	5.43	33.56	6.80
Macedonia	224.17	1.30	18.46	6.97	3.81	22.89	21.69	7.31	4.85	7.10	15.88	2.17	0.42	0.82	2.21	5.33	5.11	58.25	3.65
Montenegro	257.17	2.37	7.36	5.28	6.42	40.70	26.81	8.10	4.77	11.40	19.65	3.29	0.20	1.12	2.35	6.66	3.87	58.07	3.98
Poland	231.86	3.62	16.68	3.51	5.07	40.02	22.22	10.04	6.02	7.22	24.10	3.50	0.35	1.67	4.59	8.95	2.86	15.64	6.53
Romania	188.49	1.57	13.81	3.72	3.68	23.53	19.04	17.09	5.19	6.55	16.54	3.17	0.47	1.62	2.50	5.84	2.10	17.91	4.86
Serbia	265.23	2.56	12.39	6.02	5.62	39.25	30.21	16.05	6.37	11.55	30.10	4.59	0.59	2.38	3.61	7.25	5.01	22.81	5.19
Slovakia	307.94	4.02	21.18	4.61	5.73	44.21	24.21	10.46	9.37	11.93	37.47	9.48	0.87	3.29	6.84	9.46	5.41	37.34	6.34
Slovenia	282.52	3.90	20.58	3.53	4.23	34.35	34.09	9.21	8.74	13.44	33.56	6.38	0.52	4.11	5.33	8.20	8.25	26.41	6.71
Belarus	219.92	2.62	31.99	2.42	4.92	29.00	21.52	8.30	5.23	7.11	21.91	6.33	0.40	1.29	1.43	5.32	2.54	21.70	5.82
Estonia	263.50	2.96	27.42	2.96	3.75	36.11	27.47	12.25	8.46	11.70	27.91	4.42	0.75	1.81	2.12	8.28	4.44	25.04	8.22
Latvia	231.69	2.72	25.40	2.66	4.13	33.16	24.57	7.48	8.70	8.83	24.16	4.07	0.72	1.46	1.58	8.45	3.68	18.76	7.53

Lithuania	251.66	2.75	25.49	2.21	4.75	32.26	25.90	9.70	7.95	13.50	22.87	4.31	0.66	1.48	2.24	7.82	3.45	27.39	8.22
Moldova	221.37	2.25	18.10	2.94	3.82	25.51	20.94	11.13	4.60	5.06	21.10	4.33	1.01	2.38	1.62	6.16	2.05	44.66	4.60
Russian Federation	260.32	4.57	30.01	4.38	4.32	33.08	22.41	8.44	8.65	7.73	23.11	4.25	0.61	1.86	1.65	7.23	3.69	38.75	6.05
Ukraine	316.72	3.10	28.36	2.09	5.06	35.74	24.61	10.16	5.08	5.43	25.97	4.39	0.66	1.82	1.42	4.87	3.21	102.23	4.92
Brunei	245.06	3.59	23.64	13.50	4.39	35.58	19.60	19.34	3.39	7.22	31.20	8.16	3.92	2.16	3.98	4.48	1.01	12.60	4.59
Japan	258.62	6.09	58.76	17.84	1.98	27.38	17.74	6.11	3.02	6.98	36.37	2.84	0.36	0.79	9.53	9.08	1.09	8.37	3.52
South Korea	197.80	5.04	49.24	28.67	3.78	20.08	9.88	8.38	3.57	3.43	14.30	1.92	0.54	0.46	9.29	6.52	0.43	4.46	1.34
Singapore	202.56	3.72	19.58	12.58	2.76	35.46	22.30	9.33	2.25	5.82	34.07	2.77	7.80	0.76	1.74	3.56	0.69	7.63	3.80
Australia	915.22	4.49	11.65	2.07	3.08	35.00	40.16	5.83	6.21	30.14	47.78	7.59	1.03	2.39	3.36	7.60	34.38	590.09	5.40
New Zealand	764.70	4.56	11.93	2.27	2.64	34.76	52.52	9.22	8.05	50.27	62.07	6.46	0.93	1.58	3.03	6.83	36.55	387.96	6.84
Andorra	359.60	3.47	11.24	2.23	2.54	33.45	36.49	4.71	5.98	27.63	36.13	5.33	0.81	1.67	3.15	7.63	9.96	79.74	5.77
Austria	313.74	2.23	17.26	4.53	3.18	28.26	43.59	9.86	9.70	23.64	40.78	3.88	0.83	2.08	5.41	8.88	8.33	27.35	8.15
Belgium	353.75	3.34	13.05	2.90	5.62	45.12	53.90	6.09	7.35	25.72	39.65	5.52	1.47	2.12	3.72	7.94	7.67	39.05	7.67
Cyprus	206.37	1.03	8.02	3.14	2.09	19.29	31.22	4.35	6.07	16.24	19.84	2.89	0.51	0.39	2.84	4.43	3.30	29.17	4.69
Denmark	364.76	4.22	10.43	3.38	3.83	45.23	45.99	9.88	8.20	17.01	28.41	5.02	0.94	2.67	3.41	8.92	11.26	56.03	8.53
Finland	358.79	3.13	16.78	3.61	1.88	32.29	37.37	3.82	7.98	20.71	24.68	3.56	0.65	1.07	4.45	10.94	8.00	96.49	7.62
France	347.71	6.99	11.19	6.19	8.27	30.19	39.52	6.17	7.45	22.16	34.32	10.82	2.04	6.34	3.74	6.91	6.57	61.86	6.29
Germany	325.72	3.44	19.29	3.32	3.24	35.54	42.31	8.53	10.70	20.29	45.40	5.69	1.07	2.78	6.88	8.86	9.65	19.09	8.94
Greece	300.66	1.57	12.03	11.89	4.26	33.59	31.06	5.36	5.37	15.03	17.26	2.58	1.08	0.55	2.12	6.34	3.58	81.52	3.89
Iceland	416.05	4.55	17.31	2.37	2.13	33.55	46.69	5.79	8.35	37.63	29.73	4.64	0.95	1.09	3.50	8.47	9.25	97.45	7.50
Ireland	372.75	6.31	13.31	2.50	2.77	35.61	46.85	6.50	6.24	21.85	39.65	5.43	0.95	1.23	2.92	8.76	6.54	99.06	7.72
Israel	287.19	1.52	10.30	2.70	1.42	19.89	37.80	4.10	3.89	11.70	26.73	1.82	1.34	0.59	3.31	7.96	7.24	90.85	5.96
Italy	338.11	2.76	20.93	9.29	6.10	35.25	44.89	3.13	3.97	18.46	34.18	5.40	1.31	2.05	4.40	7.92	7.64	42.58	5.55
Luxembourg	398.95	4.14	13.64	4.23	5.28	37.94	48.67	5.78	12.73	22.56	41.78	7.65	1.73	3.33	3.80	8.33	10.06	81.51	8.46
Malta	276.73	2.50	11.78	2.21	3.38	24.43	45.25	4.17	7.57	14.37	25.44	4.46	2.05	1.05	2.28	7.51	3.83	52.28	6.67
Netherlands	352.77	3.89	15.59	1.53	3.11	45.95	56.03	4.99	8.19	26.04	41.94	3.93	1.03	1.36	4.81	8.17	11.32	25.80	7.88
Norway	528.88	2.46	13.84	1.62	2.07	27.85	33.75	7.40	7.55	27.61	41.68	4.67	0.71	1.52	3.09	9.05	15.20	234.01	8.15
Portugal	241.40	4.16	25.75	3.94	3.81	18.01	34.67	9.05	7.56	17.99	29.51	5.53	1.38	1.44	2.82	6.26	2.42	13.69	3.94
Spain	316.01	3.54	19.29	6.79	7.78	30.97	33.21	5.41	7.76	14.46	30.80	8.81	1.80	2.25	4.61	6.42	5.03	55.03	5.24
Sweden	487.30	2.70	11.77	3.69	1.68	20.50	41.29	5.68	8.72	28.91	33.00	3.82	0.59	1.32	19.81	8.63	13.42	197.66	8.29
Switzerland	366.70	3.93	12.42	4.42	3.46	30.54	46.85	6.68	6.72	31.42	20.94	5.76	1.14	1.96	3.94	8.20	12.85	82.73	6.23
United Kingdom	446.10	6.18	15.54	1.86	2.64	46.02	58.57	9.22	5.53	24.92	42.71	3.97	1.06	1.70	2.88	7.93	12.59	107.92	9.19
Argentina	235.78	6.58	15.04	4.14	3.69	25.18	24.26	16.17	4.95	11.16	20.38	2.75	0.50	1.20	8.44	7.74	1.29	28.80	3.80
Chile	227.10	6.89	30.65	5.99	1.71	16.58	16.34	21.67	2.74	11.67	13.44	1.96	0.23	0.70	18.00	5.80	1.60	25.35	3.54
Uruguay	288.11	8.62	19.73	1.85	5.82	36.16	33.75	17.32	5.85	15.49	27.75	4.42	0.68	1.93	9.67	8.91	1.76	28.06	3.77
Canada	317.01	3.72	12.93	2.39	3.72	45.43	47.06	5.84	7.31	29.81	40.06	6.09	0.57	1.36	4.17	8.63	9.17	10.64	5.90
United States	1162.82	3.61	7.66	2.98	3.71	54.05	59.64	6.51	10.54	46.94	46.13	6.73	0.72	2.57	2.23	8.89	10.95	806.72	6.48
Antigua and Barbuda	196.24	3.12	13.78	6.26	2.26	8.13	24.31	14.19	4.23	43.86	13.95	3.13	0.39	1.77	3.61	1.44	1.74	16.27	1.11
The Bahamas	240.16	5.90	12.22	6.40	3.63	15.04	37.34	17.34	6.62	40.59	20.52	5.98	0.62	2.71	4.42	1.63	1.85	17.33	1.86
Barbados	237.38	5.24	14.50	5.63	2.01	8.64	35.11	19.89	8.13	40.89	21.96	3.84	0.58	2.14	4.12	1.28	1.07	16.57	1.11
Belize	136.84	1.78	11.24	7.13	1.59	9.59	8.34	19.66	4.14	17.24	7.73	2.00	0.35	0.72	2.95	1.55	0.48	17.60	0.63
Cuba	246.51	3.46	7.38	5.78	5.73	29.65	23.66	13.54	8.12	28.45	21.95	5.11	0.56	1.85	3.54	1.61	1.20	42.46	0.96
Dominica	218.86	3.88	20.60	6.42	2.34	11.65	23.71	21.32	4.01	41.37	12.02	4.46	0.46	2.26	4.39	1.43	0.78	16.36	0.72
Dominican Republic	116.41	1.52	6.84	4.31	1.84	9.23	8.50	10.45	3.62	13.70	7.39	3.12	0.44	1.11	1.89	1.38	0.35	18.13	0.73
Grenada	228.10	6.15	13.31	8.00	2.17	11.76	23.79	27.19	8.76	31.70	15.62	4.49	0.82	1.85	5.07	1.42	1.36	15.56	1.39
Guyana	144.39	1.77	10.08	5.40	1.28	6.36	12.73	21.06	4.06	20.68	10.64	2.29	0.29	0.76	3.20	1.34	0.59	16.28	1.12
Haiti	213.57	4.48	23.05	7.58	3.80	15.72	14.15	36.48	3.83	22.42	10.73	3.19	0.96	1.41	3.67	1.73	0.89	17.23	1.40
Jamaica	159.15	2.92	12.98	3.94	1.44	14.16	20.86	17.83	3.38	21.62	13.25	2.39	0.43	0.85	2.30	1.35	0.60	10.35	0.89
Saint Lucia	218.19	4.69	16.90	5.79	2.91	11.66	25.26	24.38	5.24	35.06	12.72	5.38	0.85	1.80	3.48	1.49	1.28	16.37	1.38
Saint Vincent and the Grenadines	205.95	2.04	13.09	5.51	2.66	8.36	25.17	30.76	6.71	29.29	12.95	5.75	0.62	1.84	3.21	1.45	1.36	16.12	1.19
Suriname	150.69	1.32	8.75	7.59	1.01	11.88	12.90	19.60	1.95	15.05	12.93	2.29	0.88	0.76	3.06	1.25	0.69	16.45	1.08
Trinidad and Tobago	183.34	2.30	10.09	6.44	1.87	10.74	25.73	16.11	5.69	33.30	18.63	3.04	0.42	1.21	3.65	1.29	0.63	4.03	0.86
Bolivia	235.46	3.00	42.04	8.61	2.14	12.10	10.56	29.18	4.62	12.43	10.32	2.18	0.43	1.62	7.00	3.18	1.30	46.06	1.88
Ecuador	159.83	2.18	26.74	7.27	1.20	7.50	7.75	14.83	5.60	11.66	7.73	1.64	0.24	1.29	5.00	1.60	1.13	29.52	0.88
Peru	145.41	1.59	19.39	5.80	1.42	12.09	9.16	14.58	3.64	10.08	8.64	2.15	0.26	1.54	4.31	1.98	0.77	16.96	1.18

Colombia	231.05	3.40	23.68	6.71	2.58	13.13	15.99	13.46	3.71	15.76	11.38	2.72	0.70	0.87	4.41	4.66	1.13	73.54	3.31
Costa Rica	233.29	2.60	38.53	5.37	2.40	10.66	19.97	15.19	3.78	21.26	15.06	2.80	0.85	1.09	4.60	3.17	1.63	47.76	1.70
El Salvador	163.60	1.25	13.17	3.95	0.99	6.47	8.17	13.99	4.55	8.30	6.08	2.42	0.73	1.29	3.34	1.60	0.32	61.72	1.09
Guatemala	166.12	1.34	20.97	7.97	1.32	7.24	6.34	10.25	3.12	7.38	5.18	1.66	0.46	0.86	4.78	1.30	0.23	66.14	0.58
Honduras	156.28	1.19	8.01	1.91	1.16	7.30	8.91	6.34	5.24	7.63	5.81	1.94	0.87	1.39	3.03	2.16	0.39	63.25	1.95
Mexico	309.03	1.65	11.10	6.72	2.02	12.50	12.65	18.92	2.25	13.14	7.66	1.73	0.42	0.48	4.09	4.63	0.70	178.37	2.76
Nicaragua	169.39	1.02	12.50	6.59	1.45	6.43	8.34	25.07	1.91	9.77	6.98	1.26	0.42	0.51	3.65	1.64	0.37	60.80	0.91
Panama	161.50	1.50	14.16	3.90	2.52	12.58	14.75	18.89	3.26	17.88	12.80	3.04	0.65	1.17	2.80	2.54	0.73	20.52	1.64
Venezuela	232.18	2.29	17.98	6.23	2.86	16.23	16.30	19.57	5.25	19.96	11.76	2.50	0.49	0.96	3.88	1.42	0.67	69.74	0.82
Brazil	260.36	5.74	15.93	4.79	3.12	15.06	15.79	13.15	2.69	13.30	10.62	3.86	0.34	1.96	3.37	4.64	1.30	111.68	2.85
Paraguay	122.12	3.18	10.49	3.38	1.51	10.47	11.89	20.64	4.11	9.77	7.24	2.50	0.25	0.93	2.56	2.27	0.65	5.06	1.34
Algeria	94.23	1.07	7.13	1.62	2.27	9.85	9.36	6.88	0.76	4.92	6.86	1.37	2.23	0.82	4.47	1.73	0.32	7.17	1.19
Bahrain	157.62	3.56	10.29	3.78	2.64	35.55	16.23	3.03	2.13	9.79	11.58	2.72	0.76	0.65	1.23	5.13	0.29	7.42	3.46
Egypt	90.57	1.52	4.60	13.49	0.97	5.74	7.12	1.79	1.05	3.96	4.93	0.88	0.23	0.30	1.16	1.73	0.19	6.50	2.03
Iran	143.46	4.91	20.50	2.96	3.02	9.65	8.59	2.89	1.04	7.02	8.57	1.04	0.37	0.25	1.04	2.09	0.91	40.17	1.36
Iraq	128.36	1.47	7.15	4.47	3.68	16.57	17.68	3.04	1.68	4.47	8.06	1.78	0.79	0.63	1.08	3.41	0.25	11.63	1.83
Jordan	122.47	1.21	7.27	2.34	2.23	12.21	16.09	3.37	2.37	5.00	12.58	1.94	1.09	0.47	1.72	2.46	0.38	12.18	1.90
Kuwait	107.71	1.48	4.28	3.53	1.61	10.09	14.91	2.52	2.16	8.24	8.20	1.96	0.66	0.61	1.75	2.98	0.42	5.34	2.39
Lebanon	213.89	1.37	11.61	3.47	5.17	25.44	28.90	5.79	3.53	10.05	17.26	2.29	1.00	0.56	3.07	3.52	0.94	20.41	4.68
Libya	143.74	1.32	6.88	5.23	3.90	20.93	9.43	4.76	1.89	6.92	14.24	1.87	2.60	0.42	3.15	4.60	0.35	9.68	2.14
Morocco	101.99	1.31	5.18	1.60	3.01	12.95	14.15	8.81	1.29	4.17	6.20	1.55	2.37	0.53	1.17	1.80	0.43	8.99	2.12
Palestine	138.43	1.26	8.92	6.09	1.39	16.48	18.16	3.32	3.81	7.72	16.09	1.30	0.57	0.28	0.94	3.64	0.26	12.86	2.28
Oman	99.47	2.62	13.22	4.73	1.40	7.74	6.90	4.24	0.85	6.48	7.46	2.44	0.66	0.51	1.31	1.95	0.33	7.04	1.34
Qatar	167.08	4.78	10.15	13.17	1.61	19.09	16.52	2.16	1.22	13.31	17.06	2.14	0.31	0.48	3.17	6.06	0.54	13.05	1.72
Saudi Arabia	90.99	2.08	6.99	6.36	1.29	7.57	5.62	1.47	0.82	9.92	6.67	1.84	1.47	0.44	1.55	1.85	0.22	10.19	0.88
Syria	97.12	0.97	5.96	4.01	1.11	9.09	7.95	2.37	1.00	6.44	6.93	1.02	0.24	0.28	0.80	1.93	0.24	13.10	1.05
Tunisia	116.90	0.85	6.88	1.66	3.35	17.35	11.13	3.80	1.23	5.47	8.76	2.06	2.26	0.58	2.74	1.91	0.45	14.34	1.62
Turkey	186.99	2.23	20.49	4.20	3.82	38.48	10.22	4.19	2.92	7.03	13.99	2.07	1.30	0.48	1.95	5.76	1.62	16.35	3.09
United Arab Emirates	166.52	6.14	9.31	4.48	3.26	13.66	10.07	5.01	0.81	6.52	13.27	2.14	0.68	0.81	1.66	3.93	0.60	13.58	1.43
Yemen	106.85	4.79	21.25	3.12	2.58	10.25	5.80	4.63	0.61	3.49	6.90	1.23	0.70	0.36	1.66	1.29	0.41	12.76	0.87
Afghanistan	169.70	9.58	36.36	6.50	4.10	13.06	10.74	9.61	0.98	5.66	10.63	1.74	1.26	0.52	2.25	2.15	0.62	13.71	1.48
Bangladesh	119.46	4.19	12.37	2.43	5.24	21.60	5.47	13.41	1.14	4.05	9.63	4.98	1.61	3.95	2.48	1.52	0.23	1.89	1.17
Bhutan	110.85	7.50	10.02	3.26	4.34	7.99	8.87	13.72	1.36	3.42	6.63	8.97	1.42	3.88	2.60	1.80	0.23	1.77	1.47
India	99.43	4.14	10.74	2.06	4.44	6.93	6.98	9.83	1.18	3.10	5.78	8.11	1.26	5.02	2.07	1.49	0.19	6.72	1.48
Nepal	106.96	6.16	11.24	2.15	4.68	11.01	8.23	15.07	1.21	3.03	5.80	7.84	1.35	3.43	2.60	1.45	0.21	1.81	1.21
Pakistan	142.78	7.13	5.53	4.50	5.55	11.96	14.88	4.95	3.55	3.70	6.37	20.17	1.43	3.36	4.02	1.44	0.50	4.70	4.97
Angola	182.40	12.43	13.35	13.38	2.40	14.07	9.23	31.21	1.66	9.11	9.70	2.82	0.92	0.66	1.58	3.06	0.67	27.77	1.80
Central African Republic	189.21	13.65	14.81	12.40	2.64	12.34	12.72	36.70	1.85	7.86	9.78	3.39	1.01	0.68	1.60	3.02	0.69	27.57	2.07
Congo	200.42	14.25	13.04	11.55	2.58	14.52	15.25	35.40	2.34	8.20	12.19	3.35	1.04	0.84	1.76	4.04	0.74	27.74	2.84
Democratic Republic of the Congo	154.82	9.29	9.80	10.94	1.77	9.75	9.31	25.79	1.56	9.06	8.03	2.52	0.70	0.48	1.30	2.63	0.61	27.50	1.89
Equatorial Guinea	186.10	15.10	14.74	11.91	2.52	11.26	10.94	36.30	1.89	7.70	9.95	2.88	0.98	0.63	1.55	3.06	0.67	27.22	1.95
Gabon	173.42	11.30	9.56	9.65	2.33	13.77	13.26	22.73	1.88	8.00	12.00	3.73	0.83	0.94	1.72	4.23	0.70	27.55	2.47
Burundi	189.96	18.66	13.07	8.88	3.06	9.21	13.17	36.72	2.89	9.76	8.68	3.92	2.08	1.53	1.33	2.86	0.97	12.45	3.55
Comoros	185.63	15.26	10.61	8.34	2.23	10.44	12.46	34.23	2.87	11.87	11.78	3.14	1.82	1.06	1.26	3.18	1.04	13.03	3.92
Djibouti	174.85	12.83	9.15	8.07	2.14	10.05	11.64	28.62	2.90	11.88	13.43	3.14	1.67	1.13	1.27	3.27	0.97	13.02	3.84
Eritrea	215.27	18.34	15.86	10.23	3.17	10.24	13.85	43.12	3.40	11.29	13.04	3.76	2.49	1.36	1.56	3.11	1.31	12.68	4.01
Ethiopia	197.11	9.81	14.20	5.77	1.61	12.28	12.79	30.31	1.74	4.08	14.07	3.80	1.79	0.60	2.20	2.87	0.44	33.44	4.04
Kenya	124.30	8.77	9.91	4.27	1.77	6.28	9.04	14.31	1.02	7.08	8.19	2.91	1.64	0.92	1.65	2.21	0.50	21.00	2.01
Madagascar	157.31	11.37	9.25	6.45	1.90	7.93	10.67	28.92	2.25	9.67	10.07	3.01	1.61	0.94	0.98	2.29	0.96	12.89	3.13
Malawi	149.03	21.69	5.16	5.26	0.90	5.84	7.28	25.67	1.62	6.06	4.95	2.65	0.82	0.23	0.96	2.13	1.85	7.74	1.83
Mauritius	125.67	2.98	12.84	3.28	2.83	12.55	12.41	10.29	6.64	6.29	9.91	4.63	1.31	0.67	1.56	3.84	0.23	5.94	3.01
Mozambique	167.12	7.27	8.17	23.79	2.30	8.56	11.45	33.65	2.39	4.71	12.12	3.09	0.16	0.64	1.27	2.03	1.06	12.92	3.16
Rwanda	189.58	15.74	12.99	9.62	2.97	9.38	12.47	37.24	2.68	11.87	8.38	3.62	2.04	1.44	1.34	2.98	1.00	12.65	3.61
Seychelles	184.09	5.99	10.98	1.82	7.42	14.92	15.82	14.30	4.30	18.95	15.02	10.28	3.23	3.41	1.62	5.68	0.77	6.19	5.56
Somalia	175.30	16.23	11.84	8.47	2.24	8.49	9.77	33.80	2.56	11.60	11.21	2.69	1.68	0.90	1.20	2.58	0.87	13.08	2.97

Tanzania	150.87	5.52	8.67	7.86	1.95	7.08	10.09	26.78	2.42	11.99	10.22	1.83	1.43	1.01	1.17	2.64	0.92	12.99	3.32
Uganda	158.30	12.44	7.60	8.60	1.57	5.31	12.03	25.32	2.59	17.58	7.75	3.02	1.72	1.27	0.81	1.87	0.96	8.22	3.43
Zambia	207.28	7.79	11.97	12.08	2.71	9.36	14.50	39.84	3.38	16.59	13.10	2.96	2.14	1.40	1.42	3.81	1.20	13.72	4.52
Botswana	181.09	11.61	7.68	7.02	2.31	14.84	11.06	19.42	1.85	14.33	9.07	4.56	0.74	0.73	1.21	3.82	1.52	43.79	2.45
Lesotho	181.28	13.15	10.32	8.93	2.75	12.44	10.06	23.29	1.94	11.33	7.42	4.33	0.83	0.67	1.18	3.29	1.40	43.35	2.21
Namibia	139.51	3.57	5.53	2.85	3.64	8.37	14.04	17.86	2.26	9.12	7.84	7.09	0.92	1.50	1.06	2.48	3.10	17.07	2.48
South Africa	229.80	12.35	6.37	9.11	2.20	17.51	14.21	24.42	1.64	12.11	9.33	4.48	0.70	0.60	1.23	4.40	1.20	82.04	2.79
Swaziland	208.83	17.62	9.74	8.52	2.95	16.33	11.45	26.64	2.35	13.39	10.67	5.34	0.92	0.90	1.39	4.87	1.62	44.02	3.10
Zimbabwe	190.45	12.83	10.38	15.99	2.36	11.57	9.15	20.53	2.25	17.87	9.66	2.15	0.66	0.38	1.39	4.03	1.31	33.03	2.54
Benin	136.56	3.59	14.45	18.65	1.33	9.59	8.69	18.87	1.58	9.48	6.59	1.91	0.58	0.45	1.60	2.42	0.42	11.92	1.46
Burkina Faso	191.53	6.16	15.68	49.15	1.41	7.89	14.61	26.44	1.81	8.87	12.32	1.87	0.73	0.53	1.80	2.16	0.47	12.00	1.63
Cameroon	154.44	4.43	14.62	21.77	1.54	10.38	10.92	20.97	1.97	9.74	8.49	2.40	0.70	0.63	1.75	3.31	0.48	12.06	1.85
Cape Verde	132.40	7.74	24.41	12.86	1.39	8.16	8.98	17.03	2.14	4.88	3.97	3.52	0.37	0.86	0.78	3.56	0.21	11.59	1.28
Chad	136.23	2.86	13.06	28.24	1.13	8.32	8.01	20.31	1.49	7.88	6.47	1.86	0.55	0.41	1.51	1.75	0.39	11.89	1.23
Cote d'Ivoire	109.03	1.05	6.74	9.48	1.56	7.39	10.72	9.01	1.19	21.44	5.70	2.11	0.95	0.74	1.59	1.90	0.43	5.13	1.90
The Gambia	115.52	1.86	7.24	36.98	0.83	7.57	5.80	13.04	1.16	4.66	5.89	1.72	0.64	0.41	1.47	1.84	0.29	4.71	1.14
Ghana	147.85	3.38	11.07	16.71	1.29	6.19	10.91	17.74	3.03	17.01	7.93	2.19	0.41	0.81	1.27	5.02	0.23	11.89	1.72
Guinea	164.47	1.74	14.25	36.06	1.18	8.36	9.25	33.46	2.03	8.80	6.01	4.71	0.92	1.33	1.65	1.95	1.03	6.65	1.81
Guinea-Bissau	183.48	5.30	21.74	30.00	2.03	12.83	10.79	28.47	1.93	9.30	11.36	2.47	0.91	0.66	1.90	2.92	0.49	11.92	1.69
Liberia	137.36	3.79	12.77	21.16	1.33	8.29	8.46	18.32	1.39	9.66	7.79	1.94	0.60	0.46	1.43	2.35	0.41	11.91	1.30
Mali	163.84	2.55	22.93	40.51	1.21	7.00	9.71	20.35	1.20	5.30	7.55	1.77	0.97	0.30	1.26	2.29	0.82	7.02	0.98
Mauritania	150.78	4.25	14.73	19.56	1.28	9.95	11.74	22.29	2.31	8.57	9.13	2.19	0.69	0.52	1.67	3.03	0.53	11.88	1.97
Niger	154.89	3.14	14.63	42.18	1.22	8.11	6.90	20.80	1.43	8.63	6.69	1.75	0.59	0.39	1.55	1.81	0.41	11.87	1.16
Nigeria	133.88	2.53	5.57	3.80	1.61	7.97	18.60	16.43	0.81	21.93	8.50	1.05	0.85	0.23	1.28	3.02	0.59	14.39	1.66
Sao Tome and Principe	126.17	2.27	14.45	1.63	0.80	11.53	7.74	22.26	2.45	5.53	8.44	0.85	0.20	0.42	1.91	1.24	0.19	11.60	2.00
Senegal	145.98	3.50	14.50	19.03	1.38	11.41	9.08	17.29	1.55	10.18	9.25	1.96	0.56	0.45	1.55	2.57	0.46	16.60	1.42
Sierra Leone	158.07	3.05	13.46	44.40	1.34	9.96	8.54	17.00	1.36	8.31	8.15	1.85	0.55	0.47	1.45	2.07	0.38	11.83	1.29
Togo	134.03	3.10	13.42	17.37	1.28	8.86	9.91	20.22	1.63	8.63	6.53	2.00	0.62	0.47	1.50	2.25	0.43	11.90	1.62
American Samoa	196.60	1.16	15.62	7.77	1.78	25.63	17.20	7.71	8.81	27.51	15.85	2.69	1.97	1.84	1.39	3.72	1.09	24.09	5.95
Bermuda	282.49	6.27	11.01	5.21	3.84	29.52	40.57	10.07	6.83	33.02	32.28	6.22	0.59	2.60	3.62	1.75	4.43	21.56	5.92
Greenland	567.98	13.24	19.98	4.98	2.45	105.92	24.45	19.35	1.25	6.40	45.44	8.99	10.71	4.58	3.07	18.36	2.20	221.91	8.16
Guam	191.60	1.90	5.85	7.14	2.25	34.20	17.26	7.08	8.27	17.01	18.84	4.25	4.23	1.45	1.19	3.77	0.94	22.97	2.22
Northern Mariana Islands	212.52	1.43	10.07	6.50	3.28	36.76	19.37	14.08	8.71	15.18	16.55	9.68	2.57	2.64	1.04	4.22	1.17	29.31	2.11
Puerto Rico	202.68	5.86	10.54	6.44	3.90	14.38	25.69	7.32	4.78	29.10	19.54	5.80	0.61	2.20	1.34	1.87	1.44	22.07	1.21
Virgin Islands, U.S.	242.49	3.38	10.77	4.03	2.84	17.26	30.25	12.85	4.99	51.34	27.69	3.61	0.88	1.78	2.97	3.37	2.51	19.46	3.82
South Sudan	168.89	17.06	10.62	9.99	2.20	9.90	9.52	26.00	2.41	12.51	10.52	2.76	1.64	1.09	1.26	2.91	0.86	13.28	2.75
Sudan	105.87	4.85	17.61	3.63	2.39	8.25	6.28	3.98	0.60	4.69	6.60	1.36	0.67	0.34	1.50	1.41	0.42	13.28	0.93

Age-standardized Incidence in 2017

	Total	Esophageal cancer	Stomach cancer	Liver cancer	Larynx cancer	Tracheal, bronchus, and lung cancer	Breast cancer	Cervical cancer	Uterine cancer	Prostate cancer	Colon and rectum cancer	Lip and oral cavity cancer	Nasopharynx cancer	Other pharynx cancer	Gallbladder and biliary tract cancer	Pancreatic cancer	Malignant skin melanoma	Non-melanoma skin cancer	Ovarian cancer
China	244.21	12.23	28.97	26.04	1.98	42.05	18.22	5.44	3.26	7.76	22.42	2.44	2.38	0.51	1.73	4.37	0.88	13.37	2.07
North Korea	173.37	8.14	20.35	26.13	1.56	22.94	15.58	9.72	3.49	6.67	14.26	2.19	2.61	0.42	1.75	2.99	0.41	2.46	2.79
China Taiwan	315.82	8.92	15.84	33.31	2.56	30.46	31.60	5.50	7.38	16.71	48.02	17.51	4.32	5.57	3.33	5.85	0.86	9.30	4.46
Cambodia	144.11	2.55	9.60	8.06	2.06	19.48	15.91	12.46	3.03	7.11	13.12	3.85	2.58	1.05	1.82	3.07	0.35	5.91	3.87
Indonesia	138.80	0.80	7.45	7.80	1.70	17.45	15.53	8.72	2.85	7.39	9.26	2.41	2.24	0.85	1.50	3.45	0.33	17.04	3.74
Laos	140.08	2.48	8.52	9.44	1.87	20.47	13.75	11.16	2.49	7.20	11.72	3.03	2.34	0.94	1.67	3.12	0.37	6.08	3.71
Malaysia	180.74	2.65	7.11	6.54	2.11	18.35	28.58	8.64	4.46	10.08	26.92	4.99	5.40	1.25	1.28	3.27	0.63	6.53	4.00
Maldives	101.27	1.45	2.43	4.57	1.45	6.81	12.00	4.17	1.23	7.73	9.07	7.55	0.53	0.40	0.94	2.51	0.64	6.01	3.43
Myanmar	176.77	8.95	10.02	9.79	2.01	17.76	25.00	14.77	3.93	8.43	14.92	3.82	2.73	1.05	2.00	4.13	0.45	6.00	5.66
Philippines	160.41	1.23	3.98	11.38	1.33	17.53	27.57	8.84	4.04	10.54	18.39	3.97	2.99	0.70	0.67	3.61	0.51	4.55	6.13
Sri Lanka	124.69	3.93	4.27	3.07	1.48	6.79	16.25	3.92	3.21	7.64	10.13	8.78	1.39	2.46	2.16	2.59	0.40	5.83	3.20
Thailand	166.22	2.93	4.80	22.34	2.32	19.06	18.45	8.80	2.36	8.07	15.97	6.40	2.27	1.10	5.25	3.33	0.55	5.39	3.78
Timor-Leste	132.37	2.09	8.14	8.35	1.78	17.43	11.36	10.21	2.63	9.83	10.20	3.24	2.43	0.78	1.75	2.88	0.38	6.34	3.25
Vietnam	182.83	3.39	8.98	15.11	2.36	38.45	14.82	9.17	2.18	5.84	21.02	7.58	2.71	3.02	1.23	3.03	0.32	7.15	3.34
Fiji	180.14	2.40	5.47	8.30	1.34	7.62	31.49	22.57	6.56	11.76	11.78	4.62	0.71	1.32	1.16	3.45	0.55	28.79	1.73
Kiribati	189.40	4.19	11.31	11.43	1.04	15.42	17.59	49.61	5.39	5.27	10.33	11.10	0.95	1.52	1.34	2.70	0.46	13.14	1.65
Marshall Islands	229.44	3.23	15.06	14.08	2.43	26.50	25.50	21.75	9.45	13.78	17.23	4.52	3.41	1.78	1.32	5.15	0.96	24.10	5.06
Federated States of Micronesia	191.59	2.09	11.36	11.05	1.77	23.37	23.04	15.95	7.71	10.30	13.69	4.00	2.50	1.41	1.17	4.24	0.74	24.09	4.51
Papua New Guinea	181.02	2.11	15.96	9.66	2.22	26.48	17.43	24.25	6.01	9.01	10.02	4.04	3.38	1.56	1.36	2.96	0.82	12.73	3.43
Samoa	151.60	1.49	10.10	4.61	0.68	7.33	13.23	11.24	5.83	8.34	11.21	2.27	1.78	0.31	1.07	3.10	1.56	36.08	3.96
Solomon Islands	163.49	2.15	12.51	10.10	1.75	20.13	15.44	19.09	5.57	9.27	8.96	3.16	2.59	1.23	1.16	2.94	0.70	19.27	3.21
Tonga	243.61	2.36	13.58	24.28	1.38	25.29	35.13	18.81	6.32	19.46	9.38	3.47	1.75	1.06	0.92	4.54	0.78	29.98	3.63
Vanuatu	226.17	2.07	13.08	16.09	2.20	23.71	21.00	19.20	6.76	18.97	12.89	5.42	3.48	1.50	1.86	4.25	1.48	29.75	4.39
Armenia	259.74	1.56	12.49	9.19	3.40	28.13	33.84	8.46	8.57	12.11	18.72	1.99	0.32	0.56	1.42	8.57	1.39	57.15	4.09
Azerbaijan	225.42	8.12	19.51	7.87	2.93	19.60	18.67	5.96	4.83	9.25	12.86	2.18	0.32	0.72	1.41	5.52	1.06	60.21	3.14
Georgia	278.25	2.56	14.20	7.30	4.42	25.42	35.25	10.14	12.90	10.29	15.69	4.43	0.50	1.71	2.10	5.28	3.41	66.95	5.26
Kazakhstan	219.45	6.42	13.04	5.55	1.97	18.32	20.69	9.17	6.74	6.91	16.44	3.61	0.53	1.27	1.07	5.43	2.36	57.72	4.76
Kyrgyzstan	169.19	3.37	13.99	6.97	0.92	10.87	12.37	9.20	4.15	3.93	8.51	2.24	0.41	0.89	0.92	3.87	0.98	59.29	3.32
Mongolia	302.88	18.46	35.62	93.44	1.37	24.58	9.49	10.29	2.96	3.53	8.25	2.87	0.31	0.60	2.16	4.49	0.21	56.20	2.77
Tajikistan	160.95	5.02	17.35	5.61	0.80	7.50	10.97	3.24	3.90	4.62	8.02	1.33	0.58	0.65	0.54	3.62	0.61	55.48	2.58
Turkmenistan	184.96	8.73	9.66	5.66	1.21	9.70	18.97	9.40	1.68	5.78	9.59	3.61	0.51	1.41	1.03	3.51	0.93	56.70	3.25
Uzbekistan	176.05	4.75	10.83	5.84	2.13	10.02	17.80	7.71	4.48	5.66	9.50	3.01	0.66	1.37	0.60	3.45	0.79	56.76	2.14
Albania	223.69	1.34	10.48	6.85	2.98	23.11	19.45	3.85	5.49	14.67	11.18	2.12	0.46	0.99	1.08	5.22	2.19	61.22	2.33
Bosnia and Herzegovina	273.12	2.05	9.33	7.29	3.27	35.32	26.62	6.52	7.22	12.80	29.42	2.50	0.26	1.62	3.18	8.39	4.54	59.08	5.77
Bulgaria	301.80	1.80	9.54	5.32	5.33	28.32	35.07	10.26	14.21	12.26	35.51	4.51	0.45	2.40	1.62	8.40	5.86	57.27	5.96
Croatia	365.06	2.51	12.51	6.12	4.28	38.46	35.67	6.64	14.54	23.21	45.95	4.42	0.42	3.52	3.73	9.15	10.37	52.63	6.51
Czech Republic	397.78	3.04	8.59	4.26	2.83	33.28	32.36	6.63	11.31	26.76	40.12	4.57	0.28	3.02	5.24	11.13	17.70	98.43	6.31
Hungary	343.30	3.50	8.41	4.39	5.40	45.39	34.31	6.37	8.25	15.01	44.75	7.85	0.68	6.18	3.29	9.83	8.94	63.98	5.25
Macedonia	274.03	1.28	11.26	6.58	4.63	29.51	29.53	6.39	10.62	14.93	24.07	2.15	0.31	1.03	1.67	7.09	8.64	60.53	4.69
Montenegro	292.40	2.38	6.17	5.32	7.10	43.26	36.64	6.68	6.96	16.89	23.80	3.36	0.16	1.23	2.02	7.80	5.95	59.14	4.42
Poland	302.98	2.92	8.66	3.12	4.21	37.12	31.21	5.52	11.38	17.00	29.70	4.30	0.35	2.75	3.21	8.22	6.67	54.81	6.46
Romania	294.47	2.39	9.54	8.22	5.09	29.68	28.51	13.83	8.19	15.54	30.51	5.50	1.00	4.71	1.57	8.01	5.40	52.74	5.22
Serbia	343.55	2.45	8.71	5.51	5.50	43.12	40.40	11.14	11.75	16.90	38.37	4.04	0.45	2.74	2.92	9.01	7.62	58.94	5.85
Slovakia	385.51	3.48	13.89	4.86	3.37	35.11	33.90	8.99	12.58	22.65	52.38	5.80	0.56	5.07	7.10	10.82	11.81	64.31	6.70
Slovenia	339.15	2.52	11.80	4.84	2.81	32.46	32.82	4.62	9.38	29.11	39.39	4.03	0.24	4.85	4.02	8.94	17.52	54.48	4.82
Belarus	263.92	3.25	18.57	2.73	4.21	22.55	23.91	7.74	5.88	22.26	28.28	5.03	0.54	3.33	1.46	6.13	7.31	32.83	4.76
Estonia	339.65	3.63	18.69	3.37	3.21	27.84	30.81	6.87	13.04	37.07	34.83	4.04	0.48	3.13	1.83	9.19	11.56	46.67	6.80

Latvia	316.90	4.17	17.21	3.47	4.36	30.02	30.28	4.74	16.59	24.96	29.77	4.83	0.43	3.25	1.31	9.12	9.53	45.31	6.97
Lithuania	319.92	4.08	17.39	3.42	5.44	29.71	30.61	6.25	14.21	38.16	29.25	4.68	0.49	4.15	1.93	8.59	8.45	33.58	6.71
Moldova	222.84	1.71	9.88	8.75	4.12	18.86	22.67	8.01	6.04	10.75	25.17	3.67	1.19	4.01	0.74	6.75	3.30	42.84	3.41
Russian Federation	301.51	3.59	17.83	3.96	3.34	26.35	32.41	8.29	8.95	15.78	29.94	4.32	0.43	2.52	1.47	7.69	6.95	53.81	5.36
Ukraine	357.69	3.20	17.82	3.61	4.41	27.21	27.17	6.03	9.64	13.28	31.54	7.09	1.08	3.67	1.59	7.47	7.00	103.62	6.48
Brunei	284.16	2.42	13.65	11.41	2.01	31.87	37.15	15.18	5.05	14.36	43.85	7.51	3.71	3.06	3.63	5.75	2.36	11.97	8.55
Japan	274.21	5.83	29.56	11.97	1.52	28.53	29.21	6.04	5.50	15.72	45.01	3.93	0.38	1.81	8.88	10.47	2.12	10.32	4.14
South Korea	245.83	3.25	29.15	23.46	2.06	30.73	21.64	4.43	2.26	12.36	32.50	2.24	0.41	1.08	8.55	7.86	1.84	5.56	2.55
Singapore	198.68	1.95	10.17	12.14	1.61	25.48	28.45	3.74	4.36	14.22	34.88	1.96	3.14	0.82	1.42	4.59	1.77	7.76	3.66
Australia	830.08	4.38	8.96	5.30	2.18	31.81	43.87	3.20	7.59	49.18	45.75	6.09	0.80	2.44	2.41	8.46	48.60	470.25	4.27
New Zealand	856.85	4.81	7.84	4.89	1.58	31.07	46.15	3.47	8.14	50.33	50.21	4.98	0.62	1.49	2.48	7.48	46.83	495.41	4.91
Andorra	403.71	3.51	9.16	3.43	2.03	30.52	46.47	4.00	8.74	45.47	38.32	4.43	0.61	1.82	2.18	7.75	16.89	80.72	5.71
Austria	310.44	2.75	10.20	6.85	2.19	32.58	38.89	4.24	8.89	37.80	31.54	3.90	0.76	4.06	3.33	10.72	14.04	18.26	5.00
Belgium	383.63	4.19	7.74	4.52	3.11	37.32	47.54	3.80	8.88	33.33	35.51	5.71	0.91	3.05	1.83	7.97	14.27	76.64	4.85
Cyprus	331.89	1.12	8.50	4.16	2.14	26.01	46.75	3.39	9.44	38.67	28.98	3.13	0.51	0.56	1.98	6.30	9.51	67.17	4.74
Denmark	467.03	4.81	6.81	4.45	2.61	44.04	47.34	4.91	9.04	38.50	45.59	5.03	0.63	4.51	2.72	9.77	24.52	102.84	6.41
Finland	401.82	2.93	7.50	5.68	1.40	25.46	43.87	2.51	9.02	46.37	28.94	3.84	0.37	1.54	2.92	10.75	16.77	98.81	5.69
France	391.01	3.64	7.17	7.21	4.08	33.70	43.52	4.12	9.32	29.20	33.03	5.69	0.98	4.59	1.78	8.32	13.61	93.36	4.67
Germany	407.40	4.76	11.81	6.09	2.87	37.99	49.04	4.60	9.59	41.11	41.11	5.44	0.62	4.04	4.31	10.80	16.96	66.44	5.45
Greece	363.32	1.25	10.37	6.15	4.19	36.08	42.13	4.59	10.41	25.53	27.63	2.99	1.20	0.81	2.49	8.01	9.12	82.72	5.17
Iceland	431.80	4.43	8.84	3.96	1.69	34.05	38.54	2.92	7.46	50.61	31.67	4.43	0.52	1.33	1.81	8.58	17.47	101.60	4.49
Ireland	440.67	6.11	9.78	4.17	2.64	31.80	48.70	5.05	9.54	39.68	40.63	4.02	0.66	1.94	2.05	8.20	18.58	115.64	6.49
Israel	304.79	1.26	6.63	3.16	1.61	19.41	39.09	3.56	7.22	16.19	27.91	2.21	0.55	0.48	1.31	8.69	11.16	85.80	4.54
Italy	369.72	1.83	12.70	9.12	3.64	29.54	44.17	3.29	12.22	29.38	37.18	3.81	1.03	1.87	3.75	9.08	14.00	56.10	4.82
Luxembourg	431.44	3.59	8.90	6.12	3.57	36.57	50.15	3.45	15.26	34.07	42.14	5.45	0.99	3.52	2.14	9.63	20.63	81.69	6.56
Malta	343.18	2.35	8.18	2.58	2.82	26.37	50.12	2.84	11.30	24.82	34.41	4.33	1.72	1.44	1.46	8.88	9.10	64.25	6.09
Netherlands	456.24	7.27	11.32	4.24	2.49	44.37	57.29	3.89	12.11	42.80	50.85	4.39	0.76	2.21	2.88	9.94	26.51	67.08	5.84
Norway	588.79	2.73	7.10	2.94	1.52	30.46	38.02	4.02	9.63	61.45	48.41	3.53	0.45	1.83	2.56	9.06	25.16	236.65	6.24
Portugal	319.45	3.11	15.70	4.99	2.80	19.44	36.70	5.49	9.63	30.47	41.40	5.42	1.11	4.03	2.00	6.16	7.24	54.12	3.61
Spain	345.57	2.83	12.30	7.35	4.66	32.92	33.09	3.89	10.48	27.73	43.35	5.70	0.93	2.84	3.19	7.34	9.88	48.41	4.49
Sweden	380.53	2.64	5.40	3.86	1.27	20.67	46.42	4.01	8.92	49.97	34.70	3.30	0.41	1.73	2.53	7.59	23.89	79.73	4.79
Switzerland	373.97	3.33	6.61	6.01	1.78	24.87	38.39	2.88	6.59	38.17	29.38	4.12	0.60	2.47	1.96	7.88	18.97	100.95	4.29
United Kingdom	511.33	7.19	10.33	4.79	2.48	39.77	53.88	5.33	9.62	37.97	41.72	5.17	0.70	2.93	2.17	8.60	22.80	156.08	6.96
Argentina	231.54	4.14	9.60	3.85	2.64	20.27	30.72	16.52	5.12	17.23	26.12	2.94	0.30	0.67	5.27	8.44	2.92	23.24	4.09
Chile	234.35	3.47	18.59	5.33	1.31	14.70	22.29	10.85	5.32	25.30	22.24	1.92	0.17	0.61	10.83	6.26	3.58	23.28	3.57
Uruguay	305.95	5.61	13.36	2.60	3.88	29.18	39.26	15.47	6.52	25.76	33.30	4.38	0.52	1.56	7.58	11.27	4.36	35.34	4.94
Canada	400.25	4.12	10.50	4.68	2.43	38.01	42.64	4.81	9.37	33.55	38.53	4.30	0.43	1.42	2.77	7.94	15.40	90.21	4.73
United States	1278.51	3.85	6.00	6.69	3.19	44.99	50.39	5.06	14.66	49.96	39.14	5.75	0.52	2.64	2.00	10.07	17.54	930.81	5.07
Antigua and Barbuda	239.91	2.61	9.23	5.22	2.27	7.56	35.71	10.68	8.24	66.02	19.93	2.69	0.46	1.28	1.23	4.34	2.53	18.50	4.82
The Bahamas	276.62	4.74	8.35	5.29	3.62	12.82	52.29	15.09	10.39	57.96	25.80	4.95	0.75	2.06	1.76	4.01	2.50	17.69	6.49
Barbados	284.57	3.87	8.92	4.15	2.19	7.80	47.30	15.12	13.66	65.84	31.77	3.31	0.75	1.64	1.33	5.47	1.61	17.32	5.39
Belize	166.36	2.19	8.36	8.28	2.07	11.70	12.45	17.33	5.67	28.08	11.41	2.42	0.53	0.82	1.27	5.06	0.87	18.59	2.15
Cuba	282.42	4.64	6.27	4.52	8.58	30.59	30.28	9.82	15.35	46.15	29.86	5.67	0.68	1.83	1.06	4.89	1.89	28.84	3.31
Dominica	279.36	4.05	15.54	5.95	3.09	13.16	31.08	17.98	6.37	78.36	17.41	4.83	0.75	2.28	1.59	5.77	1.27	17.67	2.85
Dominican Republic	178.56	1.86	7.13	7.44	1.98	12.32	22.06	11.90	3.88	37.12	13.88	3.70	1.02	1.77	0.93	4.46	0.62	19.02	2.23
Grenada	238.92	5.53	8.16	5.32	2.42	12.05	31.62	15.74	13.31	46.31	18.68	4.19	0.64	1.31	1.40	6.26	1.26	18.44	5.87
Guyana	164.50	1.64	6.55	5.02	1.32	7.12	19.87	18.72	7.19	26.55	13.18	2.27	0.39	0.66	1.25	4.01	0.85	17.14	5.33
Haiti	201.24	3.45	15.12	6.37	3.09	12.13	19.06	24.53	4.56	32.65	12.79	3.14	0.89	1.08	2.21	3.06	0.97	17.51	2.87
Jamaica	228.06	3.08	9.24	3.83	2.35	19.18	34.83	16.02	9.17	45.21	22.02	2.32	0.89	0.98	1.45	4.10	1.11	9.81	4.52
Saint Lucia	226.89	3.72	10.16	3.98	2.90	10.41	28.66	15.02	7.62	51.57	15.12	4.49	0.99	1.36	1.01	5.62	1.70	17.97	4.92
Saint Vincent and the Grenadines	241.92	2.33	9.37	5.43	3.55	9.35	29.45	21.96	9.19	54.13	16.54	5.89	0.81	1.76	1.14	4.70	1.55	19.25	4.35
Suriname	180.28	1.31	6.07	7.50	1.21	13.67	18.49	16.78	3.29	25.61	18.97	2.37	1.20	0.68	1.10	5.35	1.00	16.99	4.92
Trinidad and Tobago	187.04	1.39	4.52	3.26	1.48	9.55	29.13	11.23	9.05	41.19	20.93	2.31	0.51	0.85	1.13	4.66	0.76	6.67	5.24
Bolivia	227.11	2.22	24.90	8.52	1.40	11.10	15.85	17.75	5.36	21.33	13.21	2.27	0.32	1.17	4.88	5.24	1.66	47.91	3.56
Ecuador	202.12	1.31	17.28	7.34	0.94	8.24	15.28	12.47	6.45	21.31	13.43	1.96	0.25	0.92	2.93	4.12	1.72	45.35	3.51

Peru	182.60	1.06	14.12	6.10	0.73	8.94	14.24	11.61	4.45	19.18	15.00	1.89	0.21	0.76	3.13	4.02	1.34	41.05	3.10
Colombia	222.08	1.49	15.89	3.86	1.34	9.68	20.68	9.09	4.09	25.55	16.11	1.83	0.36	0.48	2.19	3.65	1.93	68.26	3.61
Costa Rica	286.48	1.72	30.85	6.16	1.90	9.13	30.49	8.83	7.25	45.54	28.43	2.37	0.94	1.09	2.16	5.88	3.20	50.99	3.35
El Salvador	222.35	1.42	16.70	6.44	1.16	8.69	19.69	16.86	8.02	20.89	15.18	2.38	0.73	1.00	2.91	4.25	0.59	62.57	3.66
Guatemala	199.05	1.56	19.37	15.10	0.89	6.10	10.85	14.93	4.18	17.43	9.29	1.57	0.59	0.64	1.73	3.17	0.59	66.13	2.09
Honduras	187.67	1.46	7.07	2.48	1.10	7.69	13.80	4.98	11.15	13.84	9.70	2.77	0.86	1.67	3.49	3.99	0.68	63.98	5.10
Mexico	322.34	1.22	10.06	6.09	1.31	8.10	20.95	9.49	4.05	24.21	14.48	1.72	0.32	0.42	2.17	4.38	1.53	171.05	4.13
Nicaragua	175.63	0.70	9.44	7.05	0.94	5.86	14.40	14.17	2.34	16.25	10.94	1.13	0.34	0.37	1.93	3.10	0.52	62.37	2.46
Panama	190.46	1.34	14.98	4.54	1.55	9.37	22.51	11.95	6.59	33.79	18.55	2.11	0.63	1.02	1.41	3.80	1.14	20.06	3.02
Venezuela	270.65	1.73	13.95	4.57	2.82	16.42	27.56	16.06	5.75	45.13	17.87	2.47	0.55	0.84	1.62	4.51	1.28	68.01	3.86
Brazil	312.87	4.68	9.48	4.98	3.06	13.63	22.56	9.66	3.59	22.36	16.30	3.99	0.40	2.19	2.22	5.24	2.27	147.81	3.33
Paraguay	155.56	3.45	8.10	3.19	1.78	13.92	20.89	18.36	4.04	19.66	13.88	2.52	0.25	1.19	1.92	4.98	1.11	5.16	3.08
Algeria	108.02	0.95	4.34	2.00	1.77	8.21	17.76	4.74	1.12	9.54	8.62	1.35	2.01	0.95	3.43	2.69	0.56	6.44	1.76
Bahrain	117.00	1.17	3.85	2.86	1.08	12.04	19.54	1.57	2.95	16.40	11.25	1.53	0.34	0.38	0.72	3.72	0.38	6.69	2.56
Egypt	118.34	1.77	3.51	19.93	1.05	7.14	13.79	1.55	1.78	8.21	7.33	1.10	0.22	0.40	1.06	2.65	0.31	4.39	3.03
Iran	182.21	4.01	14.61	3.61	2.94	10.61	20.01	2.16	2.26	22.33	14.00	1.46	0.29	0.33	1.08	3.85	1.33	34.27	2.69
Iraq	79.71	0.64	2.79	3.24	1.41	9.14	12.40	1.33	1.77	4.32	5.55	0.96	0.20	0.32	0.53	2.77	0.19	4.30	1.80
Jordan	136.73	0.99	4.16	2.52	0.95	11.86	23.77	1.57	3.09	15.52	15.95	2.11	0.67	0.50	1.33	3.74	0.53	8.96	2.15
Kuwait	108.96	1.00	2.27	3.97	0.95	6.95	14.88	1.09	3.01	19.79	10.90	1.12	0.31	0.39	0.76	3.03	0.48	5.02	1.49
Lebanon	351.52	1.21	7.73	3.22	4.75	23.84	74.09	3.38	7.55	32.62	28.08	2.97	0.67	0.72	2.39	4.77	3.22	21.49	6.62
Libya	205.06	1.65	6.43	5.88	4.45	20.43	25.18	4.93	3.75	16.71	21.76	2.45	2.44	0.52	2.70	6.42	0.75	9.82	3.90
Morocco	124.47	1.24	3.79	1.74	2.97	13.42	24.59	6.33	1.86	8.40	8.79	1.89	2.15	0.62	1.02	2.71	0.67	9.25	3.22
Palestine	142.16	0.93	5.16	5.42	0.90	14.11	24.38	2.04	4.20	11.77	16.97	1.21	0.44	0.26	0.81	4.43	0.29	13.35	3.01
Oman	119.57	2.26	7.05	4.37	1.14	6.47	13.30	2.08	1.32	16.26	10.92	2.30	0.41	0.56	0.94	3.09	0.61	6.93	1.60
Qatar	177.32	2.22	5.61	8.72	2.05	12.71	23.13	1.24	2.52	39.34	17.76	1.87	0.29	0.62	1.07	4.48	0.80	12.30	1.92
Saudi Arabia	155.50	1.96	4.82	6.42	1.37	9.06	18.33	1.31	2.36	31.56	16.63	2.53	1.31	0.66	1.55	3.87	0.58	3.92	1.67
Syria	117.40	0.96	4.10	3.61	1.06	9.19	14.90	1.59	1.44	16.18	9.66	1.18	0.21	0.34	0.63	3.06	0.40	13.56	1.64
Tunisia	146.87	0.85	5.00	1.72	3.38	17.20	22.66	2.82	2.13	11.85	12.31	2.40	1.83	0.73	2.27	2.76	0.86	15.07	2.43
Turkey	210.28	1.29	9.87	3.73	2.81	30.79	18.76	2.08	4.62	20.83	17.64	1.33	0.79	0.43	1.16	5.85	3.14	33.97	3.07
United Arab Emirates	226.55	8.50	8.45	5.72	4.37	15.68	17.39	3.04	1.17	19.92	19.95	2.51	0.67	0.86	1.31	6.57	1.07	14.46	1.76
Yemen	119.41	3.88	18.49	2.97	2.53	9.91	10.59	3.67	0.82	7.99	9.54	1.39	0.54	0.34	1.41	2.09	0.52	13.43	1.49
Afghanistan	170.37	8.07	32.80	5.74	3.78	11.83	14.32	8.61	1.21	6.74	12.86	1.69	0.92	0.49	2.15	2.63	0.64	13.75	2.24
Bangladesh	100.20	2.55	6.17	2.25	2.73	19.83	10.31	6.13	1.02	3.81	8.43	6.80	0.96	3.41	1.85	1.73	0.24	1.76	1.57
Bhutan	98.49	4.31	5.69	3.84	2.69	7.13	10.76	5.27	1.19	5.28	8.10	7.74	1.00	4.20	2.58	2.85	0.30	1.88	2.03
India	112.50	3.66	7.46	3.18	3.47	7.52	13.02	7.80	1.57	4.31	7.94	9.32	1.16	6.29	2.77	2.79	0.28	6.02	2.63
Nepal	101.84	4.91	7.63	3.22	3.28	10.39	11.54	7.93	1.17	4.15	6.96	7.02	0.97	3.95	2.88	2.57	0.25	1.84	2.02
Pakistan	186.25	7.85	5.22	5.78	5.50	13.33	27.66	4.74	5.22	5.25	9.37	27.03	1.47	4.52	4.56	2.47	0.73	1.95	9.52
Angola	158.53	8.37	6.74	8.10	1.71	10.82	13.29	21.45	1.75	10.72	10.28	2.94	0.62	0.65	1.35	3.91	0.70	27.86	2.47
Central African Republic	167.42	9.81	10.36	7.90	2.11	10.55	15.31	28.22	1.65	8.14	9.79	3.15	0.80	0.58	1.34	3.04	0.64	28.10	2.23
Congo	186.29	8.89	7.34	7.92	1.82	11.85	20.83	27.00	2.35	12.43	12.82	3.27	0.77	0.71	1.43	4.82	0.80	28.11	3.58
Democratic Republic of the Congo	142.45	6.56	7.12	7.26	1.44	8.13	12.42	22.46	1.63	9.14	8.37	2.50	0.57	0.45	1.17	2.59	0.63	27.45	2.13
Equatorial Guinea	167.21	9.83	4.50	9.35	1.37	10.39	19.18	17.69	2.48	11.95	12.15	3.27	0.51	0.79	1.29	5.53	0.83	27.64	3.29
Gabon	171.05	8.48	5.68	8.11	1.86	13.02	17.48	15.77	1.85	12.31	13.22	3.64	0.66	0.84	1.27	5.49	0.78	28.06	3.02
Burundi	135.12	8.90	7.40	5.64	1.77	6.91	10.80	19.90	1.79	10.91	8.44	3.11	1.36	0.98	0.98	2.46	0.76	12.83	2.63
Comoros	159.24	8.06	6.20	6.20	1.40	7.80	17.34	22.77	2.59	13.59	12.00	3.02	1.36	0.83	1.06	3.50	1.01	12.80	4.66
Djibouti	165.25	8.16	6.05	7.35	1.63	9.72	15.08	18.62	2.46	16.34	14.40	3.23	1.45	0.96	1.04	4.24	0.96	13.46	4.18
Eritrea	198.01	9.76	9.11	8.50	1.94	8.61	20.35	31.80	3.41	13.08	14.34	3.93	2.08	1.18	1.49	4.00	1.46	12.60	5.55
Ethiopia	150.86	4.89	5.48	4.07	0.90	7.90	13.02	13.90	1.28	6.30	11.67	3.48	1.16	0.47	1.69	2.85	0.44	34.36	4.09
Kenya	148.53	7.92	9.04	5.68	1.81	5.92	12.72	11.65	1.18	10.94	9.63	2.98	1.94	1.10	1.70	3.17	0.62	33.56	2.49
Madagascar	135.19	7.32	6.62	5.28	1.35	6.74	12.23	22.19	2.01	10.23	9.96	2.60	1.27	0.78	0.91	2.37	0.90	12.75	3.08
Malawi	150.25	23.00	3.25	5.34	0.76	5.29	9.11	20.47	1.60	6.97	6.06	2.76	0.62	0.25	0.82	2.55	2.07	8.83	1.95
Mauritius	160.03	2.20	6.79	3.65	1.91	10.98	33.21	6.36	6.07	13.09	19.37	5.52	1.11	0.92	1.18	4.33	0.40	5.93	5.10
Mozambique	169.24	7.47	6.66	28.62	1.98	9.46	15.11	25.52	2.46	4.47	14.45	3.32	0.16	0.76	1.10	2.80	0.97	12.72	3.54
Rwanda	137.58	6.61	5.51	6.05	1.37	6.75	13.68	18.55	2.08	12.72	8.21	2.99	1.12	0.89	1.04	3.02	0.93	12.49	3.71
Seychelles	274.95	5.11	5.68	5.94	7.85	14.56	29.87	20.56	4.85	51.33	36.40	13.17	2.68	5.62	1.35	5.93	1.14	6.32	8.25

Somalia	166.04	10.47	8.71	7.72	1.72	7.62	11.96	28.52	2.31	12.64	12.73	2.90	1.57	0.83	1.24	2.95	0.94	12.93	3.53
Tanzania	140.72	4.26	5.76	6.31	1.38	6.79	12.44	18.57	2.14	13.87	10.10	1.80	1.22	0.91	1.04	3.20	0.94	12.91	3.73
Uganda	163.91	13.33	5.13	8.56	1.30	6.15	14.21	16.24	2.57	22.92	9.46	3.65	1.92	1.62	0.59	3.13	1.20	7.10	3.41
Zambia	160.04	5.77	6.87	8.99	1.71	7.87	14.16	21.19	2.46	13.58	12.18	2.63	1.56	1.13	1.12	4.09	0.97	13.18	4.16
Botswana	186.00	7.55	4.51	6.86	1.49	11.34	18.87	17.36	2.84	19.47	10.51	3.78	0.57	0.68	0.97	5.06	1.70	44.84	3.56
Lesotho	217.02	13.87	9.56	10.20	2.74	15.60	17.91	27.15	2.98	13.66	10.67	4.95	0.99	0.96	1.32	5.38	1.62	44.15	3.95
Namibia	164.15	2.86	3.10	3.08	2.77	6.89	23.66	13.47	2.13	17.11	8.61	7.65	0.63	1.54	0.77	3.31	3.46	30.38	2.47
South Africa	262.09	9.46	4.35	5.10	1.71	15.66	17.51	18.03	2.39	16.62	11.07	3.63	0.50	0.67	1.09	5.04	1.50	121.35	3.26
Swaziland	218.85	14.87	7.27	13.17	2.40	14.74	17.65	22.95	2.81	15.18	13.32	4.95	0.91	1.00	1.21	6.38	1.72	44.45	4.06
Zimbabwe	195.93	13.82	10.47	15.09	2.31	10.15	15.06	23.99	3.33	20.75	11.69	2.71	0.85	0.49	1.30	5.79	1.66	9.50	4.24
Benin	143.28	6.27	11.46	14.15	1.32	8.87	12.01	17.56	2.09	15.24	7.90	1.99	0.46	0.51	1.29	4.27	0.40	12.20	1.98
Burkina Faso	157.06	6.36	12.82	15.68	1.53	7.49	16.68	21.58	2.16	12.68	13.70	1.83	0.52	0.60	1.36	3.58	0.42	12.26	1.89
Cameroon	158.50	6.76	11.58	15.56	1.67	10.50	13.58	16.59	2.17	17.76	9.44	2.45	0.50	0.69	1.36	5.52	0.44	12.33	2.38
Cape Verde	162.02	10.11	15.60	9.86	1.05	10.68	13.94	11.14	3.65	21.21	8.94	4.75	0.47	1.06	0.83	6.70	0.41	11.98	2.11
Chad	144.22	5.26	13.46	14.73	1.57	11.16	9.50	19.64	1.70	15.70	8.22	2.07	0.50	0.55	1.37	3.16	0.39	12.24	1.48
Cote d'Ivoire	124.10	1.27	5.46	8.83	1.42	6.38	14.26	7.36	1.36	29.15	5.84	2.21	0.86	0.72	1.37	2.54	0.47	9.23	2.47
The Gambia	135.27	2.08	5.66	38.76	0.75	7.45	10.19	13.43	1.59	6.61	6.64	2.10	0.63	0.45	1.40	2.87	0.36	10.81	1.87
Ghana	151.44	3.83	7.67	16.33	1.62	6.83	16.73	17.24	4.07	14.84	9.47	2.52	0.20	0.92	1.20	7.82	0.29	12.10	2.62
Guinea	177.50	2.30	14.52	35.96	1.65	9.44	12.94	27.96	2.32	11.00	7.73	5.73	0.83	1.45	1.42	2.79	1.13	10.54	2.37
Guinea-Bissau	170.49	8.04	15.18	18.01	1.75	10.22	14.86	24.37	2.46	13.19	10.79	2.44	0.63	0.66	1.51	4.71	0.44	12.39	2.35
Liberia	136.66	5.69	10.65	14.94	1.24	7.23	11.87	16.11	1.86	15.71	9.02	1.83	0.42	0.45	1.10	3.58	0.37	12.24	1.70
Mali	150.25	2.32	14.61	31.75	0.90	6.61	11.73	12.83	1.12	7.78	7.83	1.91	0.58	0.28	1.02	2.87	0.89	13.55	1.14
Mauritania	153.38	4.98	9.63	12.53	1.17	9.15	15.29	16.76	2.61	21.69	9.55	2.29	0.44	0.50	1.24	4.96	0.56	12.28	2.49
Niger	135.44	4.26	11.92	21.74	1.18	7.74	8.39	19.56	1.72	13.75	6.62	1.71	0.41	0.41	1.23	2.64	0.37	12.16	1.43
Nigeria	151.28	3.47	4.01	3.59	1.21	7.67	28.61	14.24	1.10	32.85	9.22	1.27	0.67	0.23	1.17	4.02	0.73	10.53	2.59
Sao Tome and Principe	160.91	4.16	15.52	1.99	1.03	14.48	15.74	21.52	3.74	9.32	13.77	1.23	0.22	0.62	2.04	2.22	0.26	11.83	3.34
Senegal	158.14	4.92	11.97	14.42	1.48	10.76	13.40	17.62	2.07	20.58	8.26	2.12	0.47	0.52	1.39	4.23	0.47	16.93	2.07
Sierra Leone	144.94	4.92	12.19	13.56	1.49	9.55	13.40	19.21	2.01	14.76	9.30	2.00	0.48	0.59	1.24	3.58	0.39	12.14	1.90
Togo	134.86	4.79	11.28	11.45	1.32	8.56	13.68	17.35	2.09	13.16	7.66	2.06	0.46	0.51	1.18	3.75	0.40	12.23	2.08
American Samoa	252.74	1.31	12.57	9.83	1.59	23.70	34.97	9.20	17.15	33.04	18.49	3.90	2.18	2.82	1.05	5.13	1.12	28.98	13.34
Bermuda	285.92	4.09	7.72	3.09	3.84	21.45	32.69	4.13	7.92	59.51	35.95	4.32	0.53	1.74	0.80	7.62	6.72	22.58	4.85
Greenland	516.29	10.76	11.40	5.85	2.48	71.02	18.76	10.47	1.31	11.25	38.97	6.55	5.91	3.80	2.40	16.07	2.40	247.06	7.26
Guam	234.94	2.51	5.27	11.49	2.35	35.61	27.74	7.72	9.82	17.93	23.83	6.25	5.19	2.24	0.91	5.88	0.97	22.70	6.17
Northern Mariana Islands	251.57	1.69	7.33	7.49	3.33	29.70	28.54	11.87	14.20	24.34	17.76	15.08	2.34	3.48	0.86	5.64	1.45	36.55	3.49
Puerto Rico	224.73	2.16	6.38	5.21	2.16	10.92	34.71	6.13	8.27	33.78	30.37	2.95	0.45	1.14	0.72	4.80	2.63	22.13	3.59
Virgin Islands, U.S.	346.50	3.87	9.96	4.27	3.87	20.85	44.83	10.15	8.64	93.99	43.53	4.35	1.04	2.27	1.60	7.75	5.04	21.03	7.94
South Sudan	157.56	11.53	7.96	8.12	1.68	9.69	10.45	22.95	2.05	13.14	11.12	2.67	1.51	0.95	1.12	3.18	0.84	13.21	2.91
Sudan	110.60	4.42	13.94	3.35	2.15	7.77	9.44	2.71	0.72	8.95	8.31	1.39	0.46	0.31	1.20	2.28	0.49	13.77	1.33

Age-standardized Incidence change from 1990 to 2017(%)

	Total	Esophageal cancer	Stomach cancer	Liver cancer	Larynx cancer	Tracheal, bronchus, and lung cancer	Breast cancer	Cervical cancer	Uterine cancer	Prostate cancer	Colon and rectum cancer	Lip and oral cavity cancer	Nasopharynx cancer	Other pharynx cancer	Gallbladder and biliary tract cancer	Pancreatic cancer	Malignant skin melanoma	Non-melanoma skin cancer	Ovarian cancer
China	27.86	-36.89	-14.94	-4.14	30.15	53.43	94.73	9.09	13.35	112.32	84.10	79.71	-46.05	33.30	23.80	49.88	110.32	88.63	46.23
North Korea	5.03	-6.16	-2.21	-5.34	6.88	0.56	32.09	15.66	-5.91	10.08	16.79	3.64	7.79	-12.59	-7.33	4.79	2.54	14.16	14.78
China Taiwan	68.90	62.51	-17.00	48.85	21.84	34.55	189.26	-58.79	236.73	130.14	141.86	207.88	-26.37	248.78	26.39	75.37	85.96	103.35	119.25
Cambodia	-10.33	-43.40	-50.56	-13.67	-23.60	-16.60	42.46	-31.41	-6.13	38.39	5.58	3.68	-15.85	-2.36	-20.88	17.18	5.51	3.05	33.36
Indonesia	8.57	-12.91	-29.39	17.30	0.81	12.89	15.83	-16.77	4.24	72.37	18.05	7.85	-0.48	6.51	-5.86	45.56	21.67	5.85	32.59
Laos	-14.62	-46.74	-53.94	-19.22	-34.63	-18.25	23.77	-32.02	-15.57	61.77	-1.59	-20.92	-30.55	-22.06	-25.11	15.64	0.37	4.84	29.58
Malaysia	17.62	-7.31	-35.27	27.98	-0.77	0.35	71.23	-30.78	49.79	67.15	31.14	-2.95	-19.47	16.76	-13.42	50.85	40.35	0.18	47.65
Maldives	-15.71	-59.22	-67.59	-27.32	-33.64	-47.48	30.86	-70.27	-3.02	54.14	15.61	29.43	-52.41	-26.00	-27.46	3.17	8.48	-1.40	3.97
Myanmar	-12.63	-27.36	-54.94	-14.70	-34.56	-20.12	15.65	-33.36	-2.35	61.87	5.45	-5.51	-24.87	-13.71	-23.12	20.74	5.35	2.44	25.71
Philippines	36.10	-11.63	-39.46	-7.64	3.22	11.43	128.00	102.35	3.16	112.47	181.91	-19.77	22.63	-7.10	-39.01	46.10	52.97	27.56	74.83
Sri Lanka	21.68	-12.66	-46.30	70.60	44.55	-5.14	87.19	1.12	100.79	32.89	69.35	17.55	-18.51	-6.69	-47.70	38.82	56.26	51.19	61.24
Thailand	-3.41	-2.45	-44.74	-2.25	-18.88	-32.87	70.87	-44.05	31.12	80.72	29.26	-22.92	0.03	0.95	-12.06	4.58	38.40	-36.85	17.22
Timor-Leste	11.85	-31.89	-36.29	6.51	-2.48	12.86	63.38	-15.88	10.63	99.90	43.06	12.78	3.99	7.67	-6.25	42.68	34.51	7.02	52.47
Vietnam	16.23	-7.72	-49.62	4.18	34.71	15.08	43.26	-11.96	42.94	53.26	51.92	23.96	15.19	58.94	-0.79	71.11	47.25	62.50	33.54
Fiji	23.39	29.67	-13.13	17.01	22.38	-3.65	32.72	-9.92	23.17	13.02	26.61	2.04	1.42	37.12	-3.95	27.01	10.83	172.51	40.82
Kiribati	1.99	15.43	-23.64	-1.84	-0.25	-0.42	20.38	-11.38	5.36	-10.92	9.04	27.71	9.25	20.44	-5.41	28.46	9.12	34.31	43.86
Marshall Islands	19.78	0.99	-17.46	8.95	13.45	8.98	62.00	4.35	35.94	55.03	21.87	21.45	20.85	27.61	-8.20	46.38	10.90	23.12	94.97
Federated States of Micronesia	8.76	-12.34	-24.37	-8.79	-6.27	-1.27	55.12	-18.12	20.44	13.21	15.35	11.50	-2.21	5.75	-22.09	27.91	-2.42	48.61	78.25
Papua New Guinea	6.39	-8.66	-15.63	1.78	4.62	7.21	20.63	-12.94	9.88	30.66	8.02	12.77	4.41	8.54	-8.08	29.53	5.41	76.96	58.70
Samoa	7.10	0.74	-12.30	-2.65	-11.55	-4.16	7.11	-7.96	15.84	9.38	9.57	-16.53	-17.02	-0.10	-5.36	9.30	-4.49	37.49	21.70
Solomon Islands	0.64	-8.56	-24.33	-5.20	-8.35	-10.71	41.30	-14.28	13.34	2.20	13.79	9.34	-2.08	10.40	-12.73	26.20	-7.94	15.37	75.90
Tonga	3.57	5.31	-21.08	3.81	8.69	-9.03	26.63	-20.50	26.78	10.73	19.45	30.69	16.49	22.75	-7.03	23.76	13.90	-14.51	71.13
Vanuatu	12.61	-0.01	-15.96	-24.35	4.94	-2.24	38.93	-8.29	24.33	33.03	12.47	18.82	16.16	17.41	2.26	38.70	13.60	62.72	80.63
Armenia	9.52	-30.66	-37.51	3.58	-24.00	-2.05	33.07	-22.94	113.23	235.60	27.08	-0.52	-17.01	-17.46	77.92	24.23	101.60	2.34	3.19
Azerbaijan	15.48	-0.54	-15.36	11.07	3.83	3.80	50.53	15.22	71.11	132.05	30.12	58.34	23.47	84.17	3.35	56.82	47.97	4.26	36.34
Georgia	33.44	58.76	-4.03	43.75	6.06	28.10	21.39	-12.26	142.67	150.60	34.22	59.00	-28.66	80.17	75.12	78.49	27.36	16.93	93.79
Kazakhstan	-16.09	-64.27	-55.15	-41.66	-47.38	-48.88	34.64	-15.05	8.13	74.39	6.18	-7.75	-12.44	-25.95	-24.81	313.89	21.59	2.44	23.56
Kyrgyzstan	-7.39	-55.35	-50.51	3.15	-61.00	-52.46	-13.73	-21.82	30.58	17.47	-31.48	-41.02	-17.36	6.68	-28.77	-0.87	-15.93	93.73	-0.88
Mongolia	-6.50	-28.20	-40.10	57.41	41.05	-31.36	43.05	-34.26	36.10	21.82	-2.56	-50.50	0.99	-23.28	-26.66	57.30	12.78	-2.02	62.51
Tajikistan	-5.16	-49.64	-25.35	1.71	-35.03	-40.38	47.94	-35.48	151.75	32.24	6.80	6.34	-12.69	-0.34	-16.74	14.68	30.00	-0.46	20.25
Turkmenistan	-5.84	-71.90	-49.50	49.79	-51.03	-27.52	103.72	30.96	-26.50	71.78	28.81	13.66	5.57	4.56	-57.76	152.01	-12.90	-3.81	11.70
Uzbekistan	4.11	-68.10	-39.00	29.44	10.62	-28.76	79.06	20.83	100.28	79.12	28.20	42.85	13.10	77.82	8.40	88.55	56.00	-0.80	71.17
Albania	20.07	-31.15	-23.23	-28.89	-13.56	-3.70	126.85	-8.38	91.33	85.37	37.55	-17.88	-37.97	-12.68	5.02	79.41	67.06	4.10	50.64
Bosnia and Herzegovina	29.40	-17.12	-23.62	39.80	-25.11	6.36	109.65	-2.03	161.91	117.72	81.79	-1.75	80.30	9.09	-42.34	26.88	120.42	5.46	69.52
Bulgaria	16.07	-18.27	-43.39	-12.85	44.84	17.84	41.86	6.60	45.63	67.93	47.54	42.96	47.85	93.17	2.67	42.56	136.25	-24.14	38.23
Croatia	10.63	-34.24	-44.97	97.33	-31.49	-3.50	11.30	-47.74	46.63	65.19	33.55	-35.87	-51.55	-9.94	-19.10	9.80	98.97	-0.62	-18.23
Czech Republic	9.52	1.51	-51.67	-21.79	-24.75	-27.29	-5.69	-43.37	-0.13	81.80	-17.52	-0.62	-47.75	61.59	-38.70	-0.99	104.77	87.30	-20.86
Hungary	4.05	-14.85	-55.53	-30.85	-9.04	-0.74	-0.73	-48.80	-12.81	20.07	9.79	-1.29	-12.75	58.98	-52.19	4.51	64.55	90.64	-22.83
Macedonia	22.24	-1.63	-38.98	-5.65	21.46	28.90	36.15	-12.57	118.91	110.17	51.56	-0.74	-26.24	25.14	-24.47	32.99	69.13	3.93	28.39
Montenegro	13.70	0.58	-16.22	0.69	10.65	6.28	36.64	-17.44	45.93	48.07	21.13	2.14	-20.13	9.69	-13.93	17.11	53.94	1.83	11.10
Poland	30.67	-19.34	-48.08	-11.03	-16.94	-7.24	40.50	-45.06	88.84	135.62	23.27	22.75	0.30	64.57	-30.08	-8.17	133.49	250.48	-1.09
Romania	56.22	52.26	-30.98	121.04	38.23	26.12	49.76	-19.08	57.82	137.05	84.41	73.71	113.29	190.61	-37.35	37.15	157.30	194.50	7.39
Serbia	29.53	-4.29	-29.70	-8.55	-2.26	9.87	33.72	-30.57	84.32	46.29	27.47	-11.79	-23.03	15.01	-19.34	24.20	52.08	158.42	12.67
Slovakia	25.19	-13.44	-34.42	5.32	-41.15	-20.59	39.99	-14.03	34.23	89.91	39.80	-38.81	-35.02	54.12	3.82	14.38	118.21	72.26	5.65
Slovenia	20.04	-35.43	-42.66	37.14	-33.60	-5.51	-3.71	-49.82	7.38	116.54	17.37	-36.81	-53.38	17.97	-24.62	9.01	112.31	106.27	-28.22
Belarus	20.01	24.03	-41.96	13.01	-14.57	-22.26	11.11	-6.77	12.57	213.27	29.08	-20.56	35.21	157.10	1.91	15.26	187.71	51.28	-18.24
Estonia	28.90	22.58	-31.84	13.69	-14.34	-22.92	12.17	-43.91	54.09	216.83	24.83	-8.70	-36.22	72.62	-13.73	11.09	160.12	86.38	-17.28

Latvia	36.78	53.32	-32.24	30.45	5.41	-9.49	23.24	-36.68	90.67	182.83	23.23	18.55	-40.15	122.54	-16.62	7.84	158.99	141.57	-7.44
Lithuania	27.12	48.56	-31.78	54.25	14.44	-7.91	18.20	-35.60	78.72	182.69	27.90	8.61	-25.90	179.57	-13.83	9.92	145.36	22.62	-18.42
Moldova	0.67	-23.78	-45.40	197.95	8.02	-26.07	8.25	-28.04	31.31	112.31	19.28	-15.36	17.30	68.42	-54.03	9.69	60.97	-4.07	-25.86
Russian Federation	15.82	-21.45	-40.58	-9.55	-22.81	-20.35	44.62	-1.86	3.47	104.17	29.53	1.65	-29.57	35.61	-10.47	6.42	88.35	38.88	-11.37
Ukraine	12.94	3.10	-37.17	72.30	-12.83	-23.88	10.42	-40.60	89.85	144.31	21.47	61.62	64.47	101.40	12.51	53.33	118.40	1.36	31.57
Brunei	15.96	-32.62	-42.27	-15.52	-54.23	-10.40	89.60	-21.52	49.11	99.03	40.53	-7.88	-5.24	41.97	-8.95	28.42	134.76	-4.98	86.47
Japan	6.03	-4.28	-49.70	-32.90	-23.25	4.22	64.65	-1.06	82.16	125.12	23.77	38.34	6.35	128.41	-6.83	15.27	94.95	23.29	17.52
South Korea	24.28	-35.61	-40.80	-18.18	-45.46	53.05	119.09	-47.18	-36.80	260.00	127.25	16.59	-24.70	136.17	-7.95	20.69	328.99	24.68	89.58
Singapore	-1.91	-47.67	-48.05	-3.54	-41.63	-28.15	27.59	-59.90	94.23	144.23	2.38	-29.44	-59.80	8.05	-18.50	28.95	154.22	1.78	-3.64
Australia	-9.30	-2.64	-23.11	156.24	-29.38	-9.11	9.22	-45.19	22.19	63.14	-4.26	-19.71	-22.66	2.04	-28.26	11.26	41.37	-20.31	-21.03
New Zealand	12.05	5.52	-34.26	115.42	-40.01	-10.61	-12.12	-62.32	1.09	0.13	-19.09	-22.88	-33.61	-5.71	-17.97	9.57	28.11	27.70	-28.23
Andorra	12.27	1.02	-18.52	54.07	-20.19	-8.78	27.35	-15.16	46.09	64.53	6.07	-16.90	-24.23	9.04	-30.67	1.62	69.61	1.23	-0.97
Austria	-1.05	23.11	-40.90	51.20	-31.24	15.30	-10.77	-56.98	-8.33	59.88	-22.64	0.59	-7.67	95.53	-38.36	20.64	68.65	-33.23	-38.60
Belgium	8.45	25.50	-40.71	56.07	-44.64	-17.29	-11.80	-37.64	20.85	29.59	-10.45	3.43	-38.01	43.56	-50.94	0.36	86.02	96.28	-36.85
Cyprus	60.82	8.44	5.96	32.32	2.59	34.83	49.71	-22.08	55.41	138.05	46.07	8.06	1.12	43.72	-30.13	42.32	187.98	130.31	1.08
Denmark	28.04	14.03	-34.75	31.42	-31.80	-2.64	2.94	-50.27	10.23	126.31	60.48	0.26	-33.04	68.94	-20.25	9.58	117.76	83.55	-24.92
Finland	11.99	-6.21	-55.30	57.09	-25.67	-21.17	17.39	-34.29	12.96	123.92	17.26	7.69	-43.57	43.66	-34.30	-1.70	109.60	2.41	-25.41
France	12.45	-47.87	-35.97	16.51	-50.74	11.61	10.13	-33.26	25.19	31.77	-3.75	-47.39	-52.22	-27.58	-52.53	20.38	107.02	50.93	-25.74
Germany	25.08	38.37	-38.76	83.64	-11.20	6.89	15.91	-46.01	-10.44	102.59	-9.45	-4.40	-41.74	45.13	-37.25	21.91	75.80	247.99	-39.04
Greece	20.84	-20.74	-13.78	-48.29	-1.63	7.41	35.62	-14.42	93.67	69.86	60.11	15.76	11.02	46.91	17.71	26.32	154.84	1.48	32.83
Iceland	3.79	-2.57	-48.94	67.06	-20.78	1.48	-17.46	-49.64	-10.65	34.52	6.53	-4.40	-45.24	22.35	-48.38	1.34	88.89	4.26	-40.05
Ireland	18.22	-3.18	-26.55	66.90	-4.72	-10.70	3.95	-22.26	52.79	81.60	2.45	-25.88	-30.69	57.89	-29.86	-6.39	184.30	16.73	-15.98
Israel	6.13	-17.01	-35.59	17.17	12.93	-2.42	3.41	-13.07	85.76	38.37	4.45	21.47	-58.56	-19.05	-60.31	9.20	54.17	-5.56	-23.89
Italy	9.35	-33.67	-39.33	-1.81	-40.29	-16.21	-1.59	5.02	207.64	59.17	8.78	-29.48	-21.11	-8.85	-14.75	14.73	83.27	31.76	-13.04
Luxembourg	8.14	-13.27	-34.76	44.53	-32.43	-3.60	3.05	-40.41	19.88	51.04	0.87	-28.77	-42.72	5.76	-43.80	15.62	105.07	0.22	-22.46
Malta	24.01	-5.83	-30.58	16.97	-16.76	7.96	10.75	-31.81	49.31	72.76	35.25	-2.96	-16.43	38.02	-35.89	18.19	137.37	22.89	-8.64
Netherlands	29.33	86.55	-27.38	177.30	-20.06	-3.43	2.26	-21.88	47.92	64.34	21.25	11.70	-26.82	62.78	-39.99	21.60	134.13	160.00	-25.93
Norway	11.33	11.33	-48.68	81.13	-26.80	9.38	12.64	-45.63	27.50	122.58	16.15	-24.39	-36.65	20.44	-17.15	0.07	65.55	1.13	-23.42
Portugal	32.33	-25.23	-39.04	26.75	-26.41	7.94	5.86	-39.34	27.41	69.36	40.29	-1.98	-20.17	180.42	-29.21	-1.54	199.20	295.46	-8.57
Spain	9.35	-20.07	-36.22	8.24	-40.11	6.28	-0.36	-28.07	34.98	91.74	40.76	-35.35	-48.20	26.29	-30.88	14.31	96.48	-12.03	-14.39
Sweden	-21.91	-2.37	-54.17	4.66	-24.66	0.85	12.42	-29.37	2.29	72.83	5.15	-13.58	-30.77	31.00	-87.20	-12.04	77.99	-59.66	-42.31
Switzerland	1.98	-15.11	-46.77	35.94	-48.40	-18.57	-18.05	-56.94	-2.05	21.48	40.33	-28.41	-47.23	26.29	-50.14	-3.91	47.68	22.02	-31.15
United Kingdom	14.62	16.43	-33.50	157.34	-5.89	-13.58	-8.00	-42.24	73.98	52.38	-2.32	30.28	-33.84	71.82	-24.46	8.51	81.00	44.62	-24.29
Argentina	-1.80	-37.11	-36.15	-7.00	-28.48	-19.49	26.63	2.14	3.43	54.35	28.13	6.62	-39.26	-43.96	-37.54	9.00	126.31	-19.33	7.74
Chile	3.19	-49.64	-39.33	-10.96	-23.30	-11.38	36.41	-49.96	94.25	116.87	65.46	-1.93	-24.83	-12.60	-39.82	7.90	124.12	-8.14	0.70
Uruguay	6.19	-35.00	-32.27	40.00	-33.35	-19.29	16.33	-10.68	11.42	66.27	19.99	-0.87	-23.64	-19.49	-21.60	26.51	148.33	25.93	31.25
Canada	26.26	10.65	-18.84	95.37	-34.78	-16.34	-9.39	-17.57	28.22	12.53	-3.82	-29.50	-24.92	4.38	-33.70	-7.93	68.01	747.79	-19.85
United States	9.95	6.74	-21.67	124.68	-13.79	-16.77	-15.50	-22.25	39.16	6.43	-15.14	-14.57	-28.68	2.86	-10.65	13.27	60.11	15.38	-21.77
Antigua and Barbuda	22.25	-16.35	-33.05	-16.58	0.59	-6.99	46.92	-24.70	94.77	50.52	42.94	-14.15	17.11	-27.44	-65.82	201.40	44.99	13.72	335.52
The Bahamas	15.18	-19.69	-31.69	-17.40	-0.23	-14.79	40.04	-13.03	57.02	42.78	25.74	-17.16	20.10	-24.20	-60.25	146.27	35.26	2.09	248.38
Barbados	19.88	-26.12	-38.48	-26.17	9.28	-9.75	34.70	-23.97	68.01	61.01	44.65	-13.67	30.34	-23.23	-67.77	326.41	50.05	4.49	384.46
Belize	21.57	23.37	-25.64	16.22	30.09	21.99	49.33	-11.87	36.87	62.92	47.64	20.87	51.53	13.82	-57.02	226.15	80.40	5.64	241.13
Cuba	14.56	33.83	-15.04	-21.72	49.82	3.15	27.98	-27.46	88.92	62.24	36.02	10.86	21.19	-1.15	-70.13	204.44	57.42	-32.08	244.88
Dominica	27.64	4.25	-24.56	-7.37	32.20	12.93	31.06	-15.63	58.74	89.44	44.84	8.25	62.89	0.99	-63.72	302.75	62.41	7.96	297.40
Dominican Republic	53.40	22.64	4.21	72.47	7.69	33.54	159.63	13.92	7.36	171.00	87.71	18.56	131.06	58.64	-50.64	223.64	76.93	4.95	204.61
Grenada	4.74	-10.15	-38.69	-33.49	11.69	2.42	32.90	-42.11	51.89	46.10	19.64	-6.70	-20.96	-29.20	-72.32	339.72	-6.98	18.53	321.35
Guyana	13.93	-7.35	-35.03	-7.00	3.13	11.89	56.11	-11.10	76.97	28.40	23.90	-0.80	34.50	-13.60	-60.98	198.27	44.71	5.30	374.14
Haiti	-5.77	-22.94	-34.44	-15.94	-18.66	-22.86	34.74	-32.74	19.04	45.62	19.15	-1.38	-6.99	-23.71	-39.80	76.73	8.80	1.61	105.17
Jamaica	43.30	5.33	-28.78	-2.72	63.23	35.45	66.99	-10.15	171.11	109.14	66.14	-2.86	108.61	14.29	-36.84	204.74	85.43	-5.26	406.01
Saint Lucia	3.99	-20.67	-39.92	-31.22	-0.18	-10.73	13.46	-38.40	45.50	47.07	18.93	-16.67	17.08	-24.69	-70.81	278.55	32.53	9.78	255.88
Saint Vincent and the Grenadines	17.47	14.23	-28.42	-1.42	33.37	11.82	17.02	-28.62	36.90	84.80	27.71	2.43	30.50	-4.43	-64.51	224.57	13.81	19.43	264.08
Suriname	19.64	-0.23	-30.61	-1.22	18.84	15.03	43.39	-14.38	69.23	70.16	46.70	3.57	35.91	-11.10	-63.98	327.80	45.99	3.27	356.34
Trinidad and Tobago	2.02	-39.59	-55.18	-49.41	-20.77	-11.08	13.21	-30.30	59.13	23.70	12.37	-24.13	21.95	-29.84	-69.17	262.08	20.92	65.31	507.51
Bolivia	-3.55	-25.82	-40.78	-1.01	-34.21	-8.31	50.03	-39.17	16.08	71.59	27.95	4.18	-24.45	-27.86	-30.24	64.93	27.83	4.03	89.90
Ecuador	26.46	-40.18	-35.39	1.03	-21.72	9.81	97.12	-15.90	15.09	82.82	73.79	19.48	3.45	-28.66	-41.46	157.38	51.44	53.60	301.00

Peru	25.58	-33.10	-27.19	5.21	-48.41	-26.07	55.52	-20.43	22.13	90.29	73.57	-12.18	-17.74	-50.23	-27.48	103.20	73.54	142.04	163.56
Colombia	-3.88	-56.18	-32.92	-42.45	-47.99	-26.28	29.31	-32.47	10.15	62.12	41.50	-32.89	-48.95	-45.06	-50.26	-21.59	71.41	-7.19	8.97
Costa Rica	22.80	-33.87	-19.94	14.84	-20.91	-14.41	52.66	-41.87	91.79	114.22	88.79	-15.64	9.89	-0.18	-53.04	85.73	95.82	6.77	97.02
El Salvador	35.91	13.68	26.74	62.95	17.57	34.42	141.01	20.52	76.18	151.72	149.81	-1.84	-0.06	-22.58	-12.86	165.92	83.17	1.37	237.36
Guatemala	19.82	17.17	-7.61	89.53	-32.30	-15.77	70.98	45.59	34.11	136.16	79.41	-5.22	29.03	-25.43	-63.74	143.71	150.22	-0.01	262.81
Honduras	20.08	22.56	-11.71	30.16	-5.38	5.26	54.86	-21.37	112.82	81.43	67.07	42.32	-0.31	19.50	15.37	84.89	73.40	1.16	161.69
Mexico	4.31	-26.17	-9.39	-9.48	-35.33	-35.19	65.69	-49.83	80.15	84.26	88.93	-1.09	-23.26	-12.71	-46.94	-5.37	118.86	-4.10	49.39
Nicaragua	3.69	-31.41	-24.42	7.02	-35.07	-8.97	72.74	-43.48	22.34	66.43	56.78	-9.92	-19.02	-26.05	-47.13	89.53	41.32	2.58	172.15
Panama	17.93	-10.98	5.82	16.56	-38.35	-25.50	52.61	-36.74	102.10	88.96	44.95	-30.43	-2.88	-12.85	-49.67	49.74	55.97	-2.21	84.12
Venezuela	16.57	-24.53	-22.43	-26.72	-1.64	1.16	69.06	-17.90	9.57	126.06	52.01	-1.55	13.53	-12.78	-58.28	216.76	91.01	-2.48	368.98
Brazil	20.17	-18.59	-40.47	4.04	-1.95	-9.51	42.92	-26.49	33.49	68.10	53.50	3.47	18.49	11.55	-34.15	12.92	75.23	32.35	16.76
Paraguay	27.38	8.52	-22.75	-5.60	17.32	32.92	75.75	-11.05	-1.90	101.17	91.69	1.03	1.15	28.37	-24.79	119.30	69.58	2.00	129.93
Algeria	14.63	-11.52	-39.21	23.58	-21.95	-16.67	89.68	-31.14	47.47	94.06	25.69	-1.61	-10.16	15.47	-23.21	55.57	72.94	-10.13	48.19
Bahrain	-25.77	-67.10	-62.62	-24.21	-59.00	-66.15	20.39	-48.05	38.59	67.52	-2.83	-43.57	-54.80	-42.27	-41.31	-27.40	31.40	-9.83	-26.20
Egypt	30.66	16.50	-23.64	47.74	9.00	24.35	93.56	-13.36	69.76	107.58	48.61	25.22	-4.19	35.23	-8.57	53.37	67.37	-32.51	49.16
Iran	27.00	-18.30	-28.74	21.75	-2.93	9.93	132.94	-25.22	117.37	218.20	63.34	39.72	-21.30	29.96	3.85	84.35	46.09	-14.70	98.04
Iraq	-37.90	-56.01	-60.92	-27.40	-61.74	-44.84	-29.88	-56.23	5.50	-3.41	-31.14	-46.28	-75.09	-48.66	-51.05	-18.75	-26.01	-63.01	-1.80
Jordan	11.65	-18.31	-42.86	7.81	-57.55	-2.90	47.72	-53.35	30.39	210.50	26.78	8.83	-38.58	7.59	-22.75	52.30	39.69	-26.48	13.24
Kuwait	1.16	-32.25	-46.96	12.69	-41.17	-31.12	-0.25	-56.60	39.23	140.03	32.93	-42.61	-52.30	-36.42	-56.56	1.68	12.92	-5.90	-37.71
Lebanon	64.35	-11.54	-33.43	-7.29	-8.06	-6.29	156.35	-41.61	114.00	224.73	62.68	29.51	-32.82	30.58	-22.13	35.63	243.97	5.28	41.49
Libya	42.66	24.98	-6.59	12.41	14.08	-2.39	167.18	3.67	98.81	141.49	52.84	31.24	-6.21	22.72	-14.29	39.56	113.73	1.45	82.35
Morocco	22.05	-5.95	-26.83	8.50	-1.31	3.58	73.81	-28.13	44.37	101.39	41.78	22.14	-9.36	17.30	-12.91	50.46	56.05	2.88	51.76
Palestine	2.69	-25.95	-42.08	-10.88	-35.21	-14.36	34.26	-38.51	10.19	52.53	5.46	-6.97	-23.66	-8.13	-14.32	21.86	13.13	3.85	32.43
Oman	20.21	-13.71	-46.68	-7.71	-18.89	-16.39	92.71	-51.01	54.96	150.79	46.28	-6.03	-37.78	9.24	-28.17	58.26	84.50	-1.61	19.70
Qatar	6.13	-53.60	-44.71	-33.80	27.29	-33.44	40.04	-42.44	106.63	195.53	4.09	-12.38	-5.20	27.85	-66.41	-26.05	48.33	-5.70	12.07
Saudi Arabia	70.89	-5.73	-30.98	0.95	6.21	19.69	226.49	-11.01	187.85	218.22	149.21	37.94	-10.67	49.94	0.38	109.67	163.34	-61.52	90.82
Syria	20.88	-1.71	-31.25	-10.01	-4.61	1.10	87.33	-32.90	44.97	151.40	39.48	15.10	-11.39	22.05	-21.24	58.64	72.21	3.48	55.62
Tunisia	25.64	0.17	-27.31	3.34	0.83	-0.86	103.60	-25.80	73.15	116.79	40.50	16.61	-18.97	27.21	-17.19	44.53	89.18	5.09	49.72
Turkey	12.46	-42.15	-51.84	-11.04	-26.44	-19.98	83.48	-50.41	58.24	196.17	26.11	-35.66	-39.20	-10.09	-40.31	1.49	94.12	107.77	-0.82
United Arab Emirates	36.05	38.49	-9.22	27.86	34.03	14.82	72.62	-39.19	44.67	205.65	50.32	16.94	-0.95	5.97	-20.74	67.06	79.11	6.49	23.44
Yemen	11.75	-19.07	-13.01	-4.76	-2.05	-3.38	82.51	-20.75	33.33	128.90	38.17	13.05	-23.47	-6.98	-15.51	61.95	24.61	5.26	70.96
Afghanistan	0.39	-15.71	-9.80	-11.66	-7.70	-9.39	33.40	-10.42	23.47	19.13	21.02	-2.62	-26.81	-6.63	-4.24	22.33	4.57	0.27	51.00
Bangladesh	-16.12	-39.24	-50.12	-7.42	-47.98	-8.20	88.37	-54.28	-9.80	-5.84	-12.53	36.66	-39.99	-13.76	-25.20	14.12	4.95	-7.21	33.37
Bhutan	-11.15	-42.61	-43.24	17.58	-38.15	-10.84	21.35	-61.62	-12.89	54.46	22.14	-13.73	-29.72	8.17	-0.52	58.43	30.13	6.22	38.18
India	13.15	-11.46	-30.52	53.92	-21.76	8.46	86.48	-20.70	33.10	39.15	37.53	14.93	-7.47	25.17	33.84	87.11	49.16	-10.45	77.85
Nepal	-4.79	-20.22	-32.09	50.01	-29.96	-5.68	40.23	-47.35	-3.25	36.97	20.07	-10.49	-28.05	15.00	11.03	76.76	17.23	1.57	66.46
Pakistan	30.44	10.08	-5.68	28.44	-0.86	11.39	85.86	-4.38	46.99	41.70	47.09	33.99	2.87	34.68	13.28	71.05	46.00	-58.59	91.64
Angola	-13.09	-32.65	-49.53	-39.48	-28.45	-23.05	43.91	-31.26	5.21	17.57	6.01	4.31	-32.61	-1.92	-14.69	27.74	4.17	0.32	37.25
Central African Republic	-11.52	-28.16	-30.02	-36.31	-19.80	-14.46	20.29	-23.10	-10.56	3.60	0.04	-7.21	-20.73	-13.77	-16.23	0.76	-7.39	1.90	7.46
Congo	-7.05	-37.58	-43.71	-31.42	-29.56	-18.43	36.57	-23.74	0.64	51.58	5.16	-2.54	-25.43	-14.90	-18.66	19.26	7.95	1.33	26.00
Democratic Republic of the Congo	-7.99	-29.43	-27.36	-33.67	-18.67	-16.58	33.46	-12.90	4.38	0.84	4.18	-0.77	-18.29	-5.21	-10.54	-1.30	4.30	-0.21	13.04
Equatorial Guinea	-10.15	-34.89	-69.48	-21.51	-45.85	-7.72	75.34	-51.28	31.23	55.14	22.15	13.81	-48.22	25.40	-16.43	80.88	25.38	1.53	68.48
Gabon	-1.37	-24.90	-40.51	-15.96	-20.04	-5.44	31.86	-30.62	-1.80	53.87	10.19	-2.45	-20.27	-11.52	-25.99	29.67	10.84	1.85	22.08
Burundi	-28.87	-52.30	-43.39	-36.51	-42.20	-24.91	-18.04	-45.81	-38.06	11.78	-2.84	-20.60	-34.84	-35.71	-26.21	-13.91	-20.96	3.04	-26.01
Comoros	-14.21	-47.18	-41.57	-25.65	-37.07	-25.33	39.19	-33.48	-9.98	14.51	1.92	-3.80	-25.49	-22.25	-15.96	9.89	-2.20	-1.78	18.89
Djibouti	-5.49	-36.44	-33.86	-8.97	-23.73	-3.29	29.51	-34.95	-15.38	37.51	7.22	2.97	-13.35	-14.90	-17.42	29.49	-0.87	3.41	8.95
Eritrea	-8.02	-46.78	-42.57	-16.85	-38.89	-15.88	46.87	-26.27	0.43	15.89	9.90	4.61	-16.68	-12.77	-4.57	28.88	11.36	-0.63	38.44
Ethiopia	-23.47	-50.19	-61.42	-29.56	-44.27	-35.64	1.77	-54.14	-26.56	54.53	-17.05	-8.39	-34.98	-21.45	-22.98	-0.86	-0.88	2.76	1.04
Kenya	19.49	-9.76	-8.75	33.03	2.47	-5.76	40.59	-18.54	15.98	54.48	17.50	2.31	18.06	20.14	2.82	43.40	24.77	59.80	23.90
Madagascar	-14.06	-35.60	-28.38	-18.20	-28.89	-15.03	14.64	-23.26	-10.67	5.82	-1.05	-13.62	-21.24	-16.72	-7.10	3.56	-6.78	-1.10	-1.54
Malawi	0.82	6.00	-36.98	1.42	-15.72	-9.52	25.05	-20.24	-1.29	15.14	22.32	4.16	-24.99	10.58	-14.89	19.83	12.06	14.14	6.38
Mauritius	27.34	-26.14	-47.10	11.33	-32.43	-12.49	167.61	-38.24	-8.45	108.16	95.41	19.38	-14.98	36.71	-24.30	12.79	72.54	-0.03	69.33
Mozambique	1.27	2.78	-18.49	20.30	-13.91	10.49	32.05	-24.16	2.66	-5.15	19.23	7.53	1.98	17.92	-13.10	37.86	-8.29	-1.55	11.83
Rwanda	-27.43	-57.98	-57.57	-37.14	-53.92	-28.05	9.68	-50.18	-22.47	7.13	-2.01	-17.43	-45.00	-38.35	-22.05	1.13	-6.42	-1.26	2.85
Seychelles	49.36	-14.76	-48.23	225.92	5.76	-2.44	88.86	43.81	12.59	170.83	142.39	28.16	-17.00	64.65	-16.30	4.39	48.43	2.01	48.42

Somalia	-5.28	-35.53	-26.41	-8.86	-23.27	-10.27	22.33	-15.62	-9.72	8.96	13.57	7.93	-6.50	-8.12	3.14	14.26	8.21	-1.15	19.00
Tanzania	-6.73	-22.89	-33.65	-19.78	-29.01	-4.06	23.33	-30.67	-11.52	15.62	-1.26	-1.69	-14.62	-9.93	-11.04	21.34	2.03	-0.62	12.53
Uganda	3.54	7.15	-32.51	-0.47	-17.13	15.86	18.19	-35.87	-0.89	30.41	22.12	20.73	11.64	26.97	-26.55	67.56	24.73	-13.59	-0.45
Zambia	-22.79	-25.89	-42.57	-25.53	-37.09	-15.97	-2.31	-46.80	-27.15	-18.10	-6.98	-10.98	-27.29	-19.75	-21.07	7.15	-19.75	-3.95	-7.92
Botswana	2.71	-34.97	-41.18	-2.32	-35.45	-23.59	70.62	-10.62	53.23	35.90	15.89	-16.93	-23.43	-6.99	-19.97	32.43	12.08	2.41	45.35
Lesotho	19.71	5.46	-7.32	14.28	-0.47	25.41	78.13	16.56	53.79	20.54	43.85	14.33	20.19	43.90	11.67	63.67	16.04	1.85	78.64
Namibia	17.66	-19.96	-43.90	8.01	-23.78	-17.68	68.55	-24.59	-5.76	87.64	9.84	7.98	-31.46	3.22	-27.43	33.63	11.63	78.02	-0.46
South Africa	14.05	-23.41	-31.73	-44.03	-22.27	-10.56	23.25	-26.15	45.59	37.25	18.75	-18.91	-28.76	11.39	-10.98	14.71	24.74	47.91	16.89
Swaziland	4.80	-15.58	-25.36	54.70	-18.82	-9.72	54.09	-13.83	19.71	13.34	24.80	-7.42	-1.40	11.72	-12.67	31.13	6.12	0.98	30.82
Zimbabwe	2.88	7.72	0.91	-5.62	-2.12	-12.31	64.66	16.85	48.10	16.07	20.97	25.88	28.72	30.06	-6.31	43.71	26.94	-71.24	66.61
Benin	4.92	74.64	-20.69	-24.11	-0.75	-7.56	38.17	-6.92	32.63	60.79	19.99	4.35	-20.82	13.72	-19.67	76.70	-5.33	2.36	35.41
Burkina Faso	-18.00	3.35	-18.22	-68.10	8.53	-5.11	14.17	-18.39	19.47	42.94	11.16	-2.33	-29.17	13.54	-24.68	65.50	-11.72	2.12	16.35
Cameroon	2.63	52.60	-20.80	-28.54	8.73	1.18	24.37	-20.88	10.44	82.22	11.29	2.24	-28.42	9.11	-22.01	66.85	-8.05	2.25	28.32
Cape Verde	22.37	30.70	-36.11	-23.38	-24.61	30.76	55.20	-34.57	70.02	334.47	124.91	34.95	29.24	22.72	6.43	88.37	94.84	3.34	64.03
Chad	5.87	84.13	3.04	-47.86	39.35	34.15	18.56	-3.31	14.28	99.24	27.06	11.65	-8.08	34.63	-9.53	80.44	0.40	2.95	20.05
Cote d'Ivoire	13.82	21.18	-19.06	-6.85	-8.82	-13.76	33.02	-18.35	14.28	35.98	2.48	5.06	-9.67	-2.27	-13.56	33.32	9.49	80.05	30.49
The Gambia	17.10	12.15	-21.86	4.81	-8.67	-1.48	75.67	3.00	36.73	41.93	12.81	22.67	-1.48	10.64	-4.94	56.17	24.27	129.49	64.12
Ghana	2.43	13.25	-30.71	-2.27	25.77	10.46	53.26	-2.83	34.20	-12.76	19.46	15.08	-50.03	12.66	-5.59	55.85	29.25	1.80	52.67
Guinea	7.93	32.24	1.93	-0.28	40.36	12.90	39.90	-16.43	14.58	25.06	28.67	21.62	-9.41	8.41	-13.63	43.00	9.14	58.53	30.98
Guinea-Bissau	-7.08	51.84	-30.20	-39.97	-13.62	-20.35	37.72	-14.37	27.78	41.85	-5.00	-1.29	-30.12	-1.14	-20.90	61.13	-10.62	3.98	39.26
Liberia	-0.50	50.05	-16.60	-29.41	-7.15	-12.86	40.23	-12.05	34.12	62.65	15.81	-5.86	-30.54	-2.26	-23.46	52.77	-10.22	2.76	31.47
Mali	-8.29	-9.02	-36.27	-21.64	-25.53	-5.70	20.83	-36.97	-6.63	46.71	3.77	8.24	-40.17	-7.16	-19.61	25.59	8.22	92.91	16.43
Mauritania	1.73	17.07	-34.64	-35.93	-8.53	-8.03	30.15	-24.80	13.14	153.15	4.63	4.94	-35.70	-3.17	-25.70	63.46	5.58	3.35	26.36
Niger	-12.56	35.39	-18.51	-48.45	-3.38	-4.53	21.50	-5.97	20.02	59.27	-1.02	-2.56	-30.76	4.34	-20.82	45.47	-9.80	2.45	23.15
Nigeria	13.00	36.93	-28.05	-5.55	-24.39	-3.66	53.81	-13.33	36.03	49.82	8.48	20.61	-20.92	-2.85	-8.17	32.74	24.77	-26.84	56.50
Sao Tome and Principe	27.53	83.68	7.42	21.90	29.41	25.59	103.33	-3.30	52.59	68.37	63.07	44.37	10.26	49.06	6.76	78.74	37.02	1.95	67.08
Senegal	8.33	40.61	-17.49	-24.26	7.24	-5.69	47.59	1.88	33.69	102.17	-10.78	8.28	-16.44	15.39	-10.53	64.87	2.94	1.98	45.40
Sierra Leone	-8.30	61.38	-9.44	-69.47	10.53	-4.18	56.95	12.95	48.13	77.63	14.06	7.90	-12.86	23.99	-14.45	72.97	1.59	2.62	46.95
Togo	0.62	54.36	-15.95	-34.09	3.37	-3.36	38.09	-14.19	27.64	52.44	17.32	3.25	-24.55	9.07	-21.34	66.65	-6.53	2.72	28.62
American Samoa	28.55	13.37	-19.55	26.49	-10.74	-7.51	103.30	19.31	94.60	20.13	16.66	45.13	10.89	52.86	-24.37	37.91	2.37	20.32	124.33
Bermuda	1.21	-34.83	-29.87	-40.64	0.22	-27.35	-19.43	-59.02	15.91	80.21	11.37	-30.56	-9.55	-33.10	-77.86	334.80	51.68	4.73	-18.13
Greenland	-9.10	-18.75	-42.95	17.28	1.00	-32.95	-23.27	-45.87	4.73	75.77	-14.24	-27.16	-44.80	-16.89	-22.00	-12.50	9.00	11.33	-11.03
Guam	22.62	32.36	-10.00	60.92	4.36	4.14	60.74	8.94	18.72	5.41	26.49	46.99	22.78	53.95	-23.51	55.91	3.35	-1.16	177.44
Northern Mariana Islands	18.37	18.45	-27.27	15.29	1.54	-19.19	47.32	-15.68	63.09	60.33	7.33	55.76	-8.85	31.70	-17.26	33.77	23.90	24.72	65.43
Puerto Rico	10.88	-63.17	-39.48	-19.02	-44.62	-24.02	35.14	-16.27	73.19	16.11	55.46	-49.20	-25.95	-48.35	-45.91	155.98	83.03	0.27	197.17
Virgin Islands, U.S.	42.89	14.38	-7.50	5.96	36.40	20.77	48.20	-20.97	73.27	83.07	57.21	20.55	18.55	27.28	-45.97	130.29	100.95	8.07	107.97
South Sudan	-6.71	-32.43	-25.01	-18.74	-23.40	-2.13	9.69	-11.73	-14.92	5.08	5.71	-3.40	-7.95	-13.33	-11.51	9.42	-2.02	-0.53	5.90
Sudan	4.47	-8.91	-20.83	-7.89	-9.95	-5.84	50.39	-31.84	18.82	90.88	25.84	2.60	-32.03	-8.31	-19.93	62.10	15.60	3.67	42.65

Age-standardized Incidence in 1990

	Testicular cancer	Kidney cancer	Bladder cancer	Brain and nervous system cancer	Thyroid cancer	Mesothelioma	Hodgkin lymphoma	Non-Hodgkin lymphoma	Multiple myeloma	Leukemia	Other malignant neoplasms
China	0.17	2.27	3.38	4.51	1.07	0.14	0.89	2.19	0.65	8.58	5.18
North Korea	0.24	3.21	3.41	3.60	1.78	0.14	0.48	2.49	0.59	6.01	4.82
China Taiwan	0.30	2.47	5.83	3.98	2.72	0.16	0.45	4.27	0.89	5.42	9.03
Cambodia	0.13	2.77	3.33	2.90	1.94	0.37	1.17	3.26	0.74	7.46	5.45
Indonesia	0.01	2.31	3.11	3.27	1.63	0.19	0.73	2.75	0.58	6.08	4.71
Laos	0.15	3.16	3.63	3.45	1.90	0.39	1.28	3.72	0.72	8.14	5.62
Malaysia	0.43	2.41	4.31	2.10	2.51	0.18	0.67	3.49	0.73	6.88	5.64
Maldives	0.01	3.17	4.90	2.50	3.14	1.29	0.37	3.30	0.85	5.89	3.70
Myanmar	0.16	3.65	4.30	3.83	2.28	1.31	1.41	3.91	0.84	9.55	6.88
Philippines	0.23	2.96	1.82	2.75	2.86	0.23	0.41	2.21	0.59	4.90	10.61
Sri Lanka	0.05	8.35	2.19	1.46	2.30	0.46	0.98	1.98	0.78	6.73	4.97
Thailand	0.06	2.20	4.19	2.61	2.02	0.58	0.57	2.83	0.68	7.69	4.73
Timor-Leste	0.11	2.33	2.72	2.61	1.40	0.19	0.96	2.79	0.61	6.93	5.07
Vietnam	0.25	1.76	1.96	2.90	2.70	0.22	0.70	3.94	0.57	3.96	6.75
Fiji	0.93	2.17	2.40	1.53	3.58	0.24	0.30	1.77	1.01	5.85	5.74
Kiribati	1.74	3.22	1.85	1.22	0.61	0.15	0.25	1.90	0.92	4.81	5.60
Marshall Islands	0.49	3.68	2.89	1.82	1.81	0.31	0.51	3.15	1.48	6.10	6.00
Federated States of Micronesia	0.32	3.21	3.17	1.85	1.48	0.38	0.52	2.84	1.25	6.39	6.13
Papua New Guinea	0.48	2.55	2.36	2.01	1.36	0.37	0.72	2.74	0.88	6.90	6.16
Samoa	0.95	2.08	2.50	1.80	2.31	0.22	0.69	4.52	1.36	5.39	5.84
Solomon Islands	0.36	2.56	2.36	1.56	1.25	0.35	0.54	2.44	1.00	5.85	5.54
Tonga	10.05	3.05	2.68	1.60	1.24	0.19	0.26	4.95	1.67	3.97	4.25
Vanuatu	0.35	3.20	3.63	2.01	1.46	0.37	0.72	3.27	0.99	8.45	8.18
Armenia	1.01	1.59	10.34	5.34	0.71	0.75	0.23	1.81	0.56	6.17	14.33
Azerbaijan	0.17	8.43	4.14	4.21	1.43	0.13	1.19	1.69	0.58	5.18	6.09
Georgia	0.95	5.07	4.87	2.88	1.55	0.12	2.58	1.82	0.71	5.73	3.78
Kazakhstan	1.23	8.68	5.20	1.10	4.17	0.33	1.05	3.37	1.00	5.45	10.85
Kyrgyzstan	0.60	2.89	3.27	1.69	2.17	0.10	0.77	1.92	0.52	4.81	5.65
Mongolia	0.01	3.49	3.32	1.91	0.92	0.45	0.31	3.09	0.55	3.36	17.48
Tajikistan	0.01	4.94	2.05	5.61	0.37	0.26	0.31	2.58	0.58	4.92	5.85
Turkmenistan	0.65	6.35	1.79	1.14	1.41	0.15	0.83	1.31	0.73	4.63	6.23
Uzbekistan	0.40	2.98	2.31	3.07	0.42	0.14	0.83	2.25	0.47	5.04	4.10
Albania	1.03	3.59	1.69	5.37	1.89	0.14	1.23	2.02	0.59	5.18	9.17
Bosnia and Herzegovina	0.40	5.45	4.86	5.57	1.58	0.25	0.82	1.88	0.58	4.37	5.88
Bulgaria	2.49	2.82	6.49	5.50	2.45	0.11	2.14	3.08	0.80	5.37	5.98
Croatia	2.71	4.86	10.15	8.09	4.45	0.40	3.26	5.33	1.98	9.63	9.49
Czech Republic	4.64	10.85	12.51	5.84	4.69	0.42	4.15	6.42	2.44	9.08	9.73
Hungary	4.47	12.81	10.44	6.94	4.05	0.33	2.50	5.17	1.83	7.80	8.13
Macedonia	2.05	2.13	7.05	5.59	2.05	0.11	1.39	2.49	0.73	5.16	7.21
Montenegro	2.75	5.67	6.80	8.57	3.26	0.18	3.66	3.22	0.89	6.54	3.23
Poland	2.15	6.72	7.87	6.06	3.81	0.27	2.29	3.31	1.26	6.36	9.19
Romania	1.55	5.60	6.81	4.52	2.36	0.27	1.69	3.02	0.91	5.57	7.01
Serbia	3.31	6.96	9.54	8.54	2.44	0.28	2.43	4.00	1.04	7.31	7.82
Slovakia	3.41	5.52	9.15	5.44	3.27	0.32	2.59	4.84	2.78	8.13	10.29
Slovenia	2.74	5.56	7.76	5.52	3.64	0.65	2.82	6.09	2.22	7.31	6.65
Belarus	0.77	2.87	6.17	3.47	3.01	0.20	2.48	2.50	1.02	8.47	9.11
Estonia	1.28	4.15	7.52	5.29	3.87	0.18	2.94	4.09	1.77	8.50	7.85
Latvia	1.22	3.27	7.54	5.06	3.46	0.27	2.36	3.18	1.74	8.28	7.25

Lithuania	1.03	3.88	7.35	5.54	4.00	0.40	3.00	3.18	1.94	9.35	9.05
Moldova	0.44	5.84	5.99	4.04	2.29	0.17	2.34	3.40	0.96	7.14	6.53
Russian Federation	0.79	9.77	6.55	4.60	3.06	0.37	3.87	3.84	1.28	6.05	9.35
Ukraine	0.69	7.24	6.10	4.75	2.30	0.29	1.98	2.75	0.94	9.17	11.39
Brunei	0.97	5.12	6.92	4.05	3.62	0.46	0.67	7.21	1.94	6.50	5.29
Japan	1.81	3.46	6.25	3.53	3.63	0.37	0.48	5.60	1.85	6.10	7.68
South Korea	0.12	2.13	5.20	3.11	1.49	0.17	0.16	2.33	0.92	5.94	4.92
Singapore	0.39	2.59	4.18	1.85	2.77	0.57	0.42	3.38	1.06	4.82	3.89
Australia	2.98	6.63	10.45	6.80	2.68	2.39	2.27	12.42	3.80	14.07	12.45
New Zealand	3.94	8.03	10.55	6.51	3.53	1.57	2.89	11.98	4.95	10.00	12.28
Andorra	4.19	5.76	16.30	10.49	3.16	2.44	3.06	10.62	3.81	9.72	12.11
Austria	2.61	9.45	12.49	5.87	5.44	0.89	3.00	6.39	2.41	7.81	9.46
Belgium	2.07	7.03	16.36	9.97	4.67	1.36	2.81	7.38	2.96	10.72	10.53
Cyprus	0.99	2.15	10.29	4.88	2.90	1.27	1.44	6.90	2.58	7.81	5.64
Denmark	5.44	5.35	19.89	13.82	2.71	1.31	2.53	9.03	3.17	16.08	12.08
Finland	1.84	8.25	9.16	11.30	3.14	1.15	2.49	10.04	3.57	11.03	11.79
France	2.98	7.17	14.31	5.56	3.69	1.31	2.28	7.67	2.94	9.66	13.09
Germany	5.05	10.45	12.25	5.54	4.13	1.16	3.78	7.48	3.12	10.85	6.91
Greece	2.54	4.56	14.71	9.73	2.39	0.40	3.76	4.21	1.85	8.74	8.68
Iceland	2.46	9.94	13.76	15.67	11.30	0.84	3.30	8.19	3.97	11.72	13.95
Ireland	2.41	6.09	9.67	7.28	2.94	1.04	2.16	7.72	3.61	8.57	7.05
Israel	0.61	5.40	9.02	4.39	2.70	0.67	1.61	7.02	2.68	9.34	4.62
Italy	2.66	8.56	19.72	6.90	7.13	1.55	3.36	9.42	3.27	9.77	9.97
Luxembourg	4.51	4.38	14.26	10.23	6.16	1.16	2.62	7.70	3.00	11.77	11.56
Malta	2.35	5.53	13.50	5.02	3.64	1.07	2.33	5.89	2.03	6.90	7.23
Netherlands	3.42	7.28	15.94	7.86	3.19	2.33	3.42	10.96	3.81	9.40	13.58
Norway	5.23	7.46	14.52	14.11	3.75	1.07	2.83	9.63	4.73	9.14	14.19
Portugal	0.95	6.01	10.13	5.12	3.79	0.35	1.60	5.10	1.73	7.29	7.39
Spain	1.32	8.72	16.60	5.33	2.64	0.71	2.66	7.17	2.85	10.11	8.69
Sweden	3.17	7.27	11.06	13.07	2.71	1.21	1.88	9.12	3.44	7.39	15.50
Switzerland	5.09	5.15	10.24	7.74	4.88	1.39	3.34	11.82	3.49	12.48	10.92
United Kingdom	5.52	7.13	14.32	7.09	2.34	2.78	3.86	10.58	4.05	13.83	14.17
Argentina	1.70	12.99	7.74	3.12	2.42	0.29	0.97	4.13	1.62	6.30	8.41
Chile	3.84	9.10	4.94	2.00	2.32	0.42	0.87	4.02	1.94	5.48	7.31
Uruguay	2.39	14.18	10.15	3.45	1.40	0.25	1.18	5.60	2.15	7.73	8.06
Canada	2.63	5.33	12.53	8.32	3.63	1.20	3.32	11.92	3.89	9.87	9.53
United States	2.10	12.43	9.68	5.77	3.69	0.74	3.38	14.63	3.99	8.01	11.36
Antigua and Barbuda	0.05	5.85	4.10	0.68	2.30	0.17	0.28	4.22	2.39	5.91	6.75
The Bahamas	0.02	7.25	3.32	0.87	2.52	0.32	0.72	5.05	4.20	6.12	7.75
Barbados	0.03	9.22	4.18	0.61	2.51	0.27	0.80	6.92	4.35	6.94	8.83
Belize	0.03	4.42	2.54	0.67	0.83	0.47	0.71	1.99	0.88	4.43	5.13
Cuba	0.10	6.66	7.00	0.92	2.28	0.19	0.28	5.78	2.49	6.78	9.01
Dominica	0.02	6.45	4.45	0.53	1.58	0.26	0.91	6.50	3.59	7.05	9.35
Dominican Republic	0.03	3.89	1.68	0.99	1.10	0.11	0.16	1.73	1.02	4.91	6.27
Grenada	0.04	5.05	3.97	0.70	3.54	0.63	0.66	11.07	2.80	7.88	11.37
Guyana	0.03	5.46	2.09	0.56	1.17	0.44	0.27	2.37	0.93	4.54	6.59
Haiti	0.05	6.67	3.71	1.82	1.34	0.62	1.47	5.39	1.76	9.21	8.77
Jamaica	0.03	4.62	4.22	0.64	1.22	0.21	0.44	4.18	1.87	4.16	6.02
Saint Lucia	0.07	6.07	5.55	0.62	2.91	0.31	0.81	6.72	3.47	6.91	8.12
Saint Vincent and the Grenadines	0.03	6.25	3.55	0.66	2.72	0.24	0.41	6.95	1.87	6.69	8.49
Suriname	0.05	5.72	2.42	0.87	1.48	0.25	0.74	3.97	1.82	5.21	8.71
Trinidad and Tobago	0.04	8.81	3.59	0.61	1.92	0.19	0.38	4.89	2.19	6.50	8.20
Bolivia	0.23	4.51	3.00	2.34	2.35	0.84	1.41	5.31	1.67	7.86	7.29
Ecuador	0.09	3.84	2.08	0.96	1.27	0.20	0.88	3.05	1.43	5.40	6.85
Peru	0.11	5.21	2.27	1.49	1.67	1.01	0.49	3.83	1.66	5.73	6.39

Colombia	0.56	2.76	3.37	2.50	2.65	0.29	0.91	3.60	1.52	7.48	4.26
Costa Rica	0.36	2.87	4.17	1.69	3.16	0.19	2.06	5.30	2.19	7.75	5.10
El Salvador	0.04	2.63	1.67	1.30	2.61	0.17	1.19	1.63	1.05	7.44	4.44
Guatemala	0.02	3.21	1.58	0.67	1.19	0.18	0.84	1.36	0.76	4.46	4.74
Honduras	0.05	2.28	1.77	1.93	1.31	0.24	0.26	1.96	1.51	11.33	5.15
Mexico	0.89	4.95	2.51	1.94	1.83	0.33	1.06	2.53	1.07	5.84	4.26
Nicaragua	0.04	3.26	1.55	1.12	1.04	0.08	0.48	1.84	0.71	5.10	4.57
Panama	0.09	2.32	2.55	1.88	1.88	0.17	0.37	3.09	1.63	6.03	6.16
Venezuela	0.06	7.27	3.22	0.64	1.39	0.19	0.88	3.89	1.69	6.22	7.82
Brazil	0.43	3.69	4.28	2.87	1.66	0.56	0.75	3.07	1.16	5.50	6.20
Paraguay	0.10	4.15	1.96	1.17	1.41	0.18	0.57	2.13	0.78	5.50	5.94
Algeria	0.22	1.32	3.21	1.90	1.98	0.07	1.76	3.64	1.04	4.07	5.02
Bahrain	0.02	4.21	10.71	2.36	1.54	0.23	1.26	3.41	1.89	6.87	4.87
Egypt	0.03	1.90	12.07	2.88	0.88	0.11	2.24	1.83	0.68	4.96	4.81
Iran	0.23	2.35	3.86	4.35	1.34	0.30	0.59	1.91	0.80	7.90	3.43
Iraq	0.29	2.92	8.04	6.04	2.12	0.34	0.86	3.87	0.84	7.67	5.69
Jordan	0.69	1.76	5.35	2.81	2.86	0.35	0.32	5.54	1.41	8.89	5.68
Kuwait	0.51	2.44	7.63	2.78	3.36	0.14	0.69	4.75	1.15	6.49	4.63
Lebanon	0.60	2.17	19.87	4.65	3.69	0.12	3.15	8.52	2.56	10.43	9.09
Libya	0.28	2.95	8.68	4.61	2.18	0.11	2.46	6.07	1.79	7.42	6.90
Morocco	0.17	1.23	2.38	2.31	2.22	0.09	2.06	4.68	0.68	2.77	5.77
Palestine	0.01	3.47	4.98	6.77	1.69	0.11	0.61	3.01	1.36	7.69	3.39
Oman	0.15	1.46	4.04	2.18	1.84	0.24	1.29	5.48	1.52	5.66	4.38
Qatar	0.03	5.79	7.53	3.84	3.61	0.09	0.32	4.50	1.09	8.89	4.86
Saudi Arabia	0.18	1.38	3.00	2.12	1.87	0.06	0.97	4.02	0.93	4.92	4.33
Syria	0.02	1.43	2.51	3.85	0.61	0.15	0.15	2.82	0.62	17.83	2.63
Tunisia	0.27	1.77	6.62	2.42	1.82	0.06	1.50	4.48	1.29	5.34	4.89
Turkey	1.02	4.51	7.00	9.33	1.73	1.96	0.91	4.29	2.05	9.32	4.66
United Arab Emirates	0.14	3.11	6.91	4.07	1.71	0.08	0.81	4.27	1.31	6.23	40.53
Yemen	0.11	1.46	3.58	3.15	0.94	0.12	1.16	2.23	0.64	6.65	4.08
Afghanistan	0.13	3.32	4.39	6.03	1.53	0.66	2.17	3.64	1.02	10.26	5.60
Bangladesh	0.02	1.50	1.92	3.68	1.09	0.27	1.54	1.91	0.76	5.08	4.33
Bhutan	0.28	1.68	1.92	2.80	1.22	0.22	1.34	2.12	0.89	4.75	4.38
India	0.30	1.23	1.63	2.29	1.10	0.21	0.98	1.86	0.73	4.07	3.52
Nepal	0.29	1.17	1.83	2.30	1.14	0.20	1.24	1.66	0.73	4.21	3.69
Pakistan	0.59	1.29	5.34	2.74	2.76	0.13	2.29	5.46	1.52	4.77	7.18
Angola	0.19	2.92	4.48	1.81	0.75	0.28	0.90	3.06	1.17	4.60	6.42
Central African Republic	0.19	2.45	3.40	1.70	0.82	0.34	0.94	2.73	1.15	4.38	6.35
Congo	0.19	3.13	5.22	1.98	0.94	0.49	0.82	3.25	1.29	4.54	6.90
Democratic Republic of the Congo	0.13	2.13	3.72	1.48	0.67	0.20	0.67	2.35	1.03	3.87	5.63
Equatorial Guinea	0.20	2.94	3.14	1.71	0.82	0.35	0.98	2.93	1.24	4.42	6.11
Gabon	0.15	2.88	5.74	1.66	0.89	0.31	0.60	3.28	1.47	3.79	6.00
Burundi	0.14	2.31	3.91	2.24	1.38	0.42	1.56	7.56	1.77	4.02	11.86
Comoros	0.12	2.20	3.60	2.08	1.21	0.22	1.49	6.79	1.75	4.42	13.19
Djibouti	0.14	2.23	4.13	2.08	1.12	0.17	1.18	6.67	1.83	3.99	12.28
Eritrea	0.18	2.42	4.36	2.43	1.22	0.39	2.06	7.56	1.62	5.13	15.10
Ethiopia	0.05	3.24	2.94	4.08	3.71	0.41	2.94	7.98	1.14	7.44	7.35
Kenya	0.08	1.18	1.32	1.63	0.50	0.15	0.58	2.95	1.58	2.75	8.11
Madagascar	0.12	1.79	3.79	1.97	1.10	0.16	1.39	5.91	1.44	3.88	11.46
Malawi	0.23	2.64	9.08	1.13	1.13	0.15	0.72	13.24	1.00	2.54	14.52
Mauritius	0.19	2.65	6.15	1.43	1.52	0.15	0.53	1.56	0.68	5.32	4.27
Mozambique	0.02	1.88	4.17	2.98	1.08	0.20	1.78	3.99	1.65	6.01	4.62
Rwanda	0.10	2.33	3.66	2.29	1.36	0.33	1.53	7.80	1.75	4.22	12.19
Seychelles	0.74	3.00	8.98	4.94	1.22	0.30	0.76	3.75	1.10	6.59	6.47
Somalia	0.11	2.26	3.42	1.78	0.98	0.25	1.47	6.19	1.55	4.02	11.09

Tanzania	0.01	2.07	3.52	2.31	1.18	0.17	1.07	6.22	1.75	3.70	10.96
Uganda	0.08	1.47	2.48	1.13	1.07	0.14	1.11	8.37	1.68	2.62	16.06
Zambia	0.07	2.96	5.12	3.46	1.55	0.25	1.48	8.44	2.05	4.86	14.54
Botswana	0.18	1.95	3.48	1.34	0.85	0.74	0.48	2.54	1.45	3.97	6.11
Lesotho	0.22	1.83	2.67	1.29	0.82	1.07	0.59	2.39	1.43	4.06	6.04
Namibia	0.34	1.88	2.52	1.77	0.86	1.11	0.99	4.42	1.24	3.33	10.27
South Africa	0.23	2.95	4.08	1.54	1.02	0.74	0.39	2.21	1.55	4.62	3.77
Swaziland	0.18	2.82	3.81	1.56	1.08	0.94	0.61	3.20	1.84	4.45	6.52
Zimbabwe	0.14	1.54	8.63	1.54	1.54	0.11	0.47	4.68	2.29	3.32	8.12
Benin	0.14	2.08	4.66	1.04	0.55	0.24	0.60	3.16	0.94	3.75	5.83
Burkina Faso	0.17	1.87	4.93	3.08	0.67	0.36	0.68	3.32	0.99	3.98	5.95
Cameroon	0.14	3.01	4.82	1.24	0.78	0.38	0.57	4.22	1.31	3.86	6.11
Cape Verde	0.13	3.03	2.66	2.49	0.51	0.08	0.13	2.10	0.75	2.92	3.84
Chad	0.13	1.51	3.41	0.85	0.50	0.26	0.55	2.62	0.84	3.28	4.92
Cote d'Ivoire	0.12	1.45	3.30	0.75	0.60	0.34	0.63	4.90	1.58	2.82	3.52
The Gambia	0.21	1.65	2.04	0.59	0.55	0.24	0.62	4.09	0.68	3.02	4.61
Ghana	0.01	3.29	5.47	2.56	0.26	0.41	0.17	6.58	0.95	4.78	4.56
Guinea	0.25	1.98	4.88	0.89	0.67	0.33	0.92	2.86	0.73	3.34	6.43
Guinea-Bissau	0.20	2.67	4.85	1.25	0.72	0.59	0.88	4.03	1.11	4.31	6.17
Liberia	0.12	2.52	4.85	1.08	0.62	0.23	0.58	3.57	0.99	3.69	5.74
Mali	0.23	1.87	10.82	0.64	1.11	0.37	1.22	2.66	0.85	3.45	6.88
Mauritania	0.21	2.45	5.14	1.12	0.67	0.42	0.55	3.36	1.14	3.79	5.63
Niger	0.19	1.95	3.26	1.04	0.50	0.24	0.71	3.14	0.83	3.86	5.88
Nigeria	0.10	2.19	1.56	1.15	0.37	0.19	3.03	3.33	1.36	3.13	6.69
Sao Tome and Principe	0.09	2.47	4.71	0.64	0.55	0.15	0.10	2.97	0.74	3.29	14.95
Senegal	0.18	2.15	4.79	1.04	0.55	0.27	0.57	3.16	0.95	3.74	5.82
Sierra Leone	0.14	1.93	4.74	1.06	0.54	0.19	0.56	3.24	0.85	3.57	5.75
Togo	0.16	1.85	4.62	1.07	0.59	0.32	0.55	2.99	0.91	3.62	5.62
American Samoa	0.04	2.74	3.34	1.68	2.46	0.33	0.21	2.29	1.69	3.62	6.42
Bermuda	0.03	11.10	10.00	0.97	3.62	1.16	0.81	7.98	3.79	9.81	7.91
Greenland	0.20	11.58	8.18	2.75	1.40	1.29	0.87	6.12	1.70	6.29	6.14
Guam	0.36	4.87	4.00	1.56	1.60	0.20	0.37	4.93	1.78	5.95	5.13
Northern Mariana Islands	0.16	4.30	3.22	2.09	2.06	0.13	0.23	2.57	2.66	5.77	4.68
Puerto Rico	0.20	4.73	4.60	0.99	3.18	0.25	1.43	5.96	2.83	6.68	7.76
Virgin Islands, U.S.	0.01	8.00	3.55	1.52	1.41	0.61	0.28	5.65	4.43	6.96	6.29
South Sudan	0.10	2.35	3.21	1.62	1.09	0.12	1.13	6.57	1.68	3.47	10.29
Sudan	0.10	1.96	5.11	3.56	1.03	0.08	1.09	2.46	0.71	6.91	4.06

Age-standardized Incidence in 2017

	Testicular cancer	Kidney cancer	Bladder cancer	Brain and nervous system cancer	Thyroid cancer	Mesothelioma	Hodgkin lymphoma	Non-Hodgkin lymphoma	Multiple myeloma	Leukemia	Other malignant neoplasms
China	0.35	2.69	3.89	7.87	2.17	0.15	1.19	4.47	0.99	10.63	13.68
North Korea	0.26	3.72	3.70	4.09	1.99	0.23	0.49	2.73	0.62	6.47	4.60
China Taiwan	1.42	5.86	8.07	7.06	5.48	0.15	1.13	9.70	1.83	7.48	16.14
Cambodia	0.11	3.14	4.00	2.66	2.18	0.29	0.61	3.22	0.85	6.57	4.60
Indonesia	0.01	3.18	4.52	3.27	2.02	0.24	0.50	3.23	0.75	6.06	4.51
Laos	0.09	3.65	4.10	3.02	1.91	0.24	0.65	3.41	0.79	7.15	4.72
Malaysia	1.06	3.26	5.35	2.69	4.98	0.21	0.77	5.13	1.06	6.57	6.86
Maldives	0.04	2.47	5.52	2.10	4.35	0.49	0.24	3.00	1.02	4.60	4.52
Myanmar	0.12	4.40	4.81	3.51	2.58	0.60	0.69	3.85	0.99	8.13	5.69
Philippines	0.34	4.37	2.12	2.97	5.66	0.23	0.28	3.21	0.73	6.60	5.93
Sri Lanka	0.18	6.27	3.16	2.72	3.63	0.40	0.94	3.51	1.10	6.75	8.55
Thailand	0.57	2.59	3.93	4.15	2.85	0.33	0.55	2.68	0.79	7.50	7.10
Timor-Leste	0.12	2.95	4.35	2.73	1.93	0.21	0.61	3.24	0.73	7.32	5.12
Vietnam	0.51	1.98	2.55	3.60	6.67	0.22	0.66	4.45	0.71	4.34	7.44
Fiji	1.04	2.52	3.37	1.69	4.04	0.35	0.36	2.62	1.29	5.56	5.67
Kiribati	1.95	4.32	2.18	1.19	0.68	0.21	0.25	2.13	0.98	4.68	6.39
Marshall Islands	0.51	4.74	4.35	2.41	2.93	0.43	0.45	3.89	1.75	6.32	6.34
Federated States of Micronesia	0.38	3.59	3.93	1.99	2.12	0.46	0.38	3.04	1.46	5.67	5.54
Papua New Guinea	0.47	2.71	3.07	2.17	1.78	0.41	0.58	2.91	0.98	6.50	6.02
Samoa	0.75	2.13	2.65	1.62	3.14	0.23	0.51	4.79	1.47	4.75	5.36
Solomon Islands	0.31	2.86	2.88	1.66	1.57	0.38	0.44	2.52	1.12	5.39	5.15
Tonga	12.92	4.14	3.55	2.01	1.99	0.23	0.27	5.86	1.82	4.14	4.56
Vanuatu	0.35	4.03	5.10	2.47	1.93	0.51	0.71	3.80	1.10	9.13	8.94
Armenia	1.16	6.10	9.80	7.27	2.78	0.69	0.64	2.95	0.80	5.10	10.48
Azerbaijan	0.26	8.30	5.22	5.24	2.54	0.14	1.56	3.02	0.80	7.05	7.12
Georgia	2.92	6.93	8.09	5.82	3.41	0.54	2.18	4.49	0.99	6.95	8.10
Kazakhstan	0.91	7.75	4.59	3.83	3.58	0.33	1.39	2.51	0.76	4.48	7.33
Kyrgyzstan	0.42	4.55	2.71	2.79	1.78	0.14	0.44	1.68	0.51	3.22	4.78
Mongolia	0.02	5.03	1.93	3.15	1.52	0.17	0.23	2.49	0.54	3.17	7.05
Tajikistan	0.01	5.59	2.21	6.44	0.51	0.29	0.30	2.96	0.63	3.92	5.68
Turkmenistan	0.90	8.12	2.91	4.71	1.80	0.18	1.12	2.12	1.20	4.34	6.22
Uzbekistan	0.52	4.35	3.23	5.04	0.89	0.13	1.03	3.37	0.64	4.49	4.86
Albania	2.84	4.89	2.25	9.50	3.00	0.17	2.13	3.15	0.87	6.06	13.85
Bosnia and Herzegovina	1.85	7.61	8.08	8.67	2.95	0.19	1.29	4.23	1.06	5.18	6.84
Bulgaria	3.23	5.58	9.98	8.76	3.06	0.12	3.38	6.48	1.23	6.62	9.27
Croatia	4.96	12.26	13.10	15.12	3.67	1.00	3.32	9.66	3.06	8.28	9.97
Czech Republic	5.42	13.11	13.02	7.20	4.07	0.33	4.38	9.39	2.74	6.85	15.41
Hungary	4.62	9.79	11.63	6.97	3.42	0.29	1.99	7.21	1.99	6.46	7.14
Macedonia	3.13	4.45	10.09	8.37	2.63	0.08	2.19	3.75	0.88	5.49	8.08
Montenegro	3.16	5.65	8.18	10.31	4.54	0.21	4.71	4.68	1.01	5.97	4.72
Poland	4.14	8.11	11.43	8.14	4.25	0.59	2.86	6.81	2.25	5.72	11.07
Romania	3.22	7.09	9.94	7.99	3.85	0.30	2.31	6.02	1.38	5.79	10.94
Serbia	4.72	7.88	12.31	9.89	3.33	0.34	2.85	6.32	1.51	6.57	12.42
Slovakia	6.32	14.14	10.61	8.23	3.27	0.24	4.17	10.83	4.17	11.11	9.02
Slovenia	4.40	8.06	7.85	7.25	3.70	0.94	3.37	14.16	2.91	6.36	11.49
Belarus	1.06	9.80	6.25	7.16	4.51	0.26	4.02	5.73	2.20	7.42	14.75
Estonia	1.34	12.08	9.09	10.19	5.38	0.26	4.33	9.50	3.49	10.32	10.61

Latvia	1.64	10.33	10.71	9.62	4.87	0.33	3.98	7.27	3.18	9.29	9.33
Lithuania	1.27	11.12	8.22	9.16	4.66	0.26	3.36	7.50	3.00	9.95	14.33
Moldova	0.65	6.85	5.96	5.15	2.85	0.16	2.23	4.57	1.04	4.48	7.04
Russian Federation	0.97	10.24	7.43	5.78	5.33	0.38	3.90	6.20	2.26	6.30	19.74
Ukraine	1.83	9.54	7.75	10.50	4.34	0.43	4.47	5.98	1.51	8.57	20.85
Brunei	1.22	8.10	7.01	6.37	6.20	0.44	1.72	10.78	3.54	7.33	7.94
Japan	2.29	4.44	6.56	5.14	4.72	0.46	0.92	9.30	2.19	5.32	11.92
South Korea	0.39	4.32	5.88	6.86	12.87	0.16	0.70	6.28	1.88	5.07	9.51
Singapore	0.35	3.26	3.50	5.51	3.76	0.31	0.99	5.57	1.20	3.75	8.37
Australia	3.39	8.75	8.33	7.42	5.38	2.30	3.30	15.95	5.26	9.79	14.70
New Zealand	3.86	9.00	7.82	7.46	4.83	1.25	3.06	15.73	5.83	8.67	16.63
Andorra	3.78	6.70	14.85	13.61	4.03	2.25	3.81	14.63	4.12	8.66	15.50
Austria	4.38	7.03	11.32	9.02	4.56	0.79	2.21	10.84	3.40	9.65	11.22
Belgium	1.99	7.43	13.87	11.60	4.39	1.34	3.16	11.65	3.79	8.64	14.62
Cyprus	0.36	4.16	12.74	9.23	3.70	0.81	2.75	10.98	3.42	8.63	12.05
Denmark	4.46	8.46	16.09	19.04	3.84	1.36	3.30	13.54	4.42	11.98	16.01
Finland	2.99	9.38	7.47	14.19	4.24	0.95	3.90	15.11	4.20	9.06	15.97
France	3.81	7.84	13.91	10.70	3.87	1.50	3.27	10.97	3.52	7.98	15.66
Germany	6.02	10.53	10.46	7.99	5.20	1.08	3.40	12.99	4.22	7.36	15.10
Greece	4.14	6.82	16.15	15.44	3.38	0.40	6.68	7.00	2.96	8.85	10.67
Iceland	3.04	12.54	12.15	19.67	11.57	1.01	2.86	12.93	5.24	8.84	17.55
Ireland	2.98	8.15	9.86	13.62	3.99	0.66	4.01	14.67	4.46	8.58	14.01
Israel	1.26	6.33	9.99	8.59	4.52	0.45	2.38	11.55	3.57	7.41	7.93
Italy	4.33	8.87	15.89	9.95	6.79	1.27	4.25	13.46	4.35	8.33	14.69
Luxembourg	3.89	4.44	13.61	18.09	6.69	1.03	3.36	14.52	4.10	10.38	16.91
Malta	5.00	7.09	12.34	10.44	5.15	1.19	4.45	12.02	2.96	6.65	12.82
Netherlands	4.55	9.14	15.61	13.42	4.74	2.00	3.92	15.20	4.53	8.95	17.94
Norway	4.52	11.34	12.74	15.82	5.18	0.91	3.09	14.25	6.02	9.19	13.97
Portugal	1.44	6.59	12.14	8.83	4.23	0.37	2.41	10.94	2.40	5.59	11.08
Spain	1.69	11.64	17.05	9.86	3.59	0.63	3.39	11.47	3.58	9.67	11.62
Sweden	3.22	7.60	11.48	12.85	3.57	0.87	2.31	11.35	4.07	7.68	13.70
Switzerland	2.81	6.01	8.85	10.43	4.34	1.23	2.69	11.79	3.98	10.07	12.52
United Kingdom	3.74	9.50	9.81	11.20	3.44	2.52	4.11	15.20	5.14	12.13	16.04
Argentina	3.75	12.00	6.54	3.67	2.63	0.50	0.88	4.97	1.79	5.26	5.48
Chile	8.50	10.02	6.27	3.48	4.06	0.32	1.08	6.16	3.07	5.08	6.69
Uruguay	3.35	15.79	9.76	4.21	3.03	0.30	1.21	8.01	3.09	6.57	5.76
Canada	3.53	7.21	11.19	11.27	4.72	0.95	4.01	15.64	4.38	8.08	13.44
United States	3.16	12.75	9.91	7.16	5.54	0.58	3.15	15.21	4.76	7.06	10.91
Antigua and Barbuda	0.64	4.90	4.69	2.94	3.79	0.11	0.37	4.97	2.82	5.73	5.62
The Bahamas	0.09	6.21	3.77	2.67	3.75	0.29	0.77	5.67	4.76	5.65	6.46
Barbados	0.33	7.00	4.69	2.86	3.86	0.23	0.92	8.12	5.21	6.50	7.39
Belize	0.29	5.43	3.31	2.48	1.55	0.62	0.67	2.53	1.22	4.34	4.66
Cuba	1.08	5.11	8.33	5.37	3.86	0.15	2.07	6.47	2.73	5.10	7.93
Dominica	0.17	7.04	5.82	2.05	2.70	0.37	0.93	8.59	4.69	8.37	8.63
Dominican Republic	0.06	2.94	2.27	2.50	2.24	0.12	0.38	2.61	1.64	5.09	5.40
Grenada	0.10	4.26	4.44	2.67	3.23	0.32	0.38	8.59	3.51	6.45	6.45
Guyana	0.26	5.12	2.41	1.45	2.01	0.41	0.51	2.55	1.24	4.38	5.10
Haiti	0.10	4.99	4.04	2.31	1.58	0.43	1.08	4.59	1.90	7.66	7.07
Jamaica	0.34	3.84	4.33	1.99	3.61	0.22	0.50	7.21	3.60	6.78	5.52
Saint Lucia	0.96	4.53	5.75	2.30	3.89	0.23	0.85	6.42	3.79	5.47	5.49
Saint Vincent and the Grenadines	0.28	5.33	4.28	2.41	3.89	0.22	0.86	8.18	2.32	6.63	6.77
Suriname	0.47	5.00	2.97	4.27	2.41	0.23	0.77	4.23	2.26	4.63	6.50
Trinidad and Tobago	0.35	5.56	3.47	1.85	2.61	0.18	0.61	4.87	3.00	5.12	5.56
Bolivia	0.50	5.64	3.57	3.02	3.79	0.49	0.73	5.91	1.78	6.90	6.10
Ecuador	1.22	4.91	2.33	3.51	5.29	0.23	0.81	5.29	1.66	6.83	5.23

Peru	1.05	4.95	2.37	3.06	3.67	0.23	0.37	4.78	1.64	5.31	4.24
Colombia	1.73	3.58	2.60	3.23	3.57	0.24	0.79	4.16	1.62	5.71	4.77
Costa Rica	2.94	4.96	4.72	3.83	4.19	0.17	2.10	7.26	2.85	7.34	5.83
El Salvador	0.46	3.63	2.44	3.42	2.77	0.18	0.82	3.15	1.39	7.34	3.60
Guatemala	0.70	3.68	1.53	2.11	1.95	0.15	0.48	2.12	0.84	6.05	3.21
Honduras	0.06	3.43	2.50	2.33	1.83	0.34	0.18	2.67	2.07	11.89	4.54
Mexico	3.46	6.89	2.79	2.71	3.78	0.40	0.95	3.82	1.46	5.70	4.71
Nicaragua	0.63	3.23	1.70	1.92	2.36	0.11	0.39	2.35	0.71	4.37	3.53
Panama	1.04	4.35	2.52	3.62	3.46	0.17	0.61	3.57	1.68	5.07	5.99
Venezuela	1.45	6.99	3.65	2.48	3.02	0.17	1.13	4.12	1.85	5.30	5.51
Brazil	1.08	5.06	4.46	5.28	2.27	0.46	0.65	3.79	1.74	4.64	5.68
Paraguay	0.97	4.47	2.47	1.83	2.74	0.25	0.56	3.15	0.98	5.20	5.46
Algeria	0.32	1.91	3.82	2.36	4.40	0.15	1.72	4.59	1.47	4.03	5.03
Bahrain	0.04	3.23	6.33	2.16	2.07	0.38	0.66	3.59	1.59	4.43	3.49
Egypt	0.07	2.72	15.40	3.65	2.03	0.26	1.89	2.54	0.94	4.80	4.82
Iran	0.78	3.19	6.02	6.57	3.16	0.81	1.34	3.54	1.30	8.08	5.61
Iraq	0.16	2.82	5.90	4.77	1.66	0.19	0.28	1.49	0.68	4.84	3.24
Jordan	0.74	2.63	6.91	3.50	2.81	0.23	0.26	5.71	1.25	7.71	4.64
Kuwait	0.13	2.58	6.26	3.67	2.78	0.25	0.65	5.35	1.03	4.25	4.58
Lebanon	3.33	3.37	25.14	9.62	9.80	0.13	10.40	23.35	3.67	11.64	21.75
Libya	0.81	4.52	12.21	7.86	5.42	0.34	4.27	9.06	2.30	8.99	9.09
Morocco	0.31	1.85	3.29	2.86	4.13	0.20	1.83	5.93	0.94	2.74	5.74
Palestine	0.03	4.18	5.18	6.74	1.74	0.08	0.63	2.69	1.40	6.71	3.08
Oman	0.26	2.65	4.94	3.28	3.56	0.40	1.61	7.64	2.20	5.49	5.92
Qatar	0.20	3.23	7.52	4.85	2.18	0.18	0.72	5.21	1.43	6.82	6.34
Saudi Arabia	0.75	3.22	5.10	4.26	7.16	0.10	2.08	8.58	1.73	5.96	6.63
Syria	0.05	2.11	4.16	5.16	1.05	0.16	0.17	2.37	0.82	14.83	2.82
Tunisia	0.61	2.22	9.06	3.34	3.75	0.14	1.96	5.92	1.65	4.78	5.18
Turkey	2.60	4.54	8.43	8.17	3.28	0.92	1.08	4.97	2.39	6.88	4.87
United Arab Emirates	0.63	5.42	11.10	8.38	3.09	0.27	1.15	5.50	1.76	6.55	49.28
Yemen	0.16	2.08	4.99	3.37	1.47	0.24	0.85	2.26	0.75	7.04	4.62
Afghanistan	0.18	3.66	4.82	5.64	1.88	0.82	1.76	3.29	1.00	10.66	6.16
Bangladesh	0.07	1.47	1.70	3.41	1.81	0.12	0.50	1.71	0.80	3.44	3.65
Bhutan	0.22	2.05	2.38	2.57	1.88	0.19	0.53	2.41	1.09	3.82	4.53
India	0.27	1.86	2.15	2.44	1.99	0.27	0.52	2.61	0.97	3.76	4.48
Nepal	0.14	1.76	2.07	2.00	1.71	0.19	0.55	2.05	0.98	3.64	4.06
Pakistan	0.66	2.25	7.46	3.72	4.91	0.19	1.96	7.13	1.89	5.73	8.22
Angola	0.15	3.46	4.53	1.78	0.85	0.21	0.52	2.59	1.32	3.93	5.45
Central African Republic	0.20	2.92	3.21	1.97	0.67	0.29	0.85	2.26	1.05	4.46	5.83
Congo	0.14	3.94	6.10	2.42	0.99	0.35	0.56	2.85	1.29	4.38	6.51
Democratic Republic of the Congo	0.14	2.23	3.31	1.59	0.67	0.18	0.54	1.92	0.98	3.79	5.08
Equatorial Guinea	0.14	4.47	4.10	1.81	1.32	0.16	0.32	3.12	1.78	3.19	4.76
Gabon	0.16	4.22	7.02	2.44	0.94	0.25	0.44	2.97	1.57	3.85	5.66
Burundi	0.08	2.07	2.60	1.86	1.28	0.17	0.93	5.04	1.49	3.49	8.55
Comoros	0.08	2.44	3.52	2.27	1.49	0.17	0.95	5.39	1.70	4.23	10.79
Djibouti	0.10	3.18	4.79	2.51	1.52	0.17	0.88	6.18	1.92	4.18	10.50
Eritrea	0.13	3.30	4.64	3.27	1.78	0.31	1.55	7.32	1.60	5.97	14.95
Ethiopia	0.06	2.70	3.19	2.99	3.32	0.16	1.45	5.81	1.12	6.37	5.76
Kenya	0.12	1.53	1.61	1.80	0.70	0.16	0.59	3.62	1.98	3.05	9.31
Madagascar	0.07	1.87	2.65	1.77	1.23	0.13	0.88	4.44	1.26	3.45	8.81
Malawi	0.30	2.84	9.19	1.23	1.47	0.14	0.54	13.09	1.17	2.56	16.03
Mauritius	0.43	3.61	4.69	2.41	2.53	0.11	0.86	3.06	1.23	5.22	7.73
Mozambique	0.02	2.39	3.39	3.37	1.58	0.18	1.18	4.24	1.80	5.32	4.20
Rwanda	0.06	2.28	2.78	2.03	1.44	0.15	0.77	5.36	1.70	3.83	9.47
Seychelles	1.34	5.39	10.72	5.30	1.55	0.24	0.96	7.48	2.29	8.28	4.81

Somalia	0.09	2.68	3.48	2.09	1.22	0.22	1.48	5.80	1.48	4.94	11.28
Tanzania	0.01	2.90	3.22	2.63	1.51	0.15	0.86	5.94	1.79	4.22	10.12
Uganda	0.13	2.23	2.94	1.60	1.68	0.12	0.98	8.77	1.98	2.82	18.14
Zambia	0.06	2.99	4.59	2.98	1.40	0.24	1.02	6.29	1.84	4.33	10.67
Botswana	0.14	2.85	3.72	1.66	1.27	0.59	0.30	2.87	1.73	3.51	5.42
Lesotho	0.29	3.56	3.37	2.09	1.12	1.42	0.68	3.18	1.57	4.85	7.21
Namibia	0.32	2.37	2.54	1.81	1.19	0.81	0.62	4.76	1.27	2.83	11.75
South Africa	0.24	3.49	4.02	1.78	1.02	0.77	0.32	2.41	1.77	4.07	3.24
Swaziland	0.22	4.27	3.97	2.14	1.28	0.87	0.56	3.38	1.80	4.44	6.85
Zimbabwe	0.20	2.18	8.77	2.35	1.93	0.21	0.70	7.52	3.28	4.06	11.57
Benin	0.07	3.76	3.47	1.49	0.57	0.21	0.41	3.11	1.35	3.75	5.10
Burkina Faso	0.12	3.02	3.08	3.22	0.60	0.21	0.45	3.12	1.30	3.82	4.99
Cameroon	0.07	4.62	4.25	1.98	0.69	0.30	0.43	3.89	1.61	3.95	5.43
Cape Verde	0.11	5.60	4.46	3.27	0.86	0.09	0.12	3.24	1.34	3.97	4.49
Chad	0.09	2.68	3.53	1.36	0.49	0.19	0.49	2.89	1.09	3.78	4.95
Cote d'Ivoire	0.11	2.27	3.93	0.97	0.68	0.29	0.51	5.43	1.84	3.03	3.85
The Gambia	0.16	2.55	2.60	0.91	0.71	0.22	0.48	4.78	0.81	3.30	5.10
Ghana	0.02	3.14	4.31	3.19	0.33	0.23	0.14	5.08	1.47	3.56	3.67
Guinea	0.31	2.72	6.33	1.08	0.81	0.30	0.72	2.81	0.86	3.31	6.17
Guinea-Bissau	0.09	4.31	3.83	1.71	0.65	0.42	0.59	3.66	1.48	4.31	5.45
Liberia	0.06	3.23	3.27	1.23	0.54	0.18	0.36	2.77	1.25	3.34	4.42
Mali	0.18	2.48	11.61	0.75	1.29	0.16	0.66	2.56	1.12	3.19	6.55
Mauritania	0.08	3.80	4.58	1.62	0.71	0.21	0.34	3.29	1.52	3.91	5.20
Niger	0.07	2.30	2.29	1.12	0.45	0.14	0.42	2.40	1.04	3.46	4.53
Nigeria	0.05	2.72	1.75	1.76	0.47	0.11	2.16	3.46	1.47	3.18	6.97
Sao Tome and Principe	0.07	3.37	7.52	0.94	0.76	0.17	0.09	3.49	1.29	3.57	16.62
Senegal	0.08	3.32	4.31	1.54	0.54	0.25	0.44	3.09	1.30	4.11	5.47
Sierra Leone	0.07	3.01	3.53	1.52	0.60	0.24	0.41	3.10	1.19	3.62	4.97
Togo	0.10	2.76	3.37	1.48	0.54	0.24	0.39	2.65	1.21	3.45	4.65
American Samoa	0.13	2.82	5.22	2.24	3.91	0.46	0.25	3.31	2.43	3.34	8.26
Bermuda	0.14	6.23	9.68	5.26	4.47	0.47	2.63	8.54	3.54	6.92	8.49
Greenland	0.10	12.64	7.74	2.42	2.04	1.16	0.43	4.50	1.62	4.58	5.34
Guam	1.25	5.29	6.37	2.43	3.59	0.32	0.50	5.86	2.15	6.26	6.33
Northern Mariana Islands	0.59	4.29	4.65	2.78	2.97	0.13	0.36	3.94	2.98	6.21	7.52
Puerto Rico	2.48	4.30	5.11	3.66	3.08	0.22	2.60	7.79	3.46	5.58	7.93
Virgin Islands, U.S.	0.03	9.56	4.89	4.20	2.82	0.77	0.62	8.24	6.24	7.03	7.12
South Sudan	0.08	3.11	3.13	1.98	1.34	0.12	1.09	6.11	1.66	4.06	9.80
Sudan	0.13	2.55	5.23	3.33	1.53	0.16	0.73	2.44	0.86	6.56	4.11

Age-standardized Incidence change from 1990 to 2017(%)

	Testicular cancer	Kidney cancer	Bladder cancer	Brain and nervous system cancer	Thyroid cancer	Mesothelioma	Hodgkin lymphoma	Non-Hodgkin lymphoma	Multiple myeloma	Leukemia	Other malignant neoplasms
China	100.54	18.40	15.29	74.54	102.90	9.15	34.09	103.87	53.24	23.95	164.42
North Korea	7.39	15.85	8.57	13.49	11.82	66.95	1.40	9.30	5.50	7.72	-4.58
China Taiwan	372.48	136.90	38.42	77.46	101.52	-5.38	149.53	127.15	105.95	37.99	78.81
Cambodia	-16.80	13.60	20.20	-8.36	12.19	-21.86	-47.97	-1.12	14.79	-11.91	-15.69
Indonesia	-12.53	37.87	45.18	0.16	23.79	26.44	-32.47	17.54	29.11	-0.39	-4.43
Laos	-40.07	15.75	12.93	-12.39	0.54	-37.75	-49.15	-8.46	9.53	-12.09	-16.00
Malaysia	144.70	35.20	24.08	28.24	98.31	13.13	16.01	47.01	45.31	-4.54	21.81
Maldives	335.38	-22.20	12.62	-16.27	38.72	-62.40	-35.11	-9.13	19.85	-21.96	22.12
Myanmar	-24.25	20.32	11.87	-8.33	13.44	-54.67	-50.95	-1.68	16.80	-14.82	-17.26
Philippines	46.58	47.84	16.77	7.87	97.74	0.99	-32.37	45.47	22.40	34.66	-44.14
Sri Lanka	234.56	-24.89	44.02	85.74	57.84	-13.33	-4.64	77.12	40.22	0.18	72.16
Thailand	920.89	17.43	-6.21	59.09	41.16	-42.45	-3.36	-5.28	16.57	-2.47	50.04
Timor-Leste	4.41	26.60	60.01	4.85	37.28	10.06	-37.15	16.43	20.32	5.63	0.96
Vietnam	99.76	12.64	30.21	24.07	146.74	3.24	-5.08	13.02	24.12	9.62	10.14
Fiji	11.50	16.12	40.05	10.79	12.98	44.70	22.58	48.41	28.01	-5.01	-1.33
Kiribati	12.33	33.85	17.60	-2.41	11.42	36.57	-2.74	12.25	6.05	-2.64	14.04
Marshall Islands	5.11	28.85	50.56	32.59	61.97	38.19	-10.90	23.66	18.75	3.54	5.58
Federated States of Micronesia	17.29	12.03	23.82	7.67	43.43	18.86	-26.18	6.98	16.66	-11.21	-9.72
Papua New Guinea	-1.37	6.32	29.95	8.32	30.55	11.35	-18.56	6.16	11.60	-5.77	-2.31
Samoa	-21.27	2.59	6.09	-9.80	35.63	4.78	-25.79	5.80	8.08	-11.91	-8.18
Solomon Islands	-14.85	11.77	21.82	6.28	25.04	8.52	-19.28	3.45	12.26	-7.76	-7.10
Tonga	28.52	35.54	32.39	25.68	60.20	18.04	2.06	18.38	9.17	4.48	7.15
Vanuatu	-1.46	26.04	40.38	23.05	32.03	36.53	-1.04	16.17	10.64	7.97	9.23
Armenia	14.96	284.16	-5.30	36.08	293.26	-7.78	184.52	63.08	43.93	-17.35	-26.87
Azerbaijan	55.09	-1.64	26.11	24.52	77.29	12.60	31.64	78.37	37.26	36.19	16.92
Georgia	206.65	36.68	66.12	102.03	119.51	342.65	-15.52	147.17	39.37	21.25	114.39
Kazakhstan	-26.33	-10.75	-11.69	246.56	-14.16	-1.66	32.18	-25.54	-23.51	-17.94	-32.46
Kyrgyzstan	-30.68	57.32	-17.16	64.64	-18.04	38.55	-43.59	-12.87	-2.62	-33.08	-15.36
Mongolia	220.06	44.24	-41.98	64.90	66.06	-61.33	-25.49	-19.33	-1.17	-5.60	-59.66
Tajikistan	49.33	13.19	7.56	14.89	40.60	10.63	-3.67	14.40	8.84	-20.23	-2.91
Turkmenistan	37.83	27.82	62.89	314.88	27.64	22.58	35.40	61.25	64.82	-6.13	-0.31
Uzbekistan	30.69	45.88	39.97	64.02	110.27	-11.95	25.07	49.72	37.61	-10.93	18.52
Albania	175.76	36.11	33.21	77.01	58.43	22.57	72.88	55.98	47.55	16.93	50.98
Bosnia and Herzegovina	367.04	39.68	66.31	55.53	87.10	-22.96	56.57	124.56	83.41	18.53	16.33
Bulgaria	29.55	98.24	53.71	59.41	25.14	12.12	58.05	110.19	53.21	23.25	55.07
Croatia	82.99	152.25	29.11	86.87	-17.48	150.42	1.61	81.13	54.71	-14.01	5.12
Czech Republic	16.73	20.77	4.07	23.27	-13.22	-21.82	5.61	46.08	12.47	-24.55	58.44
Hungary	3.45	-23.54	11.40	0.36	-15.51	-14.06	-20.39	39.61	8.91	-17.15	-12.15
Macedonia	53.03	108.89	43.03	49.62	28.31	-30.88	57.82	50.72	20.84	6.33	12.11
Montenegro	14.84	-0.24	20.27	20.33	39.24	14.66	28.69	45.35	13.17	-8.72	46.00
Poland	92.46	20.57	45.30	34.31	11.60	122.52	24.78	105.90	78.18	-10.11	20.44
Romania	107.61	26.68	46.03	76.88	63.09	12.90	36.45	99.49	52.93	3.97	56.07
Serbia	42.77	13.29	28.98	15.70	36.21	20.28	17.42	57.78	45.51	-10.09	58.91
Slovakia	85.33	156.20	16.00	51.35	-0.03	-25.54	61.28	124.05	50.22	36.77	-12.40
Slovenia	60.83	44.84	1.19	31.38	1.62	43.44	19.60	132.49	30.78	-13.03	72.71
Belarus	38.11	241.02	1.33	105.99	49.81	30.38	62.02	129.65	114.95	-12.40	61.90
Estonia	4.81	191.08	20.96	92.60	39.15	45.07	47.57	132.42	97.35	21.36	35.19

Latvia	34.82	216.27	42.13	90.18	40.79	20.15	68.79	128.85	82.86	12.28	28.72
Lithuania	22.86	186.72	11.85	65.44	16.36	-35.00	11.77	136.18	54.21	6.45	58.36
Moldova	48.70	17.29	-0.48	27.28	24.65	-6.95	-4.63	34.64	9.00	-37.28	7.77
Russian Federation	21.43	4.76	13.44	25.80	74.02	3.16	0.72	61.24	76.23	4.22	111.06
Ukraine	164.65	31.79	27.05	120.95	89.24	52.36	126.18	117.04	59.94	-6.51	83.03
Brunei	26.32	58.23	1.35	57.40	71.17	-2.69	156.18	49.59	82.22	12.71	50.22
Japan	26.57	28.31	5.07	45.56	29.89	24.46	92.50	66.17	17.87	-12.75	55.11
South Korea	237.60	102.55	12.97	120.36	766.01	-0.50	337.72	169.07	103.63	-14.59	93.48
Singapore	-9.52	25.68	-16.35	198.50	35.54	-46.55	134.65	64.86	12.71	-22.25	115.06
Australia	13.65	31.92	-20.26	9.18	100.87	-4.05	45.01	28.38	38.48	-30.40	18.06
New Zealand	-2.13	12.10	-25.91	14.65	36.52	-19.87	5.60	31.28	17.74	-13.32	35.41
Andorra	-9.69	16.34	-8.92	29.74	27.51	-7.92	24.42	37.81	8.29	-10.91	27.98
Austria	68.27	-25.69	-9.35	53.53	-16.22	-11.12	-26.09	69.67	40.80	23.64	18.62
Belgium	-3.57	5.70	-15.21	16.34	-6.09	-1.46	12.44	57.94	28.18	-19.42	38.87
Cyprus	-63.57	93.46	23.82	88.91	27.43	-36.65	91.80	59.12	32.82	10.55	113.53
Denmark	-17.89	58.09	-19.13	37.76	41.69	4.23	30.23	50.07	39.66	-25.47	32.52
Finland	62.75	13.66	-18.48	25.62	35.12	-17.24	56.44	50.47	17.77	-17.81	35.44
France	27.93	9.23	-2.78	92.45	4.91	13.81	43.26	42.94	19.81	-17.39	19.59
Germany	19.15	0.76	-14.60	44.15	25.91	-6.22	-10.16	73.72	35.50	-32.17	118.52
Greece	63.09	49.58	9.76	58.73	41.52	1.20	77.78	66.19	59.62	1.26	22.98
Iceland	23.49	26.19	-11.70	25.46	2.34	19.10	-13.36	57.91	32.07	-24.57	25.86
Ireland	23.55	33.93	1.92	87.23	35.72	-36.19	85.48	90.16	23.56	0.08	98.67
Israel	105.92	17.30	10.65	95.54	67.03	-32.48	48.30	64.63	33.25	-20.67	71.66
Italy	62.84	3.63	-19.41	44.22	-4.70	-18.55	26.56	42.92	33.13	-14.81	47.28
Luxembourg	-13.88	1.57	-4.57	76.85	8.66	-11.86	28.48	88.64	36.45	-11.79	46.37
Malta	113.17	28.06	-8.60	107.92	41.65	11.51	90.80	104.00	45.32	-3.62	77.44
Netherlands	33.17	25.52	-2.11	70.63	48.39	-13.86	14.42	38.66	18.87	-4.76	32.04
Norway	-13.49	52.03	-12.30	12.14	38.03	-14.79	9.41	47.98	27.35	0.51	-1.51
Portugal	51.53	9.63	19.93	72.37	11.54	6.26	50.49	114.68	38.57	-23.39	49.90
Spain	28.13	33.50	2.73	84.88	36.17	-10.88	27.21	59.96	25.67	-4.35	33.69
Sweden	1.63	4.66	3.81	-1.71	31.92	-27.91	22.89	24.45	18.37	3.93	-11.60
Switzerland	-44.83	16.77	-13.53	34.72	-11.02	-11.41	-19.48	-0.31	13.90	-19.27	14.61
United Kingdom	-32.31	33.09	-31.47	58.10	46.80	-9.37	6.54	43.71	26.97	-12.29	13.20
Argentina	120.66	-7.60	-15.50	17.67	8.39	70.00	-9.38	20.27	10.73	-16.62	-34.82
Chile	121.52	10.12	26.93	73.88	75.18	-24.60	24.21	53.30	58.33	-7.40	-8.51
Uruguay	39.86	11.29	-3.88	21.80	115.98	18.46	3.24	43.09	43.45	-14.99	-28.62
Canada	34.09	35.19	-10.72	35.52	30.08	-20.58	20.51	31.13	12.57	-18.17	41.03
United States	50.09	2.55	2.43	24.10	50.17	-21.31	-6.73	3.94	19.11	-11.78	-3.95
Antigua and Barbuda	1156.28	-16.24	14.35	331.44	64.96	-37.94	32.36	17.77	18.00	-3.07	-16.65
The Bahamas	341.72	-14.24	13.55	205.46	48.71	-11.40	7.98	12.30	13.41	-7.82	-16.69
Barbados	855.64	-24.08	12.38	370.04	54.15	-13.34	14.09	17.42	19.75	-6.28	-16.35
Belize	840.22	22.65	30.48	270.94	86.92	32.69	-5.73	26.82	37.98	-2.05	-9.22
Cuba	957.83	-23.30	19.01	480.21	69.12	-21.97	647.39	11.85	9.84	-24.77	-12.01
Dominica	757.23	9.18	30.83	289.42	70.53	44.38	2.02	32.10	30.88	18.72	-7.73
Dominican Republic	138.00	-24.45	35.01	154.13	103.08	13.29	134.58	50.63	61.95	3.78	-13.82
Grenada	168.70	-15.66	12.07	280.85	-8.69	-48.99	-41.79	-22.41	25.45	-18.18	-43.30
Guyana	706.92	-6.28	15.15	160.71	71.47	-6.58	92.94	7.56	33.13	-3.57	-22.55
Haiti	80.18	-25.24	8.90	26.67	17.82	-30.66	-26.93	-14.85	8.06	-16.82	-19.45
Jamaica	929.11	-16.95	2.55	212.30	195.91	6.79	13.62	72.41	92.11	63.16	-8.18
Saint Lucia	1229.88	-25.33	3.66	268.87	33.45	-25.90	4.11	-4.53	9.16	-20.90	-32.33
Saint Vincent and the Grenadines	870.58	-14.81	20.68	263.47	43.02	-7.96	112.07	17.65	24.23	-0.87	-20.20
Suriname	796.79	-12.61	22.65	388.73	62.71	-6.73	4.74	6.80	24.38	-11.07	-25.38
Trinidad and Tobago	712.54	-36.87	-3.37	202.61	35.75	-4.55	59.89	-0.31	36.98	-21.13	-32.17
Bolivia	114.50	24.99	19.15	29.23	61.19	-42.20	-48.00	11.27	6.34	-12.18	-16.38
Ecuador	1239.36	27.98	11.78	267.49	316.42	13.13	-8.03	73.48	16.61	26.37	-23.63

Peru	811.39	-4.99	4.60	104.87	119.99	-77.38	-24.52	24.88	-1.11	-7.46	-33.64
Colombia	209.49	29.42	-22.72	29.08	34.49	-16.51	-12.71	15.49	6.59	-23.65	11.93
Costa Rica	717.47	73.02	13.18	127.08	32.50	-11.75	1.76	37.04	30.15	-5.27	14.34
El Salvador	1063.00	38.04	45.75	162.57	6.18	2.46	-31.37	93.74	32.39	-1.26	-18.78
Guatemala	3071.67	14.55	-2.68	214.66	64.39	-17.03	-43.16	56.32	10.01	35.61	-32.16
Honduras	13.40	50.47	41.55	20.72	39.69	40.87	-31.92	36.11	37.06	4.97	-11.86
Mexico	287.48	39.14	10.98	39.85	106.23	20.56	-10.29	51.30	35.51	-2.50	10.50
Nicaragua	1581.53	-0.99	9.67	71.10	127.10	28.79	-18.11	28.02	-0.31	-14.31	-22.78
Panama	1050.72	87.50	-1.14	92.89	84.07	-1.26	63.32	15.63	3.14	-16.03	-2.80
Venezuela	2400.89	-3.83	13.40	287.54	118.07	-13.08	28.50	5.87	9.34	-14.75	-29.53
Brazil	150.11	37.03	4.22	84.00	36.99	-17.50	-12.91	23.14	50.30	-15.56	-8.46
Paraguay	886.74	7.65	25.97	55.97	93.91	40.13	-0.72	48.10	26.35	-5.54	-8.02
Algeria	45.52	44.62	19.24	24.28	122.59	118.12	-2.32	26.15	42.41	-1.12	0.14
Bahrain	85.27	-23.28	-40.90	-8.54	34.55	63.53	-47.11	5.16	-16.34	-35.57	-28.29
Egypt	124.46	42.89	27.62	26.76	130.21	144.27	-15.55	38.33	38.53	-3.38	0.25
Iran	240.87	35.68	55.86	51.00	136.24	166.77	125.66	85.34	61.32	2.28	63.24
Iraq	-46.26	-3.38	-26.63	-21.03	-21.78	-43.30	-66.85	-61.35	-18.90	-36.89	-43.00
Jordan	7.30	48.97	28.96	24.45	-1.80	-33.08	-18.42	3.05	-11.29	-13.33	-18.23
Kuwait	-74.01	5.62	-17.89	31.64	-17.42	77.35	-5.78	12.73	-10.11	-34.41	-1.18
Lebanon	456.58	55.19	26.49	106.94	165.84	9.37	229.64	174.03	43.37	11.62	139.37
Libya	194.76	53.35	40.63	70.43	148.61	222.93	73.76	49.25	28.86	21.16	31.84
Morocco	78.89	49.86	38.51	23.74	86.38	126.98	-11.07	26.71	38.75	-1.02	-0.55
Palestine	74.54	20.64	4.06	-0.48	3.05	-20.61	2.45	-10.43	3.18	-12.80	-9.10
Oman	79.71	81.98	22.34	50.86	93.45	67.95	25.12	39.40	45.01	-2.92	35.13
Qatar	654.90	-44.28	-0.14	26.35	-39.70	105.90	123.77	15.59	31.03	-23.32	30.58
Saudi Arabia	314.61	133.47	69.71	101.30	282.37	81.33	113.70	113.68	84.75	21.21	53.09
Syria	126.66	47.11	65.67	33.91	73.46	4.26	11.17	-16.04	31.55	-16.85	7.22
Tunisia	126.90	25.14	36.76	38.20	106.09	151.52	30.42	31.98	28.03	-10.47	5.98
Turkey	155.20	0.63	20.41	-12.48	89.53	-53.25	18.45	15.83	16.41	-26.23	4.36
United Arab Emirates	345.28	74.02	60.56	106.13	80.59	234.37	41.85	28.84	35.05	5.06	21.59
Yemen	46.89	42.84	39.33	6.76	55.50	95.91	-26.61	1.62	18.41	5.93	13.18
Afghanistan	34.80	10.28	9.58	-6.45	22.75	23.38	-18.69	-9.60	-2.48	3.98	10.15
Bangladesh	296.00	-2.32	-11.82	-7.35	66.94	-56.10	-67.88	-10.63	5.15	-32.35	-15.66
Bhutan	-20.39	21.99	24.27	-8.28	53.65	-13.18	-60.54	13.96	22.86	-19.57	3.49
India	-11.96	51.55	31.31	6.66	82.01	27.97	-46.83	40.20	32.85	-7.48	27.24
Nepal	-50.98	49.87	12.88	-12.89	49.32	-5.19	-55.93	23.74	33.96	-13.41	10.02
Pakistan	12.11	74.51	39.59	36.04	77.97	40.87	-14.40	30.59	24.71	20.02	14.40
Angola	-23.19	18.69	1.08	-1.84	14.17	-24.53	-42.69	-15.12	13.27	-14.65	-15.06
Central African Republic	4.79	19.23	-5.57	16.08	-17.91	-13.11	-9.76	-17.43	-9.39	1.93	-8.20
Congo	-25.70	26.18	16.91	22.36	5.36	-28.90	-31.50	-12.19	0.26	-3.48	-5.69
Democratic Republic of the Congo	8.42	4.54	-11.05	7.00	0.27	-11.06	-19.79	-18.11	-4.17	-2.15	-9.84
Equatorial Guinea	-28.40	51.87	30.43	5.74	61.07	-55.72	-66.76	6.40	42.78	-27.82	-21.98
Gabon	4.36	46.18	22.19	47.10	4.65	-19.59	-25.86	-9.36	6.79	1.58	-5.60
Burundi	-42.82	-10.39	-33.55	-17.31	-7.35	-58.94	-40.54	-33.34	-15.46	-13.27	-27.95
Comoros	-34.90	10.84	-2.18	9.26	23.72	-19.34	-36.25	-20.63	-2.93	-4.26	-18.19
Djibouti	-33.12	42.71	15.87	20.88	36.20	3.18	-25.89	-7.32	4.60	4.93	-14.54
Eritrea	-27.70	36.39	6.46	34.63	46.18	-19.91	-24.66	-3.16	-1.26	16.48	-0.95
Ethiopia	14.93	-16.63	8.53	-26.65	-10.74	-60.42	-50.80	-27.21	-1.97	-14.34	-21.63
Kenya	61.16	29.54	22.19	10.33	39.41	11.99	1.54	22.88	25.66	11.08	14.85
Madagascar	-37.48	4.70	-30.17	-10.13	11.73	-22.57	-36.42	-24.87	-12.01	-10.98	-23.09
Malawi	33.77	7.27	1.24	8.12	30.11	-10.53	-24.82	-1.13	17.65	0.75	10.45
Mauritius	119.74	35.97	-23.73	68.66	66.55	-23.87	62.59	96.53	79.84	-1.90	81.11
Mozambique	2.36	27.31	-18.65	12.93	46.17	-12.32	-33.79	6.11	8.97	-11.35	-9.09
Rwanda	-43.96	-2.07	-24.09	-11.43	6.25	-54.47	-49.99	-31.25	-3.14	-9.35	-22.30
Seychelles	81.68	79.58	19.36	7.20	26.49	-19.85	25.94	99.78	108.54	25.78	-25.60

Somalia	-15.04	18.48	1.72	17.20	24.45	-11.79	0.50	-6.29	-4.65	23.12	1.68
Tanzania	-3.44	39.82	-8.44	13.81	28.37	-10.48	-19.75	-4.48	2.28	14.07	-7.70
Uganda	70.69	51.87	18.96	40.83	57.44	-16.49	-11.97	4.74	17.75	7.89	12.97
Zambia	-16.74	0.81	-10.37	-13.95	-9.49	-4.14	-31.45	-25.49	-10.16	-10.75	-26.63
Botswana	-26.60	46.20	6.77	23.88	49.00	-20.19	-37.09	12.88	19.08	-11.65	-11.37
Lesotho	32.58	94.22	26.31	62.06	36.67	33.00	14.81	33.35	9.28	19.44	19.48
Namibia	-6.06	25.97	0.54	1.82	37.26	-27.44	-38.08	7.75	2.33	-14.88	14.38
South Africa	6.22	18.02	-1.43	15.46	-0.36	3.72	-18.36	9.15	14.51	-12.01	-14.09
Swaziland	22.74	51.36	4.26	36.82	18.65	-7.40	-8.26	5.82	-2.40	-0.03	5.12
Zimbabwe	45.08	41.15	1.65	52.32	24.64	91.59	48.66	60.70	42.92	22.33	42.40
Benin	-51.55	80.99	-25.52	43.35	3.83	-11.67	-31.03	-1.55	42.83	0.03	-12.51
Burkina Faso	-31.98	61.26	-37.56	4.52	-10.29	-40.18	-33.21	-6.02	31.82	-3.95	-16.26
Cameroon	-48.16	53.46	-11.93	60.06	-11.71	-23.08	-25.15	-7.83	23.11	2.37	-11.05
Cape Verde	-18.79	85.11	67.86	30.98	68.39	5.72	-7.23	54.31	78.28	35.78	16.81
Chad	-28.95	77.14	3.44	58.86	-3.34	-28.97	-9.99	10.55	28.53	15.44	0.62
Cote d'Ivoire	-13.79	56.49	19.09	29.02	13.21	-15.23	-19.19	10.68	16.16	7.48	9.52
The Gambia	-23.86	54.27	27.53	55.07	27.78	-8.55	-22.15	16.72	18.68	9.52	10.69
Ghana	19.23	-4.61	-21.36	24.85	25.74	-44.89	-18.67	-22.78	55.09	-25.51	-19.48
Guinea	21.92	37.49	29.73	20.46	21.12	-9.17	-21.68	-1.78	17.98	-0.97	-4.13
Guinea-Bissau	-55.68	61.74	-21.03	36.71	-10.75	-29.24	-33.02	-9.31	33.59	0.03	-11.69
Liberia	-49.64	28.17	-32.52	14.13	-11.91	-19.05	-37.48	-22.45	26.51	-9.59	-23.01
Mali	-24.19	32.61	7.28	17.72	16.05	-55.61	-46.38	-3.46	31.33	-7.64	-4.90
Mauritania	-60.39	54.91	-10.91	44.37	4.97	-50.52	-38.39	-2.12	33.34	3.33	-7.62
Niger	-63.27	17.96	-29.84	7.29	-10.31	-42.73	-40.99	-23.52	24.93	-10.33	-23.03
Nigeria	-47.96	24.29	12.11	53.76	27.44	-39.76	-28.53	3.77	8.66	1.81	4.18
Sao Tome and Principe	-28.18	36.46	59.75	47.06	38.68	15.73	-14.69	17.68	73.44	8.41	11.14
Senegal	-55.09	54.06	-10.05	47.75	-0.76	-5.78	-21.94	-2.04	37.01	9.89	-6.08
Sierra Leone	-48.57	55.92	-25.56	44.11	9.52	23.63	-27.47	-4.49	40.81	1.24	-13.66
Togo	-39.43	49.66	-27.09	38.45	-8.81	-26.50	-28.70	-11.24	33.75	-4.65	-17.25
American Samoa	224.16	3.10	56.23	33.48	58.80	38.25	17.23	44.20	43.14	-7.58	28.56
Bermuda	423.25	-43.90	-3.23	444.46	23.51	-59.10	226.07	7.03	-6.53	-29.48	7.33
Greenland	-50.03	9.15	-5.37	-12.01	45.56	-9.52	-50.26	-26.49	-4.83	-27.27	-12.98
Guam	250.77	8.53	59.04	55.59	124.21	58.88	36.11	18.92	20.50	5.12	23.45
Northern Mariana Islands	275.62	-0.18	44.26	33.22	44.35	-1.96	60.64	53.01	12.21	7.48	60.84
Puerto Rico	1147.16	-8.97	11.17	270.77	-3.10	-13.30	81.79	30.82	22.13	-16.54	2.19
Virgin Islands, U.S.	153.14	19.57	37.99	175.82	99.82	25.32	117.72	45.90	40.95	1.05	13.23
South Sudan	-14.24	32.43	-2.45	22.40	23.55	2.57	-3.61	-7.00	-1.33	17.05	-4.78
Sudan	33.50	30.01	2.25	-6.48	47.92	95.72	-33.16	-1.09	21.55	-5.07	1.24
